# Molecular Systematics of *Valerianella* Mill. (Caprifoliaceae): Challenging the Taxonomic Value of Genetically Controlled Carpological Traits

**DOI:** 10.3390/plants11101276

**Published:** 2022-05-10

**Authors:** Itziar Arnelas, Ernesto Pérez-Collazos, Josefa López-Martínez, Juan Antonio Devesa, Pilar Catalán

**Affiliations:** 1 Departamento de Botánica, Ecología y Fisiología Vegetal, Universidad de Córdoba, E-14071 Córdoba, Spain; 2 Departamento de Ciencias Biológicas y Agropecuarias, Universidad Técnica Particular de Loja, CP 11-01-608 Loja, Ecuador; 3 Departamento de Ciencias Agrarias y del Medio Natural, Escuela Politécnica Superior de Huesca, Universidad de Zaragoza, E-22071 Huesca, Spain; ernextop@unizar.es; 4 Grupo de Bioquímica, Biofísica y Biología Computacional (BIFI, UNIZAR), Unidad Asociada al CSIC, E-50059 Zaragoza, Spain; 5 Departamento de Biología Vegetal, Ecología y Ciencias de la Tierra, Universidad de Extremadura, E-06006 Badajoz, Spain

**Keywords:** AFLP, carpological traits, genetic structure, phylogeny, molecular systematics, plastid phylogeny, taxonomy, *Valerianaceae*

## Abstract

*Valerianella* (cornsalad) is a taxonomically complex genus formed by 50–65 annual Holarctic species classified into at least four main sections. Carpological traits (sizes and shapes of achenes and calyx teeth) have been used to characterize its sections and species. However, the potential systematic value of these traits at different taxonomic ranks (from sections to species (and infraspecific taxa)) has not been tested phylogenetically yet. Here, we have assessed the evolutionary systematic value of *Valerianella* diagnostic carpological traits at different hierarchical ranks and have demonstrated their ability to separate taxa at the sectional level but not at species level for species of several species pairs. A total of 426 individuals (17 species, 4 sections) of *Valerianella* were analyzed using AFLP and plastid data. Genetic clusters, phylogenetic trees, and haplotype networks support the taxonomic classification of *Valerianella* at the four studied sectional levels (*V*. sects. *Valerianella*, *Cornigerae*, *Coronatae*, *Platycoelae*) but show admixture for ten taxa from five species pairs (*V. locusta—V. carinata, V. coronata—V. pumila, V. multidentata—V. discoidea, V. dentata—V. rimosa*, *V. eriocarpa—V. microcarpa*), which are not reciprocally monophyletic. Dating analyses indicate that the *Valerianella* sections are relatively old (mid-Miocene), while most species diverged in the Pliocene–Pleistocene. A new section *Valerianella* sect. *Stipitae* is described to accommodate the highly divergent and taxonomically distinct *V. fusiformis* type species. Taxonomic treatments that recognize the sectional ranks and that subsume the separate species of each species pair into single species represent a natural classification for *Valerianella*.

## 1. Introduction

Molecular systematics has revolutionized the taxonomic circumscriptions of many plant groups. Genetically supported phylogenies have provided evolutionary frameworks for a natural classification of the angiosperms and for a critical revision of some morphological traits used to identify species and other taxonomic ranks [1]. The cornsalad genus *Valerianella* Mill. comprises 50 [2,3] to 65 species [4] distributed mainly in the temperate regions of the northern hemisphere and classified into at least four main sections [2]. Many of them (c. 39) are present in the Mediterranean region, a probable center of origin of the genus [5]. Up to 11 species have been recorded from the Western Mediterranean region [6], including some of the taxonomically most complex groups of closely related species pair aggregates with a specific genetic control system in their diagnostic fruit characters [7,8]. In Flora Europaea, the classification of the separate *Valerianella* species from each of these species pairs was based solely on a few carpological features, such as inflated vs. uninflated sterile locules of the achene and the presence vs. absence (or sizes) of the calyx teeth [9], while the other reproductive and vegetative traits of the species were the same. The discovery of the genetic regulatory mechanism of the fruit morphotype led some taxonomists to postulate that these phenotypes resulted from the co-segregation of alleles that did not represent a speciation process and, therefore, the two taxa of each species pair should be treated as the same species [6,10]. 

*Valerianella* taxa are annual, self-pollinating plants displaying small flowers with slightly colored corollas, little or no scent, low pollen production, and most likely no nectar [8,11]. Species recognition has traditionally been based on fruit characteristics, as flowers are similar across taxa. In contrast, fruits are highly polymorphic, and both homocarpy and heterocarpy (the production of two or more types of fruit by the same individual) have been detected between and within several taxa [6,7,8,10,11,12,13,14]. Furthermore, fruit polymorphism can occur within populations, where each individual plant produces only one type of fruit but two or more fruit morphotypes are present within populations [7]. The fruits have three locules, one fertile and two sterile, with unequal development in the different species, which can sometimes culminate in a more or less developed, persistent calyx [6,15,16,17]. Fruit traits have been used as the main diagnostic features for the delineation of sectional boundaries in the genus and for the description of infrageneric taxa [2,6,10,15,16,18,19,20,21,22,23,24,25].

Although fruit morphology is a diagnostic character of indisputable value in the taxonomy of *Valerianella*, its use alone in the circumscription of new taxa has given rise to several uncertainties [9] and the description of spurious taxa (e.g., *V. cupulifera* Legrand). Martin and Mathez [8], in a pioneer genetic study of Mediterranean species of *Valerianella*, detected the existence of a genetically controlled fruit polymorphism system that caused the coexistence in the same population of individuals with two different types of fruits. A similar fruit morphotype genetic regulatory system was found in other self-pollinating genera of the Valerianoideae (e.g., *Fedia*, [26]) and was also attributed to some American *Valerianella* [7] and *Plectritis* [27] taxa. 

Inheritance patterns of fruit morphotype deduced for the *V. coronata–V. pumila* complex was also suspected to be found in other species pair groups of Mediterranean *Valerianella* species, namely in *V. locusta–V. carinata* and *V. dentata–V. rimosa* [6,8,10]. Based on these hypotheses and the observed occurrence of the different species pairs in sympatric areas, Devesa et al. [10] and Devesa and López [6] proposed new specific re-circumscriptions of these groups. Therefore, *V. coronata* and *V. pumila*, previously treated as separate species, were classified as two forms of *V. coronata* within *V*. sect. *Coronatae*, *V. locusta* and *V. carinata* as two forms of *V. locusta* subsp. *locusta* within *V*. sect. *Valerianella*, and *V. dentata* and *V. rimosa* as two forms of *V. dentata* within *V*. sect. *Platycoelae*.

The phylogenetic studies conducted on the Valerianoideae have demonstrated that the diploid *Valerianella* and its derived allotetraploid *Fedia* taxa form a monophyletic group within a large paraphyletic lineage of *Valeriana* [5,28,29,30,31,32]. Evolutionary analyses within *Valerianella* have shown the close relationships between the genotypes of some of the species pairs mentioned [33,34], indirectly supporting their respective common ancestors and the convenience of classifying them at the infraspecific level within each group, leading to the new taxonomic treatment proposed for *V. locusta*, *V. coronata*, and *V. dentata* by [6,10]. However, exhaustive population-level evolutionary studies within and between these *Valerianella* species pair complexes and other related taxa have yet to be conducted, questioning how widespread the genetic regulatory mechanism of the *Valerianella* fruit morphotype might be across different taxonomic ranks and time scales and whether it might have affected speciation processes within these complexes. Conserved plastid DNA sequences have provided useful information to reconstruct the evolutionary history of the Valerianoideae and their lineages [5,28,29,31,32,35,36,37]. Highly variable nuclear AFLP markers have proven their value in characterizing the genetic structure and relationships of many plant groups [38,39] and, in particular, among representatives of the Valerianoideae [33,34,40].

In order to reconstruct the genetic and evolutionary relationships between the populations of five *Valerianella* species pairs within a large suprageneric phylogenetic framework and to investigate the phylogenetic value of the diagnostic carpological traits of the *Valerianella* sections and species (Table 1) and the number of times that the mechanism of genetic regulation of fruit morphotype has evolved along *Valerianella* lineages, we have performed a large evolutionary and population genetic study of *Valerianella* species based on plastid *trn*T-L and *trn*L-F DNA sequences and nuclear AFLP data. Our study aimed to: (i) reconstruct the evolutionary relationships of *Valerianella* species and sections, using plastid data from a representative sample of Western Mediterranean and American taxa; (ii) estimate the times of divergence and origins of its main lineages; (iii) analyze genetic relationships and the role of genetic isolation in shaping the morphological differentiation of populations; (iv) elucidate the systematic value of carpological traits in sectional and specific taxonomic ranks; and (v) evaluate the evolution of the inheritance system of the fruit–calyx morphotype both at the population level and at the species level of the *Valerianella* species pairs studied. Our approach is based on the assumption that if the diagnostic carpological traits that taxonomically separate species from each species pair are exclusively controlled by co-segregating genotypes of two linked loci, sympatric populations of the two morphs would be genetically and evolutionarily closer to each other than populations of the same morph that are geographically separated. Conversely, if the diagnostic carpological features that characterize each species derive from the expression of other genes, similar morphotypes from distant populations would share a common ancestor, thus representing a single speciation event. 

## 2. Results

### 2.1. Phylogeny of Valerianella Based on Plastid Data 

**Figure 1:**
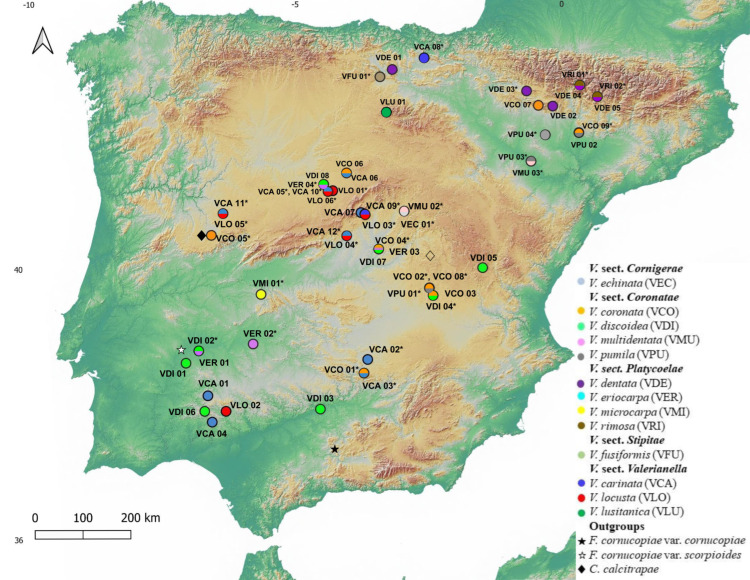
Geographical distributions of the studied populations of the *Valerianella* species, *Fedia cornucopiae* var. *cornucopiae* (white star) and var. *scorpioides* (black star) and *Centranthus calcitrapae* (diamonds) in the Iberian Peninsula. Color codes of *Valerianella* species are indicated in the chart.

To test our evolutionary hypotheses, we reconstructed a largely sectional and population-level sampled phylogeny of *Valerianella sensu lato* (diploid *Valerianella* clade plus its derived allotetraploid *Fedia* lineage) using 51 individuals from the Western Mediterranean and American regions corresponding to 16 *Valerianella* species, which covered the four main sections of the genus, and two species of *Fedia* (Figure 1, Table S1). Maximum likelihood (ML) phylogenetic analysis and Bayesian phylogenetic dating (BEAST) analysis of the plastid *trn*L-F and *trn*T-L data (Table S1) were performed using also sequences of the close core Valerianoideae *Centranthus*, *Valeriana*, and *Plectritis* and of less related Dipsacales *Nardostachys*, *Patrinia*, and *Triplostegia* species as outgroups (Table S1; see Section 4).

**Figure 2:**
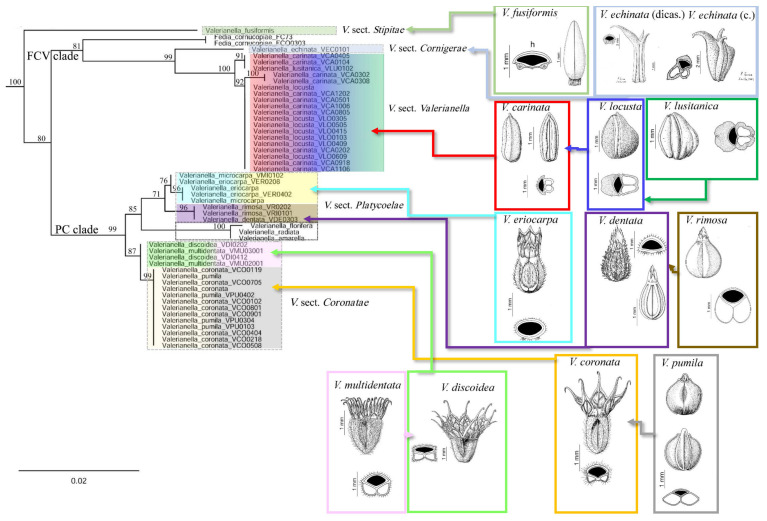
Maximum likelihood plastid *trn*L-F tree constructed with IQTREE showing the relationships between the *Valerianella* and *Fedia* species studied. Dipsacaceae sequences were used to root the tree (see Figure S1). FCV (*Fedia* + *V*. sect. *Valerianella* clade), PC (*V*. sects. *Platycoelae* + *Coronatae* clade). Numbers on branches indicate UltraFast Bootstrap support (BS) values. The icons show the different types of carpological traits (fruit and vestigial calyx) of the *Valerianella* species studied (reprinted and adapted with permission from Editorial Board from [6], Copyright 2007, Real Jardín Botánico de Madrid-CSIC).

**Figure 3:**
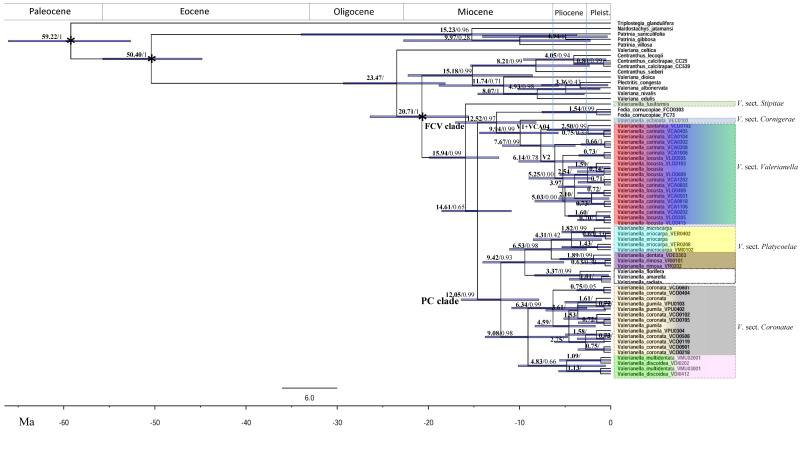
BEAST maximum clade credibility (MCC) tree based on *trn*L-F plastid data showing the estimated mean nodal ages of the *Valerianella* and *Fedia cornucopiae* lineages studied. Asterisk represents the imposed calibrations (see text). Numbers above the branches indicate the estimated ages (in bold) and the Bayesian posterior probability support (PPS) values (plain text). Bars represent 95% highest posterior density (HDP) intervals. Main clades: FCV (*Fedia* + sect. *Valerianella*), PC (sect. *Platycoelae* + sect. *Coronatae*).

Optimal ML (Figure 2 and Figure S1) and Maximum Clade Credibility (MCC) BEAST (Figure 3) phylogenetic trees based on *trn*L-F data only, and the best ML tree (Figure S2A,B) based on *trn*T-L + *trn*L-F combined data, retrieved highly supported and consistent topologies for the major *Valerianella* lineages. Both ML and BEAST *trn*L-F trees showed the early split of *V. fusiformis*, traditionally classified within *V*. sect. *Platycoelae*, from the rest (0.99 posterior probability support (PPS); 100% bootstrap support (BS)), followed by the strongly to well-supported divergences of the *Valerianella* + *Fedia* clade (0.99 PPS; 100% BS), which in turn showed the split of the sister FCV (*Fedia* and *V*. sects. *Cornigerae* + *Valerianella*) (0.97 PPS; 94% BS) and PC (*V*. sects. *Platycoelae* + *Coronatae*) (0.99 PPS; 72% BS) subclades (Figure 2, Figure S1 and Figure S2). The better-rooted ML and BEAST *trn*L-F topology recovered the early split of *V. fusiformis*, traditionally classified within *V*. sect. *Platycoelae*, from the rest (0.99 PPS) (Figure 2, Figure 3 and Figure S1), while the ML *trn*T-L + *trn*L-F tree placed it as a sister to the PC clade but with less support (72% BS; Figure S2B). Within the FCV clade, the BEAST and ML analyses recovered a sister relationship for the *Fedia* and *V*. sect. *Cornigerae* + *Valerianella* lineages (0.97 PPS, 94% BS), followed by the split of the *V*. sect. *Cornigerae* (*V. echinata*) and *V*. sect. *Valerianella* lineages (0.99 PPS, 98% BS) (Figure 2, Figure S1 and Figure S2). The strongly supported *V*. sect. *Valerianella* clade (0.99 PPS, 100% BS) showed mixed resolution of samples from the species pair *V. carinata* and *V. locusta* (and its close taxon *V. lusitanica*, also treated as a subspecies of *V. locusta*; [6,10]). The two topologies supported the divergence of the sister lineages V1 and V2 (0.99, 0.78 PPS; 89%, 98% BS), the first containing samples of *V. carinata* and *V. lusitanica* from the W Iberian Peninsula and the second showing the well to poorly supported splits of *V. carinata* and *V. locusta* samples from the NW, Central, and NE Iberian Peninsula (Figure 2, Figure S1 and Figure S2). Within the PC clade, the two analyses revealed a strongly supported sister relationship for the *V*. sect. *Platycoelae* (0.93 PPS; 100% BS) and *V*. sect. *Coronatae* (0.98 PPS; 100% BS) clades. *Valerianella* sect. *Platycoelae* in turn was divided into Old World (0.98 PPS; 100% BS) and New World (American *V. florifera* / (*V. amarella*, *V. radiata*) species) (0.99 PPS; 100% BS) well-supported clades. The Old World clade consisted of two sister lineages of strongly supported species pairs, the *V. microcarpa–V. eriocarpa* (0.99 PPS; 98% BS) and *V. dentata–V. rimosa* (0.99 PPS; 100% BS) clades. Although less largely sampled, both clades showed an admixture of species samples from each pair in each group (Figure 2, Figure S1 and Figure S2). *Valerianella* sect. *Coronatae* included two other species pair sister lineages, the *V. coronata–V. pumila* (0.99 PPS; 100% BS) clade with strong support and the *V. discoidea–V. multidentata* (0.66 PPS; 97% BS) clade with relatively good support. These lineages also showed the admixture of samples from each of the two species of each pair (Figure 2, Figure S1 and Figure S2). The topology of the combined *trn*T-L + *trn*L-F ML tree (Figure S2A,B) was similar to that of the ML and BEAST *trn*L-F trees, except for the position of *V. fusiformis*. In the *trn*T-L + *trn*L-F ML tree, this lineage was resolved as sister to the *V*. sects. *Coronatae* + *Platycoelae* clade but with low to moderate support (55% and 72% BS without and with indels, respectively; Figure S2A,B). The exchange position of *V. fusiformis* in the *trn*L-F and *trn*T-L + *trn*L-F trees was caused by homoplasies in the *trn*L-F and *trn*T-L characters (Figure 2, Figure 3 and Figure S2); however, in both analyses, *V. fusiformis* fell apart from its co-sectional *V.* sect. *Platycoelae* taxa and these divergences received high support. 

The inspection of the plastid DNA data set (*trn*T-L + *trn*L-F) revealed 36 informative indels for different lineages of the *Valerianella sensu lato* clade and *C. calcitrapae* when they were mapped onto the ML *trn*L-F tree (FCV clade 7, *V*. sect. *Cornigerae* 1, *V*. sect. *Valerianella* 1, PC clade 3, PC clade except *V. multidentata* and *V*. *discoidea* subclades 1, *V*. sect. *Coronatae* 3, *V*. sect. *Platycoelae* 1, *V. dentata–V. rimosa* 1, *V. discoidea–V. multidentata* 1, all species except *V*. sect. *Coronatae* 1, *F. cornucopiae* 1, all species except *F. cornucopiae* 7¸ *F. cornucopiae* + PC clade (except VCO05) + *V*. *fusiformis* 1¸ *C. calcitrapae* 2, all species except *C. calcitrapae* 2, *C. calcitrapae* + *F*. *cornucopiae* + *V*. *fusiformis* + PC clade 1, *C. calcitrapae* + *F. cornucopiae* + *V*. sect. *Cornigerae* + PC clade 1) (Figure S2B, Table S2). 

### 2.2. Divergence Time Estimation of Valerianella Lineages

To infer the ages of the different *Valerianella* lineages and the times of origin of the sectional diagnostic carpological traits and fruit morphotypes of species pairs, divergence times for all the MCC BEAST clades were estimated using the Bayesian search and imposing secondary calibrations for the ancestors of Dipsacoideae + Valerianoideae, Valerianoideae, core Valerianoideae (*Plectritis*, *Centranthus*, *Valeriana*, *Valerianella*, and *Fedia*), and *Valerianella* + *Fedia* following [36].

According to the BEAST chronogram, the diversification of the Valerianoideae could have started in the early Miocene at 20.7 ± 11.1 Ma (Figure 3 and Figure S2), and the split of *V. fusiformis*, a species phenotypically distinct from all its congeners (Figure 3, Table 1), from the *Valerianella* + *Fedia* clade could have occurred in the mid-Miocene at 15.9 ± 10.7 Ma. Within the FCV clade, the split of *Fedia* from *Valerianella* sects. *Cornigerae* + *Valerianella* was dated to the mid-Miocene at 12.5 ± 8.8 Ma. These two sections of *Valerianella*, *Cornigerae*, and *Valerianella*, characterized by their unique carpological traits (Table 1), were estimated to have diverged from their common ancestor at 9.9 ± 8.6 Ma, while the diversification of the admixed lineages of *V*. sect. *Valerianella* (*V. carinata* and *V. locusta*–*V. lusitanica*) was estimated to have occurred in the late Miocene, 7.6 ± 7.9 Ma. The most recent sectional splits date from the late Miocene (e.g., V2, 6.1 ± 8 Ma) to the late Pliocene (e.g., V1, 2.5 ± 6.9 Ma) (Figure 3 and Figure S2). 

The origins of the PC clade and the *V*. sect. *Platycoelae* and *V*. sect. *Coronatae* subclades, also characterized by their unique carpological features (Table 1), were dated to the mid-late Miocene at 12.0 ± 7.6, 9.4 ± 8.8 Ma and 9.0 ± 9.0 Ma, respectively. Diversifications of the phenotypically admixed *V. coronata–V. pumila* lineages were estimated to have occurred in the late Miocene (6.5 ± 8.4 Ma), and those of the also admixed *V. multidentata–V. discoidea* and *V. microcarpa–V. eriocarpa* lineages in the early Pliocene at 4.8 ± 9.6 Ma and 4.3 ± 7.5 Ma, respectively. North American *Valerianella* species were inferred to have diverged in the mid-Pliocene, 3.3 ± 8.0 Ma (node l), and the admixed *V. dentata–V. rimosa* lineages in the Pleistocene (1.8 ± 5.3 Ma, node n) (Figure 2 and Figure S2). 

### 2.3. Plastid Haplotype Network of Valerianella

**Figure 4:**
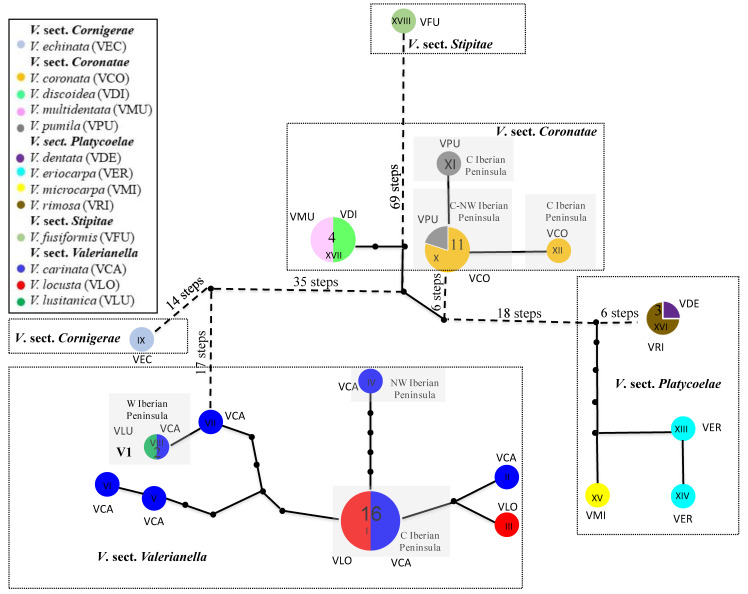
*Valerianella trn*L-F + *trn*T-L plastid haplotype parsimony network. Mutation steps are represented by dots (numbers of mutation steps are indicated within parentheses). Color codes of *Valerianella* species are indicated in the chart.

To investigate the extent of maternal haplotype exchange between species of each of the studied *Valerianella* species pairs, we built a plastid haplotype network using concatenated plastid *trn*T-L + *trn*L-F sequences from the more exhaustively studied Western Mediterranean samples (Figure 1; Table S1). The statistical parsimony haplotypic network of *Valerianella* was fully resolved (Figure 4); it showed no internal loops, suggesting the absence of introgressions between these diploid species. The analysis detected a total of 18 haplotypes and the network topology was highly consistent with that of the ML and BEAST plastid trees (Figure 2, Figure 3, and Figure S2). The highly divergent haplotype XVIII, characteristic of *V. fusiformis*, separated by 69 steps from the rest. The network showed the divergences of four main groups within the remaining haplotypes, corresponding to each of the four *Valerianella* sections (Figure 4). Haplotypes of *V*. sect. *Coronatae* occupied the central part of the network. The most common haplotype, X, was shared by 11 *V. coronata* and *V. pumila* samples, while its derived XI and XII haplotypes were unique to one *V. pumila* and one *V. coronata* sample each. Haplotype XVII, separated from the previous group by eight steps, was shared by four samples of *V. multidentata* and *V. discoidea* (Figure 4). A subnetwork of four *V*. sect. *Platycoelae* peripheral haplotypes separated from the central haplotypes by 18 steps. Haplotype XVI was shared by three *V. rimosa* and *V. dentata* samples, whereas the remaining three haplotypes were unique to *V. eriocarpa* (XIII, XIV) and *V. microcarpa* (XV) samples (Figure 4). Another peripheral subnetwork of nine *V*. sects. *Cornigerae* and *Valerianella* haplotypes was separated from the central group by 35 steps. *V echinata* (*V*. sect. *Cornigerae*) presented a single haplotype (IX) isolated by 31 steps from those of *V*. sect. *Valerianella*. Within the latter group, the most frequent haplotype, I, was shared by samples of *V. carinata* and *V. locusta* from the Central Iberian Peninsula, while haplotype IV (*V. carinata*) was distributed in its NW range and haplotypes VII (*V. carinata*) and VIII (shared by two samples of *V. carinata* and *V. lusitanica*) in its W range (Figure 4). 

### 2.4. Genetic Diversity and Structure of Valerianella Populations

**Figure 5:**
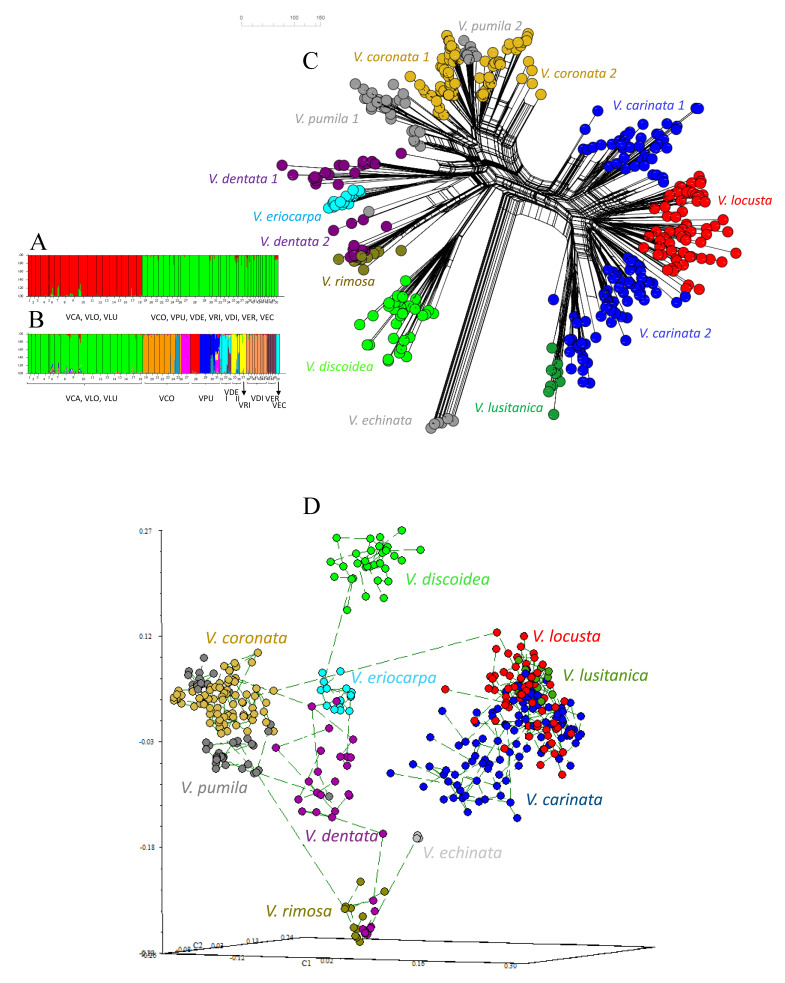
Genetic structure and relationships among 414 individuals of the *Valerianella* species studied based on total genomic AFLP data. STRUCTURE Bayesian inference analysis for the best *K* =2 (**A**) and *K* = 10 (**B**) hypothetical populations. Neighbor joining network topology (**C**). Three-dimensional PCoA plot with superimposed MST showing the genetic relationships among the 414 individuals of *Valerianella* (**D**). *V*. Sect. *Valerianella* (*V. carinata* (VCA), *V. locusta* (VLO), *V. lusitanica* (VLU)); *V*. sect. *Coronatae* (*V. coronata* (VCO), *V. pumila* (VPU), *V. discoidea* (VDI)); *V*. sect. *Platycoelae* (*V. dentata* (VDE), *V. rimosa* (VRI), *V. eriocarpa* (VER)); *V*. sect. *Cornigerae* (*V. echinata* (VEC)). Color codes of *Valerianella* species correspond to those indicated in Figure 1.

To analyze the genetic structure and diversity of the sections and species pairs of *Valerianella* studied, nuclear AFLP analysis was performed on the most extensively sampled Western Mediterranean populations (Table S1). Two out of the ten AFLP primer–pair combinations (E-ACA/M-CTG and E-ACG/M-CAT) assayed generated 230 reliable bands, of which 221 were polymorphic (96.09%). At the sectional level, the amount of genetic diversity measured by the percentage of polymorphic loci (*PLP*), within species (*hsp)* and among species (*Hsp*) Bayesian diversity, and Nei’s genetic diversity (*h*) was relatively equivalent for *V*. sect. *Valerianella* (PLP = 40.7; *Br* = 1.221; *Hsp* = 0.150; *h* = 0.789), *V*. sect. *Coronatae* (PLP=38.8; *Br* = 1.221; *Hsp* = 0.128; *h* = 0.844), and *V*. sect. *Platycoelae* (PLP = 24.5; *Br* = 1.176; *Hsp* = 0.165; *h* = 0.603), while the values for *V*. sect. *Cornigerae* could not be compared with those of the other sections since it only contained a single population (Table 2). Within sections, the genetic diversity parameters of taxa of each species pair were also equivalent when they had similar sample sizes (e.g., *V. carinata* (PLP = 55.8; *Br* = 1.267) vs. *V. locusta* (PLP = 53.6; *Br* = 1.261); *V. coronata* (PLP = 33.9; *Br* = 1.188) vs. *V. pumila* (PLP = 38.8; *Br* = 1.186)). In other species, the values varied, although it depended mainly on the size of the sampling (Table 2). Unique AFLP fragments were present in almost all the taxa studied, from one in *V. eriocarpa* to seven in *V. carinata,* although only *V. echinata* presented diagnostic fragments (*fd* = 8), fixed in all individuals of its single population (Table 2). Similar rank values were found in the genetic diversity parameters at a population level for populations of close sizes (Table S3). Almost all populations of each species pair presented relatively high to very high Nei’s *h* genetic diversity values (0.650–1.000), except for three populations that were less variable and did not present unique fragments (*V. rimosa* VEC01, 0.415; *V. lusitanica* VLU01, 0.323; *V. echinata* VEC01, 0.048) (Table S3). Analysis of the genetic structure of the Iberian populations of *Valerianella* revealed a higher probability for two genetic groups (*K* = 2), one that includes all *V*. sect. *Valerianella* populations and the other populations of the remaining sections (Figure 5A). The next best probability was for *K* = 11 genetic groups showing (i) a single group for *V*. sect. *Valerianella* (VCA, VLO, VLU); (ii) six groups within *V*. sect. *Coronatae*, five corresponding to the species pair *V. coronata* (three groups)—*V. pumila* (two, one of them showing admixture between VCO and VPU in some individuals), and one to *V. discoidea* (VDI); (iii) three groups for the *V*. sect. *Platycoelae* species pair *V. dentata* (one, VDE, plus another group shared with VRI)—*V. rimosa*, and one for *V. eriocarpa* (VER); (iv) one group for *V*. sect. *Cornigerae V. echinata* (VEC) (Figure 5B). Separate Bayesian genetic structure analysis detected genetic substructuring within each section and a few species pairs (Figure S3A–C). Interestingly, the analysis detected three genetic groups within *V. carinata–V. locusta* that were geographically structured; a first group joined populations of *V. carinata* from SW-C Spain with *V. lusitanica* (*V. locusta* subsp. *lusitanica*), a second group populations of *V. carinata* from Central and NE Spain, and a third group populations of *V. locusta* from across Spain. Geographical genetic substructuring was also detected within the *V. coronata–V. pumila* species pair, with some populations of *V. pumila* from Central Spain grouping together with populations of *V. coronata* from all over Spain, while populations of *V. pumila* from NE Spain were merged into a separate group (Figure S3B,C). In contrast, the genetic structure detected within the *V. dentata–V. rimosa* species pair was not connected to geography; a population of *V. dentata* from NE Spain formed a group with two populations of *V. rimosa* from the same site and nearby, while other populations of *V. dentata* from NE Spain formed a second group with a population from N Spain (Figure S3D). Analysis of Molecular Variance (AMOVA) revealed that 70.13% of the variability resided among populations and among taxa and 29.87% within populations.

### 2.5. Genetic Relationships of Valerianella Populations

To assess our evolutionary hypotheses using nuclear data, we framed the genetic relationships between Iberian populations of *Valerianella* through neighbor joining network (NJ network) analysis and principal coordinate analysis with Minimum Spanning Tree superimposed on it (PCoA-MST). 

The NJ network topology obtained from the analysis of all individuals (Figure 5C) showed high congruence with the plastid tree topology for all four *Valerianella* sections and for their respective species pairs (Figure 2 and Figure 3). The AFLP network showed the isolation of *V.* sect. *Valerianella* samples from the rest and from the population of *V. echinata* (*V*. sect. *Cornigerae*) (Figure 5C). Within *V.* sect. *Coronatae, V. discoidea* samples did not cluster close to those of *V. coronata–V. pumila*, while, within *V*. sect. *Platycoelae*, all sectional samples were closely related to each other, although the *V. dentata–V. rimosa* group also included *V. eriocarpa* (Figure 5C and Figure S3). Independent NJ network analysis of *V.* sect. *Valerianella* retrieved geographical genetic patterns similar to those observed in the STRUCTURE groups (Figure S3E–G), although it additionally showed the clustering of SW Iberian *V. carinata* populations with *V. lusitanica* and the separation of Central Iberian *V. carinata* populations into two genetic groups, each related to SW–central and to central *V. locusta* populations, respectively (Figure S3E). The independent NJ network relationships inferred for the species pairs *V. coronata–V. pumila* and *V. dentata–V. rimosa* (Figure S3F,G) agreed with the geographical structuring retrieved from the respective genetic structure analyses (Figure S3B–D). The 3D PCoA plot recovered similar genetic relationships for the Iberian *Valerianella* samples as those uncovered by the NJ network topology (Figure 5C), although the MST connected *V. echinata* with the *V. dentata–V. rimosa* group from NE Spain instead of with the *V*. sect. *Valerianella* group (Figure 5D).

### 2.6. Genetic Divergence and Differentiation of the Sectional Carpological Traits of Valerianella

To test the potential effect of long-term genetic divergence between populations on the morphological variation of *Valerianella* carpological traits, we conducted a distance-based redundant analysis (dbRDA) with randomization. Genetic differentiation between populations, estimated using pairwise *F_ST_* values from AFLP data, was tested against the morphological PCo scores of the populations obtained from a principal coordinate analysis (PCoA) performed with the carpological traits scored as binary characters (Table 1). dbRDA analyses could be only performed at the sectional level, for which up to six diagnostic characters could be analyzed, but not at species pair level due to a lack of sufficient diagnostic traits. The dbRDA results revealed that the genetic divergence of populations explained the morphological differentiation of their sectional carpological traits (57.28% of the variation, *p* = 0.001; Table 3).

## 3. Discussion 

### 3.1. Molecular Systematics Supports the Sectional Classification of Valerianella s.l. and the Value of Its Diagnostic Carpological Traits

Our plastid and nuclear phylogenetic analyses have generated congruent topologies and provided a robust evolutionary scenario for the divergence of the *Valerianella* + *Fedia* lineages. The plastid tree and the nuclear network reveal the clear split of the *V.* sects. *Valerianella* + *Cornigerae* and *V.* sects. *Coronatae* + *Platycoelae* clades and also support the isolation of each of the four *Valerianella* sections (Figure 2, Figure 3, and Figure 5). Our more largely sampled plastid topology also recovers the sister relationship of *Fedia* with *V*. sects. *Valerianella* + *Cornigerae* and the nesting of North American *Valerianella* species within its *V*. sect. *Platycoelae* clade (Figure 2 and Figure 3). Our results generally agree with previous evolutionary studies of Valerianaceae [35] and partially disagree with those of *Valerianella* [33,34] and *Valerianella* plus *Fedia* [5]. However, all the studies of valerians and cornsalads coincide in the nesting of the polyploid *Fedia* clade within the large diploid *Valerianella* clade [5,28,30,31,32,35,36,41], forming a natural group described as subtribe Fediinae Graebn. *sensu* Weberling [42]. The morphological similarities between the two genera refer to their annual habit and type of inflorescence [43] and the genetic polymorphism of the fruit [11,26], while they differ in the presence of three stamens in *Valerianella* and two in *Fedia* [6,44]. Our widely sampled phylogeny of cornsalads allows us to hypothesize that the polyploid ancestor of *Fedia* was probably derived from a diploid ancestor of *V*. sects. *Valerianella* + *Cornigerae* (Figure 2 and Figure 3).

The strong monophyly and isolation revealed for each of the four main sections of *Valerianella* in the ML and Bayesian plastid trees (Figure 2 and Figure 3) and the statistical parsimony haplotypic network (Figure 4) are corroborated with the same topology obtained from a larger sampled population analysis in the nuclear neighbor-net AFLP tree (Figure 5C). The only exception is the more divergent position of *V. discoidea* with respect to its co-sectional *V. coronata–V. pumila* group in the AFLP network than in the plastid tree (Figure 2, Figure 3 and Figure 5), although the *V. discoidea–V. multidentata* haplotype XVII was also differentiated from haplotypes X, XI, and XII of *V. coronata–V. pumila* by 10 steps in the plastid haplotype network (Figure 4). However, these two species pairs of *V*. sect. *Coronatae* resolve as sister lineages with high support in the plastid topologies (Figure 2 and Figure 3). 

The four sectional lineages are morphologically differentiated by their unique carpological traits (Figure 2, Table 1), although they also share some fruit features that relate them to each other [6]. Thus, *V*. sect. *Cornigerae*, represented in our study by the circum-Mediterranean species *V. echinata*, has heterocarpic plants and is characterized by its persistent calyx in the fruit, consisting of three well-developed horn-like teeth; in contrast, Mediterranean and Eurasian species of *V*. sect. *Valerianella* are characterized by their laterally compressed achenes and their non-persistent calyx in the fruit or their persistent calyx formed by three small teeth, one of them horn-shaped. Both sections share achenes with well-developed spongy tissue in the fertile and sterile cavities of the fruit, although, in *V*. sect. *Valerianella*, these are not always present (Figure 2, Table 1). The Mediterranean species of *V*. sect. *Coronatae* and the Old and New World species of *V*. sect. *Platycoelae* share a persistent crown-toothed fruit calyx; however, while the crown-toothed fruit has hooked teeth or is replaced by a trilobed disc, or a narrow (toothed) ring in the former group, it consists of unhooked teeth or is replaced by a toothed or denticulate tongue or by a truncated cylinder with a tooth in the second group (Figure 2, Table 1) [6]. Population-level dbRDA tests detected a significant correlation of genetic distances with morphological traits (Table 3); therefore, long-term genetic isolation significantly explains the carpological differentiation of the main sections of *Valerianella*. Dating analysis conducted on the plastid phylogeny estimates that the early *Valerianella* + *Fedia* splits (15.9 Ma, node b; 14.6 Ma, Figure 3) coincided with a global warming event [45]. At that time, a period of increasing seasonality could have occurred in Central Europe and the Mediterranean area, where two major seasonal phases of hot and dry and cold and wet climate date from around 16.3–15.7 and 14.7–14.5 Ma, respectively [45]. Our dating analysis also indicates that the four main *Valerianella* sectional lineages diverged in the late Miocene (9.9–7. 6 Ma; Figure 3). These ages are coincident with a warmer and wetter period characteristic of the Upper Tortonian [46]. These changing climate conditions could have caused the origin of diverse environments, promoting the successful diversification of several herbaceous lineages such as the *Valerianella* sectional ancestors and those of other Mediterranean angiosperms [47]. Evolutionary (Figure 2 and Figure 3) and statistical (Table 3) analyses support the phylogenetic value of the diagnostic carpological traits separating the four monophyletic sections of *Valerianella*. 

### 3.2. A New Taxonomic Section of Valerianella to Accommodate V. fusiformis

One of the *Valerianella* species studied, *V. fusiformis*, did not fit into its putative *V.* sect. *Platycoelae* group [6,10] or in any other major sectional group of *Valerianella* in our plastid topologies (Figure 2, Figure 3, and Figure S1) and haplotypic network (Figure 4). This species, endemic to Northern and Eastern Spain, which grows in therophytic pastures on stony substrates, preferably limestones, was classified within *V*. sect. *Platycoelae* based on its monomorphic fruits with developed sterile cavity and sterile cavities reduced to arched ribs [6]. However, our plastid phylogenies placed it either as an early split within the *Valerianella* + *Fedia* clade based on *trn*L-F data (Figure 2 and Figure 3) or as a separate, early diverging lineage within the *V*. sect. *Coronatae* + *Platycoelae* clade based on combined *trn*T-L + *trn*L-F data (Figure S1). The haplotypic network based on the later data set also showed the isolation of the *V. fusiformis* haplotype XVIII, which was separated by 69 steps from its closest *V*. sect. *Coronatae* haplotypes (Figure 4). Despite the different topological resolutions for *V. fusiformis*, which are caused by homoplasies between the plastid *trn*T-L and *trn*L-F characters, both trees support the placement of *V. fusiformis* outside of the *V*. sect. *Platycoelae* clade, a lineage that includes correctly classified species from the Old and the New Worlds (Figure 2, Figure 3, and Figure S1). Careful inspection of the carpological traits of *V. fusiformis* allows us to separate this species from the other *Valerianella* species based on the unique features of its fruit (Figure 2, Table 1). Due to the taxonomic singularity of the *V. fusiformis* carpological traits and its large phylogenetic divergence from the remaining *Valerianella* sections (Figure 2, Figure 3, and Figure S1), we propose its classification into a newly described *Valerianella* section *Stipitae* (see below). 

### 3.3. Valerianella Species Pairs Are Constituted by a Single Species with a Genetic Regulatory Mechanism for Different Carpological Traits

In contrast to the strongly supported phylogenetic divergences of *Valerianella* sectional lineages (Figure 1, Figure 2, and Figure S1) and their clear taxonomic differentiation based on several statistically significantly different carpological traits (Figure 2, Table 3), our evolutionary and population-level analyses do not separate the species of each of the studied species pairs of *V*. sects. *Valerianella*, *Coronatae*, and *Platycoelae* as reciprocally monophyletic lineages or genetically diversified groups (Figure 2, Figure 3, Figure 4, Figure 5 and Figure S1), despite their distinctive fruiting characteristics (Figure 2; [6,10]). Both the plastid phylogenetic trees plus the haplotypic network and the nuclear AFLP clusters show the mixture of individuals of both species in each of the studied *V. carinata–V. locusta* (*+ V. lusitanica*), *V. coronata–V. pumila*, and *V. dentata–V. rimosa* species pairs (Figure 2, Figure 3, Figure 4, Figure 5A–D and Figures S1–S3). Plastid reconstructions also recovered a mix of individuals from both species within the *V. discoidea–V. multidentata* and *V. eriocarpa–V. microcarpa* species pairs (Figure 2, Figure 3, Figure 4 and Figure S1). Furthermore, in the three more extensively studied species pairs (*V. carinata–V. locusta* (*+V. lusitanica*), *V. coronata–V. pumila*, *V. dentata–V. rimosa)*, our evolutionary and genetic analyses have detected a geographic structure between the populations of each pair, instead of a taxonomic structure (Figure 2, Figure 3, Figure 4, Figure 5, Figure S1, Figure S2A,B and Figure S3A–G). It represents a typical scenario for a single species where isolation-by-distance is the main factor responsible for the divergence of populations, as in many other Mediterranean or North American annual plants (e.g., *Brachypodium distachyon*, [48]; *Arabidopsis thaliana*, [49]; *Mimulus guttatus*, [50]). Within the *V. carinata–V. locusta* (*+V. lusitanica*) species pair, three to five genetic groups have been identified in the STRUCTURE plot and the neighbor network tree, respectively (Figure 5A–C and Figure S3A,E), corresponding to SW Iberian populations of VCA + VLU, Central–S Iberian populations of VCA + VLO, Central Iberian populations of VCA + VLO, and Central Iberian populations of VLO, with individuals from two of the last groups showing admixed genetic profiles between those of the three main STRUCTURE groups. This nuclear genetic profiling pattern fits the plastid haplotypic pattern where individuals of each of the five *V. carinata–V. locusta* (*+V. lusitanica*) geographic groups have private haplotypes separated from each other by several steps (Figure 4), except for the Central Iberian VCA and VCO groups that share the most common haplotype I (Figure 4). Within the *V. coronata–V. pumila* species pair, two genetic groups have been differentiated in the STRUCTURE and neighbor network analyses (Figure 5A–D and Figure S3C–E), corresponding to Central and NE Iberian populations of VCO+VPU and NE Iberian populations of VPU, which are also partially reflected in their common and private plastid haplotypes (Figure 4). Within the *V. dentata–V. rimosa* species pair, two other genetic groups have been differentiated; one of them is formed by two sympatric populations of VDE+VRI from easternmost NE Spain and the other by VDE populations from N and NE Spain (Figure 5B,C and Figure S3D–G), although all the individuals share the same plastid haplotype (Figure 4). The genetic admixture of individuals from different species is only restricted to each of the five *Valerianella* species pairs studied (Figure 2, Figure 3, Figure 4, Figure 5, Figure S1 and Figure S2) and not between other *Valerianella* species. It is demonstrated by the fact that sympatric populations of *Valerianella* species, even from the same section but from different species pairs (e.g., *V. pumila* and *V. multidentata*, NE Spain; *V. coronata* and *V. discoidea*, C Spain; Figure 1), do not show mixing between their individuals (Figure 2, Figure 3, Figure 4, Figure 5, Figure S1 and Figure S2).

Although our conservative plastid-based age estimates indicate that these *Valerianella* species pairs could have originated as early as the Late Miocene (e.g., *V. carinata–V. locusta* (*+V. lusitanica*), 7.6 Ma; *V. coronata–V. pumila*, 6.3 Ma), in the Pliocene (e.g., *V. discoidea–V. multidentata* 4.8 Ma; *V. eriocarpa–V. microcarpa*, 4.3 Ma), or more recently in the Pleistocene (e.g., *V. dentata–V. rimosa* 1.8 Ma) (Figure 3), their respective genetic admixtures are not a consequence of the lack of evolutionary time for speciation, since it is accepted that the radiations of most Mediterranean plants occurred during these late Neogene–Pleistocene time periods [51,52]. Their genetic admixture cannot be attributed to recent hybridizations because these *Valerianella* species show a predominant selfing breeding system [8] and homoploid hybrid plant species are known to have speciated until recently [48]. Furthermore, we hypothesize that the high selfing rates shared by the annual *Valerianella* species might have been acquired from their common Late Miocene ancestor (Figure 3). The aridification of the circum-Mediterranean region at the end of the Miocene probably favored the development of annual and biennial self-fertilizing species [53] since, in limited environments with low abundance of pollinators or changes between seasons to provide reproductive assurance, selfing is reproductively more efficient than outbreeding [54]. Similar evolutionary patterns have been suggested for other lineages of annual self-pollinating plants that constitute the majority of the Mediterranean flora [51,53,55]. However, the relatively high to moderate values of genetic diversity detected in some populations of the studied *Valerianella* species pairs are surprising (P_99_, *PLP*, *hsp*, *h*; Table 2, Table S3). These values are higher than those detected in populations of other highly self-fertilizing annual plants (e.g., *B. distachyon*, [48]). This could be a consequence of the large population sizes and rapid mutation rate of some of the annual *Valerianella* species and occasional interbreeding between individuals, as indicated for the *V. coronata–V. pumila* group, where outcrossing can occur randomly in nature [8]. In contrast, genetic structure is high among populations (70.13%), as previously described for *Valerianella* [34] and expected for self-pollinated plants [56,57,58], where selfing increases the genetic structure between populations due to the absence of gene flow [59]. The probable existence of a mixed mating system (selfing, crossbreeding) in the *Valerianella* species pairs [8] would explain the balance between the moderate values of genetic diversity and the high genetic structuring of their populations, since complete selfing is extremely rare in nature [60].

The morphological differentiation of the separately recognized species within each of the *Valerianella* species pairs studied is based on only one (presence vs. absence of inflated sterile cavities of the achene) or up to two carpological traits (presence vs. absence of remaining calyx or presence of two calyx shapes in the fruit), while they are similar in the rest of the vegetative and reproductive features [6,10]. Alternative fruit morphotypes of *Valerianella* and *Fedia* species pairs have been shown to be caused by a genetic regulatory mechanism governed by two linked loci [8,26]. Self-pollinating experiments of the two parental *V. coronata* and *V. pumila* individuals and of their cross-bred F1 progeny showed that the fruit shapes of *V. coronata* and *V. pumila* were determined by two codominant alleles at a single locus, and that the two morphs resulted from the expression of different segregating genotypes [8]. Since eventual crosses between both morphotypes can occur spontaneously, they produced an intermediate heterozygous morphotype that other authors misinterpreted as different species (e.g., *V. cupulifera* Legrand, [8]). However, these heterozygous morphotypes are rare and rapidly disappearing from nature as a consequence of the predominant selfing reproductive system of the parental morphotypes. Similarly, self-pollination experiments in di-morphic and tri-morphic populations of *Fedia pallescens* demonstrated that two diallelic loci are linked on the same chromosome in a functional supergene, and that one allele from each locus shows a dominant effect at the heterozygous stage, causing the co-segregation of the two morphs in the populations [26]. Both experiments suggest that the fruit morphotype regulatory system occurs within a single species and that it develops into distinct intraspecific carpological morphs when two segregating genotypes coexist in the same heteromorphic population or when each genotype is present in separate monomorphic populations. Our evolutionary and population genetics analyses of *Valerianella* have demonstrated that this genetic regulatory system of fruit morphotype occurs in all five species pairs studied from three sections of *Valerianella*, *V*. sect. *Valerianella* (*V. carinata–V. locusta* (*+ V. lusitanica*)), *V*. sect. *Coronatae* (*V.* *coronata–V. pumila*, *V. discoidea–V. multidentata*), and *V*. sect. *Platycoelae* (*V. dentata–V. rimosa, V. eriocarpa–V. microcarpa*) (Figure 1, Figure 2, Figure 3 and Figure 5), suggesting that the two linked loci responsible for these carpological traits were likely present in the ancestor of the *Valerianella* + *Fedia* clade. Interestingly, in the studied representative of *V*. sect. *Cornigerae*, *V. echinata*, the two fruit-bearing forms exist in the same heterocarpic plant; the flowers of the axillary branches have achenes without inflated sterile cavities and a long vestigial calyx, while the flowers of the terminal branches have achenes with inflated sterile cavities and a short remaining calyx (Figure 2; [6]), which suggests that the regulatory mechanism is expressed differently in different parts of the inflorescence. More comprehensive gene expression analyses would be needed to investigate the allelic composition of the two linked loci in the *Valerianella* species pairs studied to confirm their role in determining fruit morphotypes and their inheritance. Taxonomically, the five *Valerianella* species pairs studied must be considered single species. Devesa and López [6,10] treated three of them as a single species and their different fruit morphs as forms of the same species (i.e., *V. locusta* subsp. *locusta* f. *locusta* and f. *carinata*, and *V. lusitanica* as subspecies of *V. locusta*, *V. locusta* subsp. *lusitanica*, although it should probably be considered as a third form, f. *lusitanica*, due to its evolutionary closeness to some populations of *F*. *carinata* (Figure 2, Figure 3, Figure 4, Figure 5 and Figure S2); *V. coronata* f. *coronata* and f. *pumila*; *V. dentata* f. *dentata* and *f. rimosa*). Our study supports an intraspecific taxonomic treatment for the fruit morphotypes of these species pairs of *Valerianella* and also for those of *V. discoidea–V. multidentata* and *V. eriocarpa–V. microcarpa*, either as forms or as any other infraspecific rank, for a natural classification of cornsalads. Future reproductive and genomic studies of these *Valerianella* species will contribute to a better understanding of the breeding system and the evolution of this economically and ecologically important genus.

### 3.4. Description of Valerianella sect. Stipitae, sect. nova 

*Valerianella* sect. *Stipitae* López & Devesa, *sect. nov.*

*Description*: Homocarpic plants presenting monomorphic, stipitated, and fusiform achenes showing a highly developed convex fertile cavity not filled with spongy tissue and two sterile cavities reduced to arched ribs filled almost completely with spongy tissue, and absence of calyx in the fruit.

*Typus*: *Valerianella fusiformis* Pau in Bol. Soc. Esp. Hist. Nat., 21: 144 (1921) [*Ind. loc.:* “Nieva de Cameros (Logroño), 5 julio 1905”; *lectotypus*: Caroli Pau Herbarium Hispanicum / Nieva de Cameros (Logroño) / 5 Julii 1905 (MA 119385), designated here by J. López & J.A. Devesa).

The section is integrated only by *Valerianella fusiformis* Pau. It differs from the rest of the sections by the fusiform and stipitate achenes of the plants (Figure 2; [6]).

## 4. Materials and Methods

### 4.1. Population Sampling and DNA Isolation

A total of 414 individual samples were collected from 54 populations of 16 different species of *Valerianella* (13; Figure 1), *Fedia* (2), and the outgroup *Centranthus* (1) throughout the Iberian Peninsula. The number of samples collected per population ranged from 5 (VPU03, VDI03) to 30 (VDE02) depending on the size of the population and the availability of individuals. Sampling was more exhaustive on populations of the three *V. coronata*–*V. pumila*, *V. carinata*–*V. locusta*, and *V. dentata* –*V. rimosa* species pairs, including both sympatric and allopatric populations of one and the other fruit morphotype in each case (Table S1). Total genomic DNA was extracted using the DNAeasy kit (Quiagen). The extracted genomic DNA was quantified in 1% agarose gels by comparing the intensity of the band with a control DNA sample of 100 ng /µL (Invitrogen).

### 4.2. Plastid DNA Sequence Analysis

The *trn*T-L and *trn*L-F plastid regions were amplified and sequenced in a total of 37 (*trn*L-F-*trn*T-L) and 43 (*trn*L-F) individuals from 13 representative *Valerianella* species plus 2 from *Fedia cornucopiae* (Table S1) and 2 from *Centranthus calcitrapae* used to root the ML phylogenetic tree. PCR amplification and sequencing were carried out with the external forward/reverse primer–pair combinations “a”/“b” and “c”/“f” for, respectively, the *trn*T-L and *trn*L-F regions [61], following the protocols indicated in [62]. Corrected consensus sequences were aligned with the Clustal X algorithm using Se-Al v.2.0 [63] and manually adjusted. The indels were encoded as binary characters; only those gaps that were unambiguous and potentially informative [64] were added to the corresponding data matrices and used in the statistical parsimony network analysis and maximum likelihood *trn*T-L + *trn*L-F analysis.

Forty samples were selected for phylogenetic, dating, and haplotype network analysis (Table S2). Phylogenetic maximum likelihood analysis of the combined *trn*T-L and *trn*L-F data sets was conducted in IQTREE [65]. We imposed the best-fit nucleotide substitution model (K3Pu+F+I) that was automatically selected by the ModelFinder option of the program [66] according to the Bayesian Information Criterion (BIC). Each search was performed through the automated computation of 20 maximum likelihood (ML) starting trees from 98 alternative randomized maximum parsimony (MP) trees, searching for the best-scoring ML trees and estimating branch support for the best tree from 1000 bootstrap replicates (BS) using the UltraFast Bootstrap option implemented in the software [67,68]. 

We used the most widely sampled *trn*L-F data set to compute a large Valerianoideae ML tree and to estimate the divergence times of the studied *Valerianella* and allied lineages using BEAST v1.8.0 [69]. For this, we downloaded from GenBank additional sequences of 8 American *Valerianella* species [36] and 5 species of *Valeriana*, 1 of *Plectritis*, 3 of *Centranthus*, 1 of *Nardostachys,* 3 of *Patrinia*, and 1 of *Triplostegia* that were used to root the tree (Table S1; [36,70]). Due to the lack of *Valerianella* fossils, we used secondary calibrations according to the family-wide phylogeny of [36]. The estimated ages for the most recent common ancestors of Caprifoliaceae subfam. Dipsacoideae and subfam. Valerianoideae were set at 62.48 ± 3.6 Ma and 51.58 ± 2.9 Ma, respectively, and those of the core Valerianoideae (*Plectritis*, *Centranthus*, *Valeriana*, *Valerianella*, and *Fedia*) and *Valerianella* + *Fedia* were set at 20.34 ± 3.7 Ma and 10.71 ± 2.8 Ma, respectively (Figure 3). The BEAUti interface was used to create the BEAST input file using the following parameters: (i) General Time Reversible model with a gamma distribution with four categories of rates and proportion of invariant sites (GTR+I+G), (ii), a relaxed molecular clock model with uncorrelated rates drawn from a log-normal distribution, (iii), a Yule model of evolutionary process with random start to infer the tree topologies, and (iv) an angiosperm molecular evolutionary mutation rate (UCDL) of 1e-4 to 1e-1 typical of most angiosperms. We used BEAST to infer the topology, branch lengths, and tree node dates. BEAST MCMC chain length analysis was run for 200 million generations, saving data every 1500 generations. We used Tracer v1.6. [71] to evaluate parameter convergence statistics for Effective Sample Size (ESS) values >200. The maximum clade credibility tree with mean ages and 95% highest posterior probability density (HPD) of nodes was summarized with TreeAnnotator and visualized with FigTree v.1.3.1 [72].

Statistical parsimony haplotype networks of *Valerianella* species were constructed using the combined *trn*T-L + *trn*L-F plastid data set with TCS v1.2.1 [73], considering gaps as a 5th state, and establishing a maximum connection of 72 steps. 

### 4.3. AFLP Analysis

In total, 414 individuals from 10 species of *Valerianella* were used in the AFLP-based analysis (Table S1). The AFLP procedure followed the protocol of [74]. Briefly, 200 nanograms of the genomic DNA was digested by two restriction enzymes (EcoRI/MseI) and ligated to double-stranded EcoRI and MseI adaptors in successive steps. These fragments were pre-amplified using EcoRI and MseI primers with one selective nucleotide, and then amplified using more specific primers with three selective nucleotides. Products were separated on 6% denatured polyacrylamide gels. Electrophoresis was conducted for two hours at 80 watts. Bands were visualized by silver staining following Bassam et al. (1991). Ten combinations of ECO/MSE primer–pairs were tested in a preliminary assay to select the most informative and reliable primer combinations. Repeatability analysis was performed for each primer–pair combination on a reduced subset of the samples (10) to test consistency in the multilocus profiles obtained. 

AFLP bands were scored as present (1) or absent (0). Genetic diversity estimators such as number of rare fragments (*f_r_*), unique (exclusive) fragments (*f_u_*), and diagnostic (*f_d_*) fragments were calculated at species pair, species, and population levels. The fragments were treated as “rare” and “diagnostic” when the frequencies were less than 0.02 or greater than 0.99, respectively [75]. The percentage of polymorphic AFLP fragments was calculated using the TFPGA v.1.3 software [76] at the 1% level (P_99_). The intrapopulation genetic diversity of each taxon was measured from all loci in each data set as band richness after rarefaction (*Br*), and the percentage of polymorphic loci at 1% level for a standardized sample size (*PLP*) calculated using Aflpdiv 1.1 [77]. Bayesian genetic diversity was calculated as the average panmictic heterozygosity within each population (*hs*) and each species (*hsp*), and its average value was estimated between the studied species (*Hsp*) using HICKORY v.1.0.4 [78]. Nei’s (1973) genetic diversity index was calculated for each species and at the population level.

Genetic relationship between individuals was revealed through (i) neighbor joining network analysis conducted on SplitsTree5 [79], and (ii) three-dimensional principal coordinate analysis (PCoA) with a Minimum Spanning Tree (MST, [80,81]) superimposed on the PCoA plots using NTSYSpc v. 2.11a [82]. Neighbor joining network analyses were also performed separately for each *Valerianella* species pair. Neighbor joining clustering based on *F_ST_* statistics obtained from the analysis of molecular variance (AMOVA, [83]) was conducted in MEGA v.5 [84] at the population level.

A Bayesian genetic structure analysis was performed to infer the structure of *Valerianella* samples and their sections, species pairs, and populations using STRUCTURE v.2.2 [85]. We imposed the admixture ancestry model with correlated allele frequencies. We ran the analysis for a range of K values starting from 1 to 50, using a burn-in period and a run length of the Monte Carlo Markov Chain of 75,000 and 150,000 iterations, respectively. Chain convergence was estimated through visual inspection of the posterior values excluding the burn-in. Ten iterations were conducted using the ad hoc parameter ΔK of [86] to estimate the rate of change of likelihood values between successive K values, using STRUCTURE HARVESTER [87]. Genetic substructuring within *Valerianella* sections was further assessed through independent analyses of the split data matrices using the same procedures indicated above (for K groups ranging from 1 to the number of analyzed populations plus two). 

A distance-based redundancy analysis (dbRDA; [88]) was conducted to examine whether genetic distances could explain the observed morphological differentiation in diagnostic carpological traits of *Valerianella* sections. The dbRDA analysis is a multivariate method that allows testing of the influence of genetic factors on the values of a linearly dependent dissimilarity matrix (in this case, the morphological distance). To perform the dbRDA analysis, we encoded as binary traits the most important morphological qualitative diagnostic characters for the 13 *Valerianella* species studied that belong to each of the four main sections plus the newly described *V*. sect. *Stipitae* (Table 1). To calculate the phenotypic distance between populations, we used the PCoA function of the ape package [89], which computes the PCo scores of the main axes obtained from the PCoA. The first three PCo component scores of PCoA were used for dbRDA analysis. We calculated pairwise population *F_ST_* distances as genetic data and converted them to a square data set using the prcomp function of the stats package. dbRDA analysis (marginal test) was performed with the R package VEGAN [90] using the capscale function. The statistical significance of the predictors, assigned using multivariate F statistics with 9999 permutations and variance components, was obtained with the anova.cca and RsquareAdj functions. All statistical analyses were performed in R version 3.4.3 [91].

## Figures and Tables

**Table 1:** A summary of the carpological diagnostic features characterizing the four main sections of *Valerianella* (*V*. sects. *Cornigerae*, *Coronatae*, *Platycoelae*, and *Valerianella*) and the newly described *V*. sect. *Stipitae* analyzed in this study.

*Valerianella* Sections and Species	Fruit Diversity	Calix Shape	Achene Type and Shape	Achene Compression	Spongeous Tissue in Fertile and Sterile Cavities of Achene
***Coronatae*** *V. coronata* *V. discoidea* *V. multidentata* *V. pumila*	Homocarpous plant	Persistent in the fruit, forming a toothed crown with hooked teeth, a trilobulated disk, or a narrow-toothed ring	Monomorphic or dimorphic, not fusiform, not stipitate	Not (strongly) laterally compressed	Not developed
***Cornigerae*** *V. echinata*	Heterocarpous plant	Persistent in the fruit, formed by 3 teeth, all of them horn-shaped	Only dimorphic, not fusiform, not stipitate	Not (strongly) laterally compressed	Well developed
***Platycoelae*** *V. eriocarpa* *V. dentata* *V. microcarpa* *V. rimosa*	Homocarpous plant	Persistent in the fruit, forming a toothed crown with unhooked teeth, an entire tongue, toothed or denticulate, or a truncated cylinder with a tooth	Monomorphic or dimorphic, not fusiform, not stipitate	Not (strongly) laterally compressed	Not developed
***Stipitae*** *V. fusiformis*	Homocarpous plant	Not persistent in the fruit	Only monomorphic, fusiform, stipitate	Not (strongly) laterally compressed	Not developed
***Valerianella*** *V. carinata* *V. locusta* *V. lusitanica*	Homocarpous plant	Not persistent in the fruit, or formed by 3 small teeth, one of them horn-shaped	Monomorphic or dimorphic, not fusiform, not stipitate	(Strongly) laterally compressed	Not developed / well developed

**Table 2:** Genetic diversity values of the 10 Iberian species of *Valerianella* studied. Number of populations studied (*N*), number of rare fragments (*fr*), number of unique fragments (*fu*), number of diagnostic fragments (*fd*), percentage of polymorphic loci at the 99% criterion (*P99*), percentage of polymorphic loci at the 1% criterion after rarefaction (*PLP*), band richness after rarefaction (*Br*), Bayesian diversity within species (*hsp*) and between species (*Hsp*), Nei´s genetic diversity (*h*).

Taxon	*N*	*f_r_*	*fu*	*fd*	P_99_	*PLP(6)*	*Br(6)*	*h_Sp_*	*h*
**Sect. *Valerianella***									
*V. locusta*	6	20	3	0	50.8	53.6	1.261	0.113	0.720
V. carinata	11	25	7	0	47.8	55.8	1.267	0.120	0.752
*V. lusitanica*	1	0	2	0	16.1	12.9	1.137	0.082	0.323
Sect. *Valerianella*	18	45	12	0	32.8	40.7	1.221	*Hsp* = 0.150	0.789
**Sect*. Coronatae***									
*V. coronata*	9	12	2	0	37.8	38.8	1.188	0.083	0.707
*V. pumila*	4	0	2	0	33.0	33.9	1.186	0.087	0.695
*V. discoidea*	8	0	6	0	42.6	43.8	1.262	0.116	0.805
Sect. *Coronatae*	21	12	10	0	37.8	38.8	1.212	*Hsp* = 0.128	0.844
**Sect*. Platycoelae***									
*V. dentata*	5	0	3	0	46.5	47.8	1.298	0.132	0.797
*V. rimosa*	1	0	0	0	14.3	11.6	1.112	0.067	0.415
*V. eriocarpa*	4	0	1	0	14.8	14.3	1.119	0.067	0.551
Sect. *Platycoelae*	10	0	4	0	25.2	24.5	1.176	*Hsp* = 0.139	0.835
**Sect*. Cornigerae***									
*V. echinata*	1	0	0	8	6.2	-	1.009	0.058	0.048
Sect. *Cornigerae*	1	0	0	8	6.2	-	1.009	*Hsp* = 0.348	0.048
Total	50	57	26	8	30.5	-	-	*Hsp* = 0.207	0.892

**Table 3:** Results of the distance-based redundancy analysis (dbRDA) of the four *Valerianella* sections obtained from the marginal test that tested the correlation between the genetic distances of the populations (*F_ST_*) (explanatory variable) and the morphological differentiation of the sections based on their diagnostic carpological traits (response variable, see Table 1). Significance of predictors test: *p* < 0.001 ***.

dbRDA			
Marginal Test			
Variable	F	*p*	% Var
*F_ST_* (PC1)	65.34	0.001 ***	57.28
*F_ST_* (PC2)	1.95	0.258	1.71
*F_ST_* (PC3)	0.76	0.521	0.67

## Data Availability

https://github.com/Bioflora/Valerianella/.

## Supplementary Materials

The following supporting information can be downloaded at: https://www.mdpi.com/article/10.3390/plants11101276/s1, Figure S1: Maximum likelihood plastid *trn*L-F tree constructed with IQTREE showing the relationships between the *Valerianella* and *Fedia* species studied. Dipsacaceae sequences were used to root the tree. FCV (*Fedia* + *V*. sect. *Valerianella* clade), PC (*V*. sects. *Platycoelae* + *Coronatae* clade). Numbers on branches indicate UltraFast Bootstrap support (BS) values, Figure S2: Maximum likelihood plastid *trn*T-L+*trn*L-F tree constructed with IQTREE showing the relationships between the *Valerianella* and *Fedia* species studied. *Centhranthus calcitrapae* was used to root the tree. FCV (*Fedia* + *V*. sect. *Valerianella* clade), PC (*V*. sects. *Platycoelae* + *Coronatae* clade). Numbers on branches indicate UltraFast Bootstrap support (BS) values. Vertical bars on branches indicate informative *trn*T-L+*trn*L-F gap positions for the respective lineages (see Table S2), Figure S3: Bayesian genetic structure (STRUCTURE) and relationships (neighbor joining network topology) among individuals of the *Valerianella* species studied based on nuclear AFLP data for three *Valerianella* sections. (A) *V*. sect. *Valerianella*: *V. carinata* (VCA), *V. locusta* (VLO), *V. lusitanica* (VLU). (B) *V*. sect. *Coronatae*: *V. coronata* (VCO), *V. pumila* (VPU), *V. discoidea* (VDI). C) *V*. sect. *Platycoelae*: *V. dentata* (VDE), *V. rimosa* (VRI), *V. eriocarpa* (VER). Color codes of *Valerianella* species correspond to those indicated in Figure 1, Table S1: List of the *Valerianella*, *Fedia*, and *Centranthus* taxa sampled in the Iberian Peninsula and the outgroups used in the plastid and nuclear AFLP studies. For each entry, the population codes used in AFLP analysis and the localities of origin are indicated. GenBank accession codes in bold correspond to new data generated in this study. Taxonomic classification of *Valerianella* follows [6,91]. Herbaria acronyms: COFC (Córdoba University, Spain), K (Kew Royal Botanic Garden), UNEX (University of Extremadura, Spain), YU (University of Alabama, US), Table S2: Summary of the nucleotide site variation found across the plastid *trn*T-L + *trn*L-F aligned data matrices of the *Valerianella*, *Fedia*, and *Centranthus* taxa studied, Table S3: Genetic diversity indices calculated from AFLP data of 50 populations of the *Valerianella* species under study. Taxon, population code, number of studied individuals (*N*), number of unique fragments (*fu*), number of diagnostic fragments (*fd*), percentage of polymorphic loci at the 99% criterion (*P99*), Bayesian diversity within population (*hs*), Nei´s genetic diversity (*h*).
