# Supplementary material for: Molecular Systematics of Valerianella Mill. (Caprifoliaceae): Challenging the Taxonomic Value of Genetically Controlled Carpological Traits
Source: Plants (Basel). 2022 May 10;11(10):1276. doi: 10.3390/plants11101276 (PMC9146508; doi:10.3390/plants11101276)
Supplement: Supplementary file 1 [file plants-11-01276-s001.zip › plants-1677153-supplementary/table/SupplementaryTables1.pdf]

Supplementary Tables

Table S1. List of the *Valerianella*, *Fedia* and *Centranthus* taxa sampled in the Iberian Peninsula and the outgroups used in the plastid and nuclear AFLP studies. For each entry, the population codes used in AFLP analysis and the localities of origin are indicated. GenBank accession codes in bold correspond to new data generated in this study. Taxonomic classification of *Valerianella* follows Euro+Med (2006) and Devesa & López (2007). Herbaria acronyms: COFC (Córdoba University, Spain), K (Kew Royal Botanic Garden), UNEX (University of Extremadura, Spain), YU (University of Alabama, US).

**Species**	**Locality and collections details**	**AFLP Code**	**GenBank accession *trn*TL**	**GenBank accession *trn*LF**
Centranthus calcitrapae (L.) Dufr.	SPAIN, Cuenca, Montalbanejo, J. López & F. Bueno 5/06, UNEX 35366.		XXXXX	XXXXX
	SPAIN, Salamanca Ituero de Azaba, J. López & E. López 39/05, UNEX 35343.		XXXXX	XXXXX
Centranthus lecoqii Jord.	Amich et al. 354, YU.			DQ354198
Centranthus sieberi Heldr.	Maich 6083, YU.			DQ354195
Fedia cornucopiae (L.) Gaertn. var. *cornucopiae*.	SPAIN, Córdoba. Sierra Horconera.		XXXXX	XXXXX
Fedia cornucopiae var. *scorpioides* (Dufr.) J. López & Devesa	SPAIN, Badajoz, ctra. de Badajoz a Olivenza, J. López & F.J. Valtueña 3/05, UNEX 35316.		XXXXX	XXXXX
Nardostachys jatamansi (D. Don) DC.	CHINA, Sichuan province, Boufford et al. 28099, A.			AF447010
Patrinia_gibbosa Maxim.	SWITZERLAND, Cult. Zurich Bot. Gard., Switzerland. (A. Mast), 19885022.			AY792886
Patrinia villosa (Thunb.) Juss.	SWITZERLAND, Cult. Zurich Bot. Gard., Switzerland. (A. Mast), 19910230.			AY792888
Patrinia saniculifolia Hemsl.	SOUTH KOREA, Kang-won Prov, Chase 19250, K.			JF269261
Plectritis congesta (Lindl.) DC.	USA, Oregon, Benton Co. Shenk 308, YU.			AY792889
Triplostegia glandulifera Wall. ex DC.	CHINA, Sichuan Province, Boufford et al. 28440, A.			MF737297
Valeriana albonervata B.L.Rob.	MEXICO, Tamaulipas, Barrie & Nixon 1279 (YU).			AY792893
Valeriana celtica L.	SWITZERLAND, Valais. Bell, #SWITZ002 (YU).			AY360121

Species	Locality and collections details	AFLP Code	GenBank accession *trn*TL	GenBank accession *trn*LF
Valeriana dioica L.	SWITZERLAND, Cult. Zurich Bot. Gard., #16910745 (YU).			AY360119
Valeriana edulis Nutt. ex Torr. & A. Gray	USA, Colorado, Park Co., Barrie #822 (YU).			AY360127
Valeriana nivalis Wedd.	Bolivia, La Paz. Eriksen & Molau, 4830 (YU)			AY792914
Valerianella amarella (Lindh. ex Engelm.) Krok	USA, Texas, Brown, Both sides of main gravel road, 0.3 mi S of gate E of barracks area, Camp Bowie Training Site (TX National Guard), Carr 12722, TEX 00020356.			DQ354210
Valerianella coronata (L.) DC. f. *coronata*	SPAIN, Ciudad Real, Viso del Marqués, J. López 25/05, UNEX 35331.	VCO01		XXXXX
	SPAIN, Cuenca, Montalbanejo, J. López 26/05, UNEX 35332.	VCO02		XXXXX
	SPAIN, Cuenca, Pinarejo, J. López & F. Bueno 6/06, UNEX 35295.	VCO03		
	SPAIN, Madrid Arganda del Rey, J. López & F. Bueno 18/05, UNEX 35330.	VCO04		XXXXX
	SPAIN, Salamanca, El Bodón, J. López & E. López 38/05, UNEX 35342.	VCO05		XXXXX
	SPAIN, Segovia, Torrecilla del Pinar, J. López 34/05, UNEX 35337.	VCO06		
	SPAIN, Huesca, Bentué, J. López 46/05, UNEX 35347.	VCO07		XXXXX
	SPAIN, Cuenca, Montalbanejo, J. López & F. Bueno 4/06, UNEX 35363.	VCO08		
	SPAIN, Huesca, entre San Esteban de Litera y Aznauy, J. López & F. Bueno 13P/07, UNEX 35359.	VCO09		
	USA, Cult. Yale Univ., Bell 105, YU.			AY792941

**Species**	**Locality and collections details**	**AFLP Code**	**GenBank accession *trn*TL**	**GenBank accession *trn*LF**
Valerianella coronata f. *pumila* (L.) Devesa, J. López & R. Gonzalo	SPAIN, Cuenca, Montalbanejo, J. López & F. Bueno 3/06, UNEX 35362.	VPU01		XXXXX
	SPAIN, Huesca, San Esteban de Litera y Aznauy, J. López & F. Bueno 14P/07, UNEX 35358.	VPU02		
	SPAIN, Zaragoza, Osera de Ebro, J. López & F. Bueno 10P/07, UNEX 35298.	VPU03		XXXXX
	SPAIN, Huesca, San Esteban de Litera, J. López & F. Bueno 12P/07, UNEX 35297.	VPU04		XXXXX
	USA, Cult. Yale Univ., Bell 108, YU.			AY792945
Valerianella dentata (L.) Pollich f. *dentata*	SPAIN, Burgos, Oña, J. López 60/05, UNEX 35369.	VDE01		
	SPAIN, Huesca, Yebra de Basa, J. López 44/05, UNEX 35759.	VDE02		
	SPAIN, Huesca, Jaca, San Juan de la Peña, J. López 43/05, UNEX 35346.	VDE03		XXXXX
	SPAIN, Huesca, San Juan de Plan, pista a San Mamés, J. López 20/07, UNEX 35655.	VDE04		
	SPAIN, Huesca, Puerto de Bonansa, J. López 22/07, UNEX 35653.	VDE05		
Valerianella dentata f. *rimosa* (Bastard) Devesa, J. López & R. Gonzalo	SPAIN, Huesca, San Juan de Plan. Pista a San Mamés, J. López 21/07, UNEX 35652.	VRI01		XXXXX
	SPAIN, Huesca Puerto de Bonansa, J. López 23/07, UNEX 35654.	VRI02		XXXXX
Valerianella discoidea (L.) Loisel	SPAIN, Badajoz, San Jorge de Alor, A. Ortega-Olivencia & J. López 4/05, UNEX 35327.	VDI01		
	SPAIN, Badajoz, San Isidro, A. Ortega-Olivencia & J. López 1/05, UNEX 35315.	VDI02		XXXXX
	SPAIN, Córdoba, entre Villafranca de Córdoba y Aldamuz, J. López & F. Bueno, 1/06, UNEX 35361.	VDI03		

**Species**	**Locality and collections details**	**AFLP Code**	**GenBank accession *trn*TL**	**GenBank accession *trn*LF**
	SPAIN, Cuenca, Pinarejo, J. López & F. Bueno 7/06, UNEX 35296.	VDI04		XXXXX
	SPAIN, Cuenca, proximidades a El Cubillo, J. López & F. Bueno 10/06, UNEX 35364.	VDI05		
	SPAIN, Huelva, Galaroza, A. Ortega-Olivencia & J. López 7/05, UNEX 35349.	VDI06		
	SPAIN, Madrid, Arganda del Rey, J. López & F. Bueno 16/05, UNEX 35352.	VDI07		
	SPAIN, Segovia, Coca, río Eresma, J. López 32/05, UNEX 35309.	VDI08		
Valerianella echinata (L.) DC.	SPAIN, Guadalajara, Muduex, vaguada, J. López & F. Bueno 8P/07, UNEX 35357.	VEC01		XXXXX
Valerianella eriocarpa Desv.	SPAIN, Badajoz, San Isidro, J. López & E. López 2/05, UNEX 35313.	VER01		
	SPAIN, Badajoz, La Haba, J. López & F. Bueno 11/05, UNEX 35350.	VER02		XXXXX
	SPAIN, Madrid, Arganda del Rey, J. López & F. Bueno 15/05, UNEX 35351.	VER03		
	SPAIN, Segovia, Coca. Río Eresma, J. López 33/05, UNEX 35310.	VER04		XXXXX
	USA, Cult. Yale Univ., Bell 107, YU.			AY792943
Valerianella florifera Shinners	USA, Texas, Fayette, Carr 12663, TEX 00350835.			DQ354211
Valerianella fusiformis Pau	SPAIN, Burgos, Oña, J. López 59/05, UNEX 35370.			XXXXX
Valerianella locusta (L.) Laterr. subsp. *locusta* f. *locusta*	SPAIN, Segovia, de Carbonero a Bernardo, J. López 28/05, UNEX 35333.	VLO01		XXXXX
	SPAIN, Huelva, Galaroza, A. Ortega-Olivencia & J. López 8/05, UNEX 35325.	VLO02		
	SPAIN, Madrid, Guadalix de la Sierra, J.	VLO03		XXXXX

**Species**	**Locality and collections details**	**AFLP Code**	**GenBank accession *trn*TL**	**GenBank accession *trn*LF**
	López & F. Bueno 12/05, UNEX 35328.			
	SPAIN, Madrid, cruce al Valle de los Caídos, J. López & F. Bueno 20/05, UNEX 35318.	VLO04		XXXXX
	SPAIN, Salamanca, Retortillo, J. López & E. López 37/05, UNEX 35340.	VLO05		XXXXX
	SPAIN, Segovia, Coca, río Eresma, izda. puente, J. López 30/05, UNEX 35320.	VLO06		XXXXX
	Amich et al. 324, YU.			DQ354201
Valerianella locusta subsp. *locusta* f. *carinata* (Loisel) Devesa, J. López & R. Gonzalo	SPAIN, Badajoz, Fregenal de la Sierra, A. Ortega-Olivencia & J. López 10/05, UNEX 35326.	VCA01		XXXXX
	SPAIN, Ciudad Real, Calzada de Calatrava, J. López 21/05, UNEX 35322.	VCA02		XXXXX
	SPAIN, Ciudad Real, El Viso del Marqués, J. López 24/05, UNEX 35324.	VCA03		XXXXX
	SPAIN, Huelva, río Odiel, A. Ortega-Olivencia & J. López 5/05, UNEX 35311.	VCA04		XXXXX
	SPAIN, Segovia, Coca, río Eresma, drcha. puente, J. López 29/05, UNEX 35334.	VCA05		XXXXX
	SPAIN, Madrid, Miraflores de la Sierra, J. López & F. Bueno 14/05, UNEX 35321.	VCA07		
	SPAIN, Vitoria, Ibarra, J. López 61/05, UNEX 35368.	VCA08		XXXXX
	SPAIN, Madrid, Guadalix de la Sierra, J. López & F. Bueno 13/05, UNEX 35656.	VCA09		XXXXX
	SPAIN, Segovia, Coca, río Eresma, izda. puente, J. López 31/05, UNEX 35336.	VCA10		XXXXX
	SPAIN, Salamanca, Retortillo, J. López & E. López 36/05, UNEX 35339.	VCA11		XXXXX

Species	Locality and collections details	AFLP Code	GenBank accession *trn*TL	GenBank accession *trn*LF
	SPAIN, Madrid, cruce al Valle de los Caídos, J. López & F. Bueno 19/05, UNEX 35319.	VCA12		XXXXX
Valerianella locusta subsp. *lusitanica* (Pau ex Font Quer) M. Laínz	SPAIN, Burgos, Pineda de la Sierra, J. López & F. Bueno 24/06, UNEX 35302.	VLU01		XXXXX
Valerianella microcarpa Loisel	SPAIN, Cáceres, Ctra. Deleitosa-Solana, T. Rodríguez-Riaño & J. López 2P/07, UNEX 35355.	VMI01		XXXXX
	Bell s.n., YU.			DQ354203
Valerianella multidentata Loscos & J. Pardo var. *oscensis* (Fanlo) Devesa, J. López & R. Gonzalo	SPAIN, Guadalajara, Muduex, vaguada, J. López & F. Bueno 6/07, UNEX 35306.	VMU02		
	SPAIN, Zaragoza, Osera de Ebro, J. López & F. Bueno 9P/07(2), UNEX 35649.	VMU03		
Valerianella radiata (L.) Dufr.	USA, Texas, Bosque, W side of lake, Carr 9672, TEX 00020418.			DQ354209
