# Supplementary material for: Molecular Systematics of Valerianella Mill. (Caprifoliaceae): Challenging the Taxonomic Value of Genetically Controlled Carpological Traits
Source: Plants (Basel). 2022 May 10;11(10):1276. doi: 10.3390/plants11101276 (PMC9146508; doi:10.3390/plants11101276)
Supplement: Supplementary file 1 [file plants-11-01276-s001.zip › plants-1677153-supplementary/table/SupplementaryTableS2.pdf]

Table S2. Summary of the nucleotide site variation found across the plastid *trn*LF-*trn*TL aligned data matrices of the *Valerianella*, *Fedia*, and *Centranthus* taxa studied.

Position

No.	Taxon	Sequence
		1 1 1 1 1 1 1 1 1 1 1 1 1 1 1 1 1 1 1 1 1 1 1 1 1 1 1
		6 6 6 6 6 6 7 7 7 7 7 7 7 7 7 7 8 8 8 8 8 8 8 8 8 8 9 9 9 9 9 9 9 9 9 9 0 0 0 0 0 0 0 0 0 0 1 1 1 1 1 1 1 1 1 1 2 2 2 2 2 2 2
		4 5 6 7 8 9 0 1 2 3 4 5 6 7 8 9 0 1 2 3 4 5 6 7 8 9 0 1 2 3 4 5 6 7 8 9 0 1 2 3 4 5 6 7 8 9 0 1 2 3 4 5 6 7 8 9 0 1 2 3 4 5 6
1	*Centranthus calcitrapae* CC25	T C T T C T C T A A G G A A A C C T A T A T T T G T A T T T - - - - - - G G T G C A A T C A A A A A C G A T T G T A T C A G T
2	*Centranthus calcitrapae* CCACC539	. . . . - - - - - - . . . .
3	*Fedia cornucopiae* FC73	. . . . C . . . . T . . . . - - - - - - - - - - - - . . . . T .
4	*Valerianella carinata* VCA0104	. . . . - - - - - - - - - . . . . G - - T T T . . . . T .
5	*Valerianella carinata* VCA0202	. . . . - - - - - - - - - . . . . G - - T T T . . . . T .
6	*Valerianella carinata* VCA0302	. . . . - - - - - - - - - . . . . G - - T T T . . . . T . . . . T .
7	*Valerianella carinata* VCA0501	. . . . - - - - - - - - - . . . . G - - T T T . . . . T .
8	*Valerianella carinata* VCA0805	. . . . - - - - - - - - - . . . . G - - T T T . . . . T .
9	*Valerianella carinata* VCA0918	. . . . - - - - - - - - - . . . . G - - T T T . . . . T .
10	*Valerianella carinata* VCA1006	. . . . - - - - - - - - - . . . . G - - T T T . . . . T .
11	*Valerianella carinata* VCA1106	. . . . - - - - - - - - - . . . . G - - T T T . . . . T .
12	*Valerianella carinata* VCA1202	. . . . - - - - - - - - - . . . . G - - T T T . . . . T .
13	*Valerianella coronata* VCO0102	. . . . T . . . . . - - - - - - . . . . T .
14	*Valerianella coronata* VCO0218	. . . . T . . . . . - - - - - - . . . . T .
15	*Valerianella coronata* VCO0404	. . . . T . . . . . - - - - - - . . . . T .
16	*Valerianella coronata* VCO0508	. . . . T . . . . . G T A T T T . . . . T .
17	*Valerianella coronata* VCO0705	. . . . T . . . . . - - - - - - . . . . T .
18	*Valerianella coronata* VCO0805	. . . . T . . . . . - - - - - - . . . . T .
19	*Valerianella coronata* VCO0901	. . . . T . . . . . - - - - - - . . . . T .
20	*Valerianella dentata* VDE0303	. . . . T . . . . - - - - - - . . . . T .
21	*Valerianella discoidea* VDI0202	. . . . T . . . . . - - - - - - . . . . T .
22	*Valerianella discoidea* VDI0412	. . . . T . . . . . - - - - - - . . . . T .
23	*Valerianella echinata* VEC0101	. . . . - - - - - - - - . . . . - . . . . G - - T T T . . . A . . . . T .
24	*Valerianella eriocarpa* VER0208	. . . . - - - - - - . . . . T .
25	*Valerianella eriocarpa* VER0402	. . . . - - - - - - . . . . T .
26	*Valerianella fusiformis* VFU0103	. . . . A . - - - - - - A . . . . C . . . . . T .
27	*Valerianella locusta* VLO0103	. . . . - - - - - - - - - . . . . G - - T T T . . . . T .
28	*Valerianella locusta* VLO0305	. . . . - - - - - - - - - . . . . G - - T T T . . . . T .
29	*Valerianella locusta* VLO0409	. . . . - - - - - - - - - . . . . G - - T T T . . . . T .
30	*Valerianella locusta* VLO0505	. . . . - - - - - - - - - . . . . G - - T T T . . . . T .
31	*Valerianella locusta* VLO0609	. . . . - - - - - - - - - . . . . G - - T T T . . . . T .
32	*Valerianella lusitanica* VLU0102	. . . . - - - - - - - - - . . . . - G - - T T T . . . . T .
33	*Valerianella microcarpa* VMI0102	. . . . - - - - - - . . . . T .
34	*Valerianella multidentata* VMU0201	. . . . T . . . . . - - - - - - . . . . T .
35	*Valerianella multidentata* VMU0301	. . . . T . . . . . - - - - - - . . . . T .
36	*Valerianella pumila* VPU0103	. . . . T . . . . . - - - - - - . . . . T .
37	*Valerianella pumila* VPU0304	. . . . T . . . . . - - - - - - . . . . T .
38	*Valerianella pumila* VPU0402	. . . . T . . . . . - - - - - - . . . . T .
39	*Valerianella rimosa* VRI0101	. . . . T . . . . - - - - - - . . . . T .
40	*Valerianella rimosa* VRI0201	. . . . T . . . . - - - - - - . . . . T .

	Position	127	128	129	130	131	132	133	134	135	136	137	138	139	140	141	142	143	144	145	146	147	148	149	150	151	152	153	154	155	156	157	158	159	160	161	162	163	164	165	166	167	168	169	170	171	172	173	174	175	176	177	178	179	180	181	182	183	184	185	186	187	188	189
1	*Centranthus calcitrapae* CC25	T	C	T	G	T	A	T	C	C	A	C	A	A	T	T	C	A	A	T	A	T	A	G	A	T	A	G	A	T	A	G	A	T	A	C	T	A	T	C	T	A	T	A	T	A	T	A	T	C	T	C	T	C	T	T	C	C	G	T	T	C	A	T
2	*Centranthus calcitrapae* CCACC539	.	.	.	.	.	.	.	.	.	.	.	.	.	.	.	.	.	.	.	.	.	.	.	.	.	.	.	.	.	.	.	.	.	.	.	.	.	.	.	.	.	.	.	.	.	.	.	.	.	.	.	.	.	.	.	.	.	.	.	.	.	.	.
3	*Fedia cornucopiae* FC73	.	.	.	.	.	.	.	.	.	.	.	.	.	.	.	.	.	.	.	.	.	.	.	.	.	G	.	.	.	.	T	C	.	C	T	C	.	.	.	C	.	A	.	.	.	.	G	.	T	.	Y	.	.	.	.	T	T	.	.	.	.	.	C
4	*Valerianella carinata* VCA0104	.	.	.	.	.	.	.	.	.	.	.	.	.	.	C	.	.	.	.	.	.	.	.	.	.	G	.	.	.	.	T	C	.	.	T	C	.	.	T	.	.	.	.	.	C	.	G	.	.	.	.	.	.	.	.	.	T	.	.	.	.	.	C
5	*Valerianella carinata* VCA0202	.	.	.	.	.	.	.	.	.	.	.	.	.	.	C	.	.	.	.	.	.	.	.	.	.	G	.	.	.	.	T	C	.	.	T	C	.	.	T	.	.	.	.	.	C	.	G	.	.	.	.	.	.	.	.	.	T	.	.	.	.	.	C
6	*Valerianella carinata* VCA0302	.	.	.	.	.	.	.	.	.	.	.	.	.	.	C	.	.	.	.	.	.	.	.	.	.	G	.	.	.	.	T	C	.	.	T	C	.	.	T	.	.	.	.	.	C	.	.	.	.	.	.	.	.	.	.	.	T	.	.	.	.	.	C
7	*Valerianella carinata* VCA0501	.	.	.	.	.	.	.	.	.	.	.	.	.	.	C	.	.	.	.	.	.	.	.	.	.	G	.	.	.	.	T	C	.	.	T	C	.	.	T	.	.	.	.	.	C	.	G	.	.	.	.	.	.	.	.	.	T	.	.	.	.	.	C
8	*Valerianella carinata* VCA0805	.	.	.	.	.	.	.	.	.	C	.	.	.	.	C	.	.	.	.	.	.	.	.	.	.	G	.	.	.	.	T	C	.	.	T	C	.	.	T	.	.	.	.	.	C	.	G	.	.	.	.	.	T	.	.	.	T	.	.	.	.	.	C
9	*Valerianella carinata* VCA0918	.	.	.	.	.	.	.	.	.	.	.	.	.	.	C	.	.	.	.	.	.	.	.	.	.	G	.	.	.	.	T	C	.	.	T	C	.	.	T	.	.	.	.	.	C	.	G	.	.	.	.	.	.	.	.	.	T	.	.	.	.	.	C
10	*Valerianella carinata* VCA1006	.	.	.	.	.	.	.	.	.	.	.	.	.	.	C	.	.	.	.	.	.	.	.	.	.	G	.	.	.	.	T	C	.	.	T	C	.	.	T	.	.	.	.	.	C	.	G	.	.	.	.	.	.	.	.	.	T	.	.	.	.	.	C
11	*Valerianella carinata* VCA1106	.	.	.	.	.	.	.	.	.	.	.	.	.	.	C	.	.	.	.	.	.	.	.	.	.	G	.	.	.	.	T	C	.	.	T	C	.	.	T	.	.	.	.	.	C	.	G	.	.	.	.	.	.	.	.	.	T	.	.	.	.	.	C
12	*Valerianella carinata* VCA1202	.	.	.	.	.	.	.	.	.	.	.	.	.	.	C	.	.	.	.	.	.	.	.	.	.	G	.	.	.	.	T	C	.	.	T	C	.	.	T	.	.	.	.	.	C	.	G	.	.	.	.	.	.	.	.	.	T	.	.	.	.	.	C
13	*Valerianella coronata* VCO0102	.	.	.	.	.	.	.	.	.	.	.	.	.	.	.	.	.	.	.	.	.	.	.	.	.	G	.	.	.	.	T	C	.	.	T	C	.	.	.	.	.	.	.	.	.	.	G	.	T	.	T	.	.	.	.	.	.	A	.	.	.	.	.
14	*Valerianella coronata* VCO0218	.	.	.	.	.	.	.	.	.	.	.	.	.	.	.	.	.	.	.	.	.	.	.	.	.	G	.	.	.	.	T	C	.	.	T	C	.	.	.	.	.	.	.	.	.	.	G	.	T	.	T	.	.	.	.	.	.	A	.	.	.	.	.
15	*Valerianella coronata* VCO0404	.	.	.	.	.	.	.	.	.	.	.	.	.	.	.	.	.	.	.	.	.	.	.	.	.	G	.	.	.	.	T	C	.	.	T	C	.	.	.	.	.	.	.	.	.	.	G	.	T	.	T	.	.	.	.	.	.	A	.	.	.	.	.
16	*Valerianella coronata* VCO0508	.	.	.	.	.	.	.	.	.	.	.	.	.	.	.	.	.	.	.	.	.	.	.	.	.	G	.	.	.	.	T	C	.	.	T	C	.	.	.	.	.	.	.	.	.	.	G	.	T	.	T	.	.	.	.	.	.	A	.	.	.	.	.
17	*Valerianella coronata* VCO0705	.	.	.	.	.	.	.	.	.	.	.	.	.	.	.	.	.	.	.	.	.	.	.	.	.	G	.	.	.	.	T	C	.	.	T	C	.	.	.	.	.	.	.	.	.	.	G	.	T	.	T	.	.	.	.	.	.	A	.	.	.	.	.
18	*Valerianella coronata* VCO0805	.	.	.	.	.	.	.	.	.	.	.	.	.	.	.	.	.	.	.	.	.	.	.	.	.	G	.	.	.	.	T	C	.	.	T	C	.	.	.	.	.	.	.	.	.	.	G	.	T	.	T	.	.	.	.	.	.	A	.	.	.	.	.
19	*Valerianella coronata* VCO0901	.	.	.	.	.	.	.	.	.	.	.	.	.	.	.	.	.	.	.	.	.	.	.	.	.	G	.	.	.	.	T	C	.	.	T	C	.	.	.	.	.	.	.	.	.	.	G	.	T	.	T	.	.	.	.	.	.	A	.	.	.	.	.
20	*Valerianella dentata* VDE0303	.	.	.	A	.	.	.	G	.	.	.	.	.	.	.	.	.	.	.	.	.	.	.	.	.	G	.	.	.	.	T	C	.	.	T	C	.	.	.	.	.	.	.	.	.	.	G	.	.	.	.	.	.	.	.	.	.	A	.	.	.	.	.
21	*Valerianella discoidea* VDI0202	.	.	.	.	.	.	.	.	.	.	.	.	.	.	.	.	.	.	.	.	.	.	.	.	.	G	.	.	.	.	T	C	.	.	T	C	.	.	.	.	.	.	.	.	.	.	G	.	T	.	T	.	.	.	.	.	.	A	.	.	.	.	.
22	*Valerianella discoidea* VDI0412	.	.	.	.	.	.	.	.	.	.	.	.	.	.	.	.	.	.	.	.	.	.	.	.	.	G	.	.	.	.	T	C	.	.	T	C	.	.	.	.	.	.	.	.	.	.	G	.	T	.	T	.	.	.	.	.	.	A	.	.	.	.	.
23	*Valerianella echinata* VEC0101	.	.	.	.	.	.	.	.	.	.	.	.	.	.	.	.	.	.	.	.	.	.	.	.	.	G	.	.	.	.	T	C	.	.	T	C	.	.	.	.	.	.	.	.	C	.	G	.	.	.	.	.	.	.	.	.	T	.	.	.	.	.	C
24	*Valerianella eriocarpa* VER0208	.	.	.	A	.	.	.	.	.	.	.	.	.	.	.	.	.	.	.	.	.	.	.	.	.	G	.	.	.	.	T	C	.	.	T	C	.	.	.	.	.	.	.	.	.	.	G	.	.	.	.	.	.	.	.	.	.	A	.	.	.	.	.
25	*Valerianella eriocarpa* VER0402	.	.	.	A	.	.	.	.	.	.	.	.	.	.	.	.	.	.	.	.	.	.	.	.	.	G	.	.	.	.	T	C	.	.	T	C	.	.	.	.	.	.	.	.	.	.	G	.	.	.	.	.	.	.	.	.	.	A	.	.	.	.	.
26	*Valerianella fusiformis* VFU0103	.	.	.	.	.	.	.	.	.	.	.	.	.	.	.	.	.	.	.	.	.	.	.	G	.	G	.	.	.	.	T	C	.	.	T	.	.	.	.	.	.	.	.	.	.	.	.	.	.	.	.	.	.	.	.	.	.	A	.	.	.	.	.
27	*Valerianella locusta* VLO0103	.	.	.	.	.	.	.	.	.	.	.	.	.	.	C	.	.	.	.	.	.	.	.	.	.	G	.	.	.	.	T	C	.	.	T	C	.	.	T	.	.	.	.	.	C	.	G	.	.	.	.	.	.	.	.	.	T	.	.	.	.	.	C
28	*Valerianella locusta* VLO0305	.	.	.	.	.	.	.	.	.	.	.	.	.	.	C	.	.	.	.	.	.	.	.	.	.	G	.	.	.	.	T	C	.	.	T	C	.	.	T	.	.	.	.	.	C	.	G	.	.	.	.	.	.	.	.	.	T	.	.	.	.	.	C
29	*Valerianella locusta* VLO0409	.	.	.	.	.	.	.	.	.	.	.	.	.	.	C	.	.	.	.	.	.	.	.	.	.	G	.	.	.	.	T	C	.	.	T	C	.	.	T	.	.	.	.	.	C	.	G	.	.	.	.	.	.	.	.	.	T	.	.	.	.	.	C
30	*Valerianella locusta* VLO0505	.	.	.	.	.	.	.	.	.	.	.	.	.	.	C	.	.	.	.	.	.	.	.	.	.	G	.	.	.	.	T	C	.	.	T	C	.	.	T	.	.	.	.	.	C	.	G	.	.	.	.	.	.	.	.	.	T	.	.	.	.	.	C
31	*Valerianella locusta* VLO0609	.	.	.	.	.	.	.	.	.	.	.	.	.	.	C	.	.	.	.	.	.	.	.	.	.	G	.	.	.	.	T	C	.	.	T	C	.	.	T	.	.	.	.	.	C	.	G	.	.	.	.	.	.	.	.	.	T	.	.	.	.	.	C
32	*Valerianella lusitanica* VLU0102	.	.	.	.	.	.	.	.	.	.	.	.	.	.	C	.	.	.	.	.	.	.	.	.	.	G	.	.	.	.	T	C	.	.	T	C	.	.	T	.	.	.	.	.	C	.	G	.	.	.	.	.	.	.	.	.	T	.	.	.	.	.	C
33	*Valerianella microcarpa* VMI0102	.	.	.	A	.	.	.	.	.	.	.	.	.	.	.	.	.	.	.	.	.	.	.	.	.	G	.	.	.	.	T	C	.	.	T	C	.	.	.	.	.	.	.	.	.	.	G	.	.	.	.	.	.	.	.	.	.	A	.	.	.	.	.
34	*Valerianella multidentata* VMU0201	.	.	.	.	.	.	.	.	.	.	.	.	.	.	.	.	.	.	.	.	.	.	.	.	.	G	.	.	.	.	T	C	.	.	T	C	.	.	.	.	.	.	.	.	.	.	G	.	T	.	T	.	.	.	.	.	.	A	.	.	.	.	.
35	*Valerianella multidentata* VMU0301	.	.	.	.	.	.	.	.	.	.	.	.	.	.	.	.	.	.	.	.	.	.	.	.	.	G	.	.	.	.	T	C	.	.	T	C	.	.	.	.	.	.	.	.	.	.	G	.	T	.	T	.	.	.	.	.	.	A	.	.	.	.	.
36	*Valerianella pumila* VPU0103	.	.	.	.	.	.	.	.	.	T	.	.	.	.	.	.	.	.	.	.	.	.	.	.	.	G	.	.	.	.	T	C	.	.	T	C	.	.	.	.	.	.	.	.	.	.	G	.	T	.	T	.	.	.	.	.	.	A	.	.	.	.	.
37	*Valerianella pumila* VPU0304	.	.	.	.	.	.	.	.	.	.	.	.	.	.	.	.	.	.	.	.	.	.	.	.	.	G	.	.	.	.	T	C	.	.	T	C	.	.	.	.	.	.	.	.	.	.	G	.	T	.	T	.	.	.	.	.	.	A	.	.	.	.	.
38	*Valerianella pumila* VPU0402	.	.	.	.	.	.	.	.	.	.	.	.	.	.	.	.	.	.	.	.	.	.	.	.	.	G	.	.	.	.	T	C	.	.	T	C	.	.	.	.	.	.	.	.	.	.	G	.	T	.	T	.	.	.	.	.	.	A	.	.	.	.	.
39	*Valerianella rimosa* VRI0101	.	.	.	A	.	.	.	G	.	.	.	.	.	.	.	.	.	.	.	.	.	.	.	.	.	G	.	.	.	.	T	C	.	.	T	C	.	.	.	.	.	.	.	.	.	.	G	.	.	.	.	.	.	.	.	.	.	A	.	.	.	.	.
40	*Valerianella rimosa* VRI0201	.	.	.	A	.	.	.	G	.	.	.	.	.	.	.	.	.	.	.	.	.	.	.	.	.	G	.	.	.	.	T	C	.	.	T	C	.	.	.	.	.	.	.	.	.	.	G	.	.	.	.	.	.	.	.	.	.	A	.	.	.	.	.

Position

		190	191	192	193	194	195	196	197	198	199	200	201	202	203	204	205	206	207	208	209	210	211	212	213	214	215	216	217	218	219	220	221	222	223	224	225	226	227	228	229	230	231	232	233	234	235	236	237	238	239	240	241	242	243	244	245	246	247	248	249	250	251	252
1	*Centranthus calcitrapae* CC25	T	T	T	C	C	C	A	A	T	G	T	T	A	T	T	T	G	G	A	A	T	A	C	T	T	G	A	A	C	G	G	T	C	G	A	T	T	C	T	T	T	T	T	T	T	T	T	T	C	G	T	C	T	T	C	A	C	T	T	T	C	A	C
2	*Centranthus calcitrapae* CCACC539	.	.	.	.	.	.	.	.	.	.	.	.	.	.	.	.	.	.	.	.	.	.	.	.	.	.	.	.	.	.	.	.	.	.	.	.	.	.	.	.	.	.	.	.	.	.	.	.	.	.	.	.	.	.	.	.	.	.	.	.	.	.	.
3	*Fedia cornucopiae* FC73	.	.	.	T	.	.	.	.	.	.	.	C	.	.	.	.	.	.	.	.	.	.	.	.	.	.	.	.	.	.	.	.	.	.	.	.	.	.	.	.	.	.	.	.	.	-	-	-	.	T	.	T	.	.	.	.	.	.	.	.	.	M	.
4	*Valerianella carinata* VCA0104	.	.	.	T	.	.	.	.	.	.	.	C	.	.	.	.	.	.	.	.	.	.	.	.	.	.	.	.	.	.	A	.	.	.	.	.	.	.	.	.	.	.	.	.	-	-	-	-	.	A	.	.	.	.	T	.	.	.	.	.	.	.	.
5	*Valerianella carinata* VCA0202	.	.	.	T	.	.	.	.	.	.	.	C	.	.	.	.	.	.	.	.	.	.	.	.	.	.	.	.	.	.	A	.	.	.	.	.	.	.	.	.	.	.	.	.	-	-	-	-	.	A	.	.	.	.	T	.	.	.	.	.	.	.	.
6	*Valerianella carinata* VCA0302	.	.	.	T	.	A	.	.	.	.	.	C	.	.	.	.	.	.	.	.	.	.	.	.	.	.	.	.	.	.	A	.	.	.	.	.	.	.	.	.	.	.	.	.	-	-	-	-	.	A	.	.	.	.	T	.	.	.	.	.	.	.	.
7	*Valerianella carinata* VCA0501	.	.	.	T	.	.	.	.	.	.	.	C	.	.	.	.	.	.	.	.	.	.	.	.	.	.	.	.	.	.	A	.	.	.	.	.	.	.	.	.	.	.	.	.	-	-	-	-	.	A	.	.	.	.	T	.	.	.	.	.	.	.	.
8	*Valerianella carinata* VCA0805	.	.	.	T	.	.	.	.	.	.	.	C	.	.	.	.	.	.	.	.	.	.	.	.	.	.	.	.	.	.	A	.	.	.	.	.	.	.	.	.	.	.	.	.	-	-	-	-	.	A	.	.	.	.	T	.	.	.	.	.	.	.	.
9	*Valerianella carinata* VCA0918	.	.	.	T	.	.	.	.	.	.	.	C	.	.	.	.	.	.	.	.	.	.	.	.	.	.	.	.	.	.	A	.	.	.	.	.	.	.	.	.	.	.	.	.	-	-	-	-	.	A	.	.	.	.	T	.	.	.	.	.	.	.	.
10	*Valerianella carinata* VCA1006	.	.	.	T	.	.	.	.	.	.	.	C	.	.	.	.	.	.	.	.	.	.	.	.	.	.	.	.	.	.	A	.	.	.	.	.	.	.	.	.	.	.	.	.	-	-	-	-	.	A	.	.	.	.	T	.	.	.	.	.	.	.	.
11	*Valerianella carinata* VCA1106	.	.	.	T	.	.	.	.	.	.	.	C	.	.	.	.	.	.	.	.	.	.	.	.	.	.	.	.	.	.	A	.	.	.	.	.	.	.	.	.	.	.	.	.	-	-	-	-	.	A	.	.	.	.	T	.	.	.	.	.	.	.	.
12	*Valerianella carinata* VCA1202	.	.	.	T	.	.	.	.	.	.	.	C	.	.	.	.	.	.	.	.	.	.	.	.	.	.	.	.	.	.	A	.	.	.	.	.	.	.	.	.	.	.	.	.	-	-	-	-	.	A	.	.	.	.	T	.	.	.	.	.	.	.	.
13	*Valerianella coronata* VCO0102	.	.	.	T	.	.	.	.	.	.	.	C	.	.	.	.	.	.	.	G	.	.	.	.	.	.	.	.	.	.	.	.	.	.	.	.	.	.	.	.	.	.	.	.	.	-	-	-	.	A	.	.	.	.	.	.	.	.	.	.	T	G	.
14	*Valerianella coronata* VCO0218	.	.	.	T	.	.	.	.	.	.	.	C	.	.	.	.	.	.	.	G	.	.	.	.	.	.	.	.	.	.	.	.	.	.	.	.	.	.	.	.	.	.	.	.	.	-	-	-	.	A	.	.	.	.	.	.	.	.	.	.	T	G	.
15	*Valerianella coronata* VCO0404	.	.	.	T	.	.	.	.	.	.	.	C	.	.	.	.	.	.	.	G	.	.	.	.	.	.	.	.	.	.	.	.	.	.	.	.	.	.	.	.	.	.	.	.	.	-	-	-	.	A	.	.	.	.	.	.	.	.	.	.	T	G	.
16	*Valerianella coronata* VCO0508	.	.	.	T	.	.	.	.	.	.	.	C	.	.	.	.	.	.	.	G	.	.	.	.	.	.	.	.	.	.	.	.	.	.	.	.	.	.	.	.	.	.	.	.	.	-	-	-	.	A	.	.	.	.	.	.	.	.	.	.	T	G	.
17	*Valerianella coronata* VCO0705	.	.	.	T	.	.	.	.	.	.	.	C	.	.	.	.	.	.	.	G	.	.	.	.	.	.	.	.	.	.	.	.	.	.	.	.	.	.	.	.	.	.	.	.	.	-	-	-	.	A	.	.	.	.	.	.	.	.	.	.	T	G	.
18	*Valerianella coronata* VCO0805	.	.	.	T	.	.	.	.	.	.	.	C	.	.	.	.	.	.	.	G	.	.	.	.	.	.	.	.	.	.	.	.	.	.	.	.	.	.	.	.	.	.	.	.	.	-	-	-	.	A	.	.	.	.	.	.	.	.	.	.	T	G	.
19	*Valerianella coronata* VCO0901	.	.	.	T	.	.	.	.	.	.	.	C	.	.	.	.	.	.	.	G	.	.	.	.	.	.	.	.	.	.	.	.	.	.	.	.	.	.	.	.	.	.	.	.	.	-	-	-	.	A	.	.	.	.	.	.	.	.	.	.	T	G	.
20	*Valerianella dentata* VDE0303	.	.	.	T	.	.	.	.	.	.	.	C	.	.	.	.	.	A	.	G	.	.	.	.	.	.	.	.	.	.	.	.	.	.	.	.	.	.	.	.	.	.	.	.	.	-	-	-	.	A	.	.	.	.	.	.	.	.	.	.	.	G	.
21	*Valerianella discoidea* VDI0202	.	.	.	T	.	.	.	.	.	.	.	C	.	.	.	.	.	.	.	G	.	.	.	.	.	.	.	.	.	.	.	.	.	.	.	.	.	.	.	.	.	.	.	.	.	-	-	-	.	A	.	.	.	.	.	.	.	.	.	.	T	G	.
22	*Valerianella discoidea* VDI0412	.	.	.	T	.	.	.	.	.	.	.	C	.	.	.	.	.	.	.	G	.	.	.	.	.	.	.	.	.	.	.	.	.	.	.	.	.	.	.	.	.	.	.	.	.	-	-	-	.	A	.	.	.	.	.	.	.	.	.	.	T	G	.
23	*Valerianella echinata* VEC0101	.	.	.	T	.	.	.	.	.	.	.	C	.	.	.	.	.	.	.	.	.	.	.	.	.	.	.	.	.	.	.	.	.	.	.	.	.	.	.	.	.	.	.	.	.	-	-	-	.	A	.	.	.	.	.	.	.	A	.	.	.	.	.
24	*Valerianella eriocarpa* VER0208	.	.	.	T	.	.	.	.	.	.	.	C	.	.	.	.	.	.	.	G	.	.	.	.	.	.	.	.	.	.	.	.	.	.	.	.	.	.	.	.	.	.	.	.	-	-	-	-	.	A	.	.	.	.	.	.	.	.	.	.	.	G	.
25	*Valerianella eriocarpa* VER0402	.	.	.	T	.	.	.	.	.	.	.	C	.	.	.	.	.	.	.	G	.	.	.	.	.	.	.	.	.	.	.	.	.	.	.	.	.	.	.	.	.	.	.	.	-	-	-	-	.	A	.	.	.	.	.	.	.	.	.	.	.	G	.
26	*Valerianella fusiformis* VFU0103	.	.	.	T	.	.	.	.	.	.	.	.	.	.	.	.	.	.	T	.	.	.	.	.	.	.	.	.	.	.	.	.	.	.	.	.	.	.	.	.	.	.	.	.	.	-	-	-	.	A	.	.	.	.	.	.	.	.	.	.	T	.	.
27	*Valerianella locusta* VLO0103	.	.	.	T	.	.	.	.	.	.	.	C	.	.	.	.	.	.	.	.	.	.	.	.	.	.	.	.	.	.	A	.	.	.	.	.	.	.	.	.	.	.	.	.	-	-	-	-	.	A	.	.	.	.	T	.	.	.	.	.	.	.	.
28	*Valerianella locusta* VLO0305	.	.	.	T	.	.	.	.	.	.	.	C	.	.	.	.	.	.	.	.	.	.	.	.	.	.	.	.	.	.	A	.	.	.	.	.	.	.	.	.	.	.	.	.	-	-	-	-	.	A	.	.	.	.	T	.	.	.	.	.	.	.	.
29	*Valerianella locusta* VLO0409	.	.	.	T	.	.	.	.	.	.	.	C	.	.	.	.	.	.	.	.	.	.	.	.	.	.	.	.	.	.	A	.	.	.	.	.	.	.	.	.	.	.	.	.	-	-	-	-	.	A	.	.	.	.	T	.	.	.	.	.	.	.	.
30	*Valerianella locusta* VLO0505	.	.	.	T	.	.	.	.	.	.	.	C	.	.	.	.	.	.	.	.	.	.	.	.	.	.	.	.	.	.	A	.	.	.	.	.	.	.	.	.	.	.	.	.	-	-	-	-	.	A	.	.	.	.	T	.	.	.	.	.	.	.	.
31	*Valerianella locusta* VLO0609	.	.	.	T	.	.	.	.	.	.	.	C	.	.	.	.	.	.	.	.	.	.	.	.	.	.	.	.	.	.	A	.	.	.	.	.	.	.	.	.	.	.	.	.	-	-	-	-	.	A	.	.	.	.	T	.	.	.	.	.	.	.	.
32	*Valerianella lusitanica* VLU0102	.	.	.	T	.	.	.	.	.	.	.	C	.	.	.	.	.	.	.	.	.	.	.	.	.	.	.	.	.	.	A	.	.	.	.	.	.	.	.	.	.	.	.	.	-	-	-	-	.	A	.	.	.	.	T	.	.	.	.	.	.	.	.
33	*Valerianella microcarpa* VMI0102	.	.	.	T	.	.	.	.	.	.	.	C	.	.	.	.	.	.	.	G	.	.	.	.	.	.	.	.	.	.	.	.	.	.	.	.	.	.	.	.	.	.	.	.	-	-	-	-	.	A	.	.	.	.	.	.	.	.	.	.	.	G	.
34	*Valerianella multidentata* VMU0201	.	.	.	T	.	.	.	.	.	.	.	C	.	.	.	.	.	.	.	G	.	.	.	.	.	.	.	.	.	.	.	.	.	.	.	.	.	.	.	.	.	.	.	.	.	-	-	-	.	A	.	.	.	.	.	.	.	.	.	.	T	G	.
35	*Valerianella multidentata* VMU0301	.	.	.	T	.	.	.	.	.	.	.	C	.	.	.	.	.	.	.	G	.	.	.	.	.	.	.	.	.	.	.	.	.	.	.	.	.	.	.	.	.	.	.	.	.	-	-	-	.	A	.	.	.	.	.	.	.	.	.	.	T	G	.
36	*Valerianella pumila* VPU0103	.	.	.	T	.	.	.	.	.	.	.	C	.	.	.	.	.	.	.	G	.	.	.	.	.	.	.	.	.	.	.	.	.	.	.	.	.	.	.	.	.	.	.	.	.	-	-	-	.	A	.	.	.	.	.	.	.	.	.	.	T	G	.
37	*Valerianella pumila* VPU0304	.	.	.	T	.	.	.	.	.	.	.	C	.	.	.	.	.	.	.	G	.	.	.	.	.	.	.	.	.	.	.	.	.	.	.	.	.	.	.	.	.	.	.	.	.	-	-	-	.	A	.	.	.	.	.	.	.	.	.	.	T	G	.
38	*Valerianella pumila* VPU0402	.	.	.	T	.	.	.	.	.	.	.	C	.	.	.	.	.	.	.	G	.	.	.	.	.	.	.	.	.	.	.	.	.	.	.	.	.	.	.	.	.	.	.	.	.	-	-	-	.	A	.	.	.	.	.	.	.	.	.	.	T	G	.
39	*Valerianella rimosa* VRI0101	.	.	.	T	.	.	.	.	.	.	.	C	.	.	.	.	.	A	.	G	.	.	.	.	.	.	.	.	.	.	.	.	.	.	.	.	.	.	.	.	.	.	.	.	.	-	-	-	.	A	.	.	.	.	.	.	.	.	.	.	.	G	.
40	*Valerianella rimosa* VRI0201	.	.	.	T	.	.	.	.	.	.	.	C	.	.	.	.	.	A	.	G	.	.	.	.	.	.	.	.	.	.	.	.	.	.	.	.	.	.	.	.	.	.	.	.	.	-	-	-	.	A	.	.	.	.	.	.	.	.	.	.	.	G	.

Position

No.	Taxon	Sample	253	254	255	256	257	258	259	260	261	262	263	264	265	266	267	268	269	270	271	272	273	274	275	276	277	278	279	280	281	282	283	284	285	286	287	288	289	290	291	292	293	294	295	296	297	298	299	300	301	302	303	304	305	306	307	308	309	310	311	312	313	314	315
1	*Centranthus calcitrapae*	CC25	G	T	T	T	A	C	G	A	A	T	G	A	A	A	G	G	G	T	A	G	G	A	A	T	-	-	-	-	-	A	T	C	T	G	A	T	T	T	G	T	T	C	T	A	A	C	G	A	A	T	C	C	T	T	-	-	-	T	T	C	T	G	A
2	*Centranthus calcitrapae*	CCACC539	.	.	.	.	.	.	.	.	.	.	.	.	.	.	.	.	.	.	.	.	.	.	.	.	-	-	-	-	-	.	.	.	.	.	.	.	.	.	.	.	.	.	.	.	.	.	.	.	.	.	.	.	.	.	-	-	-	.	.	.	.	.	.
3	*Fedia cornucopiae*	FC73	.	.	.	.	.	.	.	.	.	.	.	.	.	.	.	.	A	.	.	.	.	.	.	.	G	A	A	A	T	.	.	T	.	.	.	.	.	A	.	.	.	T	.	.	.	G	.	.	.	C	T	Y	.	.	A	A	A	.	G	T	.	.	.
4	*Valerianella carinata*	VCA0104	.	.	.	.	.	.	.	.	.	.	.	.	.	.	.	T	.	.	.	.	.	.	.	.	G	A	A	A	T	.	.	.	.	.	.	.	.	A	.	.	.	.	.	.	.	.	.	.	.	C	.	.	.	.	A	A	A	.	G	.	.	.	.
5	*Valerianella carinata*	VCA0202	.	.	.	.	.	.	.	.	.	.	.	.	.	.	.	T	.	.	.	.	.	.	.	.	G	A	A	A	T	.	.	.	.	.	.	.	.	A	.	.	.	.	.	.	.	.	.	.	.	.	.	.	.	.	A	A	A	.	G	.	.	.	.
6	*Valerianella carinata*	VCA0302	.	.	.	.	.	.	.	.	.	.	.	.	.	.	.	T	.	.	.	.	.	.	.	.	G	A	A	A	T	.	.	.	.	.	.	.	.	A	.	.	.	.	.	.	.	.	.	.	.	C	.	.	.	.	A	A	A	.	G	.	.	.	.
7	*Valerianella carinata*	VCA0501	.	.	.	.	.	.	.	.	.	.	.	.	.	.	.	T	.	.	.	.	T	.	.	.	G	A	A	A	T	.	.	.	.	.	.	.	.	A	.	.	.	.	.	.	.	.	.	.	.	.	.	.	.	.	A	A	A	.	G	.	.	.	.
8	*Valerianella carinata*	VCA0805	.	.	.	.	.	.	.	.	.	.	.	.	.	.	.	T	.	.	.	.	.	.	.	.	G	A	A	A	T	.	.	T	.	.	.	.	.	A	.	.	.	.	.	.	.	.	.	.	.	.	.	.	.	.	A	A	A	.	G	.	.	.	.
9	*Valerianella carinata*	VCA0918	.	.	.	.	.	.	.	.	.	.	.	.	.	.	.	T	.	.	.	.	.	.	.	.	G	A	A	A	T	.	.	.	.	.	.	.	.	A	.	.	.	.	.	.	.	.	.	.	.	.	.	.	.	.	A	A	A	.	G	.	.	.	.
10	*Valerianella carinata*	VCA1006	.	.	.	.	.	.	.	.	.	.	.	.	.	.	.	T	.	.	.	.	T	.	.	.	G	A	A	A	T	.	.	.	.	.	.	.	.	A	.	.	.	.	.	.	.	.	.	.	.	.	.	.	.	.	A	A	A	.	G	.	.	.	.
11	*Valerianella carinata*	VCA1106	.	.	.	.	.	.	.	.	.	.	.	.	.	.	.	T	.	.	.	.	T	.	.	.	G	A	A	A	T	.	.	.	.	.	.	.	.	A	.	.	.	.	.	.	.	.	.	.	.	.	.	.	.	.	A	A	A	.	G	.	.	.	.
12	*Valerianella carinata*	VCA1202	.	.	.	.	.	.	.	.	.	.	.	.	.	.	.	T	.	.	.	.	T	.	.	.	G	A	A	A	T	.	.	.	.	.	.	.	.	A	.	.	.	.	.	.	.	.	.	.	.	.	.	.	.	.	A	A	A	.	G	.	.	.	.
13	*Valerianella coronata*	VCO0102	.	.	.	.	.	.	.	.	.	.	.	.	.	.	.	.	.	.	G	.	.	.	.	.	G	A	A	A	T	.	.	.	G	.	.	.	.	A	.	.	.	.	.	.	.	.	.	.	.	C	.	.	.	.	A	C	-	.	G	.	.	.	.
14	*Valerianella coronata*	VCO0218	.	.	.	.	.	.	.	.	.	.	.	.	.	.	.	.	.	.	G	.	.	.	.	.	G	A	A	A	T	.	.	.	G	.	.	.	.	A	.	.	.	.	.	.	.	.	.	.	.	C	.	.	.	.	A	C	-	.	G	.	.	.	.
15	*Valerianella coronata*	VCO0404	.	.	.	.	.	.	.	.	.	.	.	.	.	.	.	.	.	.	G	.	.	.	.	.	G	A	A	A	T	.	.	.	G	.	.	.	.	A	.	.	.	.	.	.	.	.	.	.	.	C	.	.	.	.	A	C	-	.	G	.	.	.	.
16	*Valerianella coronata*	VCO0508	.	.	.	.	.	.	.	.	.	.	.	.	.	.	.	.	.	.	G	.	.	.	.	.	G	A	A	A	T	.	.	.	G	.	.	.	.	A	.	.	.	.	.	.	.	.	.	.	.	C	.	.	.	.	A	C	-	.	G	.	.	.	.
17	*Valerianella coronata*	VCO0705	.	.	.	.	.	.	.	.	.	.	.	.	.	.	.	.	.	.	G	.	.	.	.	.	G	A	A	A	T	.	.	.	G	.	.	.	.	A	.	.	.	.	.	.	.	.	.	.	.	C	.	.	.	.	A	C	-	.	G	.	.	.	.
18	*Valerianella coronata*	VCO0805	.	.	.	.	.	.	.	.	.	.	.	.	.	.	.	.	.	.	G	.	.	.	.	.	G	A	A	A	T	.	.	.	G	.	.	.	.	A	.	.	.	.	.	.	.	.	.	.	.	C	.	.	.	.	A	C	-	.	G	.	.	.	.
19	*Valerianella coronata*	VCO0901	.	.	.	.	.	.	.	.	.	.	.	.	.	.	.	.	.	.	G	.	.	.	.	.	G	A	A	A	T	.	.	.	G	.	.	.	.	A	.	.	.	.	.	.	.	.	.	.	.	C	.	.	.	.	A	C	-	.	G	.	.	.	.
20	*Valerianella dentata*	VDE0303	.	.	.	.	.	.	.	.	.	.	.	.	.	.	.	A	.	.	G	.	.	.	.	.	G	A	A	A	T	.	.	.	.	.	.	.	.	A	.	.	.	.	.	.	.	.	.	.	.	C	.	.	.	.	A	C	-	.	G	.	.	.	.
21	*Valerianella discoidea*	VDI0202	.	.	.	.	.	.	.	.	.	.	.	.	.	.	.	.	.	.	G	.	.	.	.	.	G	A	A	A	T	.	.	.	G	.	.	.	.	A	.	.	.	.	.	.	.	.	.	.	.	C	.	.	.	.	A	C	-	.	G	.	.	.	.
22	*Valerianella discoidea*	VDI0412	.	.	.	.	.	.	.	.	.	.	.	.	.	.	.	.	.	.	G	.	.	.	.	.	G	A	A	A	T	.	.	.	G	.	.	.	.	A	.	.	.	.	.	.	.	.	.	.	.	C	.	.	.	.	A	C	-	.	G	.	.	.	.
23	*Valerianella echinata*	VEC0101	.	.	.	.	.	.	.	.	.	.	.	.	.	.	.	-	-	-	-	-	-	-	-	-	-	-	-	-	-	-	-	-	-	-	-	.	.	A	.	.	.	.	.	.	.	.	.	.	.	C	.	.	.	.	A	A	A	.	G	.	C	.	.
24	*Valerianella eriocarpa*	VER0208	.	.	.	.	.	.	.	.	.	.	.	.	.	.	.	.	.	C	G	.	.	.	.	.	G	A	A	A	T	.	.	.	.	.	.	.	.	A	.	.	.	.	.	.	.	.	.	.	.	C	.	.	.	.	A	C	-	.	G	.	.	.	.
25	*Valerianella eriocarpa*	VER0402	.	.	.	.	.	.	.	.	.	.	.	.	.	.	.	.	.	C	G	.	.	.	.	.	G	A	A	A	T	.	.	.	.	.	.	.	.	A	.	.	.	.	.	.	.	.	.	.	.	C	.	.	.	.	A	C	-	.	G	.	.	.	.
26	*Valerianella fusiformis*	VFU0103	.	.	.	.	.	.	.	.	.	G	.	.	.	.	.	.	.	.	.	.	.	.	.	C	G	A	A	A	T	.	.	.	.	.	.	.	.	A	.	.	.	.	.	.	.	.	.	.	.	C	.	.	.	.	A	C	-	.	G	.	.	.	.
27	*Valerianella locusta*	VLO0103	.	.	.	.	.	.	.	.	.	.	.	.	.	.	.	T	.	.	.	.	T	.	.	.	G	A	A	A	T	.	.	.	.	.	.	.	.	A	.	.	.	.	.	.	.	.	.	.	.	.	.	.	.	.	A	A	A	.	G	.	.	.	.
28	*Valerianella locusta*	VLO0305	.	.	.	.	.	.	.	.	.	.	.	.	.	.	.	T	.	.	.	.	T	.	.	.	G	A	A	A	T	.	.	.	.	.	.	.	.	A	.	.	.	.	.	.	.	.	.	.	.	.	.	.	.	.	A	A	A	.	G	.	.	.	.
29	*Valerianella locusta*	VLO0409	.	.	.	.	.	.	.	.	.	.	.	.	.	.	.	T	.	.	.	.	T	.	.	.	G	A	A	A	T	.	.	.	.	.	.	.	.	A	.	.	.	.	.	.	.	.	.	.	.	.	.	.	.	.	A	A	A	.	G	.	.	.	.
30	*Valerianella locusta*	VLO0505	.	.	.	.	.	.	.	.	.	.	.	.	.	.	.	T	.	.	.	.	T	.	.	.	G	A	A	A	T	.	.	.	.	.	.	.	.	A	.	.	.	.	.	.	.	.	.	.	.	.	.	.	.	.	A	A	A	.	G	.	.	.	.
31	*Valerianella locusta*	VLO0609	.	.	.	.	.	.	.	.	.	.	.	.	.	.	.	T	.	.	.	.	.	.	.	.	G	A	A	A	T	.	.	.	.	.	.	.	.	A	.	.	.	.	.	.	.	.	.	.	.	.	.	.	.	.	A	A	A	.	G	.	.	.	.
32	*Valerianella lusitanica*	VLU0102	.	.	.	.	.	.	.	.	.	.	.	.	.	.	.	T	.	.	.	.	.	.	.	.	G	A	A	A	T	.	.	.	.	.	.	.	.	A	.	.	.	.	.	.	.	.	.	.	.	C	.	.	.	.	A	A	A	.	G	.	.	.	.
33	*Valerianella microcarpa*	VMI0102	.	.	.	.	.	.	.	.	.	.	.	.	.	.	.	C	.	C	G	A	.	.	.	.	G	A	A	A	T	.	.	.	.	.	.	.	.	A	.	.	.	.	.	.	.	.	.	.	.	C	.	.	.	.	A	C	-	.	G	.	.	.	.
34	*Valerianella multidentata*	VMU0201	.	.	.	.	.	.	.	.	.	.	.	.	.	.	.	.	.	.	G	.	.	.	.	.	G	A	A	A	T	.	.	.	G	.	.	.	.	A	.	.	.	.	.	.	.	.	.	.	.	C	.	.	.	.	A	C	-	.	G	.	.	.	.
35	*Valerianella multidentata*	VMU0301	.	.	.	.	.	.	.	.	.	.	.	.	.	.	.	.	.	.	G	.	.	.	.	.	G	A	A	A	T	.	.	.	G	.	.	.	.	A	.	.	.	.	.	.	.	.	.	.	.	C	.	.	.	.	A	C	-	.	G	.	.	.	.
36	*Valerianella pumila*	VPU0103	.	.	.	.	.	.	.	.	.	.	.	.	.	.	.	.	.	.	G	.	.	.	.	.	G	A	A	A	T	.	.	.	G	.	.	.	.	A	.	.	.	.	.	.	.	.	.	.	.	C	.	.	.	.	A	C	-	.	G	.	.	.	.
37	*Valerianella pumila*	VPU0304	.	.	.	.	.	.	.	.	.	.	.	.	.	.	.	.	.	.	G	.	.	.	.	.	G	A	A	A	T	.	.	.	G	.	.	.	.	A	.	.	.	.	.	.	.	.	.	.	.	C	.	.	.	.	A	C	-	.	G	.	.	.	.
38	*Valerianella pumila*	VPU0402	.	.	.	.	.	.	.	.	.	.	.	.	.	.	.	.	.	.	G	.	.	.	.	.	G	A	A	A	T	.	.	.	G	.	.	.	.	A	.	.	.	.	.	.	.	.	.	.	.	C	.	.	.	.	A	C	-	.	G	.	.	.	.
39	*Valerianella rimosa*	VRI0101	.	.	.	.	.	.	.	.	.	.	.	.	.	.	.	A	.	.	G	.	.	.	.	.	G	A	A	A	T	.	.	.	.	.	.	.	.	A	.	.	.	.	.	.	.	.	.	.	.	C	.	.	.	.	A	C	-	.	G	.	.	.	.
40	*Valerianella rimosa*	VRI0201	.	.	.	.	.	.	.	.	.	.	.	.	.	.	.	A	.	.	G	.	.	.	.	.	G	A	A	A	T	.	.	.	.	.	.	.	.	A	.	.	.	.	.	.	.	.	.	.	.	C	.	.	.	.	A	C	-	.	G	.	.	.	.

Position

		316	317	318	319	320	321	322	323	324	325	326	327	328	329	330	331	332	333	334	335	336	337	338	339	340	341	342	343	344	345	346	347	348	349	350	351	352	353	354	355	356	357	358	359	360	361	362	363	364	365	366	367	368	369	370	371	372	373	374	375	376	377	378
1	*Centranthus calcitrapae* CC25	A	A	T	A	A	T	T	-	-	-	-	-	-	-	-	-	-	-	-	-	-	-	-	-	-	A	G	T	A	G	A	A	A	T	A	T	G	A	A	A	T	C	-	-	-	-	-	-	-	T	C	T	A	T	A	-	-	-	-	-	-	-	-
2	*Centranthus calcitrapae* CCACC539	.	.	.	.	.	.	.	-	-	-	-	-	-	-	-	-	-	-	-	-	-	-	-	-	-	.	.	.	.	.	.	.	.	.	.	.	.	.	.	.	.	.	-	-	-	-	-	-	-	.	.	.	.	.	.	-	-	-	-	-	-	-	-
3	*Fedia cornucopiae* FC73	.	.	.	.	.	.	.	A	G	T	A	A	A	G	T	T	G	A	A	A	T	A	A	T	T	.	.	.	.	.	.	.	.	W	.	.	.	.	.	.	.	Y	T	A	T	A	T	T	T	.	.	.	.	.	.	T	Y	T	A	A	C	T	A
4	*Valerianella carinata* VCA0104	.	.	.	.	.	.	.	-	-	-	-	-	-	-	-	-	-	-	-	-	-	-	-	-	-	.	.	.	.	.	.	.	.	.	.	.	.	.	.	.	.	.	T	C	T	A	T	C	T	.	.	.	.	.	.	T	C	-	-	-	-	-	-
5	*Valerianella carinata* VCA0202	.	.	.	.	.	.	.	-	-	-	-	-	-	-	-	-	-	-	-	-	-	-	-	-	-	.	.	.	.	.	.	.	.	.	.	G	.	.	.	.	.	.	T	C	T	A	T	T	T	.	.	.	.	.	.	T	C	-	-	-	-	-	-
6	*Valerianella carinata* VCA0302	.	.	.	.	.	.	.	-	-	-	-	-	-	-	-	-	-	-	-	-	-	-	-	-	-	.	.	.	.	.	.	.	.	.	.	.	.	.	.	.	.	.	T	C	T	T	T	T	T	.	.	.	.	.	.	T	C	-	-	-	-	-	-
7	*Valerianella carinata* VCA0501	.	.	.	.	.	.	.	-	-	-	-	-	-	-	-	-	-	-	-	-	-	-	-	-	-	.	.	.	.	.	.	.	.	.	.	G	.	.	.	.	.	.	T	C	T	A	T	T	T	.	.	.	.	.	.	T	C	-	-	-	-	-	-
8	*Valerianella carinata* VCA0805	.	.	.	.	.	.	.	-	-	-	-	-	-	-	-	-	-	-	-	-	-	-	-	-	-	.	.	.	.	.	.	.	.	.	.	G	.	.	.	.	.	.	T	C	T	A	T	T	T	.	.	.	.	.	.	T	C	-	-	-	-	-	-
9	*Valerianella carinata* VCA0918	.	.	.	.	.	.	.	-	-	-	-	-	-	-	-	-	-	-	-	-	-	-	-	-	-	.	.	.	.	.	.	.	.	.	.	G	.	.	.	.	.	.	T	C	T	A	T	T	T	.	.	.	.	.	.	T	C	-	-	-	-	-	-
10	*Valerianella carinata* VCA1006	.	.	.	.	.	.	.	-	-	-	-	-	-	-	-	-	-	-	-	-	-	-	-	-	-	.	.	.	.	.	.	.	.	.	.	G	.	.	.	.	.	.	T	C	T	A	T	T	T	.	.	.	.	.	.	T	C	-	-	-	-	-	-
11	*Valerianella carinata* VCA1106	.	.	.	.	.	.	.	-	-	-	-	-	-	-	-	-	-	-	-	-	-	-	-	-	-	.	.	.	.	.	.	.	.	.	.	G	.	.	.	.	.	.	T	C	T	A	T	T	T	.	.	.	.	.	.	T	C	-	-	-	-	-	-
12	*Valerianella carinata* VCA1202	.	.	.	.	.	.	.	-	-	-	-	-	-	-	-	-	-	-	-	-	-	-	-	-	-	.	.	.	.	.	.	.	.	.	.	G	.	.	.	.	.	.	T	C	T	A	T	T	T	.	.	.	.	.	.	T	C	-	-	-	-	-	-
13	*Valerianella coronata* VCO0102	.	.	.	.	.	.	.	-	-	-	-	-	-	-	-	-	-	-	-	-	-	-	-	-	-	C	.	.	.	T	.	.	.	.	.	.	.	.	.	.	.	.	-	-	-	-	-	-	-	.	.	.	.	.	.	T	C	-	-	-	-	-	-
14	*Valerianella coronata* VCO0218	.	.	.	.	.	.	.	-	-	-	-	-	-	-	-	-	-	-	-	-	-	-	-	-	-	C	.	.	.	T	.	.	.	.	.	.	.	.	.	.	.	.	-	-	-	-	-	-	-	.	.	.	.	.	.	T	C	-	-	-	-	-	-
15	*Valerianella coronata* VCO0404	.	.	.	.	.	.	.	-	-	-	-	-	-	-	-	-	-	-	-	-	-	-	-	-	-	C	.	.	.	T	.	.	.	.	.	.	.	.	.	.	.	.	-	-	-	-	-	-	-	.	.	.	.	.	.	T	C	-	-	-	-	-	-
16	*Valerianella coronata* VCO0508	.	.	.	.	.	.	.	-	-	-	-	-	-	-	-	-	-	-	-	-	-	-	-	-	-	C	.	.	.	T	.	.	.	.	.	.	.	.	.	.	.	.	-	-	-	-	-	-	-	.	.	.	.	.	.	T	C	-	-	-	-	-	-
17	*Valerianella coronata* VCO0705	.	.	.	.	.	.	.	-	-	-	-	-	-	-	-	-	-	-	-	-	-	-	-	-	-	C	.	.	.	T	.	.	.	.	.	.	.	.	.	.	.	.	-	-	-	-	-	-	-	.	.	.	.	.	.	T	C	-	-	-	-	-	-
18	*Valerianella coronata* VCO0805	.	.	.	.	.	.	.	-	-	-	-	-	-	-	-	-	-	-	-	-	-	-	-	-	-	C	.	.	.	T	.	.	.	.	.	.	.	.	.	.	.	.	-	-	-	-	-	-	-	.	.	.	.	.	.	T	C	-	-	-	-	-	-
19	*Valerianella coronata* VCO0901	.	.	.	.	.	.	.	-	-	-	-	-	-	-	-	-	-	-	-	-	-	-	-	-	-	C	.	.	.	T	.	.	.	.	.	.	.	.	.	.	.	.	-	-	-	-	-	-	-	.	.	.	.	.	.	T	C	-	-	-	-	-	-
20	*Valerianella dentata* VDE0303	.	.	.	.	.	.	.	-	-	-	-	-	-	-	-	-	-	-	-	-	-	-	-	-	-	.	.	.	.	T	.	.	.	.	.	.	.	.	.	.	.	.	-	-	-	-	-	-	-	.	.	.	.	.	.	T	C	-	-	-	-	-	-
21	*Valerianella discoidea* VDI0202	.	.	.	.	.	.	.	-	-	-	-	-	-	-	-	-	-	-	-	-	-	-	-	-	-	.	.	.	.	T	.	.	.	.	.	.	.	.	.	.	.	.	T	A	-	-	-	-	-	.	.	.	.	.	.	T	C	-	-	-	-	-	-
22	*Valerianella discoidea* VDI0412	.	.	.	.	.	.	.	-	-	-	-	-	-	-	-	-	-	-	-	-	-	-	-	-	-	.	.	.	.	T	.	.	.	.	.	.	.	.	.	.	.	.	T	A	-	-	-	-	-	.	.	.	.	.	.	T	C	-	-	-	-	-	-
23	*Valerianella echinata* VEC0101	.	.	.	.	.	.	.	-	-	-	-	-	-	-	-	-	-	-	-	-	-	-	-	-	T	.	.	.	.	.	.	.	.	.	.	G	A	.	.	.	.	.	T	A	T	A	T	C	T	.	.	.	.	.	.	T	T	-	-	-	-	-	-
24	*Valerianella eriocarpa* VER0208	.	.	.	.	.	.	C	-	-	-	-	-	-	-	-	-	-	-	-	-	-	-	-	-	-	.	.	.	.	T	.	.	.	.	.	.	.	.	.	.	.	.	-	-	-	-	-	-	-	.	.	.	.	.	.	T	C	-	-	-	-	-	-
25	*Valerianella eriocarpa* VER0402	.	.	.	.	.	.	C	-	-	-	-	-	-	-	-	-	-	-	-	-	-	-	-	-	-	.	.	.	.	T	.	.	.	.	.	.	.	.	.	.	.	.	-	-	-	-	-	-	-	.	.	.	.	.	.	T	C	-	-	-	-	-	-
26	*Valerianella fusiformis* VFU0103	.	.	.	.	.	.	.	-	-	-	-	-	-	-	-	-	-	-	-	-	-	-	-	-	-	.	.	.	.	.	.	.	.	.	.	.	.	.	.	.	.	.	T	A	G	A	T	C	T	.	.	.	.	.	.	T	C	-	-	-	-	-	-
27	*Valerianella locusta* VLO0103	.	.	.	.	.	.	.	-	-	-	-	-	-	-	-	-	-	-	-	-	-	-	-	-	-	.	.	.	.	.	.	.	.	.	.	G	.	.	.	.	.	.	T	C	T	A	T	T	T	.	.	.	.	.	.	T	C	-	-	-	-	-	-
28	*Valerianella locusta* VLO0305	.	.	.	.	.	.	.	-	-	-	-	-	-	-	-	-	-	-	-	-	-	-	-	-	-	.	.	.	.	.	.	.	.	.	.	G	.	.	.	.	.	.	T	C	T	A	T	T	T	.	.	.	.	.	.	T	C	-	-	-	-	-	-
29	*Valerianella locusta* VLO0409	.	.	.	.	.	.	.	-	-	-	-	-	-	-	-	-	-	-	-	-	-	-	-	-	-	.	.	.	.	.	.	.	.	.	.	G	.	.	.	.	.	.	T	C	T	A	T	T	T	.	.	.	.	.	.	T	C	-	-	-	-	-	-
30	*Valerianella locusta* VLO0505	.	.	.	.	.	.	.	-	-	-	-	-	-	-	-	-	-	-	-	-	-	-	-	-	-	.	.	.	.	.	.	.	.	.	.	G	.	.	.	.	.	.	T	C	T	A	T	T	T	.	.	.	.	.	.	T	C	-	-	-	-	-	-
31	*Valerianella locusta* VLO0609	.	.	.	.	.	.	.	-	-	-	-	-	-	-	-	-	-	-	-	-	-	-	-	-	-	.	.	.	.	.	.	.	.	.	.	G	.	.	.	.	.	.	T	C	T	A	T	T	T	.	.	.	.	.	.	T	C	-	-	-	-	-	-
32	*Valerianella lusitanica* VLU0102	.	.	.	.	.	.	.	-	-	-	-	-	-	-	-	-	-	-	-	-	-	-	-	-	-	.	.	.	.	.	.	.	.	.	.	.	.	.	.	.	.	.	T	C	T	A	T	C	T	.	.	.	.	.	.	T	C	-	-	-	-	-	-
33	*Valerianella microcarpa* VMI0102	.	.	.	.	.	.	C	-	-	-	-	-	-	-	-	-	-	-	-	-	-	-	-	-	-	.	.	.	.	T	.	.	.	.	.	.	.	.	.	.	.	.	-	-	-	-	-	-	-	.	.	.	.	.	.	T	C	-	-	-	-	-	-
34	*Valerianella multidentata* VMU0201	.	.	.	.	.	.	.	-	-	-	-	-	-	-	-	-	-	-	-	-	-	-	-	-	-	.	.	.	.	T	.	.	.	.	.	.	.	.	.	.	.	.	T	A	-	-	-	-	-	.	.	.	.	.	.	T	C	-	-	-	-	-	-
35	*Valerianella multidentata* VMU0301	.	.	.	.	.	.	.	-	-	-	-	-	-	-	-	-	-	-	-	-	-	-	-	-	-	.	.	.	.	T	.	.	.	.	.	.	.	.	.	.	.	.	T	A	-	-	-	-	-	.	.	.	.	.	.	T	C	-	-	-	-	-	-
36	*Valerianella pumila* VPU0103	.	.	.	.	.	.	.	-	-	-	-	-	-	-	-	-	-	-	-	-	-	-	-	-	-	C	.	.	.	T	.	.	.	.	.	.	.	.	.	.	.	.	-	-	-	-	-	-	-	.	.	.	.	.	.	T	C	-	-	-	-	-	-
37	*Valerianella pumila* VPU0304	.	.	.	.	.	.	.	-	-	-	-	-	-	-	-	-	-	-	-	-	-	-	-	-	-	C	.	.	.	T	.	.	.	.	.	.	.	.	.	.	.	.	-	-	-	-	-	-	-	.	.	.	.	.	.	T	C	-	-	-	-	-	-
38	*Valerianella pumila* VPU0402	.	.	.	.	.	.	.	-	-	-	-	-	-	-	-	-	-	-	-	-	-	-	-	-	-	C	.	.	.	T	.	.	.	.	.	.	.	.	.	.	.	.	-	-	-	-	-	-	-	.	.	.	.	.	.	T	C	-	-	-	-	-	-
39	*Valerianella rimosa* VRI0101	.	.	.	.	.	.	.	-	-	-	-	-	-	-	-	-	-	-	-	-	-	-	-	-	-	.	.	.	.	T	.	.	.	.	.	.	.	.	.	.	.	.	-	-	-	-	-	-	-	.	.	.	.	.	.	T	C	-	-	-	-	-	-
40	*Valerianella rimosa* VRI0201	.	.	.	.	.	.	.	-	-	-	-	-	-	-	-	-	-	-	-	-	-	-	-	-	-	.	.	.	.	T	.	.	.	.	.	.	.	.	.	.	.	.	-	-	-	-	-	-	-	.	.	.	.	.	.	T	C	-	-	-	-	-	-

No.	Taxon	Position
		3 3 3 3 3 3 3 3 3 3 3 3 3 3 3 3 3 3 3 3 3 4 4 4 4 4 4 4 4 4 4 4 4 4 4 4 4 4 4 4 4 4 4 4 4 4 4 4 4 4 4 4 4 4 4 4 4 4 4 4 4 4 4
		7 8 8 8 8 8 8 8 8 8 8 9 9 9 9 9 9 9 9 9 9 0 0 0 0 0 0 0 0 0 0 1 1 1 1 1 1 1 1 1 1 2 2 2 2 2 2 2 2 2 2 3 3 3 3 3 3 3 3 3 3 4 4
		9 0 1 2 3 4 5 6 7 8 9 0 1 2 3 4 5 6 7 8 9 0 1 2 3 4 5 6 7 8 9 0 1 2 3 4 5 6 7 8 9 0 1 2 3 4 5 6 7 8 9 0 1 2 3 4 5 6 7 8 9 0 1
1	*Centranthus calcitrapae* CC25	- - - - G A A A T A G A A T A A C A T A A G A T T C T G G A C T A A A A A A G A A A - - - - - - - - - - - - - - - - - - A G T
2	*Centranthus calcitrapae* CCACC539	- - - - . . . . - - - - - - - - - - - - - - - - - - . . .
3	*Fedia cornucopiae* FC73	T A T Y T . . G . . . . . W . . M M . . . . Y . . T W . . . . R . . . - - - - - - - - - - - - - - - - - A . A -
4	*Valerianella carinata* VCA0104	- - - - T . . G . . . . G . . . . T . . . . - - - - - - - - - - - - - - - - A A . A .
5	*Valerianella carinata* VCA0202	- - - - T . . G . . . . G T . . . . T . . . . - - - - - - - - - - - - - - - - A A . A .
6	*Valerianella carinata* VCA0302	- - - - T . . G . . . . G T . . . . T . . . . - - - - - - - - - - - - - - - - A A . A .
7	*Valerianella carinata* VCA0501	- - - - T . . G . . . . G T . . . . T . . . . - - - - - - - - - - - - - - - - A A . A .
8	*Valerianella carinata* VCA0805	- - - - T . . G . . . . G T . . . . T . . . . - - - - - - - - - - - - - - - - A A . A .
9	*Valerianella carinata* VCA0918	- - - - T . . G . . . . G T . . . . T . . . . - - - - - - - - - - - - - - - - A A . A .
10	*Valerianella carinata* VCA1006	- - - - T . . G . . . . G T . . . . T . . . . - - - - - - - - - - - - - - - - A A . A .
11	*Valerianella carinata* VCA1106	- - - - T . . G . . . . G T . . . . T . . . . - - - - - - - - - - - - - - - - A A . A .
12	*Valerianella carinata* VCA1202	- - - - T . . G . . . . G T . . . . T . . . . - - - - - - - - - - - - - - - - A A . A .
13	*Valerianella coronata* VCO0102	- - - - T . . G . . . . C . . . G . . . . T . . . . - - - - - - - - - - - - - - - - - - . A .
14	*Valerianella coronata* VCO0218	- - - - T . . G . . . . C . . . G . . . . T . . . . - - - - - - - - - - - - - - - - - - . A .
15	*Valerianella coronata* VCO0404	- - - - T . . G . . . . C . . . G . . . . T . . . . - - - - - - - - - - - - - - - - - - . A .
16	*Valerianella coronata* VCO0508	- - - - T . . G . . . . C . . . G . . . . T . . . . - - - - - - - - - - - - - - - - - - . A .
17	*Valerianella coronata* VCO0705	- - - - T . . G . . . . C . . . G . . . . T . . . . - - - - - - - - - - - - - - - - - - . A .
18	*Valerianella coronata* VCO0805	- - - - T . . G . . . . C . . . G . . . . T . . . . - - - - - - - - - - - - - - - - - - . A .
19	*Valerianella coronata* VCO0901	- - - - T . . G . . . . C . . . G . . . . T . . . . - - - - - - - - - - - - - - - - - - . A .
20	*Valerianella dentata* VDE0303	- - - - T . . G . . . . C . . . G . . . . T . . . . C . . - - - - - - - - - - - - - - - - - - . A .
21	*Valerianella discoidea* VDI0202	- - - - T . . G . . . . C . . . G . . . . T . . . . - - - - - - - - - - - - - - - - - - . A .
22	*Valerianella discoidea* VDI0412	- - - - T . . G . . . . C . . . G . . . . T . . . . - - - - - - - - - - - - - - - - - - . A .
23	*Valerianella echinata* VEC0101	- - - - T . . G . . . . T . . . . - - - - - - - - - - - - - - - - - - . A .
24	*Valerianella eriocarpa* VER0208	- - - - T . . G . . . . C . . . G . . . . T . . . . C . . - - - - - - - - - - - - - - - - - - . A .
25	*Valerianella eriocarpa* VER0402	- - - - T . . G . . . . C . . . G . . . . T . . . . C . . - - - - - - - - - - - - - - - - - - . A .
26	*Valerianella fusiformis* VFU0103	- - - - T . . G . . . . C . . . . T . . . T . . . . T C T G T A C T T A A A A A G A A A . A .
27	*Valerianella locusta* VLO0103	- - - - T . . G . . . . G T . . . . T . . . . - - - - - - - - - - - - - - - - A A . A .
28	*Valerianella locusta* VLO0305	- - - - T . . G . . . . G T . . . . T . . . . - - - - - - - - - - - - - - - - A A . A .
29	*Valerianella locusta* VLO0409	- - - - T . . G . . . . G T . . . . T . . . . - - - - - - - - - - - - - - - - A A . A .
30	*Valerianella locusta* VLO0505	- - - - T . . G . . . . G T . . . . T . . . . - - - - - - - - - - - - - - - - A A . A .
31	*Valerianella locusta* VLO0609	- - - - T . . G . . . . G T . . . . T . . . . - - - - - - - - - - - - - - - - A A . A .
32	*Valerianella lusitanica* VLU0102	- - - - T . . G . . . . G . . . . T . . . . - - - - - - - - - - - - - - - - A A . A .
33	*Valerianella microcarpa* VMI0102	- - - - T . . G . . . . C . . . G . . . . T . . . . C . . - - - - - - - - - - - - - - - - - - . A .
34	*Valerianella multidentata* VMU0201	- - - - T . . G . . . . C . . . G . . . . T . . . . - - - - - - - - - - - - - - - - - - . A .
35	*Valerianella multidentata* VMU0301	- - - - T . . G . . . . C . . . G . . . . T . . . . - - - - - - - - - - - - - - - - - - . A .
36	*Valerianella pumila* VPU0103	- - - - T . . G . . . . C . . . G . . . . T . . . . - - - - - - - - - - - - - - - - - - . A .
37	*Valerianella pumila* VPU0304	- - - - T . . G . . . . C . . . G . . . . T . . . . - - - - - - - - - - - - - - - - - - . A .
38	*Valerianella pumila* VPU0402	- - - - T . . G . . . . C . . . G . . . . T . . . . - - - - - - - - - - - - - - - - - - . A .
39	*Valerianella rimosa* VRI0101	- - - - T . . G . . . . C . . . G . . . . T . . . . C . . - - - - - - - - - - - - - - - - - - . A .
40	*Valerianella rimosa* VRI0201	- - - - T . . G . . . . C . . . G . . . . T . . . . C . . - - - - - - - - - - - - - - - - - - . A .

Position

No.	Taxon	442	443	444	445	446	447	448	449	450	451	452	453	454	455	456	457	458	459	460	461	462	463	464	465	466	467	468	469	470	471	472	473	474	475	476	477	478	479	480	481	482	483	484	485	486	487	488	489	490	491	492	493	494	495	496	497	498	499	500	501	502	503	504
1	*Centranthus calcitrapae* CC25	G	T	A	A	G	A	A	A	A	A	A	T	T	T	T	C	A	A	A	T	G	A	C	A	T	T	T	G	A	A	C	T	C	A	A	A	A	A	A	A	A	A	-	-	-	-	-	-	-	G	A	T	T	G	C	A	T	C	T	C	A	A	A
2	*Centranthus calcitrapae* CCACC539	.	.	.	.	.	.	.	.	.	.	.	.	.	.	.	.	.	.	.	.	.	.	.	.	.	.	.	.	.	.	.	.	.	.	.	.	.	.	.	.	.	-	-	-	-	-	-	-	-	.	.	.	.	.	.	.	.	.	.	.	.	.	.
3	*Fedia cornucopiae* FC73	.	.	.	.	T	.	.	.	.	.	.	.	.	.	.	.	.	.	.	C	.	.	A	.	.	.	.	.	.	.	.	G	.	.	.	.	.	.	.	.	.	.	A	A	A	-	-	-	-	.	.	.	.	.	.	.	.	.	.	.	.	G	.
4	*Valerianella carinata* VCA0104	.	.	.	.	T	.	.	.	.	.	.	.	.	.	.	.	.	.	.	.	.	.	A	.	.	.	.	.	.	.	.	G	.	.	.	.	.	.	.	.	.	.	A	A	A	-	-	-	C	.	.	.	.	.	.	.	.	.	.	.	.	.	.
5	*Valerianella carinata* VCA0202	.	.	.	.	T	.	.	.	.	.	.	.	.	.	.	.	.	.	.	.	.	.	A	.	.	.	.	.	.	.	.	G	.	.	.	.	.	.	.	.	.	.	A	A	-	-	-	-	C	.	.	.	.	.	.	.	.	.	.	.	.	.	.
6	*Valerianella carinata* VCA0302	.	.	.	.	T	.	.	.	.	.	.	.	.	.	.	.	.	.	.	.	.	.	A	.	.	.	.	.	.	.	.	G	.	.	.	.	.	.	.	.	.	.	A	A	A	-	-	-	C	.	.	.	.	.	.	.	.	.	.	.	.	.	.
7	*Valerianella carinata* VCA0501	.	.	.	.	T	.	.	.	.	.	.	.	.	.	.	.	.	.	.	.	.	.	A	.	.	.	.	.	.	.	.	G	.	.	.	.	.	.	.	.	.	.	A	-	-	-	-	-	C	.	.	.	.	.	.	.	.	.	.	.	.	.	.
8	*Valerianella carinata* VCA0805	.	.	.	.	T	.	.	.	.	.	.	.	.	.	.	.	.	.	.	.	.	.	A	.	.	.	.	.	.	.	.	G	.	.	.	.	.	.	.	.	.	.	A	A	-	-	-	-	C	.	.	.	.	.	.	.	.	.	.	.	.	.	.
9	*Valerianella carinata* VCA0918	.	.	.	.	T	.	.	.	.	.	.	.	.	.	.	.	.	.	.	.	.	.	A	.	.	.	.	.	.	.	.	G	.	.	.	.	.	.	.	.	.	.	A	A	-	-	-	-	C	.	.	.	.	.	.	.	.	.	.	.	.	.	.
10	*Valerianella carinata* VCA1006	.	.	.	.	T	.	.	.	.	.	.	.	.	.	.	.	.	.	.	.	.	.	A	.	.	.	.	.	.	.	.	G	.	.	.	.	.	.	.	.	.	.	A	-	-	-	-	-	C	.	.	.	.	.	.	.	.	.	.	.	.	.	.
11	*Valerianella carinata* VCA1106	.	.	.	.	T	.	.	.	.	.	.	.	.	.	.	.	.	.	.	.	.	.	A	.	.	.	.	.	.	.	.	G	.	.	.	.	.	.	.	.	.	.	A	-	-	-	-	-	C	.	.	.	.	.	.	.	.	.	.	.	.	.	.
12	*Valerianella carinata* VCA1202	.	.	.	.	T	.	.	.	.	.	.	.	.	.	.	.	.	.	.	.	.	.	A	.	.	.	.	.	.	.	.	G	.	.	.	.	.	.	.	.	.	.	A	-	-	-	-	-	C	.	.	.	.	.	.	.	.	.	.	.	.	.	.
13	*Valerianella coronata* VCO0102	.	.	.	.	T	C	.	.	.	.	.	.	.	.	.	.	.	.	.	.	T	C	.	.	.	.	.	.	.	.	.	G	.	.	.	.	.	.	.	.	.	.	-	-	-	-	-	-	-	.	.	.	.	.	.	.	.	.	.	.	.	.	.
14	*Valerianella coronata* VCO0218	.	.	.	.	T	C	.	.	.	.	.	.	.	.	.	.	.	.	.	.	T	C	.	.	.	.	.	.	.	.	.	G	.	.	.	.	.	.	.	.	.	.	-	-	-	-	-	-	-	.	.	.	.	.	.	.	.	.	.	.	.	.	.
15	*Valerianella coronata* VCO0404	.	.	.	.	T	C	.	.	.	.	.	.	.	.	.	.	.	.	.	.	T	C	.	.	.	.	.	.	.	.	.	G	.	.	.	.	.	.	.	.	.	.	-	-	-	-	-	-	-	.	.	.	.	.	.	.	.	.	.	.	.	.	.
16	*Valerianella coronata* VCO0508	.	.	.	.	T	C	.	.	.	.	.	.	.	.	.	.	.	.	.	.	T	C	.	.	.	.	.	.	.	.	.	G	.	.	.	.	.	.	.	.	.	.	-	-	-	-	-	-	-	.	.	.	.	.	.	.	.	.	.	.	.	.	.
17	*Valerianella coronata* VCO0705	.	.	.	.	T	C	.	.	.	.	.	.	.	.	.	.	.	.	.	.	T	C	.	.	.	.	.	.	.	.	.	G	.	.	.	.	.	.	.	.	.	.	-	-	-	-	-	-	-	.	.	.	.	.	.	.	.	.	.	.	.	.	.
18	*Valerianella coronata* VCO0805	.	.	.	.	T	C	.	.	.	.	.	.	.	.	.	.	.	.	.	.	T	C	.	.	.	.	.	.	.	.	.	G	.	.	.	.	.	.	.	.	.	.	-	-	-	-	-	-	-	.	.	.	.	.	.	.	.	.	.	.	.	.	.
19	*Valerianella coronata* VCO0901	.	.	.	.	T	C	.	.	.	.	.	.	.	.	.	.	.	.	.	.	T	C	.	.	.	.	.	.	.	.	.	G	.	.	.	.	.	.	.	.	.	.	-	-	-	-	-	-	-	.	.	.	.	.	.	.	.	.	.	.	.	.	.
20	*Valerianella dentata* VDE0303	.	.	.	.	T	C	.	.	.	.	.	.	.	.	.	.	.	.	.	.	.	G	A	.	.	.	.	.	.	.	.	G	.	.	.	.	.	.	.	.	-	-	-	-	-	-	-	-	-	.	.	.	.	.	.	.	.	.	.	.	.	.	.
21	*Valerianella discoidea* VDI0202	.	.	.	.	T	C	.	.	.	.	.	.	.	.	.	.	.	.	.	.	.	G	A	.	.	.	.	.	.	.	.	G	.	.	.	.	.	.	.	.	.	.	-	-	-	-	-	-	-	.	.	.	.	.	.	.	.	.	.	.	.	.	.
22	*Valerianella discoidea* VDI0412	.	.	.	.	T	C	.	.	.	.	.	.	.	.	.	.	.	.	.	.	.	G	A	.	.	.	.	.	.	.	.	G	.	.	.	.	.	.	.	.	.	.	-	-	-	-	-	-	-	.	.	.	.	.	.	.	.	.	.	.	.	.	.
23	*Valerianella echinata* VEC0101	.	C	.	.	T	.	.	.	.	.	.	.	.	.	.	.	.	.	.	.	.	.	G	.	G	.	.	.	.	.	.	G	.	.	.	.	.	.	.	.	.	.	A	A	A	A	-	-	-	.	.	.	.	.	.	.	.	.	.	.	.	.	.
24	*Valerianella eriocarpa* VER0208	.	.	.	.	T	C	.	.	.	.	.	.	.	.	.	.	.	.	.	.	.	G	A	.	.	.	.	.	.	.	.	G	.	.	.	.	.	.	.	.	.	.	A	-	-	-	-	-	-	.	.	.	.	.	.	.	.	.	.	.	.	.	.
25	*Valerianella eriocarpa* VER0402	.	.	.	.	T	C	.	.	.	.	.	.	.	.	.	.	.	.	.	.	.	G	A	.	.	.	.	.	.	.	.	G	.	.	.	.	.	.	.	.	.	.	A	-	-	-	-	-	-	.	.	.	.	.	.	.	.	.	.	.	.	.	.
26	*Valerianella fusiformis* VFU0103	.	.	.	.	T	C	.	.	.	.	.	.	.	.	.	.	.	.	.	.	.	.	A	.	.	.	.	.	.	.	.	A	.	.	.	.	.	.	.	.	.	-	-	-	-	-	-	-	-	.	.	.	.	T	G	.	.	T	.	A	.	.	.
27	*Valerianella locusta* VLO0103	.	.	.	.	T	.	.	.	.	.	.	.	.	.	.	.	.	.	.	.	.	.	A	.	.	.	.	.	.	.	.	G	.	.	.	.	.	.	.	.	.	.	A	-	-	-	-	-	C	.	.	.	.	.	.	.	.	.	.	.	.	.	.
28	*Valerianella locusta* VLO0305	.	.	.	.	T	.	.	.	.	.	.	.	.	.	.	.	.	.	.	.	.	.	A	.	.	.	.	.	.	.	.	G	.	.	.	.	.	.	.	.	.	.	A	-	-	-	-	-	C	.	.	.	.	.	.	.	.	.	.	.	.	.	.
29	*Valerianella locusta* VLO0409	.	.	.	.	T	.	.	.	.	.	.	.	.	.	.	.	.	.	.	.	.	.	A	.	.	.	.	.	.	.	.	G	.	.	.	.	.	.	.	.	.	.	A	-	-	-	-	-	C	.	.	.	.	.	.	.	.	.	.	.	.	.	.
30	*Valerianella locusta* VLO0505	.	.	.	.	T	.	.	.	.	.	.	.	.	.	.	.	.	.	.	.	.	.	A	.	.	.	.	.	.	.	.	G	.	.	.	.	.	.	.	.	.	.	A	A	-	-	-	-	C	.	.	.	.	.	.	.	.	.	.	.	.	.	.
31	*Valerianella locusta* VLO0609	.	.	.	.	T	.	.	.	.	.	.	.	.	.	.	.	.	.	.	.	.	.	A	.	.	.	.	.	.	.	.	G	.	.	.	.	.	.	.	.	.	.	A	A	-	-	-	-	C	.	.	.	.	.	.	.	.	.	.	.	.	.	.
32	*Valerianella lusitanica* VLU0102	.	.	.	.	T	.	.	.	.	.	.	.	.	.	.	.	.	.	.	.	.	.	A	.	.	.	.	.	.	.	.	G	.	.	.	.	.	.	.	.	.	.	A	A	A	A	A	A	C	.	.	.	.	.	.	.	.	.	.	.	.	.	.
33	*Valerianella microcarpa* VMI0102	.	.	.	.	T	C	.	.	.	.	.	.	.	.	.	.	.	.	.	.	.	G	A	.	.	.	.	.	.	.	.	G	.	.	.	.	.	.	.	.	.	-	-	-	-	-	-	-	-	.	.	.	.	.	.	.	.	.	.	.	.	.	.
34	*Valerianella multidentata* VMU0201	.	.	.	.	T	C	.	.	.	.	.	.	.	.	.	.	.	.	.	.	.	G	A	.	.	.	.	.	.	.	.	G	.	.	.	.	.	.	.	.	.	.	-	-	-	-	-	-	-	.	.	.	.	.	.	.	.	.	.	.	.	.	.
35	*Valerianella multidentata* VMU0301	.	.	.	.	T	C	.	.	.	.	.	.	.	.	.	.	.	.	.	.	.	G	A	.	.	.	.	.	.	.	.	G	.	.	.	.	.	.	.	.	.	.	-	-	-	-	-	-	-	.	.	.	.	.	.	.	.	.	.	.	.	.	.
36	*Valerianella pumila* VPU0103	.	.	.	.	T	C	.	.	.	.	.	.	.	.	.	.	.	.	.	.	T	C	.	.	.	.	.	.	.	.	.	G	.	.	.	.	.	.	.	.	.	.	-	-	-	-	-	-	-	.	.	.	.	.	.	.	.	.	.	.	.	.	.
37	*Valerianella pumila* VPU0304	.	.	.	.	T	C	.	.	.	.	.	.	.	.	.	.	.	.	.	.	T	C	.	.	.	.	.	.	.	.	.	G	.	.	.	.	.	.	.	.	.	.	-	-	-	-	-	-	-	.	.	.	.	.	.	.	.	.	.	.	.	.	.
38	*Valerianella pumila* VPU0402	.	.	.	.	T	C	.	.	.	.	.	.	.	.	.	.	.	.	.	.	T	C	.	.	.	.	.	.	.	.	.	G	.	.	.	.	.	.	.	.	.	.	A	-	-	-	-	-	-	.	.	.	.	.	.	.	.	.	.	.	.	.	.
39	*Valerianella rimosa* VRI0101	.	.	.	.	T	C	.	.	.	.	.	.	.	.	.	.	.	.	.	.	.	G	A	.	.	.	.	.	.	.	.	G	.	.	.	.	.	.	.	.	-	-	-	-	-	-	-	-	-	.	.	.	.	.	.	.	.	.	.	.	.	.	.
40	*Valerianella rimosa* VRI0201	.	.	.	.	T	C	.	.	.	.	.	.	.	.	.	.	.	.	.	.	.	G	A	.	.	.	.	.	.	.	.	G	.	.	.	.	.	.	.	.	-	-	-	-	-	-	-	-	-	.	.	.	.	.	.	.	.	.	.	.	.	.	.

	Position	505	506	507	508	509	510	511	512	513	514	515	516	517	518	519	520	521	522	523	524	525	526	527	528	529	530	531	532	533	534	535	536	537	538	539	540	541	542	543	544	545	546	547	548	549	550	551	552	553	554	555	556	557	558	559	560	561	562	563	564	565	566	567
1	*Centranthus calcitrapae* CC25	A	T	T	A	T	T	A	C	T	A	-	G	C	C	A	G	T	A	A	T	A	A	T	C	A	A	T	T	G	T	G	A	T	A	A	A	T	A	A	A	T	A	A	A	T	A	A	A	T	T	C	T	A	T	A	T	T	C	T	C	T	G	-
2	*Centranthus calcitrapae* CCACC539	.	.	.	.	.	.	.	.	.	.	-	.	.	.	.	.	.	.	.	.	.	.	.	.	.	.	.	.	.	.	.	.	.	.	.	.	.	.	.	.	.	.	.	.	.	.	.	.	.	.	.	.	.	.	.	.	.	.	.	.	.	.	-
3	*Fedia cornucopiae* FC73	.	.	.	.	.	.	C	.	.	.	-	.	.	.	.	.	.	.	.	.	.	.	.	T	.	.	.	.	.	.	.	.	A	.	.	.	.	.	.	.	.	.	G	.	A	.	-	-	.	.	.	.	.	-	-	-	-	-	-	-	.	.	-
4	*Valerianella carinata* VCA0104	.	.	.	.	.	.	C	.	.	.	-	.	.	.	.	.	.	.	.	.	.	.	.	.	.	.	.	.	A	.	.	.	A	.	.	.	.	.	.	.	.	.	G	.	A	.	-	-	.	.	.	.	.	G	.	.	.	.	.	A	.	.	-
5	*Valerianella carinata* VCA0202	.	.	.	.	.	.	C	.	.	.	-	.	.	.	.	.	.	.	.	.	.	.	.	.	.	.	.	.	A	.	.	.	A	.	.	.	.	.	.	.	.	.	G	.	A	.	-	-	.	.	.	.	.	G	.	.	.	.	.	A	.	.	-
6	*Valerianella carinata* VCA0302	.	.	.	.	.	.	C	.	.	.	-	.	.	.	.	.	.	.	.	.	.	.	.	.	.	.	.	.	A	.	.	.	A	.	.	.	.	.	.	.	.	.	G	.	A	.	-	-	.	.	.	.	.	G	.	.	.	.	.	A	.	.	-
7	*Valerianella carinata* VCA0501	.	.	.	.	.	.	C	.	.	.	-	.	.	.	.	.	.	.	.	.	.	.	.	.	.	.	.	.	A	.	.	.	A	.	.	.	.	.	.	.	.	.	G	.	A	.	-	-	.	.	.	.	.	G	.	.	.	.	.	A	.	.	-
8	*Valerianella carinata* VCA0805	.	.	.	.	.	.	C	.	.	.	-	.	.	.	.	.	.	.	.	.	.	.	.	.	.	.	.	.	A	.	.	.	A	.	.	.	.	.	.	.	.	.	G	.	A	.	-	-	.	.	.	.	.	G	.	.	.	.	.	A	.	.	-
9	*Valerianella carinata* VCA0918	.	.	.	.	.	.	C	.	.	.	-	.	.	.	.	.	.	.	.	.	.	.	.	.	.	.	.	.	A	.	.	.	A	.	.	.	.	.	.	.	.	.	G	.	A	.	-	-	.	.	.	.	.	G	.	.	.	.	.	A	.	.	-
10	*Valerianella carinata* VCA1006	.	.	.	.	.	.	C	.	.	.	-	.	.	.	.	.	.	.	.	.	.	.	.	.	.	.	.	.	A	.	.	.	A	.	.	.	.	.	.	.	.	.	G	.	A	.	-	-	.	.	.	.	.	G	.	.	.	.	.	A	.	.	-
11	*Valerianella carinata* VCA1106	.	.	.	.	.	.	C	.	.	.	-	.	.	.	.	.	.	.	.	.	.	.	.	.	.	.	.	.	A	.	.	.	A	.	.	.	.	.	.	.	.	.	G	.	A	.	-	-	.	.	.	.	.	G	.	.	.	.	.	A	.	.	-
12	*Valerianella carinata* VCA1202	.	.	.	.	.	.	C	.	.	.	-	.	.	.	.	.	.	.	.	.	.	.	.	.	.	.	.	.	A	.	.	.	A	.	.	.	.	.	.	.	.	.	G	.	A	.	-	-	.	.	.	.	.	G	.	.	.	.	.	A	.	.	-
13	*Valerianella coronata* VCO0102	.	.	.	.	.	.	C	.	G	.	A	.	.	.	.	.	.	.	.	.	.	.	.	.	.	C	.	.	.	.	.	G	A	.	.	.	.	.	.	.	.	.	G	.	A	.	-	-	.	.	.	.	.	G	.	.	.	.	.	A	.	.	-
14	*Valerianella coronata* VCO0218	.	.	.	.	.	.	C	.	G	.	A	.	.	.	.	.	.	.	.	.	.	.	.	.	.	C	.	.	.	.	.	G	A	.	.	.	.	.	.	.	.	.	G	.	A	.	-	-	.	.	.	.	.	G	.	.	.	.	.	A	.	.	-
15	*Valerianella coronata* VCO0404	.	.	.	.	.	.	C	.	G	.	A	.	.	.	.	.	.	.	.	.	.	.	.	.	.	C	.	.	.	.	.	G	A	.	.	.	.	.	.	.	.	.	G	.	A	.	-	-	.	.	.	.	.	G	.	.	.	.	.	A	.	.	-
16	*Valerianella coronata* VCO0508	.	.	.	.	.	.	C	.	G	.	A	.	.	.	.	.	.	.	.	.	.	.	.	.	.	C	.	.	.	.	.	G	A	.	.	.	.	.	.	.	.	.	G	.	A	.	-	-	.	.	.	.	.	G	.	.	.	.	.	A	.	.	-
17	*Valerianella coronata* VCO0705	.	.	.	.	.	.	C	.	G	.	A	.	.	.	.	.	.	.	.	.	.	.	.	.	.	C	.	.	.	.	.	G	A	.	.	.	.	.	.	.	.	.	G	.	A	.	-	-	.	.	.	.	.	G	.	.	.	.	.	A	.	.	-
18	*Valerianella coronata* VCO0805	.	.	.	.	.	.	C	.	G	.	A	.	.	.	.	.	.	.	.	.	.	.	.	.	.	C	.	.	.	.	.	G	A	.	.	.	.	.	.	.	.	.	G	.	A	.	-	-	.	.	.	.	.	G	.	.	.	.	.	A	.	.	-
19	*Valerianella coronata* VCO0901	.	.	.	.	.	.	C	.	G	.	A	.	.	.	.	.	.	.	.	.	.	.	.	.	.	C	.	.	.	.	.	G	A	.	.	.	.	.	.	.	.	.	G	.	A	.	-	-	.	.	.	.	.	G	.	.	.	.	.	A	.	.	-
20	*Valerianella dentata* VDE0303	.	.	.	.	.	.	C	.	.	.	-	.	.	.	.	.	G	.	.	.	.	.	.	.	.	.	.	.	.	.	.	G	A	.	.	.	.	.	.	.	.	.	G	.	A	.	-	-	.	.	.	.	.	G	.	.	.	.	.	A	.	.	-
21	*Valerianella discoidea* VDI0202	.	.	.	.	.	.	C	.	.	.	A	.	.	.	.	.	.	.	.	.	.	.	.	.	.	.	.	.	.	.	.	G	A	.	.	.	.	.	.	.	.	.	G	.	A	.	-	-	.	.	A	.	.	G	.	.	.	.	.	A	.	.	-
22	*Valerianella discoidea* VDI0412	.	.	.	.	.	.	C	.	.	.	A	.	.	.	.	.	.	.	.	.	.	.	.	.	.	.	.	.	.	.	.	G	A	.	.	.	.	.	.	.	.	.	G	.	A	.	-	-	.	.	A	.	.	G	.	.	.	.	.	A	.	.	-
23	*Valerianella echinata* VEC0101	.	.	.	.	.	.	T	.	.	.	-	.	.	.	.	.	.	.	.	.	.	.	.	.	.	.	.	.	.	.	.	.	A	.	.	.	.	.	.	.	.	.	G	.	A	.	-	-	.	.	.	.	.	G	.	.	.	.	.	A	.	.	-
24	*Valerianella eriocarpa* VER0208	.	.	.	.	.	.	C	.	.	.	-	.	.	.	.	.	G	.	.	.	.	.	.	.	.	.	.	.	T	.	.	G	A	.	.	.	.	.	.	.	.	.	G	.	A	.	-	-	.	.	.	.	.	G	.	.	.	.	.	A	.	.	-
25	*Valerianella eriocarpa* VER0402	.	.	.	.	.	.	C	.	.	.	-	.	.	.	.	.	G	.	.	.	.	.	.	.	.	.	.	.	T	.	.	G	A	.	.	.	.	.	.	.	.	.	G	.	A	.	-	-	.	.	.	.	.	G	.	.	.	.	.	A	.	.	-
26	*Valerianella fusiformis* VFU0103	.	A	.	.	.	.	C	.	.	.	-	.	.	.	.	.	.	.	.	.	.	.	.	.	.	.	.	C	.	C	.	.	A	.	.	.	.	.	.	.	.	.	.	.	A	.	-	-	.	.	.	.	.	G	.	.	.	.	.	A	.	.	C
27	*Valerianella locusta* VLO0103	.	.	.	.	.	.	C	.	.	.	-	.	.	.	.	.	.	.	.	.	.	.	.	.	.	.	.	.	A	.	.	.	A	.	.	.	.	.	.	.	.	.	G	.	A	.	-	-	.	.	.	.	.	G	.	.	.	.	.	A	.	.	-
28	*Valerianella locusta* VLO0305	.	.	.	.	.	.	C	.	.	.	-	.	.	.	.	.	.	.	.	.	.	.	.	.	.	.	.	.	A	.	.	.	A	.	.	.	.	.	.	.	.	.	G	.	A	.	-	-	.	.	.	.	.	G	.	.	.	.	.	A	.	.	-
29	*Valerianella locusta* VLO0409	.	.	.	.	.	.	C	.	.	.	-	.	.	.	.	.	.	.	.	.	.	.	.	.	.	.	.	.	A	.	.	.	A	.	.	.	.	.	.	.	.	.	G	.	A	.	-	-	.	.	.	.	.	G	.	.	.	.	.	A	.	.	-
30	*Valerianella locusta* VLO0505	.	.	.	.	.	.	C	.	.	.	-	.	.	.	.	.	.	.	.	.	.	.	.	.	.	.	.	.	A	.	.	.	A	.	.	.	.	.	.	.	.	.	G	.	A	.	-	-	.	.	.	.	.	G	.	.	.	.	.	A	.	.	-
31	*Valerianella locusta* VLO0609	.	.	.	.	.	.	C	.	.	.	-	.	.	.	.	.	.	.	.	.	.	.	.	.	.	.	.	.	A	.	.	.	A	.	.	.	.	.	.	.	.	.	G	.	A	.	-	-	.	.	.	.	.	G	.	.	.	.	.	A	.	.	-
32	*Valerianella lusitanica* VLU0102	.	.	.	.	.	.	C	.	.	.	-	.	.	.	.	.	.	.	.	.	.	.	.	.	.	.	.	.	A	.	.	.	A	.	.	.	.	.	.	.	.	.	G	.	A	.	-	-	.	.	.	.	.	G	.	.	.	.	.	A	.	.	-
33	*Valerianella microcarpa* VMI0102	.	.	.	.	.	.	C	.	.	.	-	.	.	.	.	.	G	.	.	.	.	.	.	.	.	.	.	.	.	.	.	G	A	.	.	.	.	.	.	.	.	.	G	.	A	.	-	-	.	.	.	.	.	G	.	.	.	.	.	A	.	.	-
34	*Valerianella multidentata* VMU0201	.	.	.	.	.	.	C	.	.	.	A	.	.	.	.	.	.	.	.	.	.	.	.	.	.	.	.	.	.	.	.	G	A	.	.	.	.	.	.	.	.	.	G	.	A	.	-	-	.	.	A	.	.	G	.	.	.	.	.	A	.	.	-
35	*Valerianella multidentata* VMU0301	.	.	.	.	.	.	C	.	.	.	A	.	.	.	.	.	.	.	.	.	.	.	.	.	.	.	.	.	.	.	.	G	A	.	.	.	.	.	.	.	.	.	G	.	A	.	-	-	.	.	A	.	.	G	.	.	.	.	.	A	.	.	-
36	*Valerianella pumila* VPU0103	.	.	.	.	.	.	C	.	G	.	A	.	.	.	.	.	.	.	.	.	.	.	.	.	.	C	.	.	.	.	.	G	A	.	.	.	.	.	.	.	.	.	G	.	A	.	-	-	.	.	.	.	.	G	.	.	.	.	.	A	.	.	-
37	*Valerianella pumila* VPU0304	.	.	.	.	.	.	C	.	G	.	A	.	.	.	.	.	.	.	.	.	.	.	.	.	.	C	.	.	.	.	.	G	A	.	.	.	.	.	.	.	.	.	G	.	A	.	-	-	.	.	.	.	.	G	.	.	.	.	.	A	.	.	-
38	*Valerianella pumila* VPU0402	.	.	.	.	.	.	C	.	G	.	A	.	.	.	.	.	.	.	.	.	.	.	.	.	.	C	.	.	.	.	.	G	A	.	.	.	.	.	.	.	.	.	G	.	A	.	-	-	.	.	.	.	.	G	.	.	.	.	.	A	.	.	-
39	*Valerianella rimosa* VRI0101	.	.	.	.	.	.	C	.	.	.	-	.	.	.	.	.	G	.	.	.	.	.	.	.	.	.	.	.	.	.	.	G	A	.	.	.	.	.	.	.	.	.	G	.	A	.	-	-	.	.	.	.	.	G	.	.	.	.	.	A	.	.	-
40	*Valerianella rimosa* VRI0201	.	.	.	.	.	.	C	.	.	.	-	.	.	.	.	.	G	.	.	.	.	.	.	.	.	.	.	.	.	.	.	G	A	.	.	.	.	.	.	.	.	.	G	.	A	.	-	-	.	.	.	.	.	G	.	.	.	.	.	A	.	.	-

		Position
		5 5 5 5 5 5 5 5 5 5 5 5 5 5 5 5 5 5 5 5 5 5 5 5 5 5 5 5 5 5 5 5 6 6 6 6 6 6 6 6 6 6 6 6 6 6 6 6 6 6 6 6 6 6 6 6 6 6 6 6 6 6 6
		6 6 7 7 7 7 7 7 7 7 7 7 8 8 8 8 8 8 8 8 8 8 9 9 9 9 9 9 9 9 9 9 0 0 0 0 0 0 0 0 0 0 1 1 1 1 1 1 1 1 1 1 2 2 2 2 2 2 2 2 2 2 3
		8 9 0 1 2 3 4 5 6 7 8 9 0 1 2 3 4 5 6 7 8 9 0 1 2 3 4 5 6 7 8 9 0 1 2 3 4 5 6 7 8 9 0 1 2 3 4 5 6 7 8 9 0 1 2 3 4 5 6 7 8 9 0
1	*Centranthus calcitrapae* CC25	- - - - - - - - - - - A T C G C T A G A T T A A T C A T T C A T A - - - T T T A A T A G A - T T T T G T A G T T G A A A T T C
2	*Centranthus calcitrapae* CCACC539	- - - - - - - - - - - . . . . - - - . . . . - . . . .
3	*Fedia cornucopiae* FC73	- - - - - - - - - - - . . T . . . . A T . . A . . . T A G . . . . - . . . . A . . . .
4	*Valerianella carinata* VCA0104	- - - - - - - - - - - . . T . . . . A T . . A . . - - - - . . . . - . . . . A . . . .
5	*Valerianella carinata* VCA0202	- - - - - - - - - - - . . T . . . . A T . . . . . - - - - . . . . - . . . . A . . . .
6	*Valerianella carinata* VCA0302	- - - - - - - - - - - . . T . . . . A T . . . . . - - - - . . . . - . . . . A . . . .
7	*Valerianella carinata* VCA0501	- - - - - - - - - - - . . T . . . . A T . . . . . - - - - . . . . - . . . . A . . . .
8	*Valerianella carinata* VCA0805	- - - - - - - - - - - . . T . . . . A T . . . . . - - - - . . . . - . . . . A . . . .
9	*Valerianella carinata* VCA0918	- - - - - - - - - - - . . T . . . . A T . . . . . - - - - . . . . - . . . . A . . . .
10	*Valerianella carinata* VCA1006	- - - - - - - - - - - . . T . . . . A T . . . . . - - - - . . . . - . . . . A . . . .
11	*Valerianella carinata* VCA1106	- - - - - - - - - - - . . T . . . . A T . . . . . - - - - . . . . - . . . . A . . . .
12	*Valerianella carinata* VCA1202	- - - - - - - - - - - . . T . . . . A T . . . . . - - - - . . . . - . . . . A . . . .
13	*Valerianella coronata* VCO0102	- - - - - - - - - - - . . T . . . . A . . . - - - . . . . . . C T . T . . . . A . . . .
14	*Valerianella coronata* VCO0218	- - - - - - - - - - - . . T . . . . A . . . - - - . . . . . . C T . T . . . . A . . . .
15	*Valerianella coronata* VCO0404	- - - - - - - - - - - . . T . . . . A . . . - - - . . . . . . C T . T . . . . A . . . .
16	*Valerianella coronata* VCO0508	- - - - - - - - - - - . . T . . . . A . . . - - - . . . . . . C T . T . . . . A . . . .
17	*Valerianella coronata* VCO0705	- - - - - - - - - - - . . T . . . . A . . . - - - . . . . . . C T . T . . . . A . . . .
18	*Valerianella coronata* VCO0805	- - - - - - - - - - - . . T . . . . A . . . - - - . . . . . . C T . T . . . . A . . . .
19	*Valerianella coronata* VCO0901	- - - - - - - - - - - . . T . . . . A . . . - - - . . . . . . C T . T . . . . A . . . .
20	*Valerianella dentata* VDE0303	- - - - - - - - - - - . . T T . . . A . . . . A . . . - - - . . . . . . C . . T . . . . A . . . .
21	*Valerianella discoidea* VDI0202	- - - - - - - - - - - . . T . . . . A . . . - - - . . . . . . C T . T . . . . A . . . .
22	*Valerianella discoidea* VDI0412	- - - - - - - - - - - . . T . . . . A . . . - - - . . . . . . C T . T . . . . A . . . .
23	*Valerianella echinata* VEC0101	- - - - - - - - - - - . . T . . . . T . . . A . . . - - - . . . . - . . . . A . . . .
24	*Valerianella eriocarpa* VER0208	- - - - - - - - - - - . . T . . . . A . . . . A . . . - - - . . . . . . C . . T . . . . A . . . .
25	*Valerianella eriocarpa* VER0402	- - - - - - - - - - - . . T . . . . A . . . . A . . . - - - . . . . . . C . . T . . . . A . . . .
26	*Valerianella fusiformis* VFU0103	T A G A T T C T A T G . . T . . . . G - - - A . . . - - - . . G . . . C . . - . . . . A . . . .
27	*Valerianella locusta* VLO0103	- - - - - - - - - - - . . T . . . . A T . . . . . - - - - . . . . - . . . . A . . . .
28	*Valerianella locusta* VLO0305	- - - - - - - - - - - . . T . . . . A T . . . . . - - - - . . . . - . . . . A . . . .
29	*Valerianella locusta* VLO0409	- - - - - - - - - - - . . T . . . . A T . . . . . - - - - . . . . - . . . . A . . . .
30	*Valerianella locusta* VLO0505	- - - - - - - - - - - . . T . . . . A T . . . . . - - - - . . . . - . . . . A . . . .
31	*Valerianella locusta* VLO0609	- - - - - - - - - - - . . T . . . . A T . . . . . - - - - . . . . - . . . . A . . . .
32	*Valerianella lusitanica* VLU0102	- - - - - - - - - - - . . T . . . . A T . . A . . - - - - . . . . - . . . . A . . . .
33	*Valerianella microcarpa* VMI0102	- - - - - - - - - - - . . T . . . . A . . . . A . . . - - - . . . . . . C . . T . . . . A . . . .
34	*Valerianella multidentata* VMU0201	- - - - - - - - - - - . . T . . . . A . . . - - - . . . . . . C T . T . . . . A . . . .
35	*Valerianella multidentata* VMU0301	- - - - - - - - - - - . . T . . . . A . . . - - - . . . . . . C T . T . . . . A . . . .
36	*Valerianella pumila* VPU0103	- - - - - - - - - - - . . T . . . . A . . . - - - . . . . . . C T . T . . . . A . . . .
37	*Valerianella pumila* VPU0304	- - - - - - - - - - - . . T . . . . A . . . - - - . . . . . . C T . T . . . . A . . . .
38	*Valerianella pumila* VPU0402	- - - - - - - - - - - . . T . . . . A . . . - - - . . . . . . C T . T . . . . A . . . .
39	*Valerianella rimosa* VRI0101	- - - - - - - - - - - . . T T . . . A . . . . A . . . - - - . . . . . . C . . T . . . . A . . . .
40	*Valerianella rimosa* VRI0201	- - - - - - - - - - - . . T T . . . A . . . . A . . . - - - . . . . . . C . . T . . . . A . . . .

	Position	631	632	633	634	635	636	637	638	639	640	641	642	643	644	645	646	647	648	649	650	651	652	653	654	655	656	657	658	659	660	661	662	663	664	665	666	667	668	669	670	671	672	673	674	675	676	677	678	679	680	681	682	683	684	685	686	687	688	689	690	691	692	693
1	*Centranthus calcitrapae* CC25	A	A	T	T	C	T	T	T	C	G	T	T	C	C	T	A	T	G	C	T	A	T	G	C	A	T	A	G	A	T	-	C	G	A	G	G	A	T	T	T	T	C	A	G	T	C	C	T	C	T	G	C	T	C	T	A	C	C	A	A	C	T	G
2	*Centranthus calcitrapae* CCACC539	.	.	.	.	.	.	.	.	.	.	.	.	.	.	.	.	.	.	.	.	.	.	.	.	.	.	.	.	.	.	-	.	.	.	.	.	.	.	.	.	.	.	.	.	.	.	.	A	.	.	.	.	.	.	.	.	.	.	.	.	.	.	.
3	*Fedia cornucopiae* FC73	G	.	.	.	.	.	.	.	.	.	.	.	.	.	G	.	.	.	.	.	.	.	.	-	-	.	.	A	T	.	T	.	.	.	.	.	.	.	.	.	.	.	.	.	.	.	.	.	.	.	.	.	.	.	.	.	.	.	.	.	.	.	.
4	*Valerianella carinata* VCA0104	G	.	.	.	.	.	.	.	.	.	.	.	.	.	G	.	.	.	.	.	.	.	.	.	.	.	.	A	T	.	T	.	.	.	.	.	.	.	.	.	.	.	.	.	.	.	.	.	.	.	.	.	.	.	.	.	.	.	.	.	.	.	.
5	*Valerianella carinata* VCA0202	G	.	.	.	.	.	.	.	.	.	.	.	.	.	G	.	.	.	.	.	.	.	.	.	.	.	.	A	T	.	T	.	.	.	.	.	.	.	.	.	.	.	.	.	.	.	.	.	.	.	.	.	.	.	.	.	.	.	.	.	.	.	.
6	*Valerianella carinata* VCA0302	G	.	.	.	.	.	.	.	.	.	.	.	.	.	G	.	.	.	.	.	.	.	.	.	.	.	.	A	T	.	T	.	.	.	.	.	.	.	.	.	.	.	.	.	.	.	.	.	.	.	.	.	.	.	.	.	.	.	.	.	.	.	.
7	*Valerianella carinata* VCA0501	G	.	.	.	.	.	.	.	.	.	.	.	.	.	G	.	.	.	.	.	.	.	.	.	.	.	.	A	T	.	T	.	.	.	.	.	.	.	.	.	.	.	.	.	.	.	.	.	.	.	.	.	.	.	.	.	.	.	.	.	.	.	.
8	*Valerianella carinata* VCA0805	G	.	.	.	.	.	.	.	.	.	.	.	.	.	G	.	.	.	.	.	.	.	.	.	.	.	.	A	T	.	T	.	.	.	.	.	.	.	.	.	.	.	.	.	.	.	.	.	.	.	.	.	.	.	.	.	.	.	.	.	.	.	.
9	*Valerianella carinata* VCA0918	G	.	.	.	.	.	.	.	.	.	.	.	.	.	G	.	.	.	.	.	.	.	.	.	.	.	.	A	T	.	T	.	.	.	.	.	.	.	.	.	.	.	.	.	.	.	.	.	.	.	.	.	.	.	.	.	.	.	.	.	.	.	.
10	*Valerianella carinata* VCA1006	G	.	.	.	.	.	.	.	.	.	.	.	.	.	G	.	.	.	.	.	.	.	.	.	.	.	.	A	T	.	T	.	.	.	.	.	.	.	.	.	.	.	.	.	.	.	.	.	.	.	.	.	.	.	.	.	.	.	.	.	.	.	.
11	*Valerianella carinata* VCA1106	G	.	.	.	.	.	.	.	.	.	.	.	.	.	G	.	.	.	.	.	.	.	.	.	.	.	.	A	T	.	T	.	.	.	.	.	.	.	.	.	.	.	.	.	.	.	.	.	.	.	.	.	.	.	.	.	.	.	.	.	.	.	.
12	*Valerianella carinata* VCA1202	G	.	.	.	.	.	.	.	.	.	.	.	.	.	G	.	.	.	.	.	.	.	.	.	.	.	.	A	T	.	T	.	.	.	.	.	.	.	.	.	.	.	.	.	.	.	.	.	.	.	.	.	.	.	.	.	.	.	.	.	.	.	.
13	*Valerianella coronata* VCO0102	G	.	.	.	.	.	.	.	.	.	.	C	.	.	G	.	.	.	.	.	.	.	.	.	.	.	.	A	T	.	T	.	.	.	.	.	.	.	.	.	.	.	.	.	.	.	.	.	.	.	.	.	.	.	.	.	.	.	.	.	.	.	.
14	*Valerianella coronata* VCO0218	G	.	.	.	.	.	.	.	.	.	.	C	.	.	G	.	.	.	.	.	.	.	.	.	.	.	.	A	T	.	T	.	.	.	.	.	.	.	.	.	.	.	.	.	.	.	.	.	.	.	.	.	.	.	.	.	.	.	.	.	.	.	.
15	*Valerianella coronata* VCO0404	G	.	.	.	.	.	.	.	.	.	.	C	.	.	G	.	.	.	.	.	.	.	.	.	.	.	.	A	T	.	T	.	.	.	.	.	.	.	.	.	.	.	.	.	.	.	.	.	.	.	.	.	.	.	.	.	.	.	.	.	.	.	.
16	*Valerianella coronata* VCO0508	G	.	.	.	.	.	.	.	.	.	.	C	.	.	G	.	.	.	.	.	.	.	.	.	.	.	.	A	T	.	T	.	.	.	.	.	.	.	.	.	.	.	.	.	.	.	.	.	.	.	.	.	.	.	.	.	.	.	.	.	.	.	.
17	*Valerianella coronata* VCO0705	G	.	.	.	.	.	.	.	.	.	.	C	.	.	G	.	.	.	.	.	.	.	.	.	.	.	.	A	T	.	T	.	.	.	.	.	.	.	.	.	.	.	.	.	.	.	.	.	.	.	.	.	.	.	.	.	.	.	.	.	.	.	.
18	*Valerianella coronata* VCO0805	G	.	.	.	.	.	.	.	.	.	.	C	.	.	G	.	.	.	.	.	.	.	.	.	.	.	.	A	T	.	T	.	.	.	.	.	.	.	.	.	.	.	.	.	.	.	.	.	.	.	.	.	.	.	.	.	.	.	.	.	.	.	.
19	*Valerianella coronata* VCO0901	G	.	.	.	.	.	.	.	.	.	.	C	.	.	G	.	.	.	.	.	.	.	.	.	.	.	.	A	T	.	T	.	.	.	.	.	.	.	.	.	.	.	.	.	.	.	.	.	.	.	.	.	.	.	.	.	.	.	.	.	.	.	.
20	*Valerianella dentata* VDE0303	G	.	.	.	.	.	.	.	T	-	.	.	.	.	G	.	.	.	.	.	.	.	.	.	.	-	.	A	T	.	T	.	.	.	.	.	.	.	.	.	.	.	.	.	.	.	.	.	.	.	.	.	.	.	.	.	.	.	.	.	.	.	.
21	*Valerianella discoidea* VDI0202	G	.	.	.	.	.	.	.	.	.	.	C	.	.	G	.	.	.	.	.	.	.	.	.	.	.	.	A	T	.	T	.	.	.	.	.	.	.	.	.	.	.	.	.	.	.	.	.	.	.	.	.	.	.	.	.	.	.	.	.	.	.	.
22	*Valerianella discoidea* VDI0412	G	.	.	.	.	.	.	.	.	.	.	C	.	.	G	.	.	.	.	.	.	.	.	.	.	.	.	A	T	.	T	.	.	.	.	.	.	.	.	.	.	.	.	.	.	.	.	.	.	.	.	.	.	.	.	.	.	.	.	.	.	.	.
23	*Valerianella echinata* VEC0101	G	.	.	.	.	.	.	.	.	.	.	C	.	.	G	.	.	.	.	.	.	.	.	.	.	.	.	A	T	.	T	.	.	.	.	.	.	.	.	.	.	.	.	.	.	.	.	.	.	.	.	.	.	.	.	.	.	.	.	.	.	.	.
24	*Valerianella eriocarpa* VER0208	G	.	.	.	.	.	.	.	.	.	.	.	.	.	G	.	.	A	.	.	.	.	.	.	.	-	.	A	T	.	T	.	.	.	.	.	.	.	.	.	.	.	.	.	.	.	.	.	.	.	.	.	.	.	.	.	.	.	.	.	.	.	.
25	*Valerianella eriocarpa* VER0402	G	.	.	.	.	.	.	.	.	.	.	.	.	.	G	.	.	A	.	.	.	.	.	.	.	-	.	A	T	.	T	.	.	.	.	.	.	.	.	.	.	.	.	.	.	.	.	.	.	.	.	.	.	.	.	.	.	.	.	.	.	.	.
26	*Valerianella fusiformis* VFU0103	.	.	.	.	.	.	.	.	.	.	.	.	.	.	G	.	.	.	.	.	.	C	.	.	.	.	.	A	T	.	T	.	.	.	.	.	.	.	.	.	.	.	.	.	.	.	.	.	.	.	.	.	.	.	.	.	.	.	.	.	.	.	.
27	*Valerianella locusta* VLO0103	G	.	.	.	.	.	.	.	.	.	.	.	.	.	G	.	.	.	.	.	.	.	.	.	.	.	.	A	T	.	T	.	.	.	.	.	.	.	.	.	.	.	.	.	.	.	.	.	.	.	.	.	.	.	.	.	.	.	.	.	.	.	.
28	*Valerianella locusta* VLO0305	G	.	.	.	.	.	.	.	.	.	.	.	.	.	G	.	.	.	.	.	.	.	.	.	.	.	.	A	T	.	T	.	.	.	.	.	.	.	.	.	.	.	.	.	.	.	.	.	.	.	.	.	.	.	.	.	.	.	.	.	.	.	.
29	*Valerianella locusta* VLO0409	G	.	.	.	.	.	.	.	.	.	.	.	.	.	G	.	.	.	.	.	.	.	.	.	.	.	.	A	T	.	T	.	.	.	.	.	.	.	.	.	.	.	.	.	.	.	.	.	.	.	.	.	.	.	.	.	.	.	.	.	.	.	.
30	*Valerianella locusta* VLO0505	G	.	.	.	.	.	.	.	.	.	.	.	.	.	G	.	.	.	.	.	.	.	.	.	.	.	.	A	T	.	T	.	.	.	.	.	.	.	.	.	.	.	.	.	.	.	.	.	.	.	.	.	.	.	.	.	.	.	.	.	.	.	.
31	*Valerianella locusta* VLO0609	G	.	.	.	.	.	.	.	.	.	.	.	.	.	G	.	.	.	.	.	.	.	.	.	.	.	.	A	T	.	T	.	.	.	.	.	.	.	.	.	.	.	.	.	.	.	.	.	.	.	.	.	.	.	.	.	.	.	.	.	.	.	.
32	*Valerianella lusitanica* VLU0102	G	.	.	.	.	.	.	.	.	.	.	.	.	.	G	.	.	.	.	.	.	.	.	.	.	.	.	A	T	.	T	.	.	.	.	.	.	.	.	.	.	.	.	.	.	.	.	.	.	.	.	.	.	.	.	.	.	.	.	.	.	.	.
33	*Valerianella microcarpa* VMI0102	G	.	.	.	.	.	.	.	.	.	.	.	.	.	G	.	.	.	.	.	.	.	.	.	.	-	.	A	T	.	T	.	.	.	.	.	.	.	.	.	.	.	.	.	.	.	.	.	.	.	.	.	.	.	.	.	.	.	.	.	.	.	.
34	*Valerianella multidentata* VMU0201	G	.	.	.	.	.	.	.	.	.	.	C	.	.	G	.	.	.	.	.	.	.	.	.	.	.	.	A	T	.	T	.	.	.	.	.	.	.	.	.	.	.	.	.	.	.	.	.	.	.	.	.	.	.	.	.	.	.	.	.	.	.	.
35	*Valerianella multidentata* VMU0301	G	.	.	.	.	.	.	.	.	.	.	C	.	.	G	.	.	.	.	.	.	.	.	.	.	.	.	A	T	.	T	.	.	.	.	.	.	.	.	.	.	.	.	.	.	.	.	.	.	.	.	.	.	.	.	.	.	.	.	.	.	.	.
36	*Valerianella pumila* VPU0103	G	.	.	.	.	.	.	.	.	.	.	C	.	.	G	.	.	.	.	.	.	.	.	.	.	.	.	A	T	.	T	.	.	.	.	.	.	.	.	.	.	.	.	.	.	.	.	.	.	.	.	.	.	.	.	.	.	.	.	.	.	.	.
37	*Valerianella pumila* VPU0304	G	.	.	.	.	.	.	.	.	.	.	C	.	.	G	.	.	.	.	.	.	.	.	.	.	.	.	A	T	.	T	.	.	.	.	.	.	.	.	.	.	.	.	.	.	.	.	.	.	.	.	.	.	.	.	.	.	.	.	.	.	.	.
38	*Valerianella pumila* VPU0402	G	.	.	.	.	.	.	.	.	.	.	C	.	.	G	.	.	.	.	.	.	.	.	.	.	.	.	A	T	.	T	.	.	.	.	.	.	.	.	.	.	.	.	.	.	.	.	.	.	.	.	.	.	.	.	.	.	.	.	.	.	.	.
39	*Valerianella rimosa* VRI0101	G	.	.	.	.	.	.	.	T	-	.	.	.	.	G	.	.	.	.	.	.	.	.	.	.	-	.	A	T	.	T	.	.	.	.	.	.	.	.	.	.	.	.	.	.	.	.	.	.	.	.	.	.	.	.	.	.	.	.	.	.	.	.
40	*Valerianella rimosa* VRI0201	G	.	.	.	.	.	.	.	T	-	.	.	.	.	G	.	.	.	.	.	.	.	.	.	.	-	.	A	T	.	T	.	.	.	.	.	.	.	.	.	.	.	.	.	.	.	.	.	.	.	.	.	.	.	.	.	.	.	.	.	.	.	.

Position

		694	695	696	697	698	699	700	701	702	703	704	705	706	707	708	709	710	711	712	713	714	715	716	717	718	719	720	721	722	723	724	725	726	727	728	729	730	731	732	733	734	735	736	737	738	739	740	741	742	743	744	745	746	747	748	749	750	751	752	753	754	755	756
1	*Centranthus calcitrapae* CC25	A	G	C	T	A	T	C	C	C	G	A	C	C	A	T	T	C	C	C	A	C	C	G	C	T	G	C	A	T	T	C	T	C	A	T	T	T	T	G	C	T	A	G	A	T	A	A	C	T	C	G	G	G	T	C	T	A	T	G	T	C	A	A
2	*Centranthus calcitrapae* CCACC539	.	.	.	.	.	.	.	.	.	.	.	.	.	.	.	.	.	.	.	.	.	.	.	.	.	.	.	.	.	.	.	.	.	.	.	.	.	.	.	.	.	.	.	.	.	.	.	.	.	.	.	.	.	.	.	.	.	.	.	.	.	.	.
3	*Fedia cornucopiae* FC73	.	.	.	.	.	.	.	.	.	.	.	.	.	.	.	.	G	T	.	G	A	.	.	.	.	.	.	.	.	.	.	.	.	.	.	.	.	.	.	.	.	.	.	.	.	.	.	.	.	.	.	.	.	.	.	.	.	.	.	.	.	.	.
4	*Valerianella carinata* VCA0104	.	.	.	.	.	.	.	.	.	.	.	.	.	.	.	.	G	.	.	G	A	.	.	.	.	.	.	.	.	.	.	.	.	.	.	.	.	.	.	.	.	.	.	.	G	.	.	.	.	.	.	.	.	.	.	.	.	.	.	.	.	.	.
5	*Valerianella carinata* VCA0202	.	.	.	.	.	.	.	.	.	.	.	.	.	.	.	.	G	.	.	G	A	.	.	.	.	.	.	.	.	.	.	.	.	.	.	.	.	.	.	.	.	.	.	.	G	.	.	.	.	.	.	.	.	.	.	.	.	.	.	.	.	.	.
6	*Valerianella carinata* VCA0302	.	.	.	.	.	.	.	.	.	.	.	.	.	.	.	.	G	.	.	G	A	.	.	.	.	.	.	.	.	.	.	.	.	.	.	.	.	.	.	.	.	.	.	.	G	.	.	.	.	.	.	.	.	.	.	.	.	.	.	.	.	.	.
7	*Valerianella carinata* VCA0501	.	.	.	.	.	.	.	.	.	.	.	.	.	.	.	.	G	.	.	G	A	.	.	.	.	.	.	.	.	.	.	.	.	.	.	.	.	.	.	.	.	.	.	.	G	.	.	.	.	.	.	.	.	.	.	.	.	.	.	.	.	.	.
8	*Valerianella carinata* VCA0805	.	.	.	.	.	.	.	.	.	.	.	.	.	.	.	.	G	.	.	G	A	.	.	.	.	.	.	.	.	.	.	.	.	.	.	.	.	.	.	.	.	.	.	.	G	.	.	.	.	.	.	.	.	.	.	.	.	.	.	.	.	.	.
9	*Valerianella carinata* VCA0918	.	.	.	.	.	.	.	.	.	.	.	.	.	.	.	.	G	.	.	G	A	.	.	.	.	.	.	.	.	.	.	.	.	.	.	.	.	.	.	.	.	.	.	.	G	.	.	.	.	.	.	.	.	.	.	.	.	.	.	.	.	.	.
10	*Valerianella carinata* VCA1006	.	.	.	.	.	.	.	.	.	.	.	.	.	.	.	.	G	.	.	G	A	.	.	.	.	.	.	.	.	.	.	.	.	.	.	.	.	.	.	.	.	.	.	.	G	.	.	.	.	.	.	.	.	.	.	.	.	.	.	.	.	.	.
11	*Valerianella carinata* VCA1106	.	.	.	.	.	.	.	.	.	.	.	.	.	.	.	.	G	.	.	G	A	.	.	.	.	.	.	.	.	.	.	.	.	.	.	.	.	.	.	.	.	.	.	.	G	.	.	.	.	.	.	.	.	.	.	.	.	.	.	.	.	.	.
12	*Valerianella carinata* VCA1202	.	.	.	.	.	.	.	.	.	.	.	.	.	.	.	.	G	.	.	G	A	.	.	.	.	.	.	.	.	.	.	.	.	.	.	.	.	.	.	.	.	.	.	.	G	.	.	.	.	.	.	.	.	.	.	.	.	.	.	.	.	.	.
13	*Valerianella coronata* VCO0102	.	.	.	.	.	.	.	.	.	.	.	.	.	.	.	.	G	.	.	G	A	.	.	.	.	.	.	.	.	.	.	.	.	.	.	.	.	.	.	.	.	.	.	.	.	.	.	.	.	.	.	.	.	.	.	.	.	.	.	.	.	.	.
14	*Valerianella coronata* VCO0218	.	.	.	.	.	.	.	.	.	.	.	.	.	.	.	.	G	.	.	G	A	.	.	.	.	.	.	.	.	.	.	.	.	.	.	.	.	.	.	.	.	.	.	.	.	.	.	.	.	.	.	.	.	.	.	.	.	.	.	.	.	.	.
15	*Valerianella coronata* VCO0404	.	.	.	.	.	.	.	.	.	.	.	.	.	.	.	.	G	.	.	G	A	.	.	.	.	.	.	.	.	.	.	.	.	.	.	.	.	.	.	.	.	.	.	.	.	.	.	.	.	.	.	.	.	.	.	.	.	.	.	.	.	.	.
16	*Valerianella coronata* VCO0508	.	.	.	.	.	.	.	.	.	.	.	.	.	.	.	.	G	.	.	G	A	.	.	.	.	.	.	.	.	.	.	.	.	.	.	.	.	.	.	.	.	.	.	.	.	.	.	.	.	.	.	.	.	.	.	.	.	.	.	.	.	.	.
17	*Valerianella coronata* VCO0705	.	.	.	.	.	.	.	.	.	.	.	.	.	.	.	.	G	.	.	G	A	.	.	.	.	.	.	.	.	.	.	.	.	.	.	.	.	.	.	.	.	.	.	.	.	.	.	.	.	.	.	.	.	.	.	.	.	.	.	.	.	.	.
18	*Valerianella coronata* VCO0805	.	.	.	.	.	.	.	.	.	.	.	.	.	.	.	.	G	.	.	G	A	.	.	.	.	.	.	.	.	.	.	.	.	.	.	.	.	.	.	.	.	.	.	.	.	.	.	.	.	.	.	.	.	.	.	.	.	.	.	.	.	.	.
19	*Valerianella coronata* VCO0901	.	.	.	.	.	.	.	.	.	.	.	.	.	.	.	.	G	.	.	G	A	.	.	.	.	.	.	.	.	.	.	.	.	.	.	.	.	.	.	.	.	.	.	.	.	.	.	.	.	.	.	.	.	.	.	.	.	.	.	.	.	.	.
20	*Valerianella dentata* VDE0303	.	.	.	.	.	.	.	.	.	.	.	.	.	.	.	.	G	.	.	G	A	.	.	.	.	.	.	.	.	.	.	.	.	.	.	.	.	.	.	.	.	.	.	.	.	.	.	.	.	.	.	.	.	.	.	.	.	.	.	.	.	.	.
21	*Valerianella discoidea* VDI0202	.	.	.	.	.	.	.	.	.	.	.	.	.	.	.	.	G	.	.	G	A	.	.	.	.	.	.	.	.	.	.	.	.	.	.	.	.	.	.	.	.	.	.	.	.	.	.	.	.	.	.	.	.	.	.	.	.	.	.	.	.	.	.
22	*Valerianella discoidea* VDI0412	.	.	.	.	.	.	.	.	.	.	.	.	.	.	.	.	G	.	.	G	A	.	.	.	.	.	.	.	.	.	.	.	.	.	.	.	.	.	.	.	.	.	.	.	.	.	.	.	.	.	.	.	.	.	.	.	.	.	.	.	.	.	.
23	*Valerianella echinata* VEC0101	.	.	.	.	.	.	.	.	.	.	.	.	.	.	.	.	G	.	T	G	A	.	.	.	.	.	.	.	.	.	.	.	.	.	.	.	.	.	.	.	.	.	.	.	.	.	.	.	.	.	.	.	.	.	.	.	.	.	.	.	.	.	.
24	*Valerianella eriocarpa* VER0208	.	.	.	.	.	.	.	.	.	.	.	.	.	.	.	.	G	.	.	G	A	.	.	.	.	.	.	.	.	.	.	.	.	.	.	.	.	.	.	.	.	.	.	.	.	.	.	.	.	.	.	.	.	.	.	.	.	.	.	.	.	.	.
25	*Valerianella eriocarpa* VER0402	.	.	.	.	.	.	.	.	.	.	.	.	.	.	.	.	G	.	A	G	A	.	.	.	.	.	.	.	.	.	.	.	.	.	.	.	.	.	.	.	.	.	.	.	.	.	.	.	.	.	.	.	.	.	.	.	.	.	.	.	.	.	.
26	*Valerianella fusiformis* VFU0103	.	.	.	.	.	.	.	.	.	.	.	.	.	.	.	.	G	.	.	G	A	.	.	.	.	.	.	.	.	.	.	.	.	.	.	.	.	.	.	.	.	.	.	.	.	.	.	.	.	T	.	.	.	.	.	.	.	.	.	.	.	.	.
27	*Valerianella locusta* VLO0103	.	.	.	.	.	.	.	.	.	.	.	.	.	.	.	.	G	.	.	G	A	.	.	.	.	.	.	.	.	.	.	.	.	.	.	.	.	.	.	.	.	.	.	.	G	.	.	.	.	.	.	.	.	.	.	.	.	.	.	.	.	.	.
28	*Valerianella locusta* VLO0305	.	.	.	.	.	.	.	.	.	.	.	.	.	.	.	.	G	.	.	G	A	.	.	.	.	.	.	.	.	.	.	.	.	.	.	.	.	.	.	.	.	.	.	.	G	.	.	.	.	.	.	.	.	.	.	.	.	.	.	.	.	.	.
29	*Valerianella locusta* VLO0409	.	.	.	.	.	.	.	.	.	.	.	.	.	.	.	.	G	.	.	G	A	.	.	.	.	.	.	.	.	.	.	.	.	.	.	.	.	.	.	.	.	.	.	.	G	.	.	.	.	.	.	.	.	.	.	.	.	.	.	.	.	.	.
30	*Valerianella locusta* VLO0505	.	.	.	.	.	.	.	.	.	.	.	.	.	.	.	.	G	.	.	G	A	.	.	.	.	.	.	.	.	.	.	.	.	.	.	.	.	.	.	.	.	.	.	.	G	.	.	.	.	.	.	.	.	.	.	.	.	.	.	.	.	.	.
31	*Valerianella locusta* VLO0609	.	.	.	.	.	.	.	.	.	.	.	.	.	.	.	.	G	.	.	G	A	.	.	.	.	.	.	.	.	.	.	.	.	.	.	.	.	.	.	.	.	.	.	.	G	.	.	.	.	.	.	.	.	.	.	.	.	.	.	.	.	.	.
32	*Valerianella lusitanica* VLU0102	.	.	.	.	.	.	.	.	.	.	.	.	.	.	.	.	G	.	.	G	A	.	.	.	.	.	.	.	.	.	.	.	.	.	.	.	.	.	.	.	.	.	.	.	G	.	.	.	.	.	.	.	.	.	.	.	.	.	.	.	.	.	.
33	*Valerianella microcarpa* VMI0102	.	.	.	.	.	.	.	.	.	.	.	.	.	.	.	.	G	.	.	G	A	.	.	.	.	.	.	.	.	.	.	.	.	.	.	.	.	.	.	.	.	.	.	.	.	.	.	.	.	.	.	.	.	.	.	.	.	.	.	.	.	.	.
34	*Valerianella multidentata* VMU0201	.	.	.	.	.	.	.	.	.	.	.	.	.	.	.	.	G	.	.	G	A	.	.	.	.	.	.	.	.	.	.	.	.	.	.	.	.	.	.	.	.	.	.	.	.	.	.	.	.	.	.	.	.	.	.	.	.	.	.	.	.	.	.
35	*Valerianella multidentata* VMU0301	.	.	.	.	.	.	.	.	.	.	.	.	.	.	.	.	G	.	.	G	A	.	.	.	.	.	.	.	.	.	.	.	.	.	.	.	.	.	.	.	.	.	.	.	.	.	.	.	.	.	.	.	.	.	.	.	.	.	.	.	.	.	.
36	*Valerianella pumila* VPU0103	.	.	.	.	.	.	.	.	.	.	.	.	.	.	.	.	G	.	.	G	A	.	.	.	.	.	.	.	.	.	.	.	.	.	.	.	.	.	.	.	.	.	.	.	.	.	.	.	.	.	.	.	.	.	.	.	.	.	.	.	.	.	.
37	*Valerianella pumila* VPU0304	.	.	.	.	.	.	.	.	.	.	.	.	.	.	.	.	G	.	.	G	A	.	.	.	.	.	.	.	.	.	.	.	.	.	.	.	.	.	.	.	.	.	.	.	.	.	.	.	.	.	.	.	.	.	.	.	.	.	.	.	.	.	.
38	*Valerianella pumila* VPU0402	.	.	.	.	.	.	.	.	.	.	.	.	.	.	.	.	G	.	.	G	A	.	.	.	.	.	.	.	.	.	.	.	.	.	.	.	.	.	.	.	.	.	.	.	.	.	.	.	.	.	.	.	.	.	.	.	.	.	.	.	.	.	.
39	*Valerianella rimosa* VRI0101	.	.	.	.	.	.	.	.	.	.	.	.	.	.	.	.	G	.	.	G	A	.	.	.	.	.	.	.	.	.	.	.	.	.	.	.	.	.	.	.	.	.	.	.	.	.	.	.	.	.	.	.	.	.	.	.	.	.	.	.	.	.	.
40	*Valerianella rimosa* VRI0201	.	.	.	.	.	.	.	.	.	.	.	.	.	.	.	.	G	.	.	G	A	.	.	.	.	.	.	.	.	.	.	.	.	.	.	.	.	.	.	.	.	.	.	.	.	.	.	.	.	.	.	.	.	.	.	.	.	.	.	.	.	.	.

#	Taxon	757	758	759	760	761	762	763	764	765	766	767	768	769	770	771	772	773	774	775	776	777	778	779	780	781	782	783	784	785	786	787	788	789	790	791	792	793	794	795	796	797	798	799	800	801	802	803	804	805	806	807	808	809	810	811	812	813	814	815	816	817	818	819
1	*Centranthus calcitrapae* CC25	C	T	G	A	A	G	G	G	G	T	A	T	T	A	C	A	A	A	G	T	C	T	T	A	T	C	C	T	C	A	T	C	C	G	G	T	A	A	T	T	T	A	T	T	G	G	-	-	-	-	-	A	T	C	T	T	C	A	A	A	A	G	A
2	*Centranthus calcitrapae* CCACC539	.	.	.	.	.	.	.	.	.	.	.	.	.	.	.	.	.	.	.	.	.	.	.	.	.	.	.	.	.	.	.	.	.	.	.	.	.	.	.	.	.	.	.	.	.	.	-	-	-	-	-	.	.	.	.	.	.	.	.	.	.	.	.
3	*Fedia cornucopiae* FC73	.	.	.	.	.	.	.	.	.	.	C	.	.	.	.	.	.	.	.	.	.	.	.	.	.	.	.	.	G	.	.	.	.	A	.	C	.	.	.	.	.	.	.	.	.	.	A	T	T	G	G	.	.	.	.	.	.	.	.	.	.	.	.
4	*Valerianella carinata* VCA0104	.	.	.	.	.	.	.	.	.	.	C	.	.	.	.	.	.	.	.	.	.	.	.	.	.	.	.	.	G	.	.	.	.	A	.	.	.	.	.	.	.	C	.	.	.	.	-	-	-	-	-	.	.	.	.	.	.	.	.	.	-	-	-
5	*Valerianella carinata* VCA0202	.	.	.	.	.	.	.	.	.	.	C	.	.	.	.	.	.	.	.	.	.	.	.	.	.	.	.	.	G	.	.	.	.	A	.	.	.	.	.	.	.	C	.	.	.	.	-	-	-	-	-	.	.	.	.	.	.	.	.	.	-	-	-
6	*Valerianella carinata* VCA0302	.	.	.	.	.	.	.	.	.	.	C	.	.	.	.	.	.	.	.	.	.	.	.	.	.	.	.	.	G	.	.	.	.	A	.	.	.	.	.	.	.	C	.	.	.	.	-	-	-	-	-	.	.	.	.	.	.	.	.	.	-	-	-
7	*Valerianella carinata* VCA0501	.	.	.	.	.	.	.	.	.	.	C	.	.	.	.	.	.	.	.	.	.	.	.	.	.	.	.	.	G	.	.	.	.	A	.	.	.	.	.	.	.	C	.	.	.	.	-	-	-	-	-	.	.	.	.	.	.	.	.	.	-	-	-
8	*Valerianella carinata* VCA0805	.	.	.	.	.	.	.	.	.	.	C	.	.	.	.	.	.	.	.	.	.	.	.	.	.	.	.	.	G	.	.	.	.	A	.	.	.	.	.	.	.	C	.	.	.	.	-	-	-	-	-	.	.	.	.	.	.	.	.	.	-	-	-
9	*Valerianella carinata* VCA0918	.	.	.	.	.	.	.	.	.	.	C	.	.	.	.	.	.	.	.	.	.	.	.	.	.	.	.	.	G	.	.	.	.	A	.	.	.	.	.	.	.	C	.	.	.	.	-	-	-	-	-	.	.	.	.	.	.	.	.	.	-	-	-
10	*Valerianella carinata* VCA1006	.	.	.	.	.	.	.	.	.	.	C	.	.	.	.	.	.	.	.	.	.	.	.	.	.	.	.	.	G	.	.	.	.	A	.	.	.	.	.	.	.	C	.	.	.	.	-	-	-	-	-	.	.	.	.	.	.	.	.	.	-	-	-
11	*Valerianella carinata* VCA1106	.	.	.	.	.	.	.	.	.	.	C	.	.	.	.	.	.	.	.	.	.	.	.	.	.	.	.	.	G	.	.	.	.	A	.	.	.	.	.	.	.	C	.	.	.	.	-	-	-	-	-	.	.	.	.	.	.	.	.	.	-	-	-
12	*Valerianella carinata* VCA1202	.	.	.	.	.	.	.	.	.	.	C	.	.	.	.	.	.	.	.	.	.	.	.	.	.	.	.	.	G	.	.	.	.	A	.	.	.	.	.	.	.	C	.	.	.	.	-	-	-	-	-	.	.	.	.	.	.	.	.	.	-	-	-
13	*Valerianella coronata* VCO0102	.	.	.	.	.	.	.	.	.	.	C	.	.	.	.	.	.	.	.	.	.	.	.	.	.	.	.	.	G	.	.	.	.	A	.	C	.	.	.	.	.	.	.	.	.	.	-	-	-	-	-	.	.	.	.	.	.	.	.	.	.	.	.
14	*Valerianella coronata* VCO0218	.	.	.	.	.	.	.	.	.	.	C	.	.	.	.	.	.	.	.	.	.	.	.	.	.	.	.	.	G	.	.	.	.	A	.	C	.	.	.	.	.	.	.	.	.	.	-	-	-	-	-	.	.	.	.	.	.	.	.	.	.	.	.
15	*Valerianella coronata* VCO0404	.	.	.	.	.	.	.	.	.	.	C	.	.	.	.	.	.	.	.	.	.	.	.	.	.	.	.	.	G	.	.	.	.	A	.	C	.	.	.	.	.	.	.	.	.	.	-	-	-	-	-	.	.	.	.	.	.	.	.	.	.	.	.
16	*Valerianella coronata* VCO0508	.	.	.	.	.	.	.	.	.	.	C	.	.	.	.	.	.	.	.	.	.	.	.	.	.	.	.	.	G	.	.	.	.	A	.	C	.	.	.	.	.	.	.	.	.	.	-	-	-	-	-	.	.	.	.	.	.	.	.	.	.	.	.
17	*Valerianella coronata* VCO0705	.	.	.	.	.	.	.	.	.	.	C	.	.	.	.	.	.	.	.	.	.	.	.	.	.	.	.	.	G	.	.	.	.	A	.	C	.	.	.	.	.	.	.	.	.	.	-	-	-	-	-	.	.	.	.	.	.	.	.	.	.	.	.
18	*Valerianella coronata* VCO0805	.	.	.	.	.	.	.	.	.	.	C	.	.	.	.	.	.	.	.	.	.	.	.	.	.	.	.	.	G	.	.	.	.	A	.	C	.	.	.	.	.	.	.	.	.	.	-	-	-	-	-	.	.	.	.	.	.	.	.	.	.	.	.
19	*Valerianella coronata* VCO0901	.	.	.	.	.	.	.	.	.	.	C	.	.	.	.	.	.	.	.	.	.	.	.	.	.	.	.	.	G	.	.	.	.	A	.	C	.	.	.	.	.	.	.	.	.	.	-	-	-	-	-	.	.	.	.	.	.	.	.	.	.	.	.
20	*Valerianella dentata* VDE0303	.	.	.	.	.	.	.	.	.	.	C	.	.	.	.	.	.	.	.	.	.	.	.	.	.	.	.	.	A	.	.	.	.	A	.	C	.	.	.	.	.	.	.	.	.	.	-	-	-	-	-	.	.	.	.	.	.	.	.	.	.	.	.
21	*Valerianella discoidea* VDI0202	.	.	.	.	.	.	.	.	.	.	C	.	.	.	.	.	.	.	.	.	.	.	.	.	.	.	.	.	G	.	.	.	.	A	.	C	.	.	.	.	.	.	.	.	.	.	-	-	-	-	-	.	.	.	.	.	.	.	.	.	.	.	.
22	*Valerianella discoidea* VDI0412	.	.	.	.	.	.	.	.	.	.	C	.	.	.	.	.	.	.	.	.	.	.	.	.	.	.	.	.	G	.	.	.	.	A	.	C	.	.	.	.	.	.	.	.	.	.	-	-	-	-	-	.	.	.	.	.	.	.	.	.	.	.	.
23	*Valerianella echinata* VEC0101	.	.	.	.	.	.	.	.	.	.	C	.	.	.	.	.	.	.	.	.	.	.	.	.	.	.	.	.	G	.	.	.	.	A	.	.	.	.	.	.	.	C	.	.	.	.	-	-	-	-	-	.	.	.	.	.	.	.	.	.	.	.	.
24	*Valerianella eriocarpa* VER0208	.	.	.	.	.	.	.	.	.	.	C	.	.	.	.	.	.	.	.	.	.	.	.	.	.	.	.	.	A	.	.	.	.	A	.	C	.	.	.	.	.	T	.	.	.	.	-	-	-	-	-	.	.	.	.	.	.	.	.	.	.	.	.
25	*Valerianella eriocarpa* VER0402	.	.	.	.	.	.	.	.	.	.	C	.	.	.	.	.	.	.	.	.	.	.	.	.	.	.	.	.	A	.	.	.	.	A	.	C	.	.	.	.	.	T	.	.	.	.	-	-	-	-	-	.	.	.	.	.	.	.	.	.	.	.	.
26	*Valerianella fusiformis* VFU0103	.	.	.	.	.	.	.	.	.	.	C	.	.	.	.	.	.	.	.	.	.	.	.	.	.	.	.	.	G	.	.	.	.	A	T	A	.	.	.	.	.	T	.	.	.	.	-	-	-	-	-	.	.	.	.	.	.	.	.	.	.	.	.
27	*Valerianella locusta* VLO0103	.	.	.	.	.	.	.	.	.	.	C	.	.	.	.	.	.	.	.	.	.	.	.	.	.	.	.	.	G	.	.	.	.	A	.	.	.	.	.	.	.	C	.	.	.	.	-	-	-	-	-	.	.	.	.	.	.	.	.	.	-	-	-
28	*Valerianella locusta* VLO0305	.	.	.	.	.	.	.	.	.	.	C	.	.	.	.	.	.	.	.	.	.	.	.	.	.	.	.	.	G	.	.	.	.	A	.	.	.	.	.	.	.	C	.	.	.	.	-	-	-	-	-	.	.	.	.	.	.	.	.	.	-	-	-
29	*Valerianella locusta* VLO0409	.	.	.	.	.	.	.	.	.	.	C	.	.	.	.	.	.	.	.	.	.	.	.	.	.	.	.	.	G	.	.	.	.	A	.	.	.	.	.	.	.	C	.	.	.	.	-	-	-	-	-	.	.	.	.	.	.	.	.	.	-	-	-
30	*Valerianella locusta* VLO0505	.	.	.	.	.	.	.	.	.	.	C	.	.	.	.	.	.	.	.	.	.	.	.	.	.	.	.	.	G	.	.	.	.	A	.	.	.	.	.	.	.	C	.	.	.	.	-	-	-	-	-	.	.	.	.	.	.	.	.	.	-	-	-
31	*Valerianella locusta* VLO0609	.	.	.	.	.	.	.	.	.	.	C	.	.	.	.	.	.	.	.	.	.	.	.	.	.	.	.	.	G	.	.	.	.	A	.	.	.	.	.	.	.	C	.	.	.	.	-	-	-	-	-	.	.	.	.	.	.	.	.	.	-	-	-
32	*Valerianella lusitanica* VLU0102	.	.	.	.	.	.	.	.	.	.	C	.	.	.	.	.	.	.	.	.	.	.	.	.	.	.	.	.	G	.	.	.	.	A	.	.	.	.	.	.	.	C	.	.	.	.	-	-	-	-	-	.	.	.	.	.	.	.	.	.	-	-	-
33	*Valerianella microcarpa* VMI0102	.	.	.	.	.	.	.	.	.	.	C	.	.	.	.	.	.	.	.	.	.	.	.	.	.	.	.	.	A	.	.	.	.	A	.	C	.	.	.	.	.	T	.	.	.	.	-	-	-	-	-	.	.	.	.	.	.	.	.	.	.	.	.
34	*Valerianella multidentata* VMU0201	.	.	.	.	.	.	.	.	.	.	C	.	.	.	.	.	.	.	.	.	.	.	.	.	.	.	.	.	G	.	.	.	.	A	.	C	.	.	.	.	.	.	.	.	.	.	-	-	-	-	-	.	.	.	.	.	.	.	.	.	.	.	.
35	*Valerianella multidentata* VMU0301	.	.	.	.	.	.	.	.	.	.	C	.	.	.	.	.	.	.	.	.	.	.	.	.	.	.	.	.	G	.	.	.	.	A	.	C	.	.	.	.	.	.	.	.	.	.	-	-	-	-	-	.	.	.	.	.	.	.	.	.	.	.	.
36	*Valerianella pumila* VPU0103	.	.	.	.	.	.	.	.	.	.	C	.	.	.	.	.	.	.	.	.	.	.	.	.	.	.	.	.	G	.	.	.	.	A	.	C	.	.	.	.	.	.	.	.	.	.	-	-	-	-	-	.	.	.	.	.	.	.	.	.	.	.	.
37	*Valerianella pumila* VPU0304	.	.	.	.	.	.	.	.	.	.	C	.	.	.	.	.	.	.	.	.	.	.	.	.	.	.	.	.	G	.	.	.	.	A	.	C	.	.	.	.	.	.	.	.	.	.	-	-	-	-	-	.	.	.	.	.	.	.	.	.	.	.	.
38	*Valerianella pumila* VPU0402	.	.	.	.	.	.	.	.	.	.	C	.	.	.	.	.	.	.	.	.	.	.	.	.	.	.	.	.	G	.	.	.	.	A	.	C	.	.	.	.	.	.	.	.	.	.	-	-	-	-	-	.	.	.	.	.	.	.	.	.	.	.	.
39	*Valerianella rimosa* VRI0101	.	.	.	.	.	.	.	.	.	.	C	.	.	.	.	.	.	.	.	.	.	.	.	.	.	.	.	.	A	.	.	.	.	A	.	C	.	.	.	.	.	.	.	.	.	.	-	-	-	-	-	.	.	.	.	.	.	.	.	.	.	.	.
40	*Valerianella rimosa* VRI0201	.	.	.	.	.	.	.	.	.	.	C	.	.	.	.	.	.	.	.	.	.	.	.	.	.	.	.	.	A	.	.	.	.	A	.	C	.	.	.	.	.	.	.	.	.	.	-	-	-	-	-	.	.	.	.	.	.	.	.	.	.	.	.

	Position	820	821	822	823	824	825	826	827	828	829	830	831	832	833	834	835	836	837	838	839	840	841	842	843	844	845	846	847	848	849	850	851	852	853	854	855	856	857	858	859	860	861	862	863	864	865	866	867	868	869	870	871	872	873	874	875	876	877	878	879	880	881	882
1	*Centranthus calcitrapae* CC25	A	A	G	A	C	T	T	T	A	T	A	A	G	T	T	T	C	A	G	T	A	T	G	A	A	T	A	A	T	G	A	T	A	T	T	C	A	T	C	A	T	G	A	A	T	T	A	T	T	C	A	A	A	T	A	G	G	G	A	T	T	C	G
2	*Centranthus calcitrapae* CCACC539	.	.	.	.	.	.	.	.	.	.	.	.	.	.	.	.	.	.	.	.	.	.	.	.	.	.	.	.	.	.	.	.	.	.	.	.	.	.	.	.	.	.	.	.	.	.	.	.	.	.	.	.	.	.	.	.	.	.	.	.	.	.	.
3	*Fedia cornucopiae* FC73	.	.	.	.	.	.	.	.	G	.	.	.	.	.	.	.	.	.	.	.	.	.	.	.	.	.	.	.	.	.	.	.	.	.	.	.	.	.	T	.	.	.	.	.	.	.	.	.	.	.	.	T	.	.	C	.	.	.	.	.	.	.	-
4	*Valerianella carinata* VCA0104	-	-	.	.	.	.	.	.	G	.	.	.	.	.	.	.	.	.	.	.	.	.	.	.	.	.	.	.	.	.	.	.	.	.	.	.	.	.	T	.	.	.	.	.	.	.	.	.	.	.	.	T	.	.	.	A	T	.	.	.	.	.	-
5	*Valerianella carinata* VCA0202	-	-	.	.	.	.	.	.	G	.	.	.	.	.	.	.	.	.	.	.	.	.	.	.	.	.	.	.	.	.	.	.	.	.	-	-	-	-	-	-	-	.	.	.	.	.	.	.	.	.	.	T	.	.	.	A	T	.	.	.	.	.	-
6	*Valerianella carinata* VCA0302	-	-	.	.	.	.	.	.	G	.	.	.	.	.	.	.	.	.	.	.	.	.	.	.	.	.	.	.	.	.	.	.	.	.	-	-	-	-	-	-	-	.	.	.	.	.	.	.	.	.	.	T	.	.	.	A	T	.	.	.	.	.	-
7	*Valerianella carinata* VCA0501	-	-	.	.	.	.	.	.	G	.	.	.	.	.	.	.	.	.	.	.	.	.	.	.	.	.	.	.	.	.	.	.	.	.	-	-	-	-	-	-	-	.	.	.	.	.	.	.	.	.	.	T	.	.	.	A	T	.	.	.	.	.	-
8	*Valerianella carinata* VCA0805	-	-	.	.	.	.	.	.	G	.	.	.	.	.	.	.	.	.	.	.	.	.	.	.	.	.	.	.	.	.	.	.	.	.	-	-	-	-	-	-	-	.	.	.	.	.	.	.	.	.	.	T	.	.	.	A	T	.	.	.	.	.	-
9	*Valerianella carinata* VCA0918	-	-	.	.	.	.	.	.	G	.	.	.	.	.	.	.	.	.	.	.	.	.	.	.	.	.	.	.	.	.	.	.	.	.	-	-	-	-	-	-	-	.	.	.	.	.	.	.	.	.	.	T	.	.	.	A	T	.	.	.	.	.	-
10	*Valerianella carinata* VCA1006	-	-	.	.	.	.	.	.	G	.	.	.	.	.	.	.	.	.	.	.	.	.	.	.	.	.	.	.	.	.	.	.	.	.	-	-	-	-	-	-	-	.	.	.	.	.	.	.	.	.	.	T	.	.	.	A	T	.	.	.	.	.	-
11	*Valerianella carinata* VCA1106	-	-	.	.	.	.	.	.	G	.	.	.	.	.	.	.	.	.	.	.	.	.	.	.	.	.	.	.	.	.	.	.	.	.	-	-	-	-	-	-	-	.	.	.	.	.	.	.	.	.	.	T	.	.	.	A	T	.	.	.	.	.	-
12	*Valerianella carinata* VCA1202	-	-	.	.	.	.	.	.	G	.	.	.	.	.	.	.	.	.	.	.	.	.	.	.	.	.	.	.	.	.	.	.	.	.	-	-	-	-	-	-	-	.	.	.	.	.	.	.	.	.	.	T	.	.	.	A	T	.	.	.	.	.	-
13	*Valerianella coronata* VCO0102	.	.	.	.	.	.	.	.	G	.	.	.	.	.	.	.	.	.	.	.	.	.	.	.	.	.	.	.	.	.	.	.	.	.	.	.	.	.	T	T	A	.	.	-	-	-	-	-	.	.	.	T	.	-	-	-	-	-	-	-	-	.	-
14	*Valerianella coronata* VCO0218	.	.	.	.	.	.	.	.	G	.	.	.	.	.	.	.	.	.	.	.	.	.	.	.	.	.	.	.	.	.	.	.	.	.	.	.	.	.	T	T	A	.	.	-	-	-	-	-	.	.	.	T	.	-	-	-	-	-	-	-	-	.	-
15	*Valerianella coronata* VCO0404	.	.	.	.	.	.	.	.	G	.	.	.	.	.	.	.	.	.	.	.	.	.	.	.	.	.	.	.	.	.	.	.	.	.	.	.	.	.	T	T	A	.	.	-	-	-	-	-	.	.	.	T	.	-	-	-	-	-	-	-	-	.	-
16	*Valerianella coronata* VCO0508	.	.	.	.	.	.	.	.	G	.	.	.	.	.	.	.	.	.	.	.	.	.	.	.	.	.	.	.	.	.	.	.	.	.	.	.	.	.	T	T	A	.	.	-	-	-	-	-	.	.	.	T	.	-	-	-	-	-	-	-	-	.	-
17	*Valerianella coronata* VCO0705	.	.	.	.	.	.	.	.	G	.	.	.	.	.	.	.	.	.	.	.	.	.	.	.	.	.	.	.	.	.	.	.	.	.	.	.	.	.	T	T	A	.	.	-	-	-	-	-	.	.	.	T	.	-	-	-	-	-	-	-	-	.	-
18	*Valerianella coronata* VCO0805	.	.	.	.	.	.	.	.	G	.	.	.	.	.	.	.	.	.	.	.	.	.	.	.	.	.	.	.	.	.	.	.	.	.	.	.	.	.	T	T	A	.	.	-	-	-	-	-	.	.	.	T	.	-	-	-	-	-	-	-	-	.	-
19	*Valerianella coronata* VCO0901	.	.	.	.	.	.	.	.	G	.	.	.	.	.	.	.	.	.	.	.	.	.	.	.	.	.	.	.	.	.	.	.	.	.	.	.	.	.	T	T	A	.	.	-	-	-	-	-	.	.	.	T	.	-	-	-	-	-	-	-	-	.	-
20	*Valerianella dentata* VDE0303	.	.	.	.	.	.	.	.	G	.	.	.	.	.	.	.	.	.	.	.	.	.	C	.	.	.	.	.	.	.	.	.	.	.	.	.	.	.	T	T	A	.	.	-	-	-	-	-	.	.	.	T	.	-	-	-	-	-	-	-	-	.	-
21	*Valerianella discoidea* VDI0202	.	.	.	.	.	.	.	.	G	.	.	.	.	.	.	.	.	.	.	.	.	.	.	.	.	.	.	.	.	.	.	.	.	.	.	.	.	.	T	T	A	.	.	-	-	-	-	-	.	.	.	T	.	-	-	-	-	-	-	-	-	.	-
22	*Valerianella discoidea* VDI0412	.	.	.	.	.	.	.	.	G	.	.	.	.	.	.	.	.	.	.	.	.	.	.	.	.	.	.	.	.	.	.	.	.	.	.	.	.	.	T	T	A	.	.	-	-	-	-	-	.	.	.	T	.	-	-	-	-	-	-	-	-	.	-
23	*Valerianella echinata* VEC0101	.	.	.	.	.	.	.	.	G	.	.	.	.	.	.	.	.	.	.	.	.	.	.	.	.	.	.	.	.	.	.	.	.	.	.	.	.	.	T	.	.	.	.	.	.	.	.	.	.	.	.	T	.	.	.	A	T	.	.	.	G	.	-
24	*Valerianella eriocarpa* VER0208	.	.	.	.	.	.	.	.	G	.	.	.	.	.	.	.	.	.	.	.	.	.	.	.	.	.	.	.	.	.	.	.	.	.	.	.	.	.	T	T	A	.	.	-	-	-	-	-	.	.	.	T	.	-	-	-	-	-	-	-	-	.	-
25	*Valerianella eriocarpa* VER0402	.	.	.	.	.	.	.	.	G	.	.	.	.	.	.	.	.	.	.	.	.	.	.	.	.	.	.	.	.	.	.	.	.	.	.	.	.	.	T	T	A	.	.	-	-	-	-	-	.	.	.	T	.	-	-	-	-	-	-	-	-	.	-
26	*Valerianella fusiformis* VFU0103	.	.	.	.	.	.	.	.	G	.	.	.	.	.	.	.	.	.	.	.	.	.	.	.	.	.	.	.	.	.	.	.	.	.	.	.	.	.	.	.	.	.	.	.	.	.	.	.	.	.	.	T	.	.	.	.	.	.	.	.	.	.	-
27	*Valerianella locusta* VLO0103	-	-	.	.	.	.	.	.	G	.	.	.	.	.	.	.	.	.	.	.	.	.	.	.	.	.	.	.	.	.	.	.	.	.	-	-	-	-	-	-	-	.	.	.	.	.	.	.	.	.	.	T	.	.	.	A	T	.	.	.	.	.	-
28	*Valerianella locusta* VLO0305	-	-	.	.	.	.	.	.	G	.	.	.	.	.	.	.	.	.	.	.	.	.	.	.	.	.	.	.	.	.	.	.	.	.	-	-	-	-	-	-	-	.	.	.	.	.	.	.	.	.	.	T	.	.	.	A	T	.	.	.	.	.	-
29	*Valerianella locusta* VLO0409	-	-	.	.	.	.	.	.	G	.	.	.	.	.	.	.	.	.	.	.	.	.	.	.	.	.	.	.	.	.	.	.	.	.	-	-	-	-	-	-	-	.	.	.	.	.	.	.	.	.	.	T	.	.	.	A	T	.	.	.	.	.	-
30	*Valerianella locusta* VLO0505	-	-	.	.	.	.	.	.	G	.	.	.	.	.	.	.	.	.	.	.	.	.	.	.	.	.	.	.	.	.	.	.	.	.	-	-	-	-	-	-	-	.	.	.	.	.	.	.	.	.	.	T	.	.	.	A	T	.	.	.	.	.	-
31	*Valerianella locusta* VLO0609	-	-	.	.	.	.	.	.	G	.	.	.	.	.	.	.	.	.	.	.	.	.	.	.	.	.	.	.	.	.	.	.	.	.	-	-	-	-	-	-	-	.	.	.	.	.	.	.	.	.	.	T	.	.	.	A	T	.	.	.	.	.	-
32	*Valerianella lusitanica* VLU0102	-	-	.	.	.	.	.	.	G	.	.	.	.	.	.	.	.	.	.	.	.	.	.	.	.	.	.	.	.	.	.	.	.	.	.	.	.	.	T	.	.	.	.	.	.	.	.	.	.	.	.	T	.	.	.	A	T	.	.	.	.	.	-
33	*Valerianella microcarpa* VMI0102	.	.	.	.	.	.	.	.	G	.	.	.	.	.	.	.	.	.	.	.	.	.	.	.	.	.	.	.	.	.	.	.	.	.	.	.	.	.	T	T	A	.	.	-	-	-	-	-	.	.	.	T	.	-	-	-	-	-	-	-	-	.	-
34	*Valerianella multidentata* VMU0201	.	.	.	.	.	.	.	.	G	.	.	.	.	.	.	.	.	.	.	.	.	.	.	.	.	.	.	.	.	.	.	.	.	.	.	.	.	.	T	T	A	.	.	-	-	-	-	-	.	.	.	T	.	-	-	-	-	-	-	-	-	.	-
35	*Valerianella multidentata* VMU0301	.	.	.	.	.	.	.	.	G	.	.	.	.	.	.	.	.	.	.	.	.	.	.	.	.	.	.	.	.	.	.	.	.	.	.	.	.	.	T	T	A	.	.	-	-	-	-	-	.	.	.	T	.	-	-	-	-	-	-	-	-	.	-
36	*Valerianella pumila* VPU0103	.	.	.	.	.	.	.	.	G	.	.	.	.	.	.	.	.	.	.	.	.	.	.	.	.	.	.	.	.	.	.	.	.	.	.	.	.	.	T	T	A	.	.	-	-	-	-	-	.	.	.	T	.	-	-	-	-	-	-	-	-	.	-
37	*Valerianella pumila* VPU0304	.	.	.	.	.	.	.	.	G	.	.	.	.	.	.	.	.	.	.	.	.	.	.	.	.	.	.	.	.	.	.	.	.	.	.	.	.	.	T	T	A	.	.	-	-	-	-	-	.	.	.	T	.	-	-	-	-	-	-	-	-	.	-
38	*Valerianella pumila* VPU0402	.	.	.	.	.	.	.	.	G	.	.	.	.	.	.	.	.	.	.	.	.	.	.	.	.	.	.	.	.	.	.	.	.	.	.	.	.	.	T	T	A	.	.	-	-	-	-	-	.	.	.	T	.	-	-	-	-	-	-	-	-	.	-
39	*Valerianella rimosa* VRI0101	.	.	.	.	.	.	.	.	G	.	.	.	.	.	.	.	.	.	.	.	.	.	C	.	.	.	.	.	.	.	.	.	.	.	.	.	.	.	T	T	A	.	.	-	-	-	-	-	.	.	.	T	.	-	-	-	-	-	-	-	-	.	-
40	*Valerianella rimosa* VRI0201	.	.	.	.	.	.	.	.	G	.	.	.	.	.	.	.	.	.	.	.	.	.	C	.	.	.	.	.	.	.	.	.	.	.	.	.	.	.	T	T	A	.	.	-	-	-	-	-	.	.	.	T	.	-	-	-	-	-	-	-	-	.	-

Position

		8	8	8	8	8	8	8	8	8	8	8	8	8	8	8	8	8	9	9	9	9	9	9	9	9	9	9	9	9	9	9	9	9	9	9	9	9	9	9	9	9	9	9	9	9	9	9	9	9	9	9	9	9	9	9	9	9	9	9	9	9	9	9
		8	8	8	8	8	8	8	9	9	9	9	9	9	9	9	9	9	0	0	0	0	0	0	0	0	0	0	1	1	1	1	1	1	1	1	1	1	2	2	2	2	2	2	2	2	2	2	3	3	3	3	3	3	3	3	3	3	4	4	4	4	4	4
		3	4	5	6	7	8	9	0	1	2	3	4	5	6	7	8	9	0	1	2	3	4	5	6	7	8	9	0	1	2	3	4	5	6	7	8	9	0	1	2	3	4	5	6	7	8	9	0	1	2	3	4	5	6	7	8	9	0	1	2	3	4	5
1	*Centranthus calcitrapae* CC25	C	T	T	A	C	G	T	A	A	A	G	A	T	G	C	G	C	A	T	T	T	G	T	A	C	A	C	G	T	A	T	T	A	T	C	T	T	A	G	A	T	C	A	-	T	A	A	G	A	C	T	T	G	T	G	A	T	A	A	G	A	A	A
2	*Centranthus calcitrapae* CCACC539	.	.	.	.	.	.	.	.	.	.	.	.	.	.	.	.	.	.	.	.	.	.	.	.	.	.	.	.	.	.	.	.	.	.	.	.	.	.	.	.	.	.	.	-	.	.	.	.	.	.	.	.	.	.	.	.	.	.	.	.	.	.	.
3	*Fedia cornucopiae* FC73	.	.	.	.	.	.	.	.	.	.	.	.	.	.	.	T	.	G	.	.	.	.	.	.	.	.	.	.	.	.	.	.	.	.	T	.	.	.	.	.	.	.	.	-	.	.	.	.	.	.	.	.	-	-	-	-	-	.	.	.	.	.	.
4	*Valerianella carinata* VCA0104	T	.	.	.	.	.	.	.	.	.	.	.	.	.	.	T	.	.	.	.	.	.	.	.	.	.	.	.	.	C	.	.	.	.	.	.	.	G	.	.	.	.	.	A	.	.	.	.	.	.	.	.	-	-	-	-	-	.	.	.	-	.	.
5	*Valerianella carinata* VCA0202	T	.	.	.	.	.	.	.	.	.	.	.	.	.	.	T	.	.	.	.	.	.	.	.	.	.	.	.	.	C	.	.	.	.	.	.	.	G	.	.	.	.	.	A	.	.	.	.	.	.	.	.	-	-	-	-	-	.	.	.	-	.	.
6	*Valerianella carinata* VCA0302	T	.	.	.	.	.	.	.	.	.	.	.	.	.	.	T	.	.	.	.	.	.	.	.	.	.	.	.	.	C	.	.	.	.	.	.	.	G	.	.	.	.	.	A	.	.	.	.	.	.	.	.	-	-	-	-	-	.	.	.	-	.	.
7	*Valerianella carinata* VCA0501	T	.	.	.	.	.	.	.	.	.	.	.	.	.	.	T	.	.	.	.	.	.	.	.	.	.	.	.	.	C	.	.	.	.	.	.	.	G	.	.	.	.	.	A	.	.	.	.	.	-	-	-	-	-	-	-	-	-	-	-	-	-	.
8	*Valerianella carinata* VCA0805	T	.	.	.	.	.	.	.	.	.	.	.	.	.	.	T	.	.	.	.	.	.	.	.	.	.	.	.	.	C	.	.	.	.	.	.	.	G	.	.	.	.	.	A	.	.	.	.	.	.	.	.	-	-	-	-	-	.	.	.	-	.	.
9	*Valerianella carinata* VCA0918	T	.	.	.	.	.	.	.	.	.	.	.	.	.	.	T	.	.	.	.	.	.	.	.	.	.	.	.	.	C	.	.	.	.	.	.	.	G	.	.	.	.	.	A	.	.	.	.	.	.	.	.	-	-	-	-	-	.	.	.	-	.	.
10	*Valerianella carinata* VCA1006	T	.	.	.	.	.	.	.	.	.	.	.	.	.	.	T	.	.	.	.	.	.	.	.	.	.	.	.	.	C	.	.	.	.	.	.	.	G	.	.	.	.	.	A	.	.	.	.	.	-	-	-	-	-	-	-	-	-	-	-	-	-	.
11	*Valerianella carinata* VCA1106	T	.	.	.	.	.	.	.	.	.	.	.	.	.	.	T	.	.	.	.	.	.	.	.	.	.	.	.	.	C	.	.	.	.	.	.	.	G	.	.	.	.	.	A	.	.	.	.	.	.	.	.	-	-	-	-	-	.	.	.	-	.	.
12	*Valerianella carinata* VCA1202	T	.	.	.	.	.	.	.	.	.	.	.	.	.	.	T	.	.	.	.	.	.	.	.	.	.	.	.	.	C	.	.	.	.	.	.	.	G	.	.	.	.	.	A	.	.	.	.	.	.	.	.	-	-	-	-	-	.	.	.	-	.	.
13	*Valerianella coronata* VCO0102	-	-	-	-	-	-	-	-	-	-	-	-	-	-	-	-	-	-	-	-	-	-	-	-	-	-	-	-	-	-	-	-	-	-	-	-	-	-	-	-	-	-	-	-	-	-	-	.	.	.	.	.	-	-	-	-	-	.	.	.	.	.	.
14	*Valerianella coronata* VCO0218	-	-	-	-	-	-	-	-	-	-	-	-	-	-	-	-	-	-	-	-	-	-	-	-	-	-	-	-	-	-	-	-	-	-	-	-	-	-	-	-	-	-	-	-	-	-	-	.	.	.	.	.	-	-	-	-	-	.	.	.	.	.	.
15	*Valerianella coronata* VCO0404	-	-	-	-	-	-	-	-	-	-	-	-	-	-	-	-	-	-	-	-	-	-	-	-	-	-	-	-	-	-	-	-	-	-	-	-	-	-	-	-	-	-	-	-	-	-	-	.	.	.	.	.	-	-	-	-	-	.	.	.	.	.	.
16	*Valerianella coronata* VCO0508	-	-	-	-	-	-	-	-	-	-	-	-	-	-	-	-	-	-	-	-	-	-	-	-	-	-	-	-	-	-	-	-	-	-	-	-	-	-	-	-	-	-	-	-	-	-	-	.	.	.	.	.	-	-	-	-	-	.	.	.	.	.	.
17	*Valerianella coronata* VCO0705	-	-	-	-	-	-	-	-	-	-	-	-	-	-	-	-	-	-	-	-	-	-	-	-	-	-	-	-	-	-	-	-	-	-	-	-	-	-	-	-	-	-	-	-	-	-	-	.	.	.	.	.	-	-	-	-	-	.	.	.	.	.	.
18	*Valerianella coronata* VCO0805	-	-	-	-	-	-	-	-	-	-	-	-	-	-	-	-	-	-	-	-	-	-	-	-	-	-	-	-	-	-	-	-	-	-	-	-	-	-	-	-	-	-	-	-	-	-	-	.	.	.	.	.	-	-	-	-	-	.	.	.	.	.	.
19	*Valerianella coronata* VCO0901	-	-	-	-	-	-	-	-	-	-	-	-	-	-	-	-	-	-	-	-	-	-	-	-	-	-	-	-	-	-	-	-	-	-	-	-	-	-	-	-	-	-	-	-	-	-	-	.	.	.	.	.	-	-	-	-	-	.	.	.	.	.	.
20	*Valerianella dentata* VDE0303	-	-	-	-	-	-	-	-	-	-	-	-	-	-	-	-	-	-	-	-	-	-	-	-	-	-	-	-	-	-	-	-	-	-	-	-	-	-	-	-	-	-	-	-	-	-	-	.	.	.	.	.	-	-	-	-	-	.	.	.	.	.	.
21	*Valerianella discoidea* VDI0202	-	-	-	-	-	-	-	-	-	-	-	-	-	-	-	-	-	-	-	-	-	-	-	-	-	-	-	-	-	-	-	-	-	-	-	-	-	-	-	-	-	-	-	-	-	-	-	.	.	.	.	.	-	-	-	-	-	.	.	.	.	.	.
22	*Valerianella discoidea* VDI0412	-	-	-	-	-	-	-	-	-	-	-	-	-	-	-	-	-	-	-	-	-	-	-	-	-	-	-	-	-	-	-	-	-	-	-	-	-	-	-	-	-	-	-	-	-	-	-	.	.	.	.	.	-	-	-	-	-	.	.	.	.	.	.
23	*Valerianella echinata* VEC0101	.	.	.	.	.	.	.	.	.	.	.	.	.	.	.	T	.	.	.	.	.	.	.	.	.	.	.	.	.	C	.	.	.	.	.	.	.	.	.	.	.	-	.	A	.	.	.	.	.	.	-	.	-	-	-	-	-	.	.	-	.	.	.
24	*Valerianella eriocarpa* VER0208	-	-	-	-	-	-	-	-	-	-	-	-	-	-	-	-	-	-	-	-	-	-	-	-	-	-	-	-	-	-	-	-	-	-	-	-	-	-	-	-	-	-	-	-	-	-	-	.	.	.	.	.	-	-	-	-	-	.	.	.	.	.	.
25	*Valerianella eriocarpa* VER0402	-	-	-	-	-	-	-	-	-	-	-	-	-	-	-	-	-	-	-	-	-	-	-	-	-	-	-	-	-	-	-	-	-	-	-	-	-	-	-	-	-	-	-	-	-	-	-	.	.	.	.	.	-	-	-	-	-	.	.	.	.	.	.
26	*Valerianella fusiformis* VFU0103	.	.	.	.	.	.	G	.	.	.	.	.	.	.	.	T	.	.	.	.	.	.	.	.	.	.	.	.	.	.	.	.	.	.	.	.	.	.	.	.	.	.	.	-	.	.	C	.	.	.	.	-	-	-	A	.	A	.	.	A	.	.	.
27	*Valerianella locusta* VLO0103	T	.	.	.	.	.	.	.	.	.	.	.	.	.	.	T	.	.	.	.	.	.	.	.	.	.	.	.	.	C	.	.	.	.	.	.	.	G	.	.	.	.	.	A	.	.	.	.	.	.	.	.	-	-	-	-	-	.	.	.	-	.	.
28	*Valerianella locusta* VLO0305	T	.	.	.	.	.	.	.	.	.	.	.	.	.	.	T	.	.	.	.	.	.	.	.	.	.	.	.	.	C	.	.	.	.	.	.	.	G	.	.	.	.	.	A	.	.	.	.	.	.	.	.	-	-	-	-	-	.	.	.	-	.	.
29	*Valerianella locusta* VLO0409	T	.	.	.	.	.	.	.	.	.	.	.	.	.	.	T	.	.	.	.	.	.	.	.	.	.	.	.	.	C	.	.	.	.	.	.	.	G	.	.	.	.	.	A	.	.	.	.	.	.	.	.	-	-	-	-	-	.	.	.	-	.	.
30	*Valerianella locusta* VLO0505	T	.	.	.	.	.	.	.	.	.	.	.	.	.	.	T	.	.	.	.	.	.	.	.	.	.	.	.	.	C	.	.	.	.	.	.	.	G	.	.	.	.	.	A	.	.	.	.	.	.	.	.	-	-	-	-	-	.	.	.	-	.	.
31	*Valerianella locusta* VLO0609	T	.	.	.	.	.	.	.	.	.	.	.	.	.	.	T	.	.	.	.	.	.	.	.	.	.	.	.	.	C	.	.	.	.	.	.	.	G	.	.	.	.	.	A	.	.	.	.	.	.	.	.	-	-	-	-	-	.	.	.	-	.	.
32	*Valerianella lusitanica* VLU0102	T	.	.	.	.	.	.	.	.	.	.	.	.	.	.	T	.	.	.	.	.	.	.	.	.	.	.	.	.	C	.	.	.	.	.	.	.	G	.	.	.	.	.	A	.	.	.	.	.	.	.	.	-	-	-	-	-	.	.	.	-	.	.
33	*Valerianella microcarpa* VMI0102	-	-	-	-	-	-	-	-	-	-	-	-	-	-	-	-	-	-	-	-	-	-	-	-	-	-	-	-	-	-	-	-	-	-	-	-	-	-	-	-	-	-	-	-	-	-	-	.	.	.	.	.	-	-	-	-	-	.	.	.	.	.	.
34	*Valerianella multidentata* VMU0201	-	-	-	-	-	-	-	-	-	-	-	-	-	-	-	-	-	-	-	-	-	-	-	-	-	-	-	-	-	-	-	-	-	-	-	-	-	-	-	-	-	-	-	-	-	-	-	.	.	.	.	.	-	-	-	-	-	.	.	.	.	.	.
35	*Valerianella multidentata* VMU0301	-	-	-	-	-	-	-	-	-	-	-	-	-	-	-	-	-	-	-	-	-	-	-	-	-	-	-	-	-	-	-	-	-	-	-	-	-	-	-	-	-	-	-	-	-	-	-	.	.	.	.	.	-	-	-	-	-	.	.	.	.	.	.
36	*Valerianella pumila* VPU0103	-	-	-	-	-	-	-	-	-	-	-	-	-	-	-	-	-	-	-	-	-	-	-	-	-	-	-	-	-	-	-	-	-	-	-	-	-	-	-	-	-	-	-	-	-	-	-	.	.	.	.	.	-	-	-	-	-	.	.	.	.	.	.
37	*Valerianella pumila* VPU0304	-	-	-	-	-	-	-	-	-	-	-	-	-	-	-	-	-	-	-	-	-	-	-	-	-	-	-	-	-	-	-	-	-	-	-	-	-	-	-	-	-	-	-	-	-	-	-	.	.	.	.	.	-	-	-	-	-	.	.	.	.	.	.
38	*Valerianella pumila* VPU0402	-	-	-	-	-	-	-	-	-	-	-	-	-	-	-	-	-	-	-	-	-	-	-	-	-	-	-	-	-	-	-	-	-	-	-	-	-	-	-	-	-	-	-	-	-	-	-	.	.	.	.	.	-	-	-	-	-	.	.	.	.	.	.
39	*Valerianella rimosa* VRI0101	-	-	-	-	-	-	-	-	-	-	-	-	-	-	-	-	-	-	-	-	-	-	-	-	-	-	-	-	-	-	-	-	-	-	-	-	-	-	-	-	-	-	-	-	-	-	-	.	.	.	.	.	-	-	-	-	-	.	.	.	.	.	.
40	*Valerianella rimosa* VRI0201	-	-	-	-	-	-	-	-	-	-	-	-	-	-	-	-	-	-	-	-	-	-	-	-	-	-	-	-	-	-	-	-	-	-	-	-	-	-	-	-	-	-	-	-	-	-	-	.	.	.	.	.	-	-	-	-	-	.	.	.	.	.	.

Position

		946	947	948	949	950	951	952	953	954	955	956	957	958	959	960	961	962	963	964	965	966	967	968	969	970	971	972	973	974	975	976	977	978	979	980	981	982	983	984	985	986	987	988	989	990	991	992	993	994	995	996	997	998	999	1000	1001	1002	1003	1004	1005	1006	1007	1008
1	*Centranthus calcitrapae* CC25	A	A	A	A	C	A	T	T	T	T	C	A	C	T	C	G	G	A	T	C	T	C	T	T	T	G	T	A	A	A	A	G	A	T	A	A	A	A	G	A	G	T	A	G	G	G	T	G	A	A	T	G	A	G	A	A	A	G	A	T	A	A	C
2	*Centranthus calcitrapae* CCACC539	.	.	.	.	.	.	.	.	.	.	.	.	.	.	.	.	.	.	.	.	.	.	.	.	.	.	.	.	.	.	.	.	.	.	.	.	.	.	.	.	.	.	.	.	.	.	.	.	.	.	.	.	.	.	.	.	.	.	.	.	.	.	.
3	*Fedia cornucopiae* FC73	.	.	.	.	T	.	.	.	.	.	.	.	.	.	-	-	-	-	-	-	-	-	-	-	-	-	-	-	-	-	-	-	-	-	-	-	-	-	-	-	-	-	-	-	-	-	-	-	-	-	-	-	-	-	-	-	-	-	-	-	-	-	-
4	*Valerianella carinata* VCA0104	.	.	.	.	G	.	-	-	-	-	-	-	-	-	-	-	-	-	-	-	-	-	-	-	-	-	-	-	-	-	-	-	-	-	-	-	-	-	-	-	-	-	-	-	-	-	-	-	-	-	-	-	-	-	-	-	-	-	-	-	-	-	-
5	*Valerianella carinata* VCA0202	.	.	.	.	G	.	-	-	-	-	-	-	-	-	-	-	-	-	-	-	-	-	-	-	-	-	-	-	-	-	-	-	-	-	-	-	-	-	-	-	-	-	-	-	-	-	-	-	-	-	-	-	-	-	-	-	-	-	-	-	-	-	-
6	*Valerianella carinata* VCA0302	.	.	.	.	G	.	-	-	-	-	-	-	-	-	-	-	-	-	-	-	-	-	-	-	-	-	-	-	-	-	-	-	-	-	-	-	-	-	-	-	-	-	-	-	-	-	-	-	-	-	-	-	-	-	-	-	-	-	-	-	-	-	-
7	*Valerianella carinata* VCA0501	.	.	.	.	G	.	-	-	-	-	-	-	-	-	-	-	-	-	-	-	-	-	-	-	-	-	-	-	-	-	-	-	-	-	-	-	-	-	-	-	-	-	-	-	-	-	-	-	-	-	-	-	-	-	-	-	-	-	-	-	-	-	-
8	*Valerianella carinata* VCA0805	.	.	.	.	G	.	-	-	-	-	-	-	-	-	-	-	-	-	-	-	-	-	-	-	-	-	-	-	-	-	-	-	-	-	-	-	-	-	-	-	-	-	-	-	-	-	-	-	-	-	-	-	-	-	-	-	-	-	-	-	-	-	-
9	*Valerianella carinata* VCA0918	.	.	.	.	G	.	-	-	-	-	-	-	-	-	-	-	-	-	-	-	-	-	-	-	-	-	-	-	-	-	-	-	-	-	-	-	-	-	-	-	-	-	-	-	-	-	-	-	-	-	-	-	-	-	-	-	-	-	-	-	-	-	-
10	*Valerianella carinata* VCA1006	.	.	.	.	G	.	-	-	-	-	-	-	-	-	-	-	-	-	-	-	-	-	-	-	-	-	-	-	-	-	-	-	-	-	-	-	-	-	-	-	-	-	-	-	-	-	-	-	-	-	-	-	-	-	-	-	-	-	-	-	-	-	-
11	*Valerianella carinata* VCA1106	.	.	.	.	G	.	-	-	-	-	-	-	-	-	-	-	-	-	-	-	-	-	-	-	-	-	-	-	-	-	-	-	-	-	-	-	-	-	-	-	-	-	-	-	-	-	-	-	-	-	-	-	-	-	-	-	-	-	-	-	-	-	-
12	*Valerianella carinata* VCA1202	.	.	.	.	G	.	-	-	-	-	-	-	-	-	-	-	-	-	-	-	-	-	-	-	-	-	-	-	-	-	-	-	-	-	-	-	-	-	-	-	-	-	-	-	-	-	-	-	-	-	-	-	-	-	-	-	-	-	-	-	-	-	-
13	*Valerianella coronata* VCO0102	.	.	.	.	G	.	.	.	.	.	.	.	.	.	-	-	-	-	-	-	-	-	-	-	-	-	-	-	-	-	-	-	-	-	-	-	-	-	-	-	-	-	-	-	-	-	-	-	-	-	-	-	-	-	-	-	-	-	-	-	-	-	-
14	*Valerianella coronata* VCO0218	.	.	.	.	G	.	.	.	.	.	.	.	.	.	-	-	-	-	-	-	-	-	-	-	-	-	-	-	-	-	-	-	-	-	-	-	-	-	-	-	-	-	-	-	-	-	-	-	-	-	-	-	-	-	-	-	-	-	-	-	-	-	-
15	*Valerianella coronata* VCO0404	.	.	.	.	G	.	.	.	.	.	.	.	.	.	-	-	-	-	-	-	-	-	-	-	-	-	-	-	-	-	-	-	-	-	-	-	-	-	-	-	-	-	-	-	-	-	-	-	-	-	-	-	-	-	-	-	-	-	-	-	-	-	-
16	*Valerianella coronata* VCO0508	.	.	.	.	G	.	.	.	.	.	.	.	.	.	-	-	-	-	-	-	-	-	-	-	-	-	-	-	-	-	-	-	-	-	-	-	-	-	-	-	-	-	-	-	-	-	-	-	-	-	-	-	-	-	-	-	-	-	-	-	-	-	-
17	*Valerianella coronata* VCO0705	.	.	.	.	G	.	.	.	.	.	.	.	.	.	-	-	-	-	-	-	-	-	-	-	-	-	-	-	-	-	-	-	-	-	-	-	-	-	-	-	-	-	-	-	-	-	-	-	-	-	-	-	-	-	-	-	-	-	-	-	-	-	-
18	*Valerianella coronata* VCO0805	.	.	.	.	G	.	.	.	.	.	.	.	.	.	-	-	-	-	-	-	-	-	-	-	-	-	-	-	-	-	-	-	-	-	-	-	-	-	-	-	-	-	-	-	-	-	-	-	-	-	-	-	-	-	-	-	-	-	-	-	-	-	-
19	*Valerianella coronata* VCO0901	.	.	.	.	G	.	.	.	.	.	.	.	.	.	-	-	-	-	-	-	-	-	-	-	-	-	-	-	-	-	-	-	-	-	-	-	-	-	-	-	-	-	-	-	-	-	-	-	-	-	-	-	-	-	-	-	-	-	-	-	-	-	-
20	*Valerianella dentata* VDE0303	.	.	.	.	G	.	.	.	.	.	.	.	.	.	-	-	-	-	-	-	-	-	-	-	-	-	-	-	-	-	-	-	-	-	-	-	-	-	-	-	-	-	-	-	-	-	-	-	-	-	-	-	-	-	-	-	-	-	-	-	-	-	-
21	*Valerianella discoidea* VDI0202	.	.	.	.	G	.	.	.	.	.	.	.	.	.	-	-	-	-	-	-	-	-	-	-	-	-	-	-	-	-	-	-	-	-	-	-	-	-	-	-	-	-	-	-	-	-	-	-	-	-	-	-	-	-	-	-	-	-	-	-	-	-	-
22	*Valerianella discoidea* VDI0412	.	.	.	.	G	.	.	.	.	.	.	.	.	.	-	-	-	-	-	-	-	-	-	-	-	-	-	-	-	-	-	-	-	-	-	-	-	-	-	-	-	-	-	-	-	-	-	-	-	-	-	-	-	-	-	-	-	-	-	-	-	-	-
23	*Valerianella echinata* VEC0101	.	.	.	.	G	.	.	.	.	.	.	.	.	.	-	-	-	-	-	-	-	-	-	-	-	-	-	-	-	-	-	-	-	-	-	-	-	-	-	-	-	-	-	-	-	-	-	-	-	-	-	-	-	-	-	-	-	-	-	-	-	-	-
24	*Valerianella eriocarpa* VER0208	.	.	.	.	G	.	.	.	.	.	.	.	.	.	-	-	-	-	-	-	-	-	-	-	-	-	-	-	-	-	-	-	-	-	-	-	-	-	-	-	-	-	-	-	-	-	-	-	-	-	-	-	-	-	-	-	-	-	-	-	-	-	-
25	*Valerianella eriocarpa* VER0402	.	.	.	.	G	.	.	.	.	.	.	.	.	.	-	-	-	-	-	-	-	-	-	-	-	-	-	-	-	-	-	-	-	-	-	-	-	-	-	-	-	-	-	-	-	-	-	-	-	-	-	-	-	-	-	-	-	-	-	-	-	-	-
26	*Valerianella fusiformis* VFU0103	C	.	C	.	G	.	.	.	.	.	.	G	.	-	-	-	-	-	-	-	-	-	-	-	-	-	-	-	-	-	-	-	-	-	-	-	-	-	-	-	-	-	-	-	-	-	-	-	-	-	-	-	-	-	-	-	-	-	-	-	-	-	-
27	*Valerianella locusta* VLO0103	.	.	.	.	G	.	-	-	-	-	-	-	-	-	-	-	-	-	-	-	-	-	-	-	-	-	-	-	-	-	-	-	-	-	-	-	-	-	-	-	-	-	-	-	-	-	-	-	-	-	-	-	-	-	-	-	-	-	-	-	-	-	-
28	*Valerianella locusta* VLO0305	.	.	.	.	G	.	-	-	-	-	-	-	-	-	-	-	-	-	-	-	-	-	-	-	-	-	-	-	-	-	-	-	-	-	-	-	-	-	-	-	-	-	-	-	-	-	-	-	-	-	-	-	-	-	-	-	-	-	-	-	-	-	-
29	*Valerianella locusta* VLO0409	.	.	.	.	G	.	-	-	-	-	-	-	-	-	-	-	-	-	-	-	-	-	-	-	-	-	-	-	-	-	-	-	-	-	-	-	-	-	-	-	-	-	-	-	-	-	-	-	-	-	-	-	-	-	-	-	-	-	-	-	-	-	-
30	*Valerianella locusta* VLO0505	.	.	.	.	G	.	-	-	-	-	-	-	-	-	-	-	-	-	-	-	-	-	-	-	-	-	-	-	-	-	-	-	-	-	-	-	-	-	-	-	-	-	-	-	-	-	-	-	-	-	-	-	-	-	-	-	-	-	-	-	-	-	-
31	*Valerianella locusta* VLO0609	.	.	.	.	G	.	-	-	-	-	-	-	-	-	-	-	-	-	-	-	-	-	-	-	-	-	-	-	-	-	-	-	-	-	-	-	-	-	-	-	-	-	-	-	-	-	-	-	-	-	-	-	-	-	-	-	-	-	-	-	-	-	-
32	*Valerianella lusitanica* VLU0102	.	.	.	.	G	.	-	-	-	-	-	-	-	-	-	-	-	-	-	-	-	-	-	-	-	-	-	-	-	-	-	-	-	-	-	-	-	-	-	-	-	-	-	-	-	-	-	-	-	-	-	-	-	-	-	-	-	-	-	-	-	-	-
33	*Valerianella microcarpa* VMI0102	.	.	.	.	G	.	-	-	-	-	-	-	-	-	-	-	-	-	-	-	-	-	-	-	-	-	-	-	-	-	-	-	-	-	-	-	-	-	-	-	-	-	-	-	-	-	-	-	-	-	-	-	-	-	-	-	-	-	-	-	-	-	-
34	*Valerianella multidentata* VMU0201	.	.	.	.	G	.	-	-	-	-	-	-	-	-	-	-	-	-	-	-	-	-	-	-	-	-	-	-	-	-	-	-	-	-	-	-	-	-	-	-	-	-	-	-	-	-	-	-	-	-	-	-	-	-	-	-	-	-	-	-	-	-	-
35	*Valerianella multidentata* VMU0301	.	.	.	.	G	.	-	-	-	-	-	-	-	-	-	-	-	-	-	-	-	-	-	-	-	-	-	-	-	-	-	-	-	-	-	-	-	-	-	-	-	-	-	-	-	-	-	-	-	-	-	-	-	-	-	-	-	-	-	-	-	-	-
36	*Valerianella pumila* VPU0103	.	.	.	.	G	.	-	-	-	-	-	-	-	-	-	-	-	-	-	-	-	-	-	-	-	-	-	-	-	-	-	-	-	-	-	-	-	-	-	-	-	-	-	-	-	-	-	-	-	-	-	-	-	-	-	-	-	-	-	-	-	-	-
37	*Valerianella pumila* VPU0304	.	.	.	.	G	.	-	-	-	-	-	-	-	-	-	-	-	-	-	-	-	-	-	-	-	-	-	-	-	-	-	-	-	-	-	-	-	-	-	-	-	-	-	-	-	-	-	-	-	-	-	-	-	-	-	-	-	-	-	-	-	-	-
38	*Valerianella pumila* VPU0402	.	.	.	.	G	.	-	-	-	-	-	-	-	-	-	-	-	-	-	-	-	-	-	-	-	-	-	-	-	-	-	-	-	-	-	-	-	-	-	-	-	-	-	-	-	-	-	-	-	-	-	-	-	-	-	-	-	-	-	-	-	-	-
39	*Valerianella rimosa* VRI0101	.	.	.	.	G	.	-	-	-	-	-	-	-	-	-	-	-	-	-	-	-	-	-	-	-	-	-	-	-	-	-	-	-	-	-	-	-	-	-	-	-	-	-	-	-	-	-	-	-	-	-	-	-	-	-	-	-	-	-	-	-	-	-
40	*Valerianella rimosa* VRI0201	.	.	.	.	G	.	-	-	-	-	-	-	-	-	-	-	-	-	-	-	-	-	-	-	-	-	-	-	-	-	-	-	-	-	-	-	-	-	-	-	-	-	-	-	-	-	-	-	-	-	-	-	-	-	-	-	-	-	-	-	-	-	-

		Position
		1 1 1 1 1 1 1 1 1 1 1 1 1 1 1 1 1 1 1 1 1 1 1 1 1 1 1 1 1 1 1 1 1 1 1 1 1 1 1 1 1 1 1 1 1 1 1 1 1 1 1 1 1 1 1 1 1 1 1 1 1 1 1
		0 0 0 0 0 0 0 0 0 0 0 0 0 0 0 0 0 0 0 0 0 0 0 0 0 0 0 0 0 0 0 0 0 0 0 0 0 0 0 0 0 0 0 0 0 0 0 0 0 0 0 0 0 0 0 0 0 0 0 0 0 0 0
		0 1 1 1 1 1 1 1 1 1 1 2 2 2 2 2 2 2 2 2 2 3 3 3 3 3 3 3 3 3 3 4 4 4 4 4 4 4 4 4 4 5 5 5 5 5 5 5 5 5 5 6 6 6 6 6 6 6 6 6 6 7 7
		9 0 1 2 3 4 5 6 7 8 9 0 1 2 3 4 5 6 7 8 9 0 1 2 3 4 5 6 7 8 9 0 1 2 3 4 5 6 7 8 9 0 1 2 3 4 5 6 7 8 9 0 1 2 3 4 5 6 7 8 9 0 1
1	*Centranthus calcitrapae* CC25	T C C G C T A A T A A T A A G A A A A G G A T A A A T A A T T G A G G A G T C C A T A T - G G T T T T T T T G G G G A T A G A
2	*Centranthus calcitrapae* CCACC539	. . . . - . . . .
3	*Fedia cornucopiae* FC73	- - - - - - - - - - - - . . A C . . . - . C . . . . A G . . . C . . . . A . . - - . . . .
4	*Valerianella carinata* VCA0104	- - - - - - - - - - - - . . A . . . - - . C . . T . . . . . . A G . . . C . . . . A . . - - . . . .
5	*Valerianella carinata* VCA0202	- - - - - - - - - - - - . . A . . . - - . C . . T . . . . . . A G . . . C . . . . A . . - - . . . .
6	*Valerianella carinata* VCA0302	- - - - - - - - - - - - . . A . . . - - . C . . T . . . . . . A G . . . C . . . . A . . - - . . . .
7	*Valerianella carinata* VCA0501	- - - - - - - - - - - - . . A . . . - - . C . . T . . . . . . A G . . . C . . . . A . . - - . . . .
8	*Valerianella carinata* VCA0805	- - - - - - - - - - - - . . A . . . - - . C . . T . . . . . . A G . . . C . . . . A . . - - . . . .
9	*Valerianella carinata* VCA0918	- - - - - - - - - - - - . . A . . . - - . C . . T . . . . . . A G . . . C . . . . A . . - - . . . .
10	*Valerianella carinata* VCA1006	- - - - - - - - - - - - . . A . . . - - . C . . T . . . . . . A G . . . C . . . . A . . - - . . . .
11	*Valerianella carinata* VCA1106	- - - - - - - - - - - - . . A . . . - - . C . . T . . . . . . A G . . . C . . . . A . . - - . . . .
12	*Valerianella carinata* VCA1202	- - - - - - - - - - - - . . A . . . - - . C . . T . . . . . . A G . . . C . . . . A . . - - . . . .
13	*Valerianella coronata* VCO0102	- - - - - - - - - - - - . . C . . . . . . C . . . . A G . T . C . . . . A . . - - . . . .
14	*Valerianella coronata* VCO0218	- - - - - - - - - - - - . . C . . . . . . C . . . . A G . T . C . . . . A . . - - . . . .
15	*Valerianella coronata* VCO0404	- - - - - - - - - - - - . . C . . . . . . C . . . . A G . T . C . . . . A . . - - . . . .
16	*Valerianella coronata* VCO0508	- - - - - - - - - - - - . . C . . . . . . C . . . . A G . T . C . . . . A . . - - . . . .
17	*Valerianella coronata* VCO0705	- - - - - - - - - - - - . . C . . . . . . C . . . . A G . T . C . . . . A . . - - . . . .
18	*Valerianella coronata* VCO0805	- - - - - - - - - - - - . . C . . . . . . C . . . . A G . T . C . . . . A . . - - . . . .
19	*Valerianella coronata* VCO0901	- - - - - - - - - - - - . . C . . . . . . C . . . . A G . T . C . . . . A . . - - . . . .
20	*Valerianella dentata* VDE0303	- - - - - - - - - - - - . . C . . . . . . C . . . . A G . T . C . . . . A . . - - . . . .
21	*Valerianella discoidea* VDI0202	- - - - - - - - - - - - . . C . . . . . . C . . . . A G . T . C . . . . A . . - - . . . .
22	*Valerianella discoidea* VDI0412	- - - - - - - - - - - - . . C . . . . . . C . . . . A G . T . C . . . . A . . - - . . . .
23	*Valerianella echinata* VEC0101	- - - - - - - - - - - - . . A . . . . - . C . . . . A G . A . C . . . . A . . - - . . . .
24	*Valerianella eriocarpa* VER0208	- - - - - - - - - - - - . . C . . . . . . C . . . . A G . T . C . . . . A . . - - . . . .
25	*Valerianella eriocarpa* VER0402	- - - - - - - - - - - - . . C . . . . . . C . . . . A G . T . C . . . . A . . - - . . . .
26	*Valerianella fusiformis* VFU0103	- - - - - - - - - - - - C . A . . . G . . C . . . . A G . . . C . . A . . . . A . . - - . . . .
27	*Valerianella locusta* VLO0103	- - - - - - - - - - - - . . A . . . - - . C . . T . . . . . . A G . . . C . . . . A . . - - . . . .
28	*Valerianella locusta* VLO0305	- - - - - - - - - - - - . . A . . . - - . C . . T . . . . . . A G . . . C . . . . A . . - - . . . .
29	*Valerianella locusta* VLO0409	- - - - - - - - - - - - . . A . . . - - . C . . T . . . . . . A G . . . C . . . . A . . - - . . . .
30	*Valerianella locusta* VLO0505	- - - - - - - - - - - - . . A . . . - - . C . . T . . . . . . A G . . . C . . . . A . . - - . . . .
31	*Valerianella locusta* VLO0609	- - - - - - - - - - - - . . A . . . - - . C . . T . . . . . . A G . . . C . . . . A . . - - . . . .
32	*Valerianella lusitanica* VLU0102	- - - - - - - - - - - - . . A . . . - - . C . . T . . . . . . A G . . . C . . . . A . . - - . . . .
33	*Valerianella microcarpa* VMI0102	- - - - - - - - - - - - . . C . . . . . . C . . . . A G . T . C . . . . A . . - - . . . .
34	*Valerianella multidentata* VMU0201	- - - - - - - - - - - - . . C . . . . . . C . . . . A G . T . C . . . . A . . - - . . . .
35	*Valerianella multidentata* VMU0301	- - - - - - - - - - - - . . C . . . . . . C . . . . A G . T . C . . . . A . . - - . . . .
36	*Valerianella pumila* VPU0103	- - - - - - - - - - - - . . C . . . . . . C . . . . A G . T . C . . . . A . . - - . . . .
37	*Valerianella pumila* VPU0304	- - - - - - - - - - - - . . C . . . . . . C . . . . A G . T . C . . . . A . . - - . . . .
38	*Valerianella pumila* VPU0402	- - - - - - - - - - - - . . C . . . . . . C . . . . A G . T . C . . . . A . . - - . . . .
39	*Valerianella rimosa* VRI0101	- - - - - - - - - - - - . . C . . . . . . C . . . . A G . T . C . . . . A . . - - . . . .
40	*Valerianella rimosa* VRI0201	- - - - - - - - - - - - . . C . . . . . . C . . . . A G . T . C . . . . A . . - - . . . .

#	Taxon	1072	1073	1074	1075	1076	1077	1078	1079	1080	1081	1082	1083	1084	1085	1086	1087	1088	1089	1090	1091	1092	1093	1094	1095	1096	1097	1098	1099	1100	1101	1102	1103	1104	1105	1106	1107	1108	1109	1110	1111	1112	1113	1114	1115	1116	1117	1118	1119	1120	1121	1122	1123	1124	1125	1126	1127	1128	1129	1130	1131	1132	1133	1134
1	*Centranthus calcitrapae* CC25	G	G	G	A	C	T	T	G	A	A	C	C	C	T	C	A	C	G	A	T	T	T	C	T	A	A	A	G	T	C	G	A	C	G	G	A	T	T	T	T	C	C	T	C	T	T	A	C	T	C	T	A	A	A	T	T	T	C	A	T	T	G	T
2	*Centranthus calcitrapae* CCACC539	.	.	.	.	.	.	.	.	.	.	.	.	.	.	.	.	.	.	.	.	.	.	.	.	.	.	.	.	.	.	.	.	.	.	.	.	.	.	.	.	.	.	.	.	.	.	.	.	.	.	.	.	.	.	.	.	.	.	.	.	.	.	.
3	*Fedia cornucopiae* FC73	.	.	.	.	.	.	.	.	.	.	.	.	.	.	.	.	.	.	.	.	.	.	.	A	.	.	.	A	.	.	.	.	.	.	.	.	.	.	.	.	.	.	.	.	.	.	.	.	.	.	.	.	.	.	.	.	.	.	.	.	.	T	.
4	*Valerianella carinata* VCA0104	.	.	.	.	.	.	.	.	.	.	.	.	.	.	.	.	.	.	.	.	.	.	.	A	.	.	.	A	.	.	.	.	.	.	.	.	.	.	.	.	.	.	.	.	.	.	.	.	.	.	.	.	.	.	.	.	.	.	.	.	.	.	.
5	*Valerianella carinata* VCA0202	.	.	.	.	.	.	.	.	.	.	.	.	.	.	.	.	.	.	.	.	.	.	.	A	.	.	.	A	.	.	.	.	.	.	.	.	.	.	.	.	.	.	.	.	.	.	.	.	.	.	.	.	.	.	.	.	.	.	.	.	.	.	.
6	*Valerianella carinata* VCA0302	.	.	.	.	.	.	.	.	.	.	.	.	.	.	.	.	.	.	.	.	.	.	.	A	.	.	.	A	.	.	.	.	.	.	.	.	.	.	.	.	.	.	.	.	.	.	.	.	.	.	.	.	.	.	.	.	.	.	.	.	.	.	.
7	*Valerianella carinata* VCA0501	.	.	.	.	.	.	.	.	.	.	.	.	.	.	.	.	.	.	.	.	.	.	.	A	.	.	.	A	.	.	.	.	.	.	.	.	.	.	.	.	.	.	.	.	.	.	.	.	.	.	.	.	.	.	.	.	.	.	.	.	.	.	.
8	*Valerianella carinata* VCA0805	.	.	.	.	.	.	.	.	.	.	.	.	.	.	.	.	.	.	.	.	.	.	.	A	.	.	.	A	.	.	.	.	.	.	.	.	.	.	.	.	.	.	.	.	.	.	.	.	.	.	.	.	.	.	.	.	.	.	.	.	.	.	.
9	*Valerianella carinata* VCA0918	.	.	.	.	.	.	.	.	.	.	.	.	.	.	.	.	.	.	.	.	.	.	.	A	.	.	.	A	.	.	.	.	.	.	.	.	.	.	.	.	.	.	.	.	.	.	.	.	.	.	.	.	.	.	.	.	.	.	.	.	.	.	.
10	*Valerianella carinata* VCA1006	.	.	.	.	.	.	.	.	.	.	.	.	.	.	.	.	.	.	.	.	.	.	.	A	.	.	.	A	.	.	.	.	.	.	.	.	.	.	.	.	.	.	.	.	.	.	.	.	.	.	.	.	.	.	.	.	.	.	.	.	.	.	.
11	*Valerianella carinata* VCA1106	.	.	.	.	.	.	.	.	.	.	.	.	.	.	.	.	.	.	.	.	.	.	.	A	.	.	.	A	.	.	.	.	.	.	.	.	.	.	.	.	.	.	.	.	.	.	.	.	.	.	.	.	.	.	.	.	.	.	.	.	.	.	.
12	*Valerianella carinata* VCA1202	.	.	.	.	.	.	.	.	.	.	.	.	.	.	.	.	.	.	.	.	.	.	.	A	.	.	.	A	.	.	.	.	.	.	.	.	.	.	.	.	.	.	.	.	.	.	.	.	.	.	.	.	.	.	.	.	.	.	.	.	.	.	.
13	*Valerianella coronata* VCO0102	.	.	.	.	.	.	.	.	.	.	.	.	.	.	.	.	.	.	.	.	.	.	.	G	.	.	.	A	.	.	.	.	.	.	.	.	.	.	.	.	.	.	.	.	.	.	.	.	.	.	.	.	.	.	.	.	.	.	.	.	.	.	.
14	*Valerianella coronata* VCO0218	.	.	.	.	.	.	.	.	.	.	.	.	.	.	.	.	.	.	.	.	.	.	.	G	.	.	.	A	.	.	.	.	.	.	.	.	.	.	.	.	.	.	.	.	.	.	.	.	.	.	.	.	.	.	.	.	.	.	.	.	.	.	.
15	*Valerianella coronata* VCO0404	.	.	.	.	.	.	.	.	.	.	.	.	.	.	.	.	.	.	.	.	.	.	.	G	.	.	.	A	.	.	.	.	.	.	.	.	.	.	.	.	.	.	.	.	.	.	.	.	.	.	.	.	.	.	.	.	.	.	.	.	.	.	.
16	*Valerianella coronata* VCO0508	.	.	.	.	.	.	.	.	.	.	.	.	.	.	.	.	.	.	.	.	.	.	.	G	.	.	.	A	.	.	.	.	.	.	.	.	.	.	.	.	.	.	.	.	.	.	.	.	.	.	.	.	.	.	.	.	.	.	.	.	.	.	.
17	*Valerianella coronata* VCO0705	.	.	.	.	.	.	.	.	.	.	.	.	.	.	.	.	.	.	.	.	.	.	.	G	.	.	.	A	.	.	.	.	.	.	.	.	.	.	.	.	.	.	.	.	.	.	.	.	.	.	.	.	.	.	.	.	.	.	.	.	.	.	.
18	*Valerianella coronata* VCO0805	.	.	.	.	.	.	.	.	.	.	.	.	.	.	.	.	.	.	.	.	.	.	.	G	.	.	.	A	.	.	.	.	.	.	.	.	.	.	.	.	.	.	.	.	.	.	.	.	.	.	.	.	.	.	.	.	.	.	.	.	.	.	.
19	*Valerianella coronata* VCO0901	.	.	.	.	.	.	.	.	.	.	.	.	.	.	.	.	.	.	.	.	.	.	.	G	.	.	.	A	.	.	.	.	.	.	.	.	.	.	.	.	.	.	.	.	.	.	.	.	.	.	.	.	.	.	.	.	.	.	.	.	.	.	.
20	*Valerianella dentata* VDE0303	.	.	.	.	.	.	.	.	.	.	.	.	.	.	.	.	.	.	.	.	.	.	.	G	.	.	.	A	.	.	.	.	.	.	.	.	.	.	.	.	.	.	.	.	.	.	.	.	.	.	.	.	.	.	.	.	.	.	.	.	.	.	.
21	*Valerianella discoidea* VDI0202	.	.	.	.	.	.	.	.	.	.	.	.	.	.	.	.	.	.	.	.	.	.	.	G	.	.	.	A	.	.	.	.	.	.	.	.	.	.	.	.	.	.	.	.	.	.	.	.	.	.	.	.	.	.	.	.	.	.	.	.	.	.	.
22	*Valerianella discoidea* VDI0412	.	.	.	.	.	.	.	.	.	.	.	.	.	.	.	.	.	.	.	.	.	.	.	G	.	.	.	A	.	.	.	.	.	.	.	.	.	.	.	.	.	.	.	.	.	.	.	.	.	.	.	.	.	.	.	.	.	.	.	.	.	.	.
23	*Valerianella echinata* VEC0101	.	.	.	.	.	.	.	.	.	.	.	.	.	.	.	.	.	.	.	.	.	.	.	A	.	.	.	A	.	.	.	.	.	.	.	.	.	.	.	.	.	.	.	.	.	.	.	.	.	.	.	.	.	.	.	.	.	.	.	.	.	.	.
24	*Valerianella eriocarpa* VER0208	.	.	.	.	.	.	.	.	.	.	.	.	.	.	.	.	.	.	.	.	.	.	.	G	.	.	.	A	.	.	.	.	.	.	.	.	.	.	.	.	.	.	.	.	.	.	.	.	.	.	.	.	.	.	.	.	.	.	.	.	.	.	.
25	*Valerianella eriocarpa* VER0402	.	.	.	.	.	.	.	.	.	.	.	.	.	.	.	.	.	.	.	.	.	.	.	G	.	.	.	A	.	.	.	.	.	.	.	.	.	.	.	.	.	.	.	.	.	.	.	.	.	.	.	.	.	.	.	.	.	.	.	.	.	.	.
26	*Valerianella fusiformis* VFU0103	.	.	.	.	.	.	.	.	.	.	.	.	.	.	.	.	.	.	.	.	.	.	.	A	.	.	.	A	.	.	.	.	.	.	.	.	.	.	.	.	.	.	.	T	.	.	.	.	.	.	.	.	.	.	.	.	.	.	.	.	.	.	.
27	*Valerianella locusta* VLO0103	.	.	.	.	.	.	.	.	.	.	.	.	.	.	.	.	.	.	.	.	.	.	.	A	.	.	.	A	.	.	.	.	.	.	.	.	.	.	.	.	.	.	.	.	.	.	.	.	.	.	.	.	.	.	.	.	.	.	.	.	.	.	.
28	*Valerianella locusta* VLO0305	.	.	.	.	.	.	.	.	.	.	.	.	.	.	.	.	.	.	.	.	.	.	.	A	.	.	.	A	.	.	.	.	.	.	.	.	.	.	.	.	.	.	.	.	.	.	.	.	.	.	.	.	.	.	.	.	.	.	.	.	.	.	.
29	*Valerianella locusta* VLO0409	.	.	.	.	.	.	.	.	.	.	.	.	.	.	.	.	.	.	.	.	.	.	.	A	.	.	.	A	.	.	.	.	.	.	.	.	.	.	.	.	.	.	.	.	.	.	.	.	.	.	.	.	.	.	.	.	.	.	.	.	.	.	.
30	*Valerianella locusta* VLO0505	.	.	.	.	.	.	.	.	.	.	.	.	.	.	.	.	.	.	.	.	.	.	.	A	.	.	.	A	.	.	.	.	.	.	.	.	.	.	.	.	.	.	.	.	.	.	.	.	.	.	.	.	.	.	.	.	.	.	.	.	.	.	.
31	*Valerianella locusta* VLO0609	.	.	.	.	.	.	.	.	.	.	.	.	.	.	.	.	.	.	.	.	.	.	.	A	.	.	.	A	.	.	.	.	.	.	.	.	.	.	.	.	.	.	.	.	.	.	.	.	.	.	.	.	.	.	.	.	.	.	.	.	.	.	.
32	*Valerianella lusitanica* VLU0102	.	.	.	.	.	.	.	.	.	.	.	.	.	.	.	.	.	.	.	.	.	.	.	A	.	.	.	A	.	.	.	.	.	.	.	.	.	.	.	.	.	.	.	.	.	.	.	.	.	.	.	.	.	.	.	.	.	.	.	.	.	.	.
33	*Valerianella microcarpa* VMI0102	.	.	.	.	.	.	.	.	.	.	.	.	.	.	.	.	.	.	.	.	.	.	.	G	.	.	.	A	.	.	.	.	.	.	.	.	.	.	.	.	.	.	.	.	.	.	.	.	.	.	.	.	.	.	.	.	.	.	.	.	.	.	.
34	*Valerianella multidentata* VMU0201	.	.	.	.	.	.	.	.	.	.	.	.	.	.	.	.	.	.	.	.	.	.	.	G	.	.	.	A	.	.	.	.	.	.	.	.	.	.	.	.	.	.	.	.	.	.	.	.	.	.	.	.	.	.	.	.	.	.	.	.	.	.	.
35	*Valerianella multidentata* VMU0301	.	.	.	.	.	.	.	.	.	.	.	.	.	.	.	.	.	.	.	.	.	.	.	G	.	.	.	A	.	.	.	.	.	.	.	.	.	.	.	.	.	.	.	.	.	.	.	.	.	.	.	.	.	.	.	.	.	.	.	.	.	.	.
36	*Valerianella pumila* VPU0103	.	.	.	.	.	.	.	.	.	.	.	.	.	.	.	.	.	.	.	.	.	.	.	G	.	.	.	A	.	.	.	.	.	.	.	.	.	.	.	.	.	.	.	.	.	.	.	.	.	.	.	.	.	.	.	.	.	.	.	.	.	.	.
37	*Valerianella pumila* VPU0304	.	.	.	.	.	.	.	.	.	.	.	.	.	.	.	.	.	.	.	.	.	.	.	G	.	.	.	A	.	.	.	.	.	.	.	.	.	.	.	.	.	.	.	.	.	.	.	.	.	.	.	.	.	.	.	.	.	.	.	.	.	.	.
38	*Valerianella pumila* VPU0402	.	.	.	.	.	.	.	.	.	.	.	.	.	.	.	.	.	.	.	.	.	.	.	G	.	.	.	A	.	.	.	.	.	.	.	.	.	.	.	.	.	.	.	.	.	.	.	.	.	.	.	.	.	.	.	.	.	.	.	.	.	.	.
39	*Valerianella rimosa* VRI0101	.	.	.	.	.	.	.	.	.	.	.	.	.	.	.	.	.	.	.	.	.	.	.	G	.	.	.	A	.	.	.	.	.	.	.	.	.	.	.	.	.	.	.	.	.	.	.	.	.	.	.	.	.	.	.	.	.	.	.	.	.	.	.
40	*Valerianella rimosa* VRI0201	.	.	.	.	.	.	.	.	.	.	.	.	.	.	.	.	.	.	.	.	.	.	.	G	.	.	.	A	.	.	.	.	.	.	.	.	.	.	.	.	.	.	.	.	.	.	.	.	.	.	.	.	.	.	.	.	.	.	.	.	.	.	.

	Position	1135	1136	1137	1138	1139	1140	1141	1142	1143	1144	1145	1146	1147	1148	1149	1150	1151	1152	1153	1154	1155	1156	1157	1158	1159	1160	1161	1162	1163	1164	1165	1166	1167	1168	1169	1170	1171	1172	1173	1174	1175	1176	1177	1178	1179	1180	1181	1182	1183	1184	1185	1186	1187	1188	1189	1190	1191	1192	1193	1194	1195	1196	1197
1	*Centranthus calcitrapae* CC25	T	G	C	C	G	G	C	A	T	T	G	G	C	A	T	G	T	A	G	A	A	T	G	G	G	A	C	T	C	T	A	T	C	T	T	T	A	T	T	C	T	C	G	T	T	C	G	A	T	T	A	A	T	C	A	G	T	T	C	T	T	C	A
2	*Centranthus calcitrapae* CCACC539	.	.	.	.	.	.	.	.	.	.	.	.	.	.	.	.	.	.	.	.	.	.	.	.	.	.	.	.	.	.	.	.	.	.	.	.	.	.	.	.	.	.	.	.	.	.	.	.	.	.	.	.	.	.	.	.	.	.	.	.	.	.	.
3	*Fedia cornucopiae* FC73	.	.	.	.	.	.	T	.	.	.	.	.	.	.	.	.	.	.	.	.	.	.	.	.	.	.	.	.	.	.	C	.	.	.	.	.	.	.	.	.	.	T	.	.	G	.	.	.	.	.	.	.	.	.	G	.	.	.	.	.	.	.	.
4	*Valerianella carinata* VCA0104	.	.	.	.	.	.	T	.	.	.	.	.	.	.	.	.	.	.	.	.	.	.	A	.	.	.	.	.	.	.	.	.	.	.	.	.	.	.	.	.	.	T	.	.	G	.	.	.	.	.	.	.	.	.	G	.	.	.	.	.	.	.	.
5	*Valerianella carinata* VCA0202	.	.	.	.	.	.	T	.	.	.	.	.	.	.	.	.	.	.	.	.	.	.	A	.	.	.	.	.	.	.	.	.	.	.	.	.	.	.	.	.	.	T	.	.	G	.	.	.	.	.	.	.	.	.	G	.	.	.	.	.	.	.	.
6	*Valerianella carinata* VCA0302	.	.	.	.	.	.	T	.	.	.	.	.	.	.	.	.	.	.	.	.	.	.	A	.	.	.	.	.	.	.	.	.	.	.	.	.	.	.	.	.	.	T	.	.	G	.	.	.	.	.	.	.	.	T	G	.	.	.	.	.	.	.	.
7	*Valerianella carinata* VCA0501	.	.	.	.	.	.	T	.	.	.	.	.	.	.	.	.	.	.	.	.	.	.	A	.	.	.	.	.	.	.	.	.	.	.	.	.	.	.	.	.	.	T	.	.	G	.	.	.	.	.	.	.	.	.	G	.	.	.	.	.	.	.	.
8	*Valerianella carinata* VCA0805	.	.	.	.	.	.	T	.	.	.	.	.	.	.	.	.	.	.	.	.	.	.	A	.	.	.	.	.	.	.	.	.	.	.	.	.	.	.	.	.	.	T	.	.	G	.	.	.	.	.	.	.	.	.	G	.	.	.	.	.	.	.	.
9	*Valerianella carinata* VCA0918	.	.	.	.	.	.	T	.	.	.	.	.	.	.	.	.	.	.	.	.	.	.	A	.	.	.	.	.	.	.	.	.	.	.	.	.	.	.	.	.	.	T	.	.	G	.	.	.	.	.	.	.	.	.	G	.	.	.	.	.	.	.	.
10	*Valerianella carinata* VCA1006	.	.	.	.	.	.	T	.	.	.	.	.	.	.	.	.	.	.	.	.	.	.	A	.	.	.	.	.	.	.	.	.	.	.	.	.	.	.	.	.	.	T	.	.	G	.	.	.	.	.	.	.	.	.	G	.	.	.	.	.	.	.	.
11	*Valerianella carinata* VCA1106	.	.	.	.	.	.	T	.	.	.	.	.	.	.	.	.	.	.	.	.	.	.	A	.	.	.	.	.	.	.	.	.	.	.	.	.	.	.	.	.	.	T	.	.	G	.	.	.	.	.	.	.	.	.	G	.	.	.	.	.	.	.	.
12	*Valerianella carinata* VCA1202	.	.	.	.	.	.	T	.	.	.	.	.	.	.	.	.	.	.	.	.	.	.	A	.	.	.	.	.	.	.	.	.	.	.	.	.	.	.	.	.	.	T	.	.	G	.	.	.	.	.	.	.	.	.	G	.	.	.	.	.	.	.	.
13	*Valerianella coronata* VCO0102	.	.	.	.	.	.	T	.	.	.	.	.	.	.	.	.	.	.	.	.	.	.	.	.	.	.	.	.	.	.	.	.	.	.	.	.	.	.	.	.	.	T	.	.	G	.	.	.	.	.	.	.	-	-	-	-	-	-	-	-	-	-	-
14	*Valerianella coronata* VCO0218	.	.	.	.	.	.	T	.	.	.	.	.	.	.	.	.	.	.	.	.	.	.	.	.	.	.	.	.	.	.	.	.	.	.	.	.	.	.	.	.	.	T	.	.	G	.	.	.	.	.	.	.	-	-	-	-	-	-	-	-	-	-	-
15	*Valerianella coronata* VCO0404	.	.	.	.	.	.	T	.	.	.	.	.	.	.	.	.	.	.	.	.	.	.	.	.	.	.	.	.	.	.	.	.	.	.	.	.	.	.	.	.	.	T	.	.	G	.	.	.	.	.	.	.	-	-	-	-	-	-	-	-	-	-	-
16	*Valerianella coronata* VCO0508	.	.	.	.	.	.	T	.	.	.	.	.	.	.	.	.	.	.	.	.	.	.	.	.	.	.	.	.	.	.	.	.	.	.	.	.	.	.	.	.	.	T	.	.	G	.	.	.	.	.	.	.	-	-	-	-	-	-	-	-	-	-	-
17	*Valerianella coronata* VCO0705	.	.	.	.	.	.	T	.	.	.	.	.	.	.	.	.	.	.	.	.	.	.	.	.	.	.	.	.	.	.	.	.	.	.	.	.	.	.	.	.	.	T	.	.	G	.	.	.	.	.	.	.	-	-	-	-	-	-	-	-	-	-	-
18	*Valerianella coronata* VCO0805	.	.	.	.	.	.	T	.	.	.	.	.	.	.	.	.	.	.	.	.	.	.	.	.	.	.	.	.	.	.	.	.	.	.	.	.	.	.	.	.	.	T	.	.	G	.	.	.	.	.	.	.	-	-	-	-	-	-	-	-	-	-	-
19	*Valerianella coronata* VCO0901	.	.	.	.	.	.	T	.	.	.	.	.	.	.	.	.	.	.	.	.	.	.	.	.	.	.	.	.	.	.	.	.	.	.	.	.	.	.	.	.	.	T	.	.	G	.	.	.	.	.	.	.	-	-	-	-	-	-	-	-	-	-	-
20	*Valerianella dentata* VDE0303	.	.	.	.	.	.	T	.	.	.	.	.	.	.	.	.	.	.	.	.	.	.	.	.	.	.	.	.	.	.	.	.	.	.	.	.	.	.	.	.	.	T	.	.	G	.	.	.	.	.	.	.	-	-	-	-	-	-	-	-	-	-	-
21	*Valerianella discoidea* VDI0202	.	.	.	.	.	.	T	.	.	.	.	.	.	.	.	.	.	.	.	.	.	.	.	.	.	.	.	.	.	.	.	.	.	.	.	.	.	.	.	.	.	T	.	.	G	.	.	.	.	.	.	.	-	-	-	-	-	-	-	-	-	-	-
22	*Valerianella discoidea* VDI0412	.	.	.	.	.	.	T	.	.	.	.	.	.	.	.	.	.	.	.	.	.	.	.	.	.	.	.	.	.	.	.	.	.	.	.	.	.	.	.	.	.	T	.	.	G	.	.	.	.	.	.	.	-	-	-	-	-	-	-	-	-	-	-
23	*Valerianella echinata* VEC0101	.	.	.	.	.	.	T	.	.	.	.	.	.	.	.	.	.	.	.	.	.	.	.	.	.	.	.	.	.	.	.	.	.	.	.	.	.	.	.	.	.	T	.	.	G	.	.	.	.	.	.	.	.	.	G	.	.	.	.	.	.	.	.
24	*Valerianella eriocarpa* VER0208	.	.	.	.	.	.	T	.	.	.	.	.	.	.	.	.	.	.	.	.	.	.	.	.	.	.	.	.	.	.	.	.	.	.	.	.	.	.	.	.	.	T	.	.	G	.	.	.	.	.	.	.	-	-	-	-	-	-	-	-	-	-	-
25	*Valerianella eriocarpa* VER0402	.	.	.	.	.	.	T	.	.	.	.	.	.	.	.	.	.	.	.	.	.	.	.	.	.	.	.	.	.	.	.	.	.	.	.	.	.	.	.	.	.	T	.	.	G	.	.	.	.	.	.	.	-	-	-	-	-	-	-	-	-	-	-
26	*Valerianella fusiformis* VFU0103	.	.	.	.	.	.	T	.	.	.	.	.	.	.	.	.	.	.	.	.	.	.	.	.	.	.	.	.	.	.	.	.	.	.	.	.	.	.	.	.	.	T	.	.	G	.	A	.	.	.	.	.	.	.	G	.	.	.	.	.	.	-	-
27	*Valerianella locusta* VLO0103	.	.	.	.	.	.	T	.	.	.	.	.	.	.	.	.	.	.	.	.	.	.	A	.	.	.	.	.	.	.	.	.	.	.	.	.	.	.	.	.	.	T	.	.	G	.	.	.	.	.	.	.	.	.	G	.	.	.	.	.	.	.	.
28	*Valerianella locusta* VLO0305	.	.	.	.	.	.	T	.	.	.	.	.	.	.	.	.	.	.	.	.	.	.	A	.	.	.	.	.	.	.	.	.	.	.	.	.	.	.	.	.	.	T	.	.	G	.	.	.	.	.	.	.	.	.	G	.	.	.	.	.	.	.	.
29	*Valerianella locusta* VLO0409	.	.	.	.	.	.	T	.	.	.	.	.	.	.	.	.	.	.	.	.	.	.	A	.	.	.	.	.	.	.	.	.	.	.	.	.	.	.	.	.	.	T	.	.	G	.	.	.	.	.	.	.	.	.	G	.	.	.	.	.	.	.	.
30	*Valerianella locusta* VLO0505	.	.	.	.	.	.	T	.	.	.	.	.	.	.	.	.	.	.	.	.	.	.	A	.	.	.	.	.	.	.	.	.	.	.	.	.	.	.	.	.	.	T	.	.	G	.	.	.	.	.	.	.	.	.	G	.	.	.	.	.	.	.	.
31	*Valerianella locusta* VLO0609	.	.	.	.	.	.	T	.	.	.	.	.	.	.	.	.	.	.	.	.	.	.	A	.	.	.	.	.	.	.	.	.	.	.	.	.	.	.	.	.	.	T	.	.	G	.	.	.	.	.	.	.	.	.	G	.	.	.	.	.	.	.	.
32	*Valerianella lusitanica* VLU0102	.	.	.	.	.	.	T	.	.	.	.	.	.	.	.	.	.	.	.	.	.	.	A	.	.	.	.	.	.	.	.	.	.	.	.	.	.	.	.	.	.	T	.	.	G	.	.	.	.	.	.	.	.	.	G	.	.	.	.	.	.	.	.
33	*Valerianella microcarpa* VMI0102	.	.	.	.	.	.	T	.	.	.	.	.	.	.	.	.	.	.	.	.	.	.	.	.	.	.	.	.	.	.	.	.	.	.	.	.	.	.	.	.	.	T	.	.	G	.	.	.	.	.	.	.	-	-	-	-	-	-	-	-	-	-	-
34	*Valerianella multidentata* VMU0201	.	.	.	.	.	.	T	.	.	.	.	.	.	.	.	.	.	.	.	.	.	.	.	.	.	.	.	.	.	.	.	.	.	.	.	.	.	.	.	.	.	T	.	.	G	.	.	.	.	.	.	.	-	-	-	-	-	-	-	-	-	-	-
35	*Valerianella multidentata* VMU0301	.	.	.	.	.	.	T	.	.	.	.	.	.	.	.	.	.	.	.	.	.	.	.	.	.	.	.	.	.	.	.	.	.	.	.	.	.	.	.	.	.	T	.	.	G	.	.	.	.	.	.	.	-	-	-	-	-	-	-	-	-	-	-
36	*Valerianella pumila* VPU0103	.	.	.	.	.	.	T	.	.	.	.	.	.	.	.	.	.	.	.	.	.	.	.	.	.	.	.	.	.	.	.	.	.	.	.	.	.	.	.	.	.	T	.	.	G	.	.	.	.	.	.	.	-	-	-	-	-	-	-	-	-	-	-
37	*Valerianella pumila* VPU0304	.	.	.	.	.	.	T	.	.	.	.	.	.	.	.	.	.	.	.	.	.	.	.	.	.	.	.	.	.	.	.	.	.	.	.	.	.	.	.	.	.	T	.	.	G	.	.	.	.	.	.	.	-	-	-	-	-	-	-	-	-	-	-
38	*Valerianella pumila* VPU0402	.	.	.	.	.	.	T	.	.	.	.	.	.	.	.	.	.	.	.	.	.	.	.	.	.	.	.	.	.	.	.	.	.	.	.	.	.	.	.	.	.	T	.	.	G	.	.	.	.	.	.	.	-	-	-	-	-	-	-	-	-	-	-
39	*Valerianella rimosa* VRI0101	.	.	.	.	.	.	T	.	.	.	.	.	.	.	.	.	.	.	.	.	.	.	.	.	.	.	.	.	.	.	.	.	.	.	.	.	.	.	.	.	.	T	.	.	G	.	.	.	.	.	.	.	-	-	-	-	-	-	-	-	-	-	-
40	*Valerianella rimosa* VRI0201	.	.	.	.	.	.	T	.	.	.	.	.	.	.	.	.	.	.	.	.	.	.	.	.	.	.	.	.	.	.	.	.	.	.	.	.	.	.	.	.	.	T	.	.	G	.	.	.	.	.	.	.	-	-	-	-	-	-	-	-	-	-	-

Position

		1198	1199	1200	1201	1202	1203	1204	1205	1206	1207	1208	1209	1210	1211	1212	1213	1214	1215	1216	1217	1218	1219	1220	1221	1222	1223	1224	1225	1226	1227	1228	1229	1230	1231	1232	1233	1234	1235	1236	1237	1238	1239	1240	1241	1242	1243	1244	1245	1246	1247	1248	1249	1250	1251	1252	1253	1254	1255	1256	1257	1258	1259	1260
1	*Centranthus calcitrapae* CC25	A	A	A	G	A	T	C	G	A	T	C	A	G	A	C	T	A	T	G	G	A	G	T	G	A	A	T	G	A	T	T	T	G	A	T	C	A	A	T	G	A	G	T	A	T	T	C	G	A	T	T	C	T	T	T	C	T	T	C	A	A	T	A
2	*Centranthus calcitrapae* CCACC539	.	.	.	.	.	.	.	.	.	.	.	.	.	.	.	.	.	.	.	.	.	.	.	.	.	.	.	.	.	.	.	.	.	.	.	.	.	.	.	.	.	.	.	.	.	.	.	.	.	.	.	.	.	.	.	.	.	.	.	.	.	.	.
3	*Fedia cornucopiae* FC73	.	.	.	.	.	.	.	.	.	.	.	.	.	.	.	.	.	G	.	.	.	.	.	.	.	T	C	.	.	.	.	.	.	G	.	.	-	-	-	-	-	-	-	-	-	-	-	-	-	-	-	-	-	-	-	-	-	-	-	-	-	-	-
4	*Valerianella carinata* VCA0104	.	.	.	.	.	.	T	.	.	.	.	.	.	.	.	.	.	G	.	.	.	.	.	.	.	T	C	.	.	.	.	.	.	.	.	.	-	-	-	-	-	-	-	-	-	-	-	-	-	-	-	-	-	-	-	-	-	-	-	-	-	-	-
5	*Valerianella carinata* VCA0202	.	.	.	.	.	.	.	.	.	.	.	.	.	.	.	.	.	G	.	.	.	.	.	.	.	T	C	.	.	.	.	.	.	.	.	.	-	-	-	-	-	-	-	-	-	-	-	-	-	-	-	-	-	-	-	-	-	-	-	-	-	-	-
6	*Valerianella carinata* VCA0302	.	.	.	.	.	.	.	.	.	.	.	.	.	.	.	.	.	G	.	.	.	.	.	.	.	T	C	.	.	.	.	.	.	.	.	.	-	-	-	-	-	-	-	-	-	-	-	-	-	-	-	-	-	-	-	-	-	-	-	-	-	-	-
7	*Valerianella carinata* VCA0501	.	.	.	.	.	.	.	.	.	.	.	.	.	.	.	.	.	G	.	.	.	.	.	.	.	T	C	.	.	.	.	.	.	.	.	.	-	-	-	-	-	-	-	-	-	-	-	-	-	-	-	-	-	-	-	-	-	-	-	-	-	-	-
8	*Valerianella carinata* VCA0805	.	.	.	.	.	.	.	.	.	.	.	.	.	.	.	.	.	G	.	.	.	.	.	.	.	T	C	.	.	.	.	.	.	.	.	.	-	-	-	-	-	-	-	-	-	-	-	-	-	-	-	-	-	-	-	-	-	-	-	-	-	-	-
9	*Valerianella carinata* VCA0918	.	.	.	.	.	.	.	.	.	.	.	.	.	.	.	.	.	G	.	.	.	.	.	.	.	T	C	.	.	.	.	.	.	.	.	.	-	-	-	-	-	-	-	-	-	-	-	-	-	-	-	-	-	-	-	-	-	-	-	-	-	-	-
10	*Valerianella carinata* VCA1006	.	.	.	.	.	.	.	.	.	.	.	.	.	.	.	.	.	G	.	.	.	.	.	.	.	T	C	.	.	.	.	.	.	.	.	.	-	-	-	-	-	-	-	-	-	-	-	-	-	-	-	-	-	-	-	-	-	-	-	-	-	-	-
11	*Valerianella carinata* VCA1106	.	.	.	.	.	.	.	.	.	.	.	.	.	.	.	.	.	G	.	.	.	.	.	.	.	T	C	.	.	.	.	.	.	.	.	.	-	-	-	-	-	-	-	-	-	-	-	-	-	-	-	-	-	-	-	-	-	-	-	-	-	-	-
12	*Valerianella carinata* VCA1202	.	.	.	.	.	.	.	.	.	.	.	.	.	.	.	.	.	G	.	.	.	.	.	.	.	T	C	.	.	.	.	.	.	.	.	.	-	-	-	-	-	-	-	-	-	-	-	-	-	-	-	-	-	-	-	-	-	-	-	-	-	-	-
13	*Valerianella coronata* VCO0102	-	-	-	-	-	-	-	-	-	-	-	-	-	-	-	-	-	-	-	-	-	-	-	-	-	-	-	-	-	-	-	-	-	-	-	-	-	-	-	-	-	-	-	-	-	-	-	-	-	-	-	-	-	-	-	-	-	-	-	-	-	-	-
14	*Valerianella coronata* VCO0218	-	-	-	-	-	-	-	-	-	-	-	-	-	-	-	-	-	-	-	-	-	-	-	-	-	-	-	-	-	-	-	-	-	-	-	-	-	-	-	-	-	-	-	-	-	-	-	-	-	-	-	-	-	-	-	-	-	-	-	-	-	-	-
15	*Valerianella coronata* VCO0404	-	-	-	-	-	-	-	-	-	-	-	-	-	-	-	-	-	-	-	-	-	-	-	-	-	-	-	-	-	-	-	-	-	-	-	-	-	-	-	-	-	-	-	-	-	-	-	-	-	-	-	-	-	-	-	-	-	-	-	-	-	-	-
16	*Valerianella coronata* VCO0508	-	-	-	-	-	-	-	-	-	-	-	-	-	-	-	-	-	-	-	-	-	-	-	-	-	-	-	-	-	-	-	-	-	-	-	-	-	-	-	-	-	-	-	-	-	-	-	-	-	-	-	-	-	-	-	-	-	-	-	-	-	-	-
17	*Valerianella coronata* VCO0705	-	-	-	-	-	-	-	-	-	-	-	-	-	-	-	-	-	-	-	-	-	-	-	-	-	-	-	-	-	-	-	-	-	-	-	-	-	-	-	-	-	-	-	-	-	-	-	-	-	-	-	-	-	-	-	-	-	-	-	-	-	-	-
18	*Valerianella coronata* VCO0805	-	-	-	-	-	-	-	-	-	-	-	-	-	-	-	-	-	-	-	-	-	-	-	-	-	-	-	-	-	-	-	-	-	-	-	-	-	-	-	-	-	-	-	-	-	-	-	-	-	-	-	-	-	-	-	-	-	-	-	-	-	-	-
19	*Valerianella coronata* VCO0901	-	-	-	-	-	-	-	-	-	-	-	-	-	-	-	-	-	-	-	-	-	-	-	-	-	-	-	-	-	-	-	-	-	-	-	-	-	-	-	-	-	-	-	-	-	-	-	-	-	-	-	-	-	-	-	-	-	-	-	-	-	-	-
20	*Valerianella dentata* VDE0303	-	-	-	-	-	-	-	-	-	-	-	-	-	-	-	-	-	-	-	-	-	-	-	-	-	-	-	-	-	-	-	-	-	-	-	-	-	-	-	-	-	-	-	-	-	-	-	-	-	-	-	-	-	-	-	-	-	-	-	-	-	-	-
21	*Valerianella discoidea* VDI0202	-	-	-	-	-	-	-	-	-	-	-	-	-	-	-	-	-	-	-	-	-	-	-	-	-	-	-	-	-	-	-	-	-	-	-	-	-	-	-	-	-	-	-	-	-	-	-	-	-	-	-	-	-	-	-	-	-	-	-	-	-	-	-
22	*Valerianella discoidea* VDI0412	-	-	-	-	-	-	-	-	-	-	-	-	-	-	-	-	-	-	-	-	-	-	-	-	-	-	-	-	-	-	-	-	-	-	-	-	-	-	-	-	-	-	-	-	-	-	-	-	-	-	-	-	-	-	-	-	-	-	-	-	-	-	-
23	*Valerianella echinata* VEC0101	.	.	.	.	G	.	.	.	.	.	.	.	.	.	T	.	.	G	.	.	.	.	.	.	.	T	C	.	.	.	.	.	.	.	.	.	-	-	-	-	-	-	-	-	-	-	-	-	-	-	-	-	-	-	-	-	-	-	-	-	-	-	-
24	*Valerianella eriocarpa* VER0208	-	-	-	-	-	-	-	-	-	-	-	-	-	-	-	-	-	-	-	-	-	-	-	-	-	-	-	-	-	-	-	-	-	-	-	-	-	-	-	-	-	-	-	-	-	-	-	-	-	-	-	-	-	-	-	-	-	-	-	-	-	-	-
25	*Valerianella eriocarpa* VER0402	-	-	-	-	-	-	-	-	-	-	-	-	-	-	-	-	-	-	-	-	-	-	-	-	-	-	-	-	-	-	-	-	-	-	-	-	-	-	-	-	-	-	-	-	-	-	-	-	-	-	-	-	-	-	-	-	-	-	-	-	-	-	-
26	*Valerianella fusiformis* VFU0103	-	-	-	-	.	-	-	-	.	.	.	.	.	.	.	.	.	G	.	.	.	.	.	.	.	.	.	.	.	.	.	.	.	.	.	.	-	-	-	-	-	-	-	-	-	-	-	-	-	-	-	-	-	-	-	-	-	-	-	-	-	-	-
27	*Valerianella locusta* VLO0103	.	.	.	.	.	.	.	.	.	.	.	.	.	.	.	.	.	G	.	.	.	.	.	.	.	T	C	.	.	.	.	.	.	.	.	.	-	-	-	-	-	-	-	-	-	-	-	-	-	-	-	-	-	-	-	-	-	-	-	-	-	-	-
28	*Valerianella locusta* VLO0305	.	.	.	.	.	.	.	.	.	.	.	.	.	.	.	.	.	G	.	.	.	.	.	.	.	T	C	.	.	.	.	.	.	.	.	.	-	-	-	-	-	-	-	-	-	-	-	-	-	-	-	-	-	-	-	-	-	-	-	-	-	-	-
29	*Valerianella locusta* VLO0409	.	.	.	.	.	.	.	.	.	.	.	.	.	.	.	.	.	G	.	.	.	.	.	.	.	T	C	.	.	.	.	.	.	.	.	.	-	-	-	-	-	-	-	-	-	-	-	-	-	-	-	-	-	-	-	-	-	-	-	-	-	-	-
30	*Valerianella locusta* VLO0505	.	.	.	.	.	.	.	.	.	.	.	.	.	.	.	.	.	G	.	.	.	.	.	.	.	T	C	.	.	.	.	.	.	.	.	.	-	-	-	-	-	-	-	-	-	-	-	-	-	-	-	-	-	-	-	-	-	-	-	-	-	-	-
31	*Valerianella locusta* VLO0609	.	.	.	.	.	.	.	.	.	.	.	.	.	.	.	.	.	G	.	.	.	.	.	.	.	T	C	.	.	.	.	.	.	.	.	.	-	-	-	-	-	-	-	-	-	-	-	-	-	-	-	-	-	-	-	-	-	-	-	-	-	-	-
32	*Valerianella lusitanica* VLU0102	.	.	.	.	.	.	T	.	.	.	.	.	.	.	.	.	.	G	.	.	.	.	.	.	.	T	C	.	.	.	.	.	.	.	.	.	-	-	-	-	-	-	-	-	-	-	-	-	-	-	-	-	-	-	-	-	-	-	-	-	-	-	-
33	*Valerianella microcarpa* VMI0102	-	-	-	-	-	-	-	-	-	-	-	-	-	-	-	-	-	-	-	-	-	-	-	-	-	-	-	-	-	-	-	-	-	-	-	-	-	-	-	-	-	-	-	-	-	-	-	-	-	-	-	-	-	-	-	-	-	-	-	-	-	-	-
34	*Valerianella multidentata* VMU0201	-	-	-	-	-	-	-	-	-	-	-	-	-	-	-	-	-	-	-	-	-	-	-	-	-	-	-	-	-	-	-	-	-	-	-	-	-	-	-	-	-	-	-	-	-	-	-	-	-	-	-	-	-	-	-	-	-	-	-	-	-	-	-
35	*Valerianella multidentata* VMU0301	-	-	-	-	-	-	-	-	-	-	-	-	-	-	-	-	-	-	-	-	-	-	-	-	-	-	-	-	-	-	-	-	-	-	-	-	-	-	-	-	-	-	-	-	-	-	-	-	-	-	-	-	-	-	-	-	-	-	-	-	-	-	-
36	*Valerianella pumila* VPU0103	-	-	-	-	-	-	-	-	-	-	-	-	-	-	-	-	-	-	-	-	-	-	-	-	-	-	-	-	-	-	-	-	-	-	-	-	-	-	-	-	-	-	-	-	-	-	-	-	-	-	-	-	-	-	-	-	-	-	-	-	-	-	-
37	*Valerianella pumila* VPU0304	-	-	-	-	-	-	-	-	-	-	-	-	-	-	-	-	-	-	-	-	-	-	-	-	-	-	-	-	-	-	-	-	-	-	-	-	-	-	-	-	-	-	-	-	-	-	-	-	-	-	-	-	-	-	-	-	-	-	-	-	-	-	-
38	*Valerianella pumila* VPU0402	-	-	-	-	-	-	-	-	-	-	-	-	-	-	-	-	-	-	-	-	-	-	-	-	-	-	-	-	-	-	-	-	-	-	-	-	-	-	-	-	-	-	-	-	-	-	-	-	-	-	-	-	-	-	-	-	-	-	-	-	-	-	-
39	*Valerianella rimosa* VRI0101	-	-	-	-	-	-	-	-	-	-	-	-	-	-	-	-	-	-	-	-	-	-	-	-	-	-	-	-	-	-	-	-	-	-	-	-	-	-	-	-	-	-	-	-	-	-	-	-	-	-	-	-	-	-	-	-	-	-	-	-	-	-	-
40	*Valerianella rimosa* VRI0201	-	-	-	-	-	-	-	-	-	-	-	-	-	-	-	-	-	-	-	-	-	-	-	-	-	-	-	-	-	-	-	-	-	-	-	-	-	-	-	-	-	-	-	-	-	-	-	-	-	-	-	-	-	-	-	-	-	-	-	-	-	-	-

	Position	1261	1262	1263	1264	1265	1266	1267	1268	1269	1270	1271	1272	1273	1274	1275	1276	1277	1278	1279	1280	1281	1282	1283	1284	1285	1286	1287	1288	1289	1290	1291	1292	1293	1294	1295	1296	1297	1298	1299	1300	1301	1302	1303	1304	1305	1306	1307	1308	1309	1310	1311	1312	1313	1314	1315	1316	1317	1318	1319	1320	1321	1322	1323
1	*Centranthus calcitrapae* CC25	T	G	G	A	A	T	C	A	A	T	T	C	A	C	A	A	T	C	A	T	T	T	T	C	C	C	A	T	T	T	T	G	A	T	A	A	T	A	C	A	G	A	T	T	C	G	G	G	T	C	G	T	C	A	T	T	A	A	T	C	A	G	T
2	*Centranthus calcitrapae* CCACC539	.	.	.	.	.	.	.	.	.	.	.	.	.	.	.	.	.	.	.	.	.	.	.	.	.	.	.	.	.	.	.	.	.	.	.	.	.	.	.	.	.	.	.	.	.	.	.	.	.	.	.	.	.	.	.	.	.	.	.	.	.	.	.
3	*Fedia cornucopiae* FC73	-	-	-	-	-	-	-	-	-	-	-	-	-	-	-	-	-	-	-	-	-	-	-	-	-	-	-	-	-	-	-	-	-	-	-	-	-	-	-	-	-	-	-	-	-	-	-	-	-	-	-	-	-	-	-	-	-	-	-	-	-	-	-
4	*Valerianella carinata* VCA0104	-	-	-	-	-	-	-	-	-	-	-	-	-	-	-	-	-	-	-	-	-	-	-	-	-	-	-	-	-	-	-	-	-	-	-	-	-	-	-	-	-	-	-	-	-	-	-	-	-	-	-	-	-	-	-	-	-	-	-	-	-	-	-
5	*Valerianella carinata* VCA0202	-	-	-	-	-	-	-	-	-	-	-	-	-	-	-	-	-	-	-	-	-	-	-	-	-	-	-	-	-	-	-	-	-	-	-	-	-	-	-	-	-	-	-	-	-	-	-	-	-	-	-	-	-	-	-	-	-	-	-	-	-	-	-
6	*Valerianella carinata* VCA0302	-	-	-	-	-	-	-	-	-	-	-	-	-	-	-	-	-	-	-	-	-	-	-	-	-	-	-	-	-	-	-	-	-	-	-	-	-	-	-	-	-	-	-	-	-	-	-	-	-	-	-	-	-	-	-	-	-	-	-	-	-	-	-
7	*Valerianella carinata* VCA0501	-	-	-	-	-	-	-	-	-	-	-	-	-	-	-	-	-	-	-	-	-	-	-	-	-	-	-	-	-	-	-	-	-	-	-	-	-	-	-	-	-	-	-	-	-	-	-	-	-	-	-	-	-	-	-	-	-	-	-	-	-	-	-
8	*Valerianella carinata* VCA0805	-	-	-	-	-	-	-	-	-	-	-	-	-	-	-	-	-	-	-	-	-	-	-	-	-	-	-	-	-	-	-	-	-	-	-	-	-	-	-	-	-	-	-	-	-	-	-	-	-	-	-	-	-	-	-	-	-	-	-	-	-	-	-
9	*Valerianella carinata* VCA0918	-	-	-	-	-	-	-	-	-	-	-	-	-	-	-	-	-	-	-	-	-	-	-	-	-	-	-	-	-	-	-	-	-	-	-	-	-	-	-	-	-	-	-	-	-	-	-	-	-	-	-	-	-	-	-	-	-	-	-	-	-	-	-
10	*Valerianella carinata* VCA1006	-	-	-	-	-	-	-	-	-	-	-	-	-	-	-	-	-	-	-	-	-	-	-	-	-	-	-	-	-	-	-	-	-	-	-	-	-	-	-	-	-	-	-	-	-	-	-	-	-	-	-	-	-	-	-	-	-	-	-	-	-	-	-
11	*Valerianella carinata* VCA1106	-	-	-	-	-	-	-	-	-	-	-	-	-	-	-	-	-	-	-	-	-	-	-	-	-	-	-	-	-	-	-	-	-	-	-	-	-	-	-	-	-	-	-	-	-	-	-	-	-	-	-	-	-	-	-	-	-	-	-	-	-	-	-
12	*Valerianella carinata* VCA1202	-	-	-	-	-	-	-	-	-	-	-	-	-	-	-	-	-	-	-	-	-	-	-	-	-	-	-	-	-	-	-	-	-	-	-	-	-	-	-	-	-	-	-	-	-	-	-	-	-	-	-	-	-	-	-	-	-	-	-	-	-	-	-
13	*Valerianella coronata* VCO0102	-	-	-	-	-	-	-	-	-	-	-	-	-	-	-	-	-	-	-	-	-	-	-	-	-	-	-	-	-	-	-	-	-	-	-	-	-	-	-	-	-	-	-	-	-	-	-	-	-	-	-	-	-	-	-	-	-	-	-	-	-	-	-
14	*Valerianella coronata* VCO0218	-	-	-	-	-	-	-	-	-	-	-	-	-	-	-	-	-	-	-	-	-	-	-	-	-	-	-	-	-	-	-	-	-	-	-	-	-	-	-	-	-	-	-	-	-	-	-	-	-	-	-	-	-	-	-	-	-	-	-	-	-	-	-
15	*Valerianella coronata* VCO0404	-	-	-	-	-	-	-	-	-	-	-	-	-	-	-	-	-	-	-	-	-	-	-	-	-	-	-	-	-	-	-	-	-	-	-	-	-	-	-	-	-	-	-	-	-	-	-	-	-	-	-	-	-	-	-	-	-	-	-	-	-	-	-
16	*Valerianella coronata* VCO0508	-	-	-	-	-	-	-	-	-	-	-	-	-	-	-	-	-	-	-	-	-	-	-	-	-	-	-	-	-	-	-	-	-	-	-	-	-	-	-	-	-	-	-	-	-	-	-	-	-	-	-	-	-	-	-	-	-	-	-	-	-	-	-
17	*Valerianella coronata* VCO0705	-	-	-	-	-	-	-	-	-	-	-	-	-	-	-	-	-	-	-	-	-	-	-	-	-	-	-	-	-	-	-	-	-	-	-	-	-	-	-	-	-	-	-	-	-	-	-	-	-	-	-	-	-	-	-	-	-	-	-	-	-	-	-
18	*Valerianella coronata* VCO0805	-	-	-	-	-	-	-	-	-	-	-	-	-	-	-	-	-	-	-	-	-	-	-	-	-	-	-	-	-	-	-	-	-	-	-	-	-	-	-	-	-	-	-	-	-	-	-	-	-	-	-	-	-	-	-	-	-	-	-	-	-	-	-
19	*Valerianella coronata* VCO0901	-	-	-	-	-	-	-	-	-	-	-	-	-	-	-	-	-	-	-	-	-	-	-	-	-	-	-	-	-	-	-	-	-	-	-	-	-	-	-	-	-	-	-	-	-	-	-	-	-	-	-	-	-	-	-	-	-	-	-	-	-	-	-
20	*Valerianella dentata* VDE0303	-	-	-	-	-	-	-	-	-	-	-	-	-	-	-	-	-	-	-	-	-	-	-	-	-	-	-	-	-	-	-	-	-	-	-	-	-	-	-	-	-	-	-	-	-	-	-	-	-	-	-	-	-	-	-	-	-	-	-	-	-	-	-
21	*Valerianella discoidea* VDI0202	-	-	-	-	-	-	-	-	-	-	-	-	-	-	-	-	-	-	-	-	-	-	-	-	-	-	-	-	-	-	-	-	-	-	-	-	-	-	-	-	-	-	-	-	-	-	-	-	-	-	-	-	-	-	-	-	-	-	-	-	-	-	-
22	*Valerianella discoidea* VDI0412	-	-	-	-	-	-	-	-	-	-	-	-	-	-	-	-	-	-	-	-	-	-	-	-	-	-	-	-	-	-	-	-	-	-	-	-	-	-	-	-	-	-	-	-	-	-	-	-	-	-	-	-	-	-	-	-	-	-	-	-	-	-	-
23	*Valerianella echinata* VEC0101	-	-	-	-	-	-	-	-	-	-	-	-	-	-	-	-	-	-	-	-	-	-	-	-	-	-	-	-	-	-	-	-	-	-	-	-	-	-	-	-	-	-	-	-	-	-	-	-	-	-	-	-	-	-	-	-	-	-	-	-	-	-	-
24	*Valerianella eriocarpa* VER0208	-	-	-	-	-	-	-	-	-	-	-	-	-	-	-	-	-	-	-	-	-	-	-	-	-	-	-	-	-	-	-	-	-	-	-	-	-	-	-	-	-	-	-	-	-	-	-	-	-	-	-	-	-	-	-	-	-	-	-	-	-	-	-
25	*Valerianella eriocarpa* VER0402	-	-	-	-	-	-	-	-	-	-	-	-	-	-	-	-	-	-	-	-	-	-	-	-	-	-	-	-	-	-	-	-	-	-	-	-	-	-	-	-	-	-	-	-	-	-	-	-	-	-	-	-	-	-	-	-	-	-	-	-	-	-	-
26	*Valerianella fusiformis* VFU0103	-	-	-	-	-	-	-	-	-	-	-	-	-	-	-	-	-	-	-	-	-	-	-	-	-	-	-	-	-	-	-	-	-	-	-	-	-	-	-	-	-	-	-	-	-	-	-	-	-	-	-	-	-	-	-	-	-	-	-	-	-	-	-
27	*Valerianella locusta* VLO0103	-	-	-	-	-	-	-	-	-	-	-	-	-	-	-	-	-	-	-	-	-	-	-	-	-	-	-	-	-	-	-	-	-	-	-	-	-	-	-	-	-	-	-	-	-	-	-	-	-	-	-	-	-	-	-	-	-	-	-	-	-	-	-
28	*Valerianella locusta* VLO0305	-	-	-	-	-	-	-	-	-	-	-	-	-	-	-	-	-	-	-	-	-	-	-	-	-	-	-	-	-	-	-	-	-	-	-	-	-	-	-	-	-	-	-	-	-	-	-	-	-	-	-	-	-	-	-	-	-	-	-	-	-	-	-
29	*Valerianella locusta* VLO0409	-	-	-	-	-	-	-	-	-	-	-	-	-	-	-	-	-	-	-	-	-	-	-	-	-	-	-	-	-	-	-	-	-	-	-	-	-	-	-	-	-	-	-	-	-	-	-	-	-	-	-	-	-	-	-	-	-	-	-	-	-	-	-
30	*Valerianella locusta* VLO0505	-	-	-	-	-	-	-	-	-	-	-	-	-	-	-	-	-	-	-	-	-	-	-	-	-	-	-	-	-	-	-	-	-	-	-	-	-	-	-	-	-	-	-	-	-	-	-	-	-	-	-	-	-	-	-	-	-	-	-	-	-	-	-
31	*Valerianella locusta* VLO0609	-	-	-	-	-	-	-	-	-	-	-	-	-	-	-	-	-	-	-	-	-	-	-	-	-	-	-	-	-	-	-	-	-	-	-	-	-	-	-	-	-	-	-	-	-	-	-	-	-	-	-	-	-	-	-	-	-	-	-	-	-	-	-
32	*Valerianella lusitanica* VLU0102	-	-	-	-	-	-	-	-	-	-	-	-	-	-	-	-	-	-	-	-	-	-	-	-	-	-	-	-	-	-	-	-	-	-	-	-	-	-	-	-	-	-	-	-	-	-	-	-	-	-	-	-	-	-	-	-	-	-	-	-	-	-	-
33	*Valerianella microcarpa* VMI0102	-	-	-	-	-	-	-	-	-	-	-	-	-	-	-	-	-	-	-	-	-	-	-	-	-	-	-	-	-	-	-	-	-	-	-	-	-	-	-	-	-	-	-	-	-	-	-	-	-	-	-	-	-	-	-	-	-	-	-	-	-	-	-
34	*Valerianella multidentata* VMU0201	-	-	-	-	-	-	-	-	-	-	-	-	-	-	-	-	-	-	-	-	-	-	-	-	-	-	-	-	-	-	-	-	-	-	-	-	-	-	-	-	-	-	-	-	-	-	-	-	-	-	-	-	-	-	-	-	-	-	-	-	-	-	-
35	*Valerianella multidentata* VMU0301	-	-	-	-	-	-	-	-	-	-	-	-	-	-	-	-	-	-	-	-	-	-	-	-	-	-	-	-	-	-	-	-	-	-	-	-	-	-	-	-	-	-	-	-	-	-	-	-	-	-	-	-	-	-	-	-	-	-	-	-	-	-	-
36	*Valerianella pumila* VPU0103	-	-	-	-	-	-	-	-	-	-	-	-	-	-	-	-	-	-	-	-	-	-	-	-	-	-	-	-	-	-	-	-	-	-	-	-	-	-	-	-	-	-	-	-	-	-	-	-	-	-	-	-	-	-	-	-	-	-	-	-	-	-	-
37	*Valerianella pumila* VPU0304	-	-	-	-	-	-	-	-	-	-	-	-	-	-	-	-	-	-	-	-	-	-	-	-	-	-	-	-	-	-	-	-	-	-	-	-	-	-	-	-	-	-	-	-	-	-	-	-	-	-	-	-	-	-	-	-	-	-	-	-	-	-	-
38	*Valerianella pumila* VPU0402	-	-	-	-	-	-	-	-	-	-	-	-	-	-	-	-	-	-	-	-	-	-	-	-	-	-	-	-	-	-	-	-	-	-	-	-	-	-	-	-	-	-	-	-	-	-	-	-	-	-	-	-	-	-	-	-	-	-	-	-	-	-	-
39	*Valerianella rimosa* VRI0101	-	-	-	-	-	-	-	-	-	-	-	-	-	-	-	-	-	-	-	-	-	-	-	-	-	-	-	-	-	-	-	-	-	-	-	-	-	-	-	-	-	-	-	-	-	-	-	-	-	-	-	-	-	-	-	-	-	-	-	-	-	-	-
40	*Valerianella rimosa* VRI0201	-	-	-	-	-	-	-	-	-	-	-	-	-	-	-	-	-	-	-	-	-	-	-	-	-	-	-	-	-	-	-	-	-	-	-	-	-	-	-	-	-	-	-	-	-	-	-	-	-	-	-	-	-	-	-	-	-	-	-	-	-	-	-

	Position	1324	1325	1326	1327	1328	1329	1330	1331	1332	1333	1334	1335	1336	1337	1338	1339	1340	1341	1342	1343	1344	1345	1346	1347	1348	1349	1350	1351	1352	1353	1354	1355	1356	1357	1358	1359	1360	1361	1362	1363	1364	1365	1366	1367	1368	1369	1370	1371	1372	1373	1374	1375	1376	1377	1378	1379	1380	1381	1382	1383	1384	1385	1386
1	*Centranthus calcitrapae* CC25	T	G	G	T	A	G	A	A	T	C	T	A	T	A	G	T	A	T	T	T	T	A	A	T	G	C	G	T	A	T	A	T	C	C	C	T	G	T	T	T	A	T	A	G	G	T	T	T	C	T	C	C	T	T	T	C	T	T	T	T	C	T	G
2	*Centranthus calcitrapae* CCACC539	.	.	.	.	.	.	.	.	.	.	.	.	.	.	.	.	.	.	.	.	.	.	.	.	.	.	.	.	.	.	.	.	.	.	.	.	.	.	.	.	.	.	.	.	.	.	.	.	.	.	.	.	.	.	.	.	.	.	.	.	.	.	.
3	*Fedia cornucopiae* FC73	-	-	-	-	-	-	-	-	-	-	-	-	-	-	-	-	-	-	-	-	-	-	-	-	-	-	-	-	-	-	-	-	-	-	-	-	-	-	-	-	-	.	.	.	.	.	.	.	.	.	.	.	.	.	.	.	.	.	.	.	.	.	.
4	*Valerianella carinata* VCA0104	-	-	-	-	-	-	-	-	-	-	-	-	-	-	-	-	-	-	-	-	-	-	-	-	-	-	-	-	-	-	-	-	-	-	-	-	-	-	-	-	-	.	.	.	.	.	.	.	.	.	.	.	.	.	.	.	.	.	.	.	.	.	.
5	*Valerianella carinata* VCA0202	-	-	-	-	-	-	-	-	-	-	-	-	-	-	-	-	-	-	-	-	-	-	-	-	-	-	-	-	-	-	-	-	-	-	-	-	-	-	-	-	-	.	.	.	.	.	.	.	.	.	.	.	.	.	.	.	.	.	.	.	.	.	.
6	*Valerianella carinata* VCA0302	-	-	-	-	-	-	-	-	-	-	-	-	-	-	-	-	-	-	-	-	-	-	-	-	-	-	-	-	-	-	-	-	-	-	-	-	-	-	-	-	-	.	.	.	.	.	.	.	.	.	.	.	.	.	.	.	.	.	.	.	.	.	.
7	*Valerianella carinata* VCA0501	-	-	-	-	-	-	-	-	-	-	-	-	-	-	-	-	-	-	-	-	-	-	-	-	-	-	-	-	-	-	-	-	-	-	-	-	-	-	-	-	-	.	.	.	.	.	.	.	.	.	.	.	.	.	.	.	.	.	.	.	.	.	.
8	*Valerianella carinata* VCA0805	-	-	-	-	-	-	-	-	-	-	-	-	-	-	-	-	-	-	-	-	-	-	-	-	-	-	-	-	-	-	-	-	-	-	-	-	-	-	-	-	-	.	.	.	.	.	.	.	.	.	.	.	.	.	.	.	.	.	.	.	.	.	.
9	*Valerianella carinata* VCA0918	-	-	-	-	-	-	-	-	-	-	-	-	-	-	-	-	-	-	-	-	-	-	-	-	-	-	-	-	-	-	-	-	-	-	-	-	-	-	-	-	-	.	.	.	.	.	.	.	.	.	.	.	.	.	.	.	.	.	.	.	.	.	.
10	*Valerianella carinata* VCA1006	-	-	-	-	-	-	-	-	-	-	-	-	-	-	-	-	-	-	-	-	-	-	-	-	-	-	-	-	-	-	-	-	-	-	-	-	-	-	-	-	-	.	.	.	.	.	.	.	.	.	.	.	.	.	.	.	.	.	.	.	.	.	.
11	*Valerianella carinata* VCA1106	-	-	-	-	-	-	-	-	-	-	-	-	-	-	-	-	-	-	-	-	-	-	-	-	-	-	-	-	-	-	-	-	-	-	-	-	-	-	-	-	-	.	.	.	.	.	.	.	.	.	.	.	.	.	.	.	.	.	.	.	.	.	.
12	*Valerianella carinata* VCA1202	-	-	-	-	-	-	-	-	-	-	-	-	-	-	-	-	-	-	-	-	-	-	-	-	-	-	-	-	-	-	-	-	-	-	-	-	-	-	-	-	-	.	.	.	.	.	.	.	.	.	.	.	.	.	.	.	.	.	.	.	.	.	.
13	*Valerianella coronata* VCO0102	-	-	-	-	-	-	-	-	-	-	-	-	-	-	-	-	-	-	-	-	-	-	-	-	-	-	-	-	-	-	-	-	-	-	-	-	-	-	-	-	-	-	-	-	-	-	-	-	-	-	-	-	-	-	-	-	-	-	-	-	-	-	.
14	*Valerianella coronata* VCO0218	-	-	-	-	-	-	-	-	-	-	-	-	-	-	-	-	-	-	-	-	-	-	-	-	-	-	-	-	-	-	-	-	-	-	-	-	-	-	-	-	-	-	-	-	-	-	-	-	-	-	-	-	-	-	-	-	-	-	-	-	-	-	.
15	*Valerianella coronata* VCO0404	-	-	-	-	-	-	-	-	-	-	-	-	-	-	-	-	-	-	-	-	-	-	-	-	-	-	-	-	-	-	-	-	-	-	-	-	-	-	-	-	-	-	-	-	-	-	-	-	-	-	-	-	-	-	-	-	-	-	-	-	-	-	.
16	*Valerianella coronata* VCO0508	-	-	-	-	-	-	-	-	-	-	-	-	-	-	-	-	-	-	-	-	-	-	-	-	-	-	-	-	-	-	-	-	-	-	-	-	-	-	-	-	-	-	-	-	-	-	-	-	-	-	-	-	-	-	-	-	-	-	-	-	-	-	.
17	*Valerianella coronata* VCO0705	-	-	-	-	-	-	-	-	-	-	-	-	-	-	-	-	-	-	-	-	-	-	-	-	-	-	-	-	-	-	-	-	-	-	-	-	-	-	-	-	-	-	-	-	-	-	-	-	-	-	-	-	-	-	-	-	-	-	-	-	-	-	.
18	*Valerianella coronata* VCO0805	-	-	-	-	-	-	-	-	-	-	-	-	-	-	-	-	-	-	-	-	-	-	-	-	-	-	-	-	-	-	-	-	-	-	-	-	-	-	-	-	-	-	-	-	-	-	-	-	-	-	-	-	-	-	-	-	-	-	-	-	-	-	.
19	*Valerianella coronata* VCO0901	-	-	-	-	-	-	-	-	-	-	-	-	-	-	-	-	-	-	-	-	-	-	-	-	-	-	-	-	-	-	-	-	-	-	-	-	-	-	-	-	-	-	-	-	-	-	-	-	-	-	-	-	-	-	-	-	-	-	-	-	-	-	.
20	*Valerianella dentata* VDE0303	-	-	-	-	-	-	-	-	-	-	-	-	-	-	-	-	-	-	-	-	-	-	-	-	-	-	-	-	-	-	-	-	-	-	-	-	-	-	-	-	-	-	-	-	-	-	-	-	-	-	-	-	-	-	-	-	-	-	-	-	-	-	.
21	*Valerianella discoidea* VDI0202	-	-	-	-	-	-	-	-	-	-	-	-	-	-	-	-	-	-	-	-	-	-	-	-	-	-	-	-	-	-	-	-	-	-	-	-	-	-	-	-	-	-	-	-	-	-	-	-	-	-	-	-	-	-	-	-	-	-	-	-	-	-	.
22	*Valerianella discoidea* VDI0412	-	-	-	-	-	-	-	-	-	-	-	-	-	-	-	-	-	-	-	-	-	-	-	-	-	-	-	-	-	-	-	-	-	-	-	-	-	-	-	-	-	-	-	-	-	-	-	-	-	-	-	-	-	-	-	-	-	-	-	-	-	-	.
23	*Valerianella echinata* VEC0101	-	-	-	-	-	-	-	-	-	-	-	-	-	-	-	-	-	-	-	-	-	-	-	-	-	-	-	-	-	-	-	-	-	-	-	-	-	-	-	-	-	-	-	-	-	-	-	-	-	-	-	-	-	-	-	-	-	-	-	-	-	-	.
24	*Valerianella eriocarpa* VER0208	-	-	-	-	-	-	-	-	-	-	-	-	-	-	-	-	-	-	-	-	-	-	-	-	-	-	-	-	-	-	-	-	-	-	-	-	-	-	-	-	-	-	-	-	-	-	-	-	-	-	-	-	-	-	-	-	-	-	-	-	-	-	.
25	*Valerianella eriocarpa* VER0402	-	-	-	-	-	-	-	-	-	-	-	-	-	-	-	-	-	-	-	-	-	-	-	-	-	-	-	-	-	-	-	-	-	-	-	-	-	-	-	-	-	-	-	-	-	-	-	-	-	-	-	-	-	-	-	-	-	-	-	-	-	-	.
26	*Valerianella fusiformis* VFU0103	-	-	-	-	-	-	-	-	-	-	-	-	-	-	-	-	-	-	-	-	-	-	-	-	-	-	-	-	-	-	-	-	-	-	-	-	-	-	-	-	-	.	.	.	.	.	.	.	.	.	.	.	.	.	.	.	.	.	.	.	.	.	.
27	*Valerianella locusta* VLO0103	-	-	-	-	-	-	-	-	-	-	-	-	-	-	-	-	-	-	-	-	-	-	-	-	-	-	-	-	-	-	-	-	-	-	-	-	-	-	-	-	-	.	.	.	.	.	.	.	.	.	.	.	.	.	.	.	.	.	.	.	.	.	.
28	*Valerianella locusta* VLO0305	-	-	-	-	-	-	-	-	-	-	-	-	-	-	-	-	-	-	-	-	-	-	-	-	-	-	-	-	-	-	-	-	-	-	-	-	-	-	-	-	-	.	.	.	.	.	.	.	.	.	.	.	.	.	.	.	.	.	.	.	.	.	.
29	*Valerianella locusta* VLO0409	-	-	-	-	-	-	-	-	-	-	-	-	-	-	-	-	-	-	-	-	-	-	-	-	-	-	-	-	-	-	-	-	-	-	-	-	-	-	-	-	-	.	.	.	.	.	.	.	.	.	.	.	.	.	.	.	.	.	.	.	.	.	.
30	*Valerianella locusta* VLO0505	-	-	-	-	-	-	-	-	-	-	-	-	-	-	-	-	-	-	-	-	-	-	-	-	-	-	-	-	-	-	-	-	-	-	-	-	-	-	-	-	-	.	.	.	.	.	.	.	.	.	.	.	.	.	.	.	.	.	.	.	.	.	.
31	*Valerianella locusta* VLO0609	-	-	-	-	-	-	-	-	-	-	-	-	-	-	-	-	-	-	-	-	-	-	-	-	-	-	-	-	-	-	-	-	-	-	-	-	-	-	-	-	-	.	.	.	.	.	.	.	.	.	.	.	.	.	.	.	.	.	.	.	.	.	.
32	*Valerianella lusitanica* VLU0102	-	-	-	-	-	-	-	-	-	-	-	-	-	-	-	-	-	-	-	-	-	-	-	-	-	-	-	-	-	-	-	-	-	-	-	-	-	-	-	-	-	.	.	.	.	.	.	.	.	.	.	.	.	.	.	.	.	.	.	.	.	.	.
33	*Valerianella microcarpa* VMI0102	-	-	-	-	-	-	-	-	-	-	-	-	-	-	-	-	-	-	-	-	-	-	-	-	-	-	-	-	-	-	-	-	-	-	-	-	-	-	-	-	-	-	-	-	-	-	-	-	-	-	-	-	-	-	-	-	-	-	-	-	-	-	.
34	*Valerianella multidentata* VMU0201	-	-	-	-	-	-	-	-	-	-	-	-	-	-	-	-	-	-	-	-	-	-	-	-	-	-	-	-	-	-	-	-	-	-	-	-	-	-	-	-	-	-	-	-	-	-	-	-	-	-	-	-	-	-	-	-	-	-	-	-	-	-	.
35	*Valerianella multidentata* VMU0301	-	-	-	-	-	-	-	-	-	-	-	-	-	-	-	-	-	-	-	-	-	-	-	-	-	-	-	-	-	-	-	-	-	-	-	-	-	-	-	-	-	-	-	-	-	-	-	-	-	-	-	-	-	-	-	-	-	-	-	-	-	-	.
36	*Valerianella pumila* VPU0103	-	-	-	-	-	-	-	-	-	-	-	-	-	-	-	-	-	-	-	-	-	-	-	-	-	-	-	-	-	-	-	-	-	-	-	-	-	-	-	-	-	-	-	-	-	-	-	-	-	-	-	-	-	-	-	-	-	-	-	-	-	-	.
37	*Valerianella pumila* VPU0304	-	-	-	-	-	-	-	-	-	-	-	-	-	-	-	-	-	-	-	-	-	-	-	-	-	-	-	-	-	-	-	-	-	-	-	-	-	-	-	-	-	-	-	-	-	-	-	-	-	-	-	-	-	-	-	-	-	-	-	-	-	-	.
38	*Valerianella pumila* VPU0402	-	-	-	-	-	-	-	-	-	-	-	-	-	-	-	-	-	-	-	-	-	-	-	-	-	-	-	-	-	-	-	-	-	-	-	-	-	-	-	-	-	-	-	-	-	-	-	-	-	-	-	-	-	-	-	-	-	-	-	-	-	-	.
39	*Valerianella rimosa* VRI0101	-	-	-	-	-	-	-	-	-	-	-	-	-	-	-	-	-	-	-	-	-	-	-	-	-	-	-	-	-	-	-	-	-	-	-	-	-	-	-	-	-	-	-	-	-	-	-	-	-	-	-	-	-	-	-	-	-	-	-	-	-	-	.
40	*Valerianella rimosa* VRI0201	-	-	-	-	-	-	-	-	-	-	-	-	-	-	-	-	-	-	-	-	-	-	-	-	-	-	-	-	-	-	-	-	-	-	-	-	-	-	-	-	-	-	-	-	-	-	-	-	-	-	-	-	-	-	-	-	-	-	-	-	-	-	.

Position

		1387	1388	1389	1390	1391	1392	1393	1394	1395	1396	1397	1398	1399	1400	1401	1402	1403	1404	1405	1406	1407	1408	1409	1410	1411	1412	1413	1414	1415	1416	1417	1418	1419	1420	1421	1422	1423	1424	1425	1426	1427	1428	1429	1430	1431	1432	1433	1434	1435	1436	1437	1438	1439	1440	1441	1442	1443	1444	1445	1446	1447	1448	1449
1	*Centranthus calcitrapae* CC25	A	A	G	T	T	T	C	T	A	T	G	G	A	A	G	G	A	T	T	C	T	T	T	T	T	A	C	C	A	A	C	C	C	A	G	T	C	A	A	C	T	C	T	A	T	T	T	G	T	T	A	G	A	A	C	A	G	C	T	T	C	C	G
2	*Centranthus calcitrapae* CCACC539	.	.	.	.	.	.	.	.	.	.	.	.	.	.	.	.	.	.	.	.	.	.	.	.	.	.	.	.	.	.	.	.	.	.	.	.	.	.	.	.	.	.	.	.	.	.	.	.	.	.	.	.	.	.	.	.	.	.	.	.	.	.	.
3	*Fedia cornucopiae* FC73	.	.	.	.	.	.	.	.	.	.	.	.	.	.	.	.	.	.	.	.	.	.	.	.	.	.	.	.	.	.	.	A	.	.	.	.	T	.	.	.	.	.	C	.	.	.	.	.	.	.	.	.	.	.	.	.	.	.	.	.	.	.	.
4	*Valerianella carinata* VCA0104	.	.	.	.	.	.	.	.	.	.	.	.	.	.	.	.	.	.	.	T	.	.	.	.	.	.	.	.	.	.	.	A	.	.	.	.	T	.	.	.	.	.	.	.	.	.	.	.	.	.	.	.	.	.	.	.	.	.	.	.	.	.	.
5	*Valerianella carinata* VCA0202	.	.	.	.	.	.	.	.	.	.	.	.	.	.	.	.	.	.	.	T	.	.	.	.	.	.	.	.	.	.	T	A	.	.	.	.	T	.	.	.	.	.	.	.	.	.	.	.	.	.	.	.	.	.	.	.	.	.	.	.	.	.	.
6	*Valerianella carinata* VCA0302	.	.	.	.	.	.	.	.	.	.	.	.	.	.	.	.	.	.	.	T	.	.	.	.	.	.	.	.	.	.	T	A	.	.	.	.	T	.	.	.	.	.	.	.	.	.	.	.	.	.	.	.	.	.	.	.	.	.	.	.	.	.	.
7	*Valerianella carinata* VCA0501	.	.	.	.	.	.	.	.	.	.	.	.	.	.	.	.	.	.	.	T	.	.	.	.	.	.	.	.	.	.	T	A	.	.	.	.	T	.	.	.	.	.	.	.	.	.	.	.	.	.	.	.	.	.	.	.	.	.	.	.	.	.	.
8	*Valerianella carinata* VCA0805	.	.	.	.	.	.	.	.	.	.	.	.	.	.	.	.	.	.	.	T	.	.	.	.	.	.	.	.	.	.	T	A	.	.	.	.	T	.	.	.	.	.	.	.	.	.	.	.	.	.	.	.	.	.	.	.	.	.	.	.	.	.	.
9	*Valerianella carinata* VCA0918	.	.	.	.	.	.	.	.	.	.	.	.	.	.	.	.	.	.	.	T	.	.	.	.	.	.	.	.	.	.	T	A	.	.	.	.	T	.	.	.	.	.	.	.	.	.	.	.	.	.	.	.	.	.	.	.	.	.	.	.	.	.	.
10	*Valerianella carinata* VCA1006	.	.	.	.	.	.	.	.	.	.	.	.	.	.	.	.	.	.	.	T	.	.	.	.	.	.	.	.	.	.	T	A	.	.	.	.	T	.	.	.	.	.	.	.	.	.	.	.	.	.	.	.	.	.	.	.	.	.	.	.	.	.	.
11	*Valerianella carinata* VCA1106	.	.	.	.	.	.	.	.	.	.	.	.	.	.	.	.	.	.	.	T	.	.	.	.	.	.	.	.	.	.	T	A	.	.	.	.	T	.	.	.	.	.	.	.	.	.	.	.	.	.	.	.	.	.	.	.	.	.	.	.	.	.	.
12	*Valerianella carinata* VCA1202	.	.	.	.	.	.	.	.	.	.	.	.	.	.	.	.	.	.	.	T	.	.	.	.	.	.	.	.	.	.	T	A	.	.	.	.	T	.	.	.	.	.	.	.	.	.	.	.	.	.	.	.	.	.	.	.	.	.	.	.	.	.	.
13	*Valerianella coronata* VCO0102	.	.	.	.	.	.	A	.	.	.	.	.	.	.	.	.	.	.	.	.	.	.	.	.	.	.	A	.	.	.	.	A	.	.	.	.	T	.	.	.	.	.	C	.	.	.	.	.	.	.	.	.	.	.	.	.	.	.	.	.	.	.	.
14	*Valerianella coronata* VCO0218	.	.	.	.	.	.	A	.	.	.	.	.	.	.	.	.	.	.	.	.	.	.	.	.	.	.	A	.	.	.	.	A	.	.	.	.	T	.	.	.	.	.	C	.	.	.	.	.	.	.	.	.	.	.	.	.	.	.	.	.	.	.	.
15	*Valerianella coronata* VCO0404	.	.	.	.	.	.	A	.	.	.	.	.	.	.	.	.	.	.	.	.	.	.	.	.	.	.	A	.	.	.	.	A	.	.	.	.	T	.	.	.	.	.	C	.	.	.	.	.	.	.	.	.	.	.	.	.	.	.	.	.	.	.	.
16	*Valerianella coronata* VCO0508	.	.	.	.	.	.	A	.	.	.	.	.	.	.	.	.	.	.	.	.	.	.	.	.	.	.	A	.	.	.	.	A	.	.	.	.	T	.	.	.	.	.	C	.	.	.	.	.	.	.	.	.	.	.	.	.	.	.	.	.	.	.	.
17	*Valerianella coronata* VCO0705	.	.	.	.	.	.	A	.	.	.	.	.	.	.	.	.	.	.	.	.	.	.	.	.	.	.	A	.	.	.	.	A	.	.	.	.	T	.	.	.	.	.	C	.	.	.	.	.	.	.	.	.	.	.	.	.	.	.	.	.	.	.	.
18	*Valerianella coronata* VCO0805	.	.	.	.	.	.	A	.	.	.	.	.	.	.	.	.	.	.	.	.	.	.	.	.	.	.	A	.	.	.	.	A	.	.	.	.	T	.	.	.	.	.	C	.	.	.	.	.	.	.	.	.	.	.	.	.	.	.	.	.	.	.	.
19	*Valerianella coronata* VCO0901	.	.	.	.	.	.	A	.	.	.	.	.	.	.	.	.	.	.	.	.	.	.	.	.	.	.	A	.	.	.	.	A	.	.	.	.	T	.	.	.	.	.	C	.	.	.	.	.	.	.	.	.	.	.	.	.	.	.	.	.	.	.	.
20	*Valerianella dentata* VDE0303	.	.	.	.	.	.	.	.	.	.	.	.	.	.	.	.	.	.	.	T	.	.	.	.	.	.	.	.	.	.	.	A	.	.	.	.	T	.	.	.	.	.	C	.	.	.	.	.	C	.	.	.	.	.	.	.	.	.	.	.	.	.	.
21	*Valerianella discoidea* VDI0202	.	.	.	.	.	.	.	.	.	.	.	.	.	.	.	.	.	.	.	.	.	.	.	.	.	.	A	.	.	.	.	A	.	.	.	.	T	.	.	.	.	.	C	.	.	.	.	.	.	.	.	.	.	.	.	.	.	.	.	.	.	.	.
22	*Valerianella discoidea* VDI0412	.	.	.	.	.	.	.	.	.	.	.	.	.	.	.	.	.	.	.	.	.	.	.	.	.	.	A	.	.	.	.	A	.	.	.	.	T	.	.	.	.	.	C	.	.	.	.	.	.	.	.	.	.	.	.	.	.	.	.	.	.	.	.
23	*Valerianella echinata* VEC0101	.	.	.	.	.	.	.	.	.	.	.	.	.	.	.	.	.	.	.	T	.	.	.	.	.	.	.	.	.	.	.	A	.	.	.	.	T	.	.	.	.	.	.	.	.	.	.	.	.	.	.	.	.	.	.	.	.	.	.	.	.	.	.
24	*Valerianella eriocarpa* VER0208	.	.	.	.	.	.	.	.	.	.	.	.	.	.	.	.	.	.	.	T	.	.	.	.	.	.	.	.	.	.	.	A	.	.	.	.	T	.	.	.	.	.	C	.	.	.	.	.	.	.	.	.	.	.	.	.	.	.	.	.	.	.	.
25	*Valerianella eriocarpa* VER0402	.	.	.	.	.	.	.	.	.	.	.	.	.	.	.	.	.	.	.	T	.	.	.	.	.	.	.	.	.	.	.	A	.	.	.	.	T	.	.	.	.	.	C	.	.	.	.	.	.	.	.	.	.	.	.	.	.	.	.	.	.	.	.
26	*Valerianella fusiformis* VFU0103	.	.	.	.	.	.	.	.	.	.	.	.	.	.	.	.	.	.	.	.	.	.	.	.	.	.	.	.	.	.	.	A	.	.	.	.	T	.	.	.	.	.	C	G	.	.	.	.	.	.	.	.	.	.	.	.	.	.	.	.	.	.	.
27	*Valerianella locusta* VLO0103	.	.	.	.	.	.	.	.	.	.	.	.	.	.	.	.	.	.	.	T	.	.	.	.	.	.	.	.	.	.	T	A	.	.	.	.	T	.	.	.	.	.	.	.	.	.	.	.	.	.	.	.	.	.	.	.	.	.	.	.	.	.	.
28	*Valerianella locusta* VLO0305	.	.	.	.	.	.	.	.	.	.	.	.	.	.	.	.	.	.	.	T	.	.	.	.	.	.	.	.	.	.	T	A	.	.	.	.	T	.	.	.	.	.	.	.	.	.	.	.	.	.	.	.	.	.	.	.	.	.	.	.	.	.	.
29	*Valerianella locusta* VLO0409	.	.	.	.	.	.	.	.	.	.	.	.	.	.	.	.	.	.	.	T	.	.	.	.	.	.	.	.	.	.	T	A	.	.	.	.	T	.	.	.	.	.	.	.	.	.	.	.	.	.	.	.	.	.	.	.	.	.	.	.	.	.	.
30	*Valerianella locusta* VLO0505	.	.	.	.	.	.	.	.	.	.	.	.	.	.	.	.	.	.	.	T	.	.	.	.	.	.	.	.	.	.	T	A	.	.	.	.	T	.	.	.	.	.	.	.	.	.	.	.	.	.	.	.	.	.	.	.	.	.	.	.	.	.	.
31	*Valerianella locusta* VLO0609	.	.	.	.	.	.	.	.	.	.	.	.	.	.	.	.	.	.	.	T	.	.	.	.	.	.	.	.	.	.	T	A	.	.	.	.	T	.	.	.	.	.	.	.	.	.	.	.	.	.	.	.	.	.	.	.	.	.	.	.	.	.	.
32	*Valerianella lusitanica* VLU0102	.	.	.	.	.	.	.	.	.	.	.	.	.	.	.	.	.	.	.	T	.	.	.	.	.	.	.	.	.	.	.	A	.	.	.	.	T	.	.	.	.	.	.	.	.	.	.	.	.	.	.	.	.	.	.	.	.	.	.	.	.	.	.
33	*Valerianella microcarpa* VMI0102	.	.	.	.	.	.	.	.	.	.	.	.	.	.	.	.	.	.	.	T	.	.	.	.	.	.	.	.	.	.	.	A	.	.	.	.	T	.	.	.	.	.	C	.	.	.	.	.	.	.	.	.	.	.	.	.	.	.	.	.	.	.	.
34	*Valerianella multidentata* VMU0201	.	.	.	.	.	.	.	.	.	.	.	.	.	.	.	.	.	.	.	.	.	.	.	.	.	.	A	.	.	.	.	A	.	.	.	.	T	.	.	.	.	.	C	.	.	.	.	.	.	.	.	.	.	.	.	.	.	.	.	.	.	.	.
35	*Valerianella multidentata* VMU0301	.	.	.	.	.	.	.	.	.	.	.	.	.	.	.	.	.	.	.	.	.	.	.	.	.	.	A	.	.	.	.	A	.	.	.	.	T	.	.	.	.	.	C	.	.	.	.	.	.	.	.	.	.	.	.	.	.	.	.	.	.	.	.
36	*Valerianella pumila* VPU0103	.	.	.	.	.	.	A	.	.	.	.	.	.	.	.	.	.	.	.	.	.	.	.	.	.	.	A	.	.	.	.	A	.	.	.	.	T	.	.	.	.	.	C	.	.	.	.	.	.	.	.	.	.	.	.	.	.	.	.	.	.	.	.
37	*Valerianella pumila* VPU0304	.	.	.	.	.	.	A	.	.	.	.	.	.	.	.	.	.	.	.	.	.	.	.	.	.	.	A	.	.	.	.	A	.	.	.	.	T	.	.	.	.	.	C	.	.	.	.	.	.	.	.	.	.	.	.	.	.	.	.	.	.	.	.
38	*Valerianella pumila* VPU0402	.	.	.	.	.	.	A	.	.	.	.	.	.	.	.	.	.	.	.	.	.	.	.	.	.	.	A	.	.	.	.	A	.	.	.	.	T	.	.	.	.	.	C	.	.	.	.	.	.	.	.	.	.	.	.	.	.	.	.	.	.	.	.
39	*Valerianella rimosa* VRI0101	.	.	.	.	.	.	.	.	.	.	.	.	.	.	.	.	.	.	.	T	.	.	.	.	.	.	.	.	.	.	.	A	.	.	.	.	T	.	.	.	.	.	C	.	.	.	.	.	C	.	.	.	.	.	.	.	.	.	.	.	.	.	.
40	*Valerianella rimosa* VRI0201	.	.	.	.	.	.	.	.	.	.	.	.	.	.	.	.	.	.	.	T	.	.	.	.	.	.	.	.	.	.	.	A	.	.	.	.	T	.	.	.	.	.	C	.	.	.	.	.	C	.	.	.	.	.	.	.	.	.	.	.	.	.	.

Position

No.	Taxon	1450	1451	1452	1453	1454	1455	1456	1457	1458	1459	1460	1461	1462	1463	1464	1465	1466	1467	1468	1469	1470	1471	1472	1473	1474	1475	1476	1477	1478	1479	1480	1481	1482	1483	1484	1485	1486	1487	1488	1489	1490	1491	1492	1493	1494	1495	1496	1497	1498	1499	1500	1501	1502	1503	1504	1505	1506	1507	1508	1509	1510	1511	1512
1	*Centranthus calcitrapae* CC25	T	T	G	A	G	T	C	T	C	T	G	C	A	C	C	T	A	T	C	C	T	T	T	T	T	T	G	T	G	A	T	T	T	T	A	G	C	C	T	T	T	-	G	T	T	-	-	-	-	T	G	T	T	T	T	T	G	G	A	A	A	A	C
2	*Centranthus calcitrapae* CCACC539	.	.	.	.	.	.	.	.	.	.	.	.	.	.	.	.	.	.	.	.	.	.	.	.	.	.	.	.	.	.	.	.	.	.	.	.	.	.	.	.	.	-	.	.	.	-	-	-	-	.	.	.	.	.	.	.	.	.	.	.	.	.	.
3	*Fedia cornucopiae* FC73	.	.	.	.	.	.	.	.	.	.	.	.	.	.	.	.	.	.	.	.	C	.	.	.	.	.	-	-	.	.	.	.	.	.	.	.	.	.	.	.	.	-	.	.	.	C	G	C	T	.	.	.	.	.	.	C	C	.	.	.	.	.	.
4	*Valerianella carinata* VCA0104	.	.	.	.	.	.	.	.	.	.	.	.	.	.	.	.	.	.	.	.	.	.	.	.	.	.	-	-	C	.	.	.	.	.	.	.	.	.	.	.	.	-	.	.	.	-	-	-	-	.	.	.	.	.	.	C	C	.	.	.	.	.	.
5	*Valerianella carinata* VCA0202	.	.	.	.	.	.	.	.	.	.	.	.	.	.	.	.	.	.	.	.	.	.	.	.	.	.	-	-	C	.	.	.	.	.	.	.	.	.	.	.	.	-	.	.	.	-	-	-	-	.	.	.	.	.	.	C	C	.	.	.	.	.	.
6	*Valerianella carinata* VCA0302	.	.	.	.	.	.	.	.	.	.	.	.	.	.	.	.	.	.	.	.	.	.	.	.	.	.	-	-	C	.	.	.	.	.	.	.	.	.	.	.	.	-	.	.	.	-	-	-	-	.	.	.	.	.	.	C	C	.	G	.	.	.	.
7	*Valerianella carinata* VCA0501	.	.	.	.	.	.	.	.	.	.	.	.	.	.	.	.	.	.	.	.	.	.	.	.	.	.	-	-	C	.	.	.	.	.	.	.	.	.	.	.	.	-	.	.	.	-	-	-	-	.	.	.	.	.	.	C	C	.	.	.	.	.	.
8	*Valerianella carinata* VCA0805	.	.	.	.	.	.	.	.	.	.	.	.	.	.	.	.	.	.	.	.	.	.	.	.	.	.	-	-	C	.	.	.	.	.	.	.	.	.	.	.	.	-	.	.	.	-	-	-	-	.	.	.	.	.	.	C	C	.	.	.	.	.	.
9	*Valerianella carinata* VCA0918	.	.	.	.	.	.	.	.	.	.	.	.	.	.	.	.	.	.	.	.	.	.	.	.	.	.	-	-	C	.	.	.	.	.	.	.	.	.	.	.	.	-	.	.	.	-	-	-	-	.	.	.	.	.	.	C	C	.	.	.	.	.	.
10	*Valerianella carinata* VCA1006	.	.	.	.	.	.	.	.	.	.	.	.	.	.	.	.	.	.	.	.	.	.	.	.	.	.	-	-	C	.	.	.	.	.	.	.	.	.	.	.	.	-	.	.	.	-	-	-	-	.	.	.	.	.	.	C	C	.	.	.	.	.	.
11	*Valerianella carinata* VCA1106	.	.	.	.	.	.	.	.	.	.	.	.	.	.	.	.	.	.	.	.	.	.	.	.	.	.	-	-	C	.	.	.	.	.	.	.	.	.	.	.	.	-	.	.	.	-	-	-	-	.	.	.	.	.	.	C	C	.	.	.	.	.	.
12	*Valerianella carinata* VCA1202	.	.	.	.	.	.	.	.	.	.	.	.	.	.	.	.	.	.	.	.	.	.	.	.	.	.	-	-	C	.	.	.	.	.	.	.	.	.	.	.	.	-	.	.	.	-	-	-	-	.	.	.	.	.	.	C	C	.	.	.	.	.	.
13	*Valerianella coronata* VCO0102	.	.	.	.	.	.	.	.	.	.	.	.	.	.	.	.	.	.	.	.	.	.	.	.	.	.	-	-	.	.	.	.	.	.	.	.	.	.	.	.	.	-	.	.	.	-	-	-	-	.	.	.	.	.	.	C	.	.	.	.	.	.	.
14	*Valerianella coronata* VCO0218	.	.	.	.	.	.	.	.	.	.	.	.	.	.	.	.	.	.	.	.	.	.	.	.	.	.	-	-	.	.	.	.	.	.	.	.	.	.	.	.	.	-	.	.	.	-	-	-	-	.	.	.	.	.	.	C	.	.	.	.	.	.	.
15	*Valerianella coronata* VCO0404	.	.	.	.	.	.	.	.	.	.	.	.	.	.	.	.	.	.	.	.	.	.	.	.	.	.	-	-	.	.	.	.	.	.	.	.	.	.	.	.	.	-	.	.	.	-	-	-	-	.	.	.	.	.	.	C	.	.	.	.	.	.	.
16	*Valerianella coronata* VCO0508	.	.	.	.	.	.	.	.	.	.	.	.	.	.	.	.	.	.	.	.	.	.	.	.	.	.	-	-	.	.	.	.	.	.	.	.	.	.	.	.	.	-	.	.	.	-	-	-	-	.	.	.	.	.	.	C	.	.	.	.	.	.	.
17	*Valerianella coronata* VCO0705	.	.	.	.	.	.	.	.	.	.	.	.	.	.	.	.	.	.	.	.	.	.	.	.	.	.	-	-	.	.	.	.	.	.	.	.	.	.	.	.	.	A	.	.	.	-	-	-	-	.	.	.	.	.	.	C	.	.	.	.	.	.	.
18	*Valerianella coronata* VCO0805	.	.	.	.	.	.	.	.	.	.	.	.	.	.	.	.	.	.	.	.	.	.	.	.	.	.	-	-	.	.	.	.	.	.	.	.	.	.	.	.	.	-	.	.	.	-	-	-	-	.	.	.	.	.	.	C	.	.	.	.	.	.	.
19	*Valerianella coronata* VCO0901	.	.	.	.	.	.	.	.	.	.	.	.	.	.	.	.	.	.	.	.	.	.	.	.	.	.	-	-	.	.	.	.	.	.	.	.	.	.	.	.	.	-	.	.	.	-	-	-	-	.	.	.	.	.	.	C	.	.	.	.	.	.	.
20	*Valerianella dentata* VDE0303	.	.	.	.	.	.	.	.	.	.	.	.	.	.	.	.	.	.	.	.	.	.	.	.	.	.	-	-	.	.	.	.	.	.	.	.	.	.	.	.	.	-	.	.	.	-	-	-	-	.	.	.	.	.	.	A	.	.	.	.	.	.	.
21	*Valerianella discoidea* VDI0202	.	.	.	.	.	.	.	.	.	.	.	.	.	.	.	.	.	.	.	.	.	.	.	.	.	.	-	-	.	.	.	.	.	.	.	.	.	.	.	.	.	-	.	.	.	-	-	-	-	.	.	.	.	.	.	C	.	.	.	.	.	.	.
22	*Valerianella discoidea* VDI0412	.	.	.	.	.	.	.	.	.	.	.	.	.	.	.	.	.	.	.	.	.	.	.	.	.	.	-	-	.	.	.	.	.	.	.	.	.	.	.	.	.	-	.	.	.	-	-	-	-	.	.	.	.	.	.	C	.	.	.	.	.	.	.
23	*Valerianella echinata* VEC0101	.	.	.	.	.	.	.	.	.	.	.	.	.	.	.	.	.	.	.	.	.	.	.	.	.	.	-	-	.	.	.	.	.	.	.	.	.	.	.	.	.	-	.	.	.	-	-	-	-	.	.	.	.	.	.	C	C	A	.	.	.	.	.
24	*Valerianella eriocarpa* VER0208	.	.	.	.	.	.	.	.	.	.	.	.	.	.	.	.	.	.	.	.	.	.	.	.	.	.	-	-	.	.	.	.	.	.	.	.	.	.	.	.	.	-	.	.	.	-	-	-	-	.	.	.	.	.	.	A	.	.	.	.	.	.	.
25	*Valerianella eriocarpa* VER0402	.	.	.	.	.	.	.	.	.	.	.	.	.	.	.	.	.	.	.	.	.	.	.	.	.	.	-	-	.	.	.	.	.	.	.	.	.	.	.	.	.	-	.	.	.	-	-	-	-	.	.	.	.	.	.	A	.	.	.	.	.	.	.
26	*Valerianella fusiformis* VFU0103	.	.	.	.	.	.	.	.	.	.	.	.	.	.	.	.	.	.	.	.	.	.	.	.	.	.	-	-	.	.	.	.	.	.	.	.	.	.	.	.	.	C	.	G	.	-	-	-	-	.	.	.	.	.	.	C	.	.	.	.	.	.	.
27	*Valerianella locusta* VLO0103	.	.	.	.	.	.	.	.	.	.	.	.	.	.	.	.	.	.	.	.	.	.	.	.	.	.	-	-	C	.	.	.	.	.	.	.	.	.	.	.	.	-	.	.	.	-	-	-	-	.	.	.	.	.	.	C	C	.	.	.	.	.	.
28	*Valerianella locusta* VLO0305	.	.	.	.	.	.	.	.	.	.	.	.	.	.	.	.	.	.	.	.	.	.	.	.	.	.	-	-	C	.	.	.	.	.	.	.	.	.	.	.	.	-	.	.	.	-	-	-	-	.	.	.	.	.	.	C	C	.	.	.	.	.	.
29	*Valerianella locusta* VLO0409	.	.	.	.	.	.	.	.	.	.	.	.	.	.	.	.	.	.	.	.	.	.	.	.	.	.	-	-	C	.	.	.	.	.	.	.	.	.	.	.	.	-	.	.	.	-	-	-	-	.	.	.	.	.	.	C	C	.	.	.	.	.	.
30	*Valerianella locusta* VLO0505	.	.	.	.	.	.	.	.	.	.	.	.	.	.	.	.	.	.	.	.	.	.	.	.	.	.	-	-	C	.	.	.	.	.	.	.	.	.	.	.	.	-	.	.	.	-	-	-	-	.	.	.	.	.	.	C	C	.	.	.	.	.	.
31	*Valerianella locusta* VLO0609	.	.	.	.	.	.	.	.	.	.	.	.	.	.	.	.	.	.	.	.	.	.	.	.	.	.	-	-	C	.	.	.	.	.	.	.	.	.	.	.	.	-	.	.	.	-	-	-	-	.	.	.	.	.	.	C	C	.	.	.	.	.	.
32	*Valerianella lusitanica* VLU0102	.	.	.	.	.	.	.	.	.	.	.	.	.	.	.	.	.	.	.	.	.	.	.	.	.	.	-	-	C	.	.	.	.	.	.	.	.	.	.	.	.	-	.	.	.	-	-	-	-	.	.	.	.	.	.	C	C	.	.	.	.	.	.
33	*Valerianella microcarpa* VMI0102	.	.	.	.	.	.	.	.	.	.	.	.	.	.	.	.	.	.	.	.	.	.	.	.	.	.	-	-	.	.	.	.	.	.	.	.	.	.	.	.	.	-	.	.	.	-	-	-	-	.	.	.	.	.	.	A	.	.	.	.	.	.	.
34	*Valerianella multidentata* VMU0201	.	.	.	.	.	.	.	.	.	.	.	.	.	.	.	.	.	.	.	.	.	.	.	.	.	.	-	-	.	.	.	.	.	.	.	.	.	.	.	.	.	-	.	.	.	-	-	-	-	.	.	.	.	.	.	C	.	.	.	.	.	.	.
35	*Valerianella multidentata* VMU0301	.	.	.	.	.	.	.	.	.	.	.	.	.	.	.	.	.	.	.	.	.	.	.	.	.	.	-	-	.	.	.	.	.	.	.	.	.	.	.	.	.	-	.	.	.	-	-	-	-	.	.	.	.	.	.	C	.	.	.	.	.	.	.
36	*Valerianella pumila* VPU0103	.	.	.	.	.	.	.	.	.	.	.	.	.	.	.	.	.	.	.	.	.	.	.	.	.	.	-	-	.	.	.	.	.	.	.	.	.	.	.	.	.	-	.	.	.	-	-	-	-	.	.	.	.	.	.	C	.	.	.	.	.	.	.
37	*Valerianella pumila* VPU0304	.	.	.	.	.	.	.	.	.	.	.	.	.	.	.	.	.	.	.	.	.	.	.	.	.	.	-	-	.	.	.	.	.	.	.	.	.	.	.	.	.	-	.	.	.	-	-	-	-	.	.	.	.	.	.	C	.	.	.	.	.	.	.
38	*Valerianella pumila* VPU0402	.	.	.	.	.	.	.	.	.	.	.	.	.	.	.	.	.	.	.	.	.	.	.	.	.	.	-	-	.	.	.	.	.	.	.	.	.	.	.	.	.	-	.	.	.	-	-	-	-	.	.	.	.	.	.	C	.	.	.	.	.	.	.
39	*Valerianella rimosa* VRI0101	.	.	.	.	.	.	.	.	.	.	.	.	.	.	.	.	.	.	.	.	.	.	.	.	.	.	-	-	.	.	.	.	.	.	.	.	.	.	.	.	.	-	.	.	.	-	-	-	-	.	.	.	.	.	.	A	.	.	.	.	.	.	.
40	*Valerianella rimosa* VRI0201	.	.	.	.	.	.	.	.	.	.	.	.	.	.	.	.	.	.	.	.	.	.	.	.	.	.	-	-	.	.	.	.	.	.	.	.	.	.	.	.	.	-	.	.	.	-	-	-	-	.	.	.	.	.	.	A	.	.	.	.	.	.	.

Position

		1513	1514	1515	1516	1517	1518	1519	1520	1521	1522	1523	1524	1525	1526	1527	1528	1529	1530	1531	1532	1533	1534	1535	1536	1537	1538	1539	1540	1541	1542	1543	1544	1545	1546	1547	1548	1549	1550	1551	1552	1553	1554	1555	1556	1557	1558	1559	1560	1561	1562	1563	1564	1565	1566	1567	1568	1569	1570	1571	1572	1573	1574	1575
1	*Centranthus calcitrapae* CC25	A	A	A	A	A	C	C	G	G	A	T	T	T	G	G	C	T	C	A	G	G	A	T	T	G	C	C	C	A	T	T	T	T	T	A	T	T	A	A	T	T	C	C	A	G	G	G	T	T	T	C	T	C	T	G	A	A	T	T	T	G	A	A
2	*Centranthus calcitrapae* CCACC539	.	.	.	.	.	.	.	.	.	.	.	.	.	.	.	.	.	.	.	.	.	.	.	.	.	.	.	.	.	.	.	.	.	.	.	.	.	.	.	.	.	.	.	.	.	.	.	.	.	.	.	.	.	.	.	.	.	.	.	.	.	.	.
3	*Fedia cornucopiae* FC73	.	.	.	.	.	.	G	.	.	.	.	.	.	.	.	.	.	.	.	.	.	.	.	.	A	.	.	.	.	.	.	.	.	.	-	.	.	.	.	.	.	.	.	.	.	.	.	.	.	.	.	.	.	.	.	.	.	.	.	.	.	.	.
4	*Valerianella carinata* VCA0104	.	.	.	.	.	.	G	.	.	.	.	.	.	.	.	.	.	.	.	.	.	.	.	.	A	.	.	.	.	.	.	.	.	.	.	.	.	.	.	.	.	.	.	.	.	.	.	.	.	.	.	.	.	.	.	.	.	.	.	.	.	.	.
5	*Valerianella carinata* VCA0202	.	.	.	.	.	.	G	.	.	.	.	.	.	.	.	.	.	.	.	.	.	.	.	.	A	.	.	.	.	.	.	.	.	.	.	.	.	.	.	.	.	.	.	.	.	.	.	.	.	.	.	.	.	.	.	.	.	.	.	.	.	.	.
6	*Valerianella carinata* VCA0302	.	.	.	.	.	.	G	.	.	.	.	.	.	.	.	.	.	.	.	.	.	.	.	.	A	.	.	.	.	.	.	.	.	.	.	.	.	.	.	.	.	.	.	.	.	.	.	.	.	.	.	.	.	.	.	.	.	.	.	.	.	.	.
7	*Valerianella carinata* VCA0501	.	.	.	.	.	.	G	.	.	.	.	.	.	.	.	.	.	.	.	.	.	.	.	.	A	.	.	.	.	.	.	.	.	.	.	.	.	.	.	.	.	.	.	.	.	.	.	.	.	.	.	.	.	.	.	.	.	.	.	.	.	.	.
8	*Valerianella carinata* VCA0805	.	.	.	.	.	.	G	.	.	.	.	.	.	.	.	.	.	.	.	.	.	.	.	.	A	.	.	.	.	.	.	.	.	.	.	.	.	.	.	.	.	.	.	.	.	.	.	.	.	.	.	.	.	.	.	.	.	.	.	.	.	.	.
9	*Valerianella carinata* VCA0918	.	.	.	.	.	.	G	.	.	.	.	.	.	.	.	.	.	.	.	.	.	.	.	.	A	.	.	.	.	.	.	.	.	.	.	.	.	.	.	.	.	.	.	.	.	.	.	.	.	.	.	.	.	.	.	.	.	.	.	.	.	.	.
10	*Valerianella carinata* VCA1006	.	.	.	.	.	.	G	.	.	.	.	.	.	.	.	.	.	.	.	.	.	.	.	.	A	.	.	.	.	.	.	.	.	.	.	.	.	.	.	.	.	.	.	.	.	.	.	.	.	.	.	.	.	.	.	.	.	.	.	.	.	.	.
11	*Valerianella carinata* VCA1106	.	.	.	.	.	.	G	.	.	.	.	.	.	.	.	.	.	.	.	.	.	.	.	.	A	.	.	.	.	.	.	.	.	.	.	.	.	.	.	.	.	.	.	.	.	.	.	.	.	.	.	.	.	.	.	.	.	.	.	.	.	.	.
12	*Valerianella carinata* VCA1202	.	.	.	.	.	.	G	.	.	.	.	.	.	.	.	.	.	.	.	.	.	.	.	.	A	.	.	.	.	.	.	.	.	.	.	.	.	.	.	.	.	.	.	.	.	.	.	.	.	.	.	.	.	.	.	.	.	.	.	.	.	.	.
13	*Valerianella coronata* VCO0102	.	.	.	.	.	.	G	.	.	.	.	.	.	.	.	.	.	.	.	.	.	.	.	.	A	.	.	.	.	.	.	.	.	.	.	.	.	.	.	.	.	.	.	.	.	.	.	.	.	.	.	.	.	.	.	.	.	.	.	.	.	.	.
14	*Valerianella coronata* VCO0218	.	.	.	.	.	.	G	.	.	.	.	.	.	.	.	.	.	.	.	.	.	.	.	.	A	.	.	.	.	.	.	.	.	.	.	.	.	.	.	.	.	.	.	.	.	.	.	.	.	.	.	.	.	.	.	.	.	.	.	.	.	.	.
15	*Valerianella coronata* VCO0404	.	.	.	.	.	.	G	.	.	.	.	.	.	.	.	.	.	.	.	.	.	.	.	.	A	.	.	.	.	.	.	.	.	.	.	.	.	.	.	.	.	.	.	.	.	.	.	.	.	.	.	.	.	.	.	.	.	.	.	.	.	.	.
16	*Valerianella coronata* VCO0508	.	.	.	.	.	.	G	.	.	.	.	.	.	.	.	.	.	.	.	.	.	.	.	.	A	.	.	.	.	.	.	.	.	.	.	.	.	.	.	.	.	.	.	.	.	.	.	.	.	.	.	.	.	.	.	.	.	.	.	.	.	.	.
17	*Valerianella coronata* VCO0705	.	.	.	.	.	.	G	.	.	.	.	.	.	.	.	.	.	.	.	.	.	.	.	.	A	.	.	.	.	.	.	.	.	.	.	.	.	.	.	.	.	.	.	.	.	.	.	.	.	.	.	.	.	.	.	.	.	.	.	.	.	.	.
18	*Valerianella coronata* VCO0805	.	.	.	.	.	.	G	.	.	.	.	.	.	.	.	.	.	.	.	.	.	.	.	.	A	.	.	.	.	.	.	.	.	.	.	.	.	.	.	.	.	.	.	.	.	.	.	.	.	.	.	.	.	.	.	.	.	.	.	.	.	.	.
19	*Valerianella coronata* VCO0901	.	.	.	.	.	.	G	.	.	.	.	.	.	.	.	.	.	.	.	.	.	.	.	.	A	.	.	.	.	.	.	.	.	.	.	.	.	.	.	.	.	.	.	.	.	.	.	.	.	.	.	.	.	.	.	.	.	.	.	.	.	.	.
20	*Valerianella dentata* VDE0303	.	.	.	.	.	.	G	.	.	.	.	.	.	.	.	.	.	.	.	.	.	.	.	.	A	.	.	.	.	.	.	.	.	.	.	.	.	.	.	.	.	.	.	.	.	.	.	.	.	.	.	.	.	.	.	.	.	.	.	.	.	.	.
21	*Valerianella discoidea* VDI0202	.	.	.	.	.	.	G	.	.	.	.	.	.	.	.	.	.	.	.	.	.	.	.	.	A	.	.	.	.	.	.	.	.	.	.	.	.	.	.	.	.	.	.	.	.	.	.	.	.	.	.	.	.	.	.	.	.	.	.	.	.	.	.
22	*Valerianella discoidea* VDI0412	.	.	.	.	.	.	G	.	.	.	.	.	.	.	.	.	.	.	.	.	.	.	.	.	A	.	.	.	.	.	.	.	.	.	.	.	.	.	.	.	.	.	.	.	.	.	.	.	.	.	.	.	.	.	.	.	.	.	.	.	.	.	.
23	*Valerianella echinata* VEC0101	.	.	.	.	.	.	.	.	.	.	.	.	.	.	.	.	.	.	.	.	.	.	.	.	A	.	.	.	.	.	.	.	.	.	.	.	.	.	.	.	.	.	.	.	.	.	.	.	.	.	.	.	.	.	.	.	.	.	.	.	.	.	.
24	*Valerianella eriocarpa* VER0208	.	.	.	.	.	.	G	.	.	.	.	.	.	.	.	.	.	.	.	.	.	.	.	.	A	.	.	.	.	.	.	.	.	.	.	.	.	.	.	.	.	.	.	.	.	.	.	.	.	.	.	.	.	.	.	.	.	.	.	.	.	.	.
25	*Valerianella eriocarpa* VER0402	.	.	.	.	.	.	G	.	.	.	.	.	.	.	.	.	.	.	.	.	.	.	.	.	A	.	.	.	.	.	.	.	.	.	.	.	.	.	.	.	.	.	.	.	.	.	.	.	.	.	.	.	.	.	.	.	.	.	.	.	.	.	.
26	*Valerianella fusiformis* VFU0103	.	.	.	.	.	.	G	.	.	.	.	.	.	.	.	.	.	.	.	.	.	.	.	.	A	.	.	.	.	.	.	.	.	.	.	.	.	.	.	.	.	.	.	.	.	.	.	.	.	.	.	.	.	.	.	.	.	.	.	.	.	.	.
27	*Valerianella locusta* VLO0103	.	.	.	.	.	.	G	.	.	.	.	.	.	.	.	.	.	.	.	.	.	.	.	.	A	.	.	.	.	.	.	.	.	.	.	.	.	.	.	.	.	.	.	.	.	.	.	.	.	.	.	.	.	.	.	.	.	.	.	.	.	.	.
28	*Valerianella locusta* VLO0305	.	.	.	.	.	.	G	.	.	.	.	.	.	.	.	.	.	.	.	.	.	.	.	.	A	.	.	.	.	.	.	.	.	.	.	.	.	.	.	.	.	.	.	.	.	.	.	.	.	.	.	.	.	.	.	.	.	.	.	.	.	.	.
29	*Valerianella locusta* VLO0409	.	.	.	.	.	.	G	.	.	.	.	.	.	.	.	.	.	.	.	.	.	.	.	.	A	.	.	.	.	.	.	.	.	.	.	.	.	.	.	.	.	.	.	.	.	.	.	.	.	.	.	.	.	.	.	.	.	.	.	.	.	.	.
30	*Valerianella locusta* VLO0505	.	.	.	.	.	.	G	.	.	.	.	.	.	.	.	.	.	.	.	.	.	.	.	.	A	.	.	.	.	.	.	.	.	.	.	.	.	.	.	.	.	.	.	.	.	.	.	.	.	.	.	.	.	.	.	.	.	.	.	.	.	.	.
31	*Valerianella locusta* VLO0609	.	.	.	.	.	.	G	.	.	.	.	.	.	.	.	.	.	.	.	.	.	.	.	.	A	.	.	.	.	.	.	.	.	.	.	.	.	.	.	.	.	.	.	.	.	.	.	.	.	.	.	.	.	.	.	.	.	.	.	.	.	.	.
32	*Valerianella lusitanica* VLU0102	.	.	.	.	.	.	G	.	.	.	.	.	.	.	.	.	.	.	.	.	.	.	.	.	A	.	.	.	.	.	.	.	.	.	.	.	.	.	.	.	.	.	.	.	.	.	.	.	.	.	.	.	.	.	.	.	.	.	.	.	.	.	.
33	*Valerianella microcarpa* VMI0102	.	.	.	.	.	.	G	.	.	.	.	.	.	.	.	.	.	.	.	.	.	.	.	.	A	.	.	.	.	.	.	.	.	.	.	.	.	.	.	.	.	.	.	.	.	.	.	.	.	.	.	.	.	.	.	.	.	.	.	.	.	.	.
34	*Valerianella multidentata* VMU0201	.	.	.	.	.	.	G	.	.	.	.	.	.	.	.	.	.	.	.	.	.	.	.	.	A	.	.	.	.	.	.	.	.	.	.	.	.	.	.	.	.	.	.	.	.	.	.	.	.	.	.	.	.	.	.	.	.	.	.	.	.	.	.
35	*Valerianella multidentata* VMU0301	.	.	.	.	.	.	G	.	.	.	.	.	.	.	.	.	.	.	.	.	.	.	.	.	A	.	.	.	.	.	.	.	.	.	.	.	.	.	.	.	.	.	.	.	.	.	.	.	.	.	.	.	.	.	.	.	.	.	.	.	.	.	.
36	*Valerianella pumila* VPU0103	.	.	.	.	.	.	G	.	.	.	.	.	.	.	.	.	.	.	.	.	.	.	.	.	A	.	.	.	.	.	.	.	.	.	.	.	.	.	.	.	.	.	.	.	.	.	.	.	.	.	.	.	.	.	.	.	.	.	.	.	.	.	.
37	*Valerianella pumila* VPU0304	.	.	.	.	.	.	G	.	.	.	.	.	.	.	.	.	.	.	.	.	.	.	.	.	A	.	.	.	.	.	.	.	.	.	.	.	.	.	.	.	.	.	.	.	.	.	.	.	.	.	.	.	.	.	.	.	.	.	.	.	.	.	.
38	*Valerianella pumila* VPU0402	.	.	.	.	.	.	G	.	.	.	.	.	.	.	.	.	.	.	.	.	.	.	.	.	A	.	.	.	.	.	.	.	.	.	.	.	.	.	.	.	.	.	.	.	.	.	.	.	.	.	.	.	.	.	.	.	.	.	.	.	.	.	.
39	*Valerianella rimosa* VRI0101	.	.	.	.	.	.	G	.	.	.	.	.	.	.	.	.	.	.	.	.	.	.	.	.	A	.	.	.	.	.	.	.	.	.	.	.	.	.	.	.	.	.	.	.	.	.	.	.	.	.	.	.	.	.	.	.	.	.	.	.	.	.	.
40	*Valerianella rimosa* VRI0201	.	.	.	.	.	.	G	.	.	.	.	.	.	.	.	.	.	.	.	.	.	.	.	.	A	.	.	.	.	.	.	.	.	.	.	.	.	.	.	.	.	.	.	.	.	.	.	.	.	.	.	.	.	.	.	.	.	.	.	.	.	.	.

Position

		1576	1577	1578	1579	1580	1581	1582	1583	1584	1585	1586	1587	1588	1589	1590	1591	1592	1593	1594	1595	1596	1597	1598	1599	1600	1601	1602	1603	1604	1605	1606	1607	1608	1609	1610	1611	1612	1613	1614	1615	1616	1617	1618	1619	1620	1621	1622	1623	1624	1625
1	*Centranthus calcitrapae* CC25	A	G	T	T	C	T	C	A	C	T	T	A	G	T	A	G	G	T	T	T	C	C	A	T	A	C	T	A	A	G	G	C	T	C	A	A	T	C	C	A	A	T	T	A	A	G	T	C	C	G
2	*Centranthus calcitrapae* CCACC539	.	.	.	.	.	.	.	.	.	.	.	.	.	.	.	.	.	.	.	.	.	.	.	.	.	.	.	.	.	.	.	.	.	.	.	.	.	.	.	.	.	.	.	.	.	.	.	.	.	.
3	*Fedia cornucopiae* FC73	.	.	.	.	.	.	.	.	.	.	.	G	.	.	.	.	.	.	.	.	.	.	.	.	.	.	C	.	.	.	.	.	.	.	.	.	.	.	.	.	.	.	.	.	.	.	.	.	.	.
4	*Valerianella carinata* VCA0104	.	.	.	.	.	.	.	.	.	.	.	.	.	.	.	.	.	.	.	.	.	.	.	.	.	.	C	.	.	.	.	.	.	.	.	.	.	.	.	.	.	.	.	.	.	.	.	.	.	.
5	*Valerianella carinata* VCA0202	.	.	.	.	.	.	.	.	.	.	.	.	.	.	.	.	.	.	.	.	.	.	.	.	.	.	C	.	.	.	.	.	.	.	.	.	.	.	.	.	.	.	.	.	.	.	.	.	.	.
6	*Valerianella carinata* VCA0302	.	.	.	.	.	.	.	.	.	.	.	.	.	.	.	.	.	.	.	.	.	.	.	.	.	.	C	.	.	.	.	.	.	.	.	.	.	.	.	.	.	.	.	.	.	.	.	.	.	.
7	*Valerianella carinata* VCA0501	.	.	.	.	.	.	.	.	.	.	.	.	.	.	.	.	.	.	.	.	.	.	.	.	.	.	C	.	.	.	.	.	.	.	.	.	.	.	.	.	.	.	.	.	.	.	.	.	.	.
8	*Valerianella carinata* VCA0805	.	.	.	.	.	.	.	.	.	.	.	.	.	.	.	.	.	.	.	.	.	.	.	.	.	.	C	.	.	.	.	.	.	.	.	.	.	.	.	.	.	.	.	.	.	.	.	.	.	.
9	*Valerianella carinata* VCA0918	.	.	.	.	.	.	.	.	.	.	.	.	.	.	.	.	.	.	.	.	.	.	.	.	.	.	C	.	.	.	.	.	.	.	.	.	.	.	.	.	.	.	.	.	.	.	.	.	.	.
10	*Valerianella carinata* VCA1006	.	.	.	.	.	.	.	.	.	.	.	.	.	.	.	.	.	.	.	.	.	.	.	.	.	.	C	.	.	.	.	.	.	.	.	.	.	.	.	.	.	.	.	.	.	.	.	.	.	.
11	*Valerianella carinata* VCA1106	.	.	.	.	.	.	.	.	.	.	.	.	.	.	.	.	.	.	.	.	.	.	.	.	.	.	C	.	.	.	.	.	.	.	.	.	.	.	.	.	.	.	.	.	.	.	.	.	.	.
12	*Valerianella carinata* VCA1202	.	.	.	.	.	.	.	.	.	.	.	.	.	.	.	.	.	.	.	.	.	.	.	.	.	.	C	.	.	.	.	.	.	.	.	.	.	.	.	.	.	.	.	.	.	.	.	.	.	.
13	*Valerianella coronata* VCO0102	.	.	.	.	.	.	.	.	.	.	.	.	.	.	.	.	.	.	.	.	.	.	.	.	.	.	C	.	.	.	.	.	.	.	.	.	.	.	.	.	.	.	.	.	.	.	.	.	.	.
14	*Valerianella coronata* VCO0218	.	.	.	.	.	.	.	.	.	.	.	.	.	.	.	.	.	.	.	.	.	.	.	.	.	.	C	.	.	.	.	.	.	.	.	.	.	.	.	.	.	.	.	.	.	.	.	.	.	.
15	*Valerianella coronata* VCO0404	.	.	.	.	.	.	.	.	.	.	.	.	.	.	.	.	.	.	.	.	.	.	.	.	.	.	C	.	.	.	.	.	.	.	.	.	.	.	.	.	.	.	.	.	.	.	.	.	.	.
16	*Valerianella coronata* VCO0508	.	.	.	.	.	.	.	.	.	.	.	.	.	.	.	.	.	.	.	.	.	.	.	.	.	.	C	.	.	.	.	.	.	.	.	.	.	.	.	.	.	.	.	.	.	.	.	.	.	.
17	*Valerianella coronata* VCO0705	.	.	.	.	.	.	.	.	.	.	.	.	.	.	.	.	.	.	.	.	.	.	.	.	.	.	C	.	.	.	.	.	.	.	.	.	.	.	.	.	.	.	.	.	.	.	.	.	.	.
18	*Valerianella coronata* VCO0805	.	.	.	.	.	.	.	.	.	.	.	.	.	.	.	.	.	.	.	.	.	.	.	.	.	.	C	.	.	.	.	.	.	.	.	.	.	.	.	.	.	.	.	.	.	.	.	.	.	.
19	*Valerianella coronata* VCO0901	.	.	.	.	.	.	.	.	.	.	.	.	.	.	.	.	.	.	.	.	.	.	.	.	.	.	C	.	.	.	.	.	.	.	.	.	.	.	.	.	.	.	.	.	.	.	.	.	.	.
20	*Valerianella dentata* VDE0303	.	.	.	.	.	.	.	.	.	.	.	.	.	.	.	.	.	.	.	.	.	.	.	.	.	.	C	.	.	.	.	.	.	.	.	.	.	.	.	.	.	.	.	.	.	.	.	.	.	.
21	*Valerianella discoidea* VDI0202	.	.	.	.	.	.	.	.	.	.	.	.	.	.	.	.	.	.	.	.	.	.	.	.	.	.	C	.	.	.	.	.	.	.	.	.	.	.	.	.	.	.	.	.	.	.	.	.	.	.
22	*Valerianella discoidea* VDI0412	.	.	.	.	.	.	.	.	.	.	.	.	.	.	.	.	.	.	.	.	.	.	.	.	.	.	C	.	.	.	.	.	.	.	.	.	.	.	.	.	.	.	.	.	.	.	.	.	.	.
23	*Valerianella echinata* VEC0101	.	.	.	.	.	.	.	.	.	.	.	.	.	.	.	.	.	.	.	.	.	.	.	.	.	.	C	.	.	.	.	.	.	.	.	.	.	.	.	.	.	.	.	.	.	.	.	.	.	.
24	*Valerianella eriocarpa* VER0208	.	.	.	.	.	.	.	.	.	.	.	.	.	.	.	.	.	.	.	.	.	.	.	.	.	.	C	.	.	.	.	.	.	.	.	.	.	.	.	.	.	.	.	.	.	.	.	.	.	.
25	*Valerianella eriocarpa* VER0402	.	.	.	.	.	.	.	.	.	.	.	.	.	.	.	.	.	.	.	.	.	.	.	.	.	.	C	.	.	.	.	.	.	.	.	.	.	.	.	.	.	.	.	.	.	.	.	.	.	.
26	*Valerianella fusiformis* VFU0103	.	.	.	.	A	.	.	.	.	.	.	G	.	.	.	.	.	.	.	.	.	.	.	.	.	.	C	.	.	.	.	.	.	.	.	.	.	.	.	.	.	.	.	.	.	.	.	.	.	.
27	*Valerianella locusta* VLO0103	.	.	.	.	.	.	.	.	.	.	.	.	.	.	.	.	.	.	.	.	.	.	.	.	.	.	C	.	.	.	.	.	.	.	.	.	.	.	.	.	.	.	.	.	.	.	.	.	.	.
28	*Valerianella locusta* VLO0305	.	.	.	.	.	.	.	.	.	.	.	.	.	.	.	.	.	.	.	.	.	.	.	.	.	.	C	.	.	.	.	.	.	.	.	.	.	.	.	.	.	.	.	.	.	.	.	.	.	.
29	*Valerianella locusta* VLO0409	.	.	.	.	.	.	.	.	.	.	.	.	.	.	.	.	.	.	.	.	.	.	.	.	.	.	C	.	.	.	.	.	.	.	.	.	.	.	.	.	.	.	.	.	.	.	.	.	.	.
30	*Valerianella locusta* VLO0505	.	.	.	.	.	.	.	.	.	.	.	.	.	.	.	.	.	.	.	.	.	.	.	.	.	.	C	.	.	.	.	.	.	.	.	.	.	.	.	.	.	.	.	.	.	.	.	.	.	.
31	*Valerianella locusta* VLO0609	.	.	.	.	.	.	.	.	.	.	.	.	.	.	.	.	.	.	.	.	.	.	.	.	.	.	C	.	.	.	.	.	.	.	.	.	.	.	.	.	.	.	.	.	.	.	.	.	.	.
32	*Valerianella lusitanica* VLU0102	.	.	.	.	.	.	.	.	.	.	.	.	.	.	.	.	.	.	.	.	.	.	.	.	.	.	C	.	.	.	.	.	.	.	.	.	.	.	.	.	.	.	.	.	.	.	.	.	.	.
33	*Valerianella microcarpa* VMI0102	.	.	.	.	.	.	.	.	.	.	.	.	.	.	.	.	.	.	.	.	.	.	.	.	.	.	C	.	.	.	.	.	.	.	.	.	.	.	.	.	.	.	.	.	.	.	.	.	.	.
34	*Valerianella multidentata* VMU0201	.	.	.	.	.	.	.	.	.	.	.	.	.	.	.	.	.	.	.	.	.	.	.	.	.	.	C	.	.	.	.	.	.	.	.	.	.	.	.	.	.	.	.	.	.	.	.	.	.	.
35	*Valerianella multidentata* VMU0301	.	.	.	.	.	.	.	.	.	.	.	.	.	.	.	.	.	.	.	.	.	.	.	.	.	.	C	.	.	.	.	.	.	.	.	.	.	.	.	.	.	.	.	.	.	.	.	.	.	.
36	*Valerianella pumila* VPU0103	.	.	.	.	.	.	.	.	.	.	.	.	.	.	.	.	.	.	.	.	.	.	.	.	.	.	C	.	.	.	.	.	.	.	.	.	.	.	.	.	.	.	.	.	.	.	.	.	.	.
37	*Valerianella pumila* VPU0304	.	.	.	.	.	.	.	.	.	.	.	.	.	.	.	.	.	.	.	.	.	.	.	.	.	.	C	.	.	.	.	.	.	.	.	.	.	.	.	.	.	.	.	.	.	.	.	.	.	.
38	*Valerianella pumila* VPU0402	.	.	.	.	.	.	.	.	.	.	.	.	.	.	.	.	.	.	.	.	.	.	.	.	.	.	C	.	.	.	.	.	.	.	.	.	.	.	.	.	.	.	.	.	.	.	.	.	.	.
39	*Valerianella rimosa* VRI0101	.	.	.	.	.	.	.	.	.	.	.	.	.	.	.	.	.	.	.	.	.	.	.	.	.	.	C	.	.	.	.	.	.	.	.	.	.	.	.	.	.	.	.	.	.	.	.	.	.	.
40	*Valerianella rimosa* VRI0201	.	.	.	.	.	.	.	.	.	.	.	.	.	.	.	.	.	.	.	.	.	.	.	.	.	.	C	.	.	.	.	.	.	.	.	.	.	.	.	.	.	.	.	.	.	.	.	.	.	.
