# Supplementary figures and images for: Molecular Systematics of Valerianella Mill. (Caprifoliaceae): Challenging the Taxonomic Value of Genetically Controlled Carpological Traits
Source: Plants (Basel). 2022 May 10;11(10):1276. doi: 10.3390/plants11101276 (PMC9146508; doi:10.3390/plants11101276)

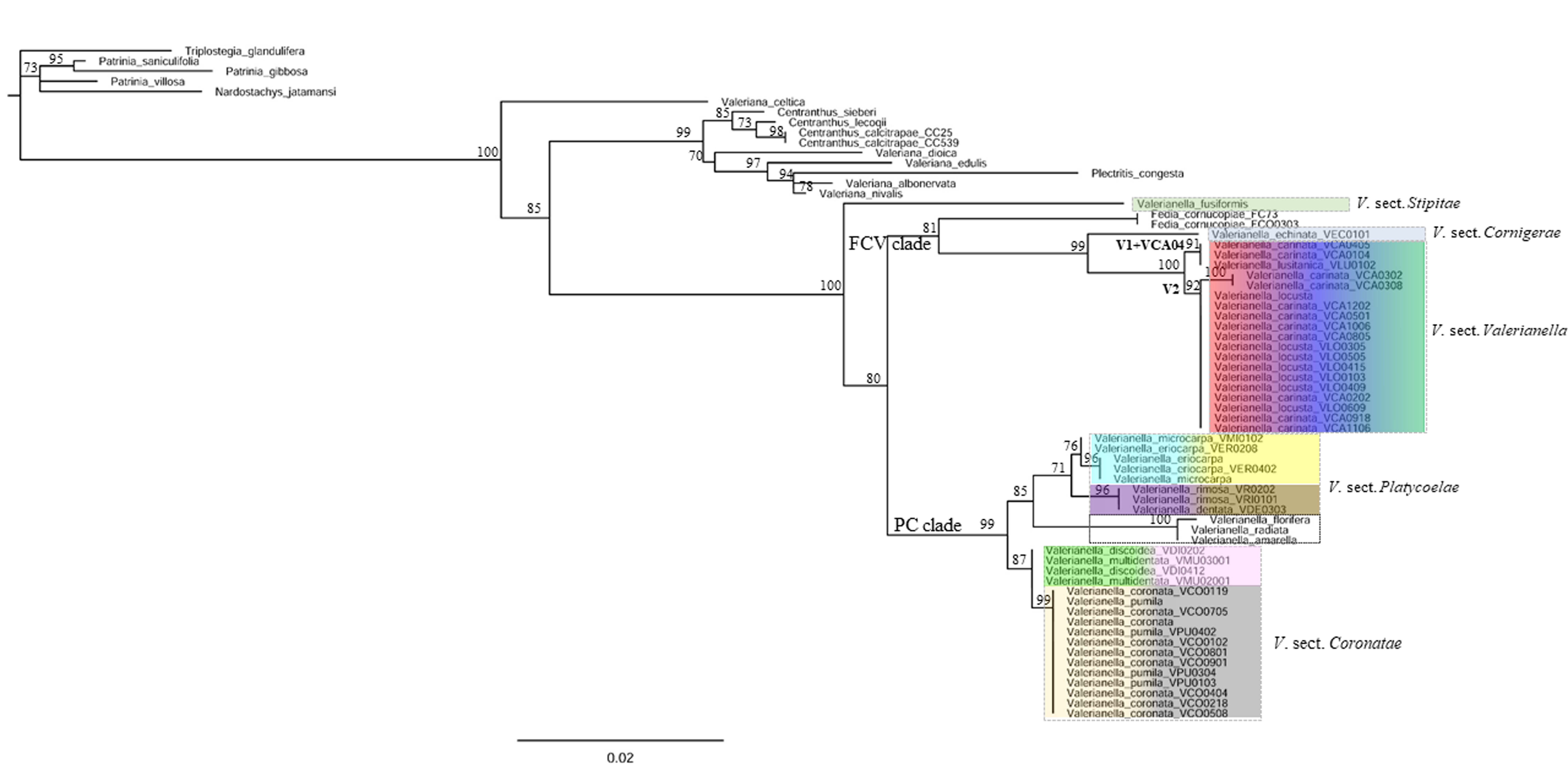

Supplement: Supplementary file 1 [file plants-11-01276-s001.zip › plants-1677153-supplementary/Figures jpg/FigS1.jpg]

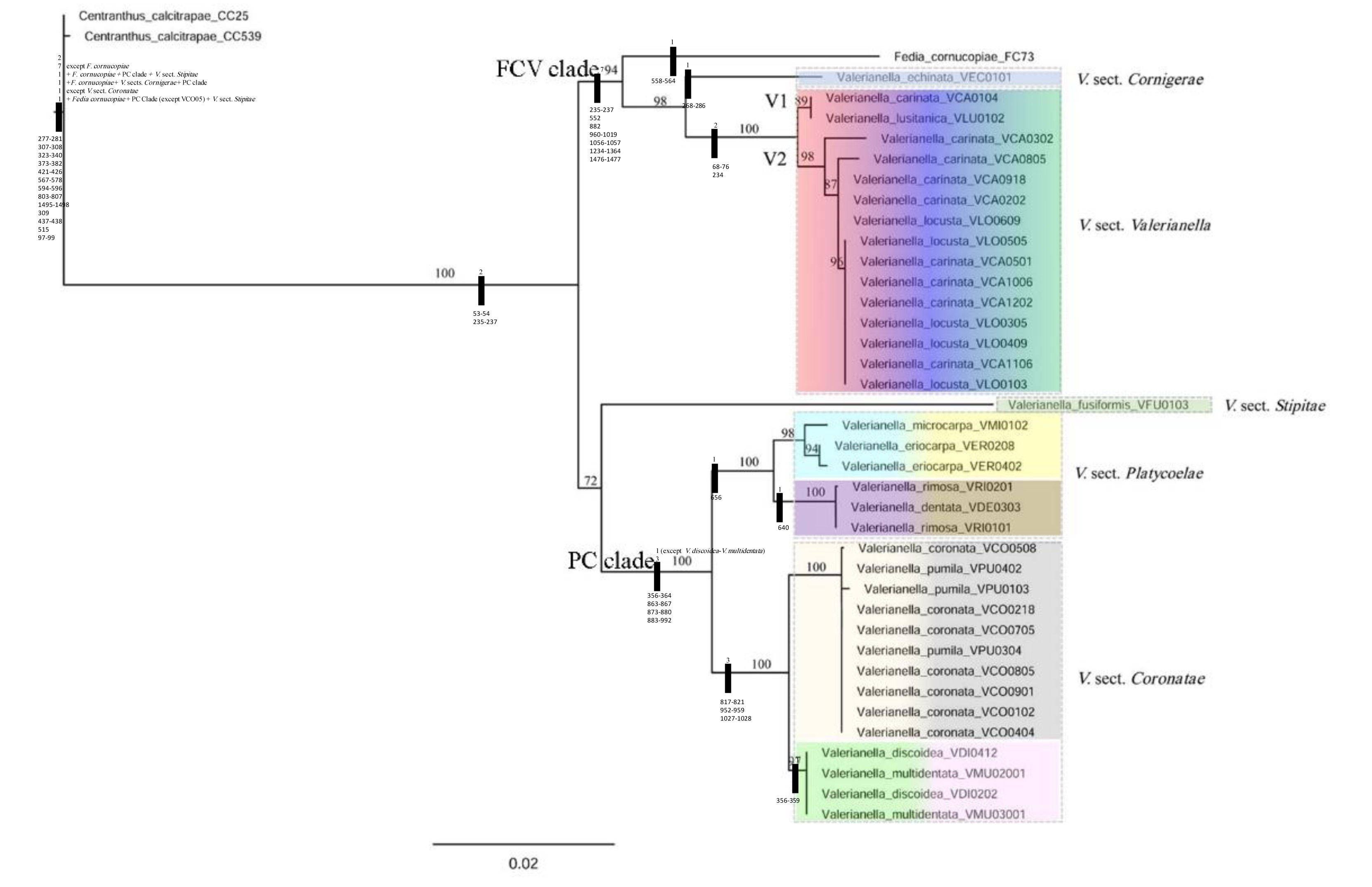

Supplement: Supplementary file 1 [file plants-11-01276-s001.zip › plants-1677153-supplementary/Figures jpg/FigS2.jpg]

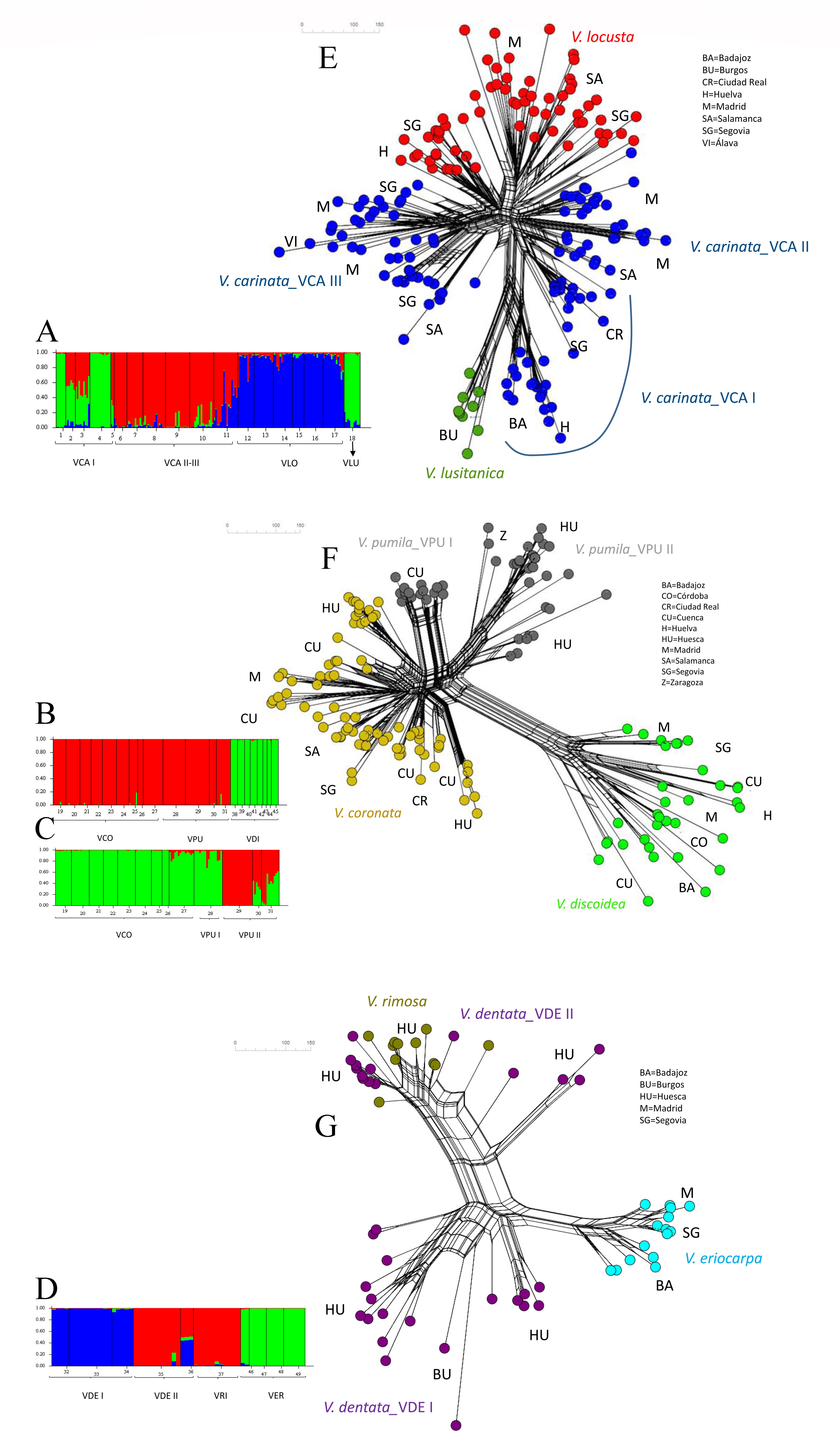

Supplement: Supplementary file 1 [file plants-11-01276-s001.zip › plants-1677153-supplementary/Figures jpg/FigS3.jpg]
